# Supplementary material for: Gene expression profiling of single cells from archival tissue with laser-capture microdissection and Smart-3SEQ
Source: Genome Res. 2019 Nov;29(11):1816–25. doi: 10.1101/gr.234807.118 (PMC6836736; doi:10.1101/gr.234807.118)
Supplement: Supplemental Material [file supp_29_11_1816__index.html]

Gene expression profiling of single cells from archival tissue with laser-capture microdissection and Smart-3SEQ — Supplemental Material 

# Gene expression profiling of single cells from archival tissue with laser-capture microdissection and Smart-3SEQ

## Supplemental Material

- Supplemental\_File\_2.pdf
- Supplemental\_File\_4.zip
- Supplemental\_File\_5.xlsx
- Supplemental\_Code.zip
- Supplemental\_File\_3.xlsx
- Supplemental\_File\_1.pdf
