# Supplementary figures and images for: Gene expression profiling of single cells from archival tissue with laser-capture microdissection and Smart-3SEQ (part 2 of 2)
Source: Genome Res. 2019 Nov;29(11):1816–25. doi: 10.1101/gr.234807.118 (PMC6836736; doi:10.1101/gr.234807.118)

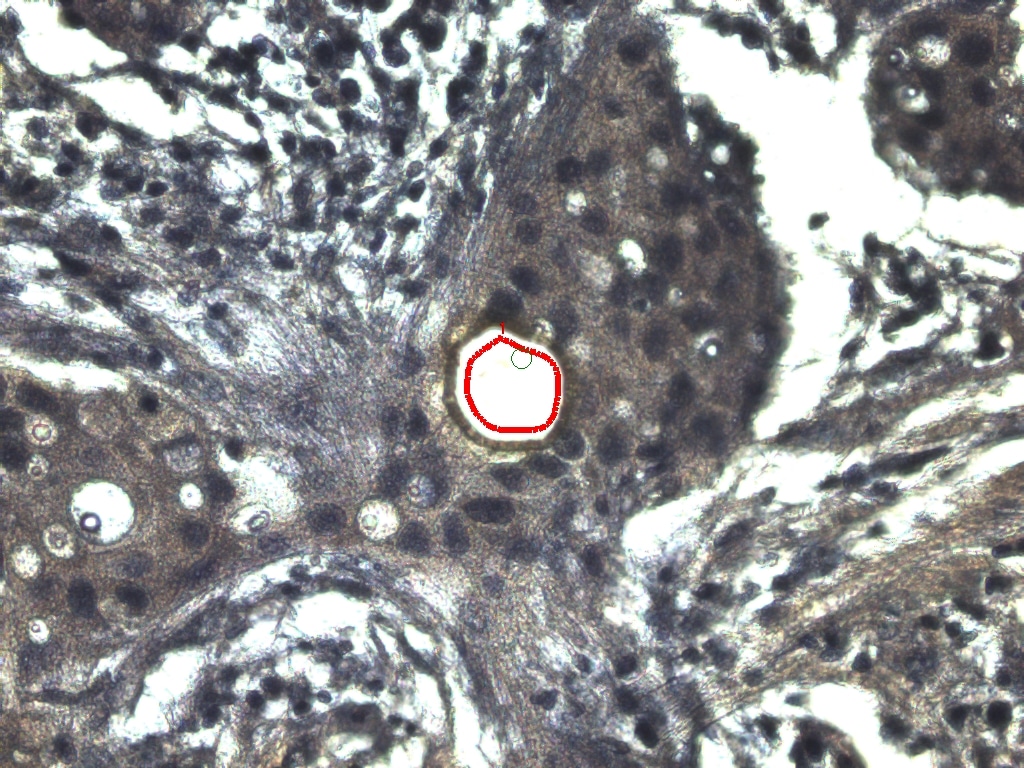

Supplement: Supplemental Material [file supp_gr.234807.118_Supplemental_File_4.zip › SINGLE CELL/DCIS single cell/DCIS-2 AFTER.jpeg]

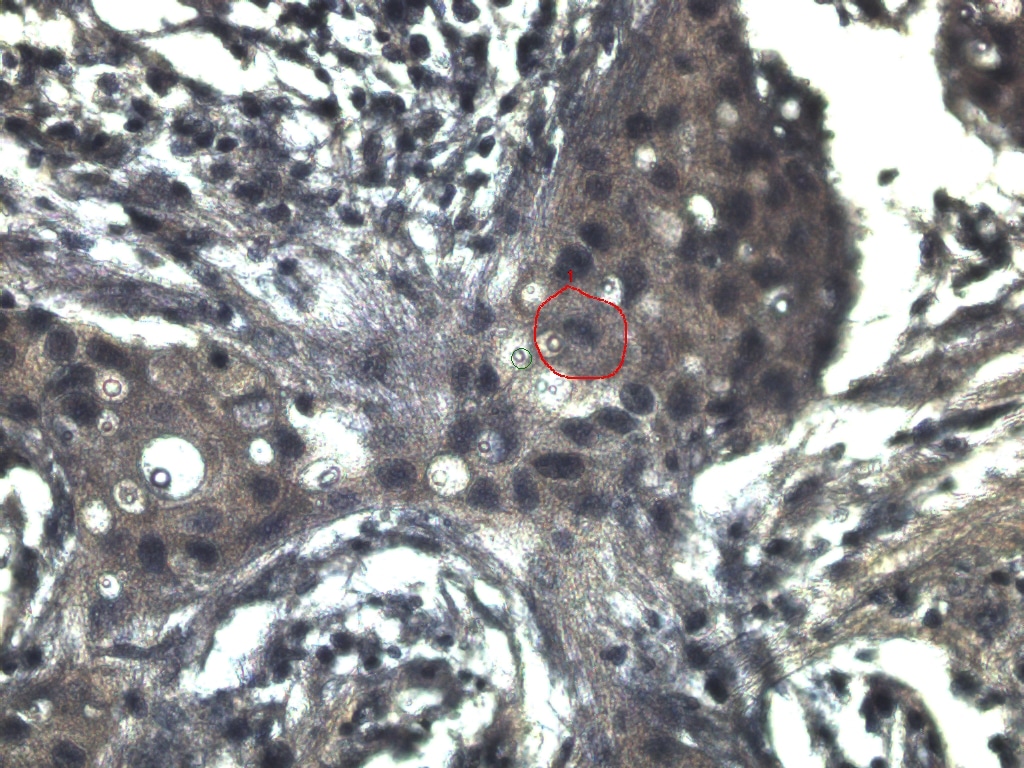

Supplement: Supplemental Material [file supp_gr.234807.118_Supplemental_File_4.zip › SINGLE CELL/DCIS single cell/DCIS-2 BEFORE.jpeg]

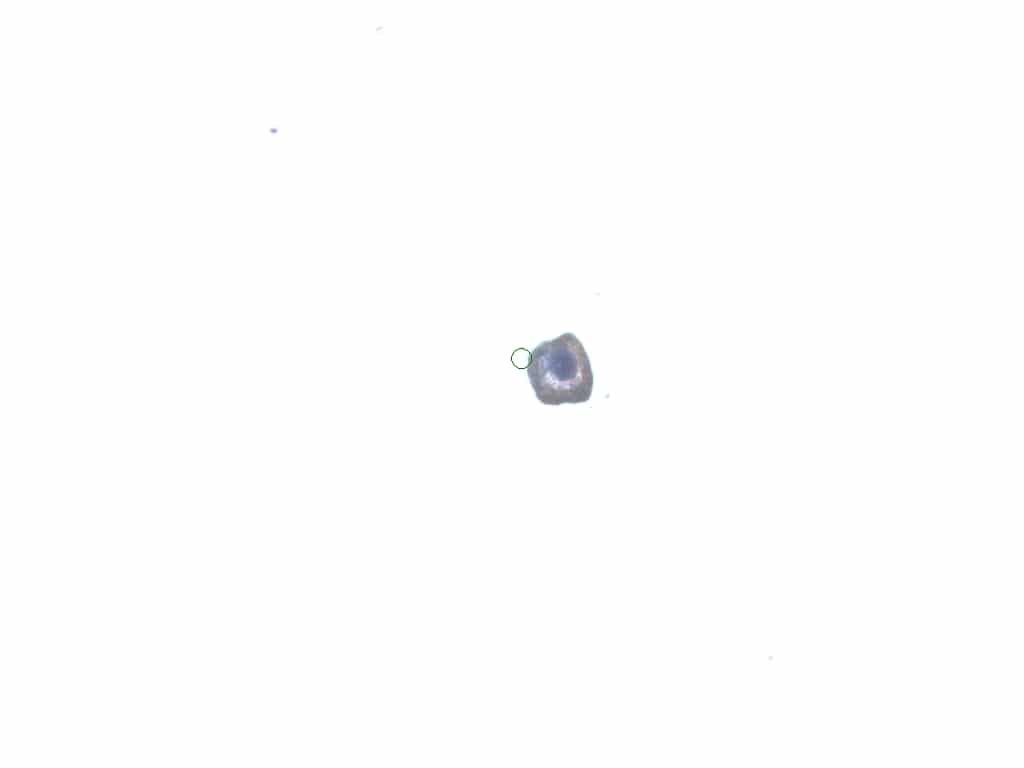

Supplement: Supplemental Material [file supp_gr.234807.118_Supplemental_File_4.zip › SINGLE CELL/DCIS single cell/DCIS-3 40x.jpeg]

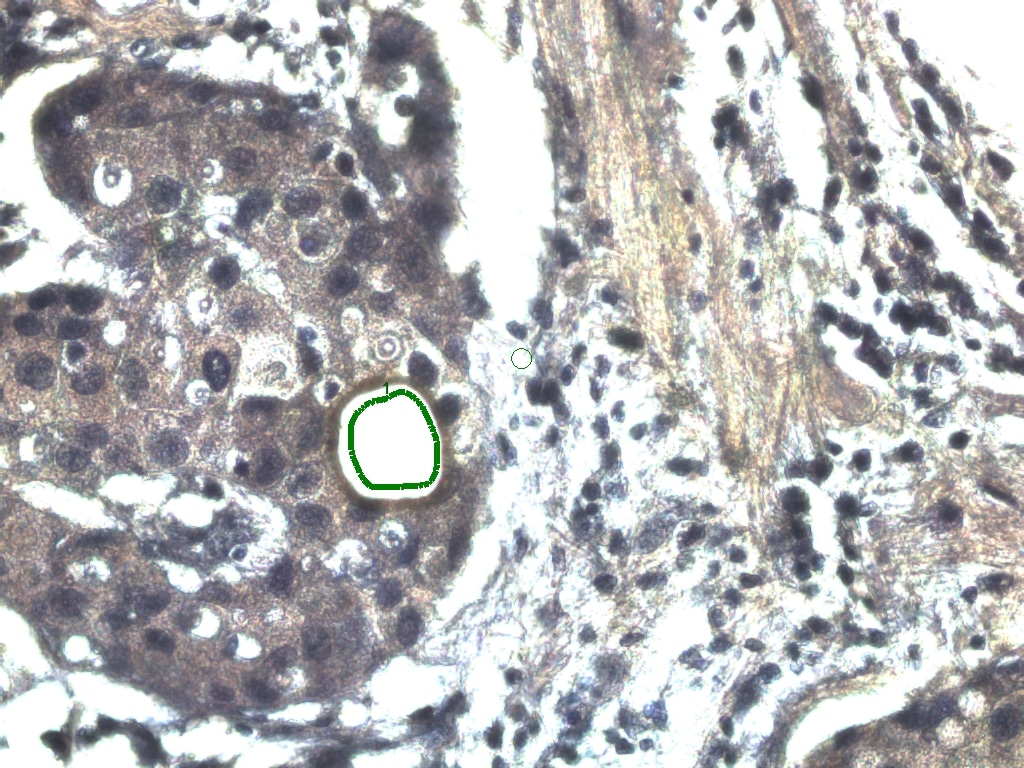

Supplement: Supplemental Material [file supp_gr.234807.118_Supplemental_File_4.zip › SINGLE CELL/DCIS single cell/DCIS-3 AFTER.jpeg]

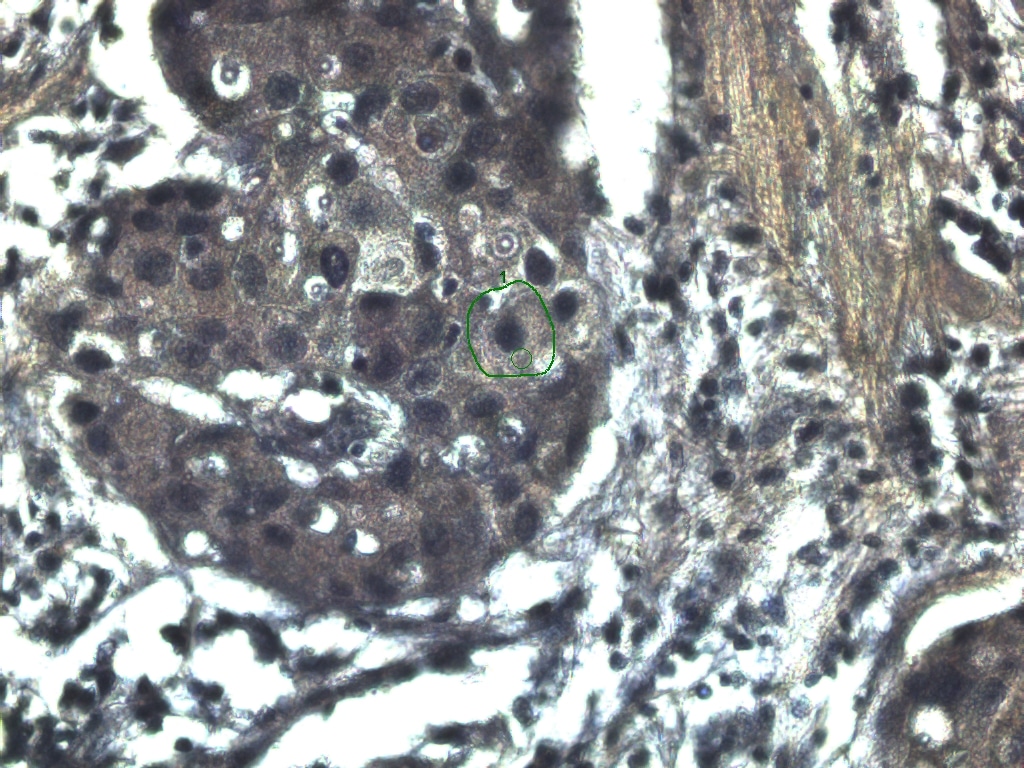

Supplement: Supplemental Material [file supp_gr.234807.118_Supplemental_File_4.zip › SINGLE CELL/DCIS single cell/DCIS-3 BEFORE.jpeg]

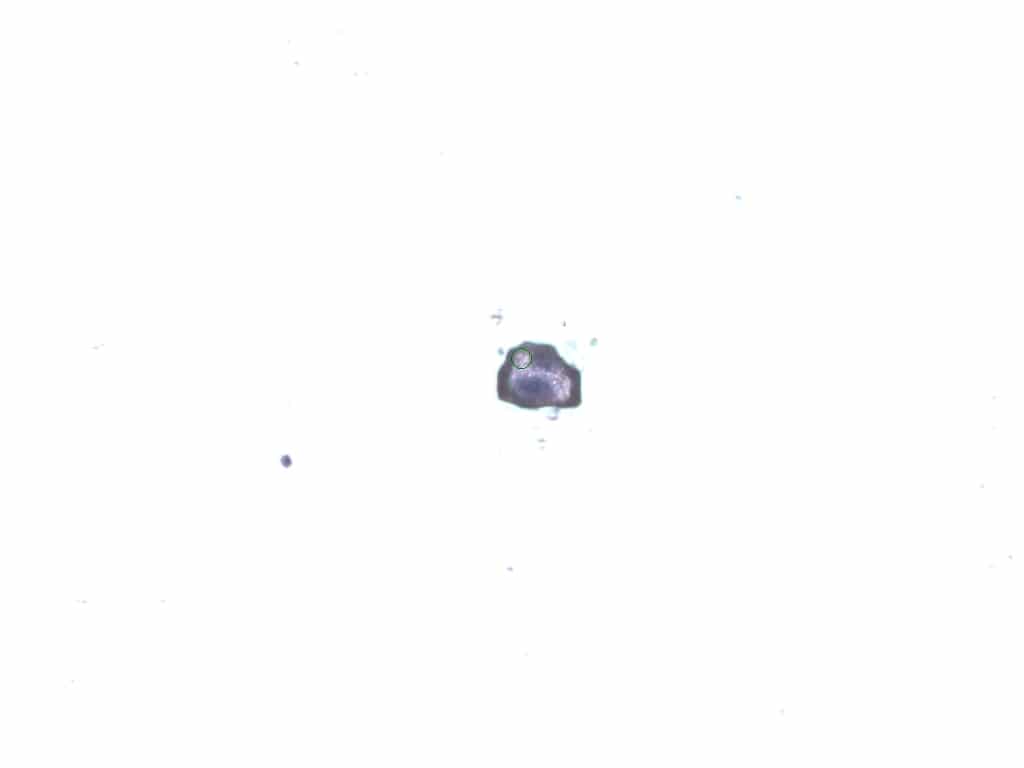

Supplement: Supplemental Material [file supp_gr.234807.118_Supplemental_File_4.zip › SINGLE CELL/DCIS single cell/DCIS-4 40x.jpeg]

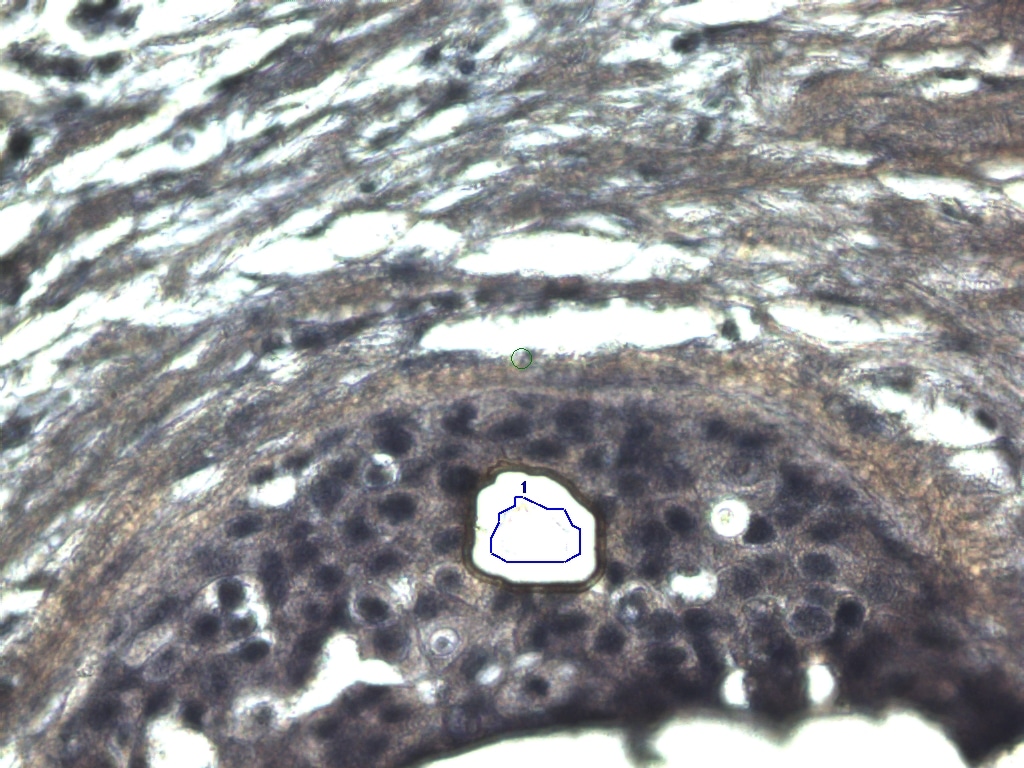

Supplement: Supplemental Material [file supp_gr.234807.118_Supplemental_File_4.zip › SINGLE CELL/DCIS single cell/DCIS-4 AFTER.jpeg]

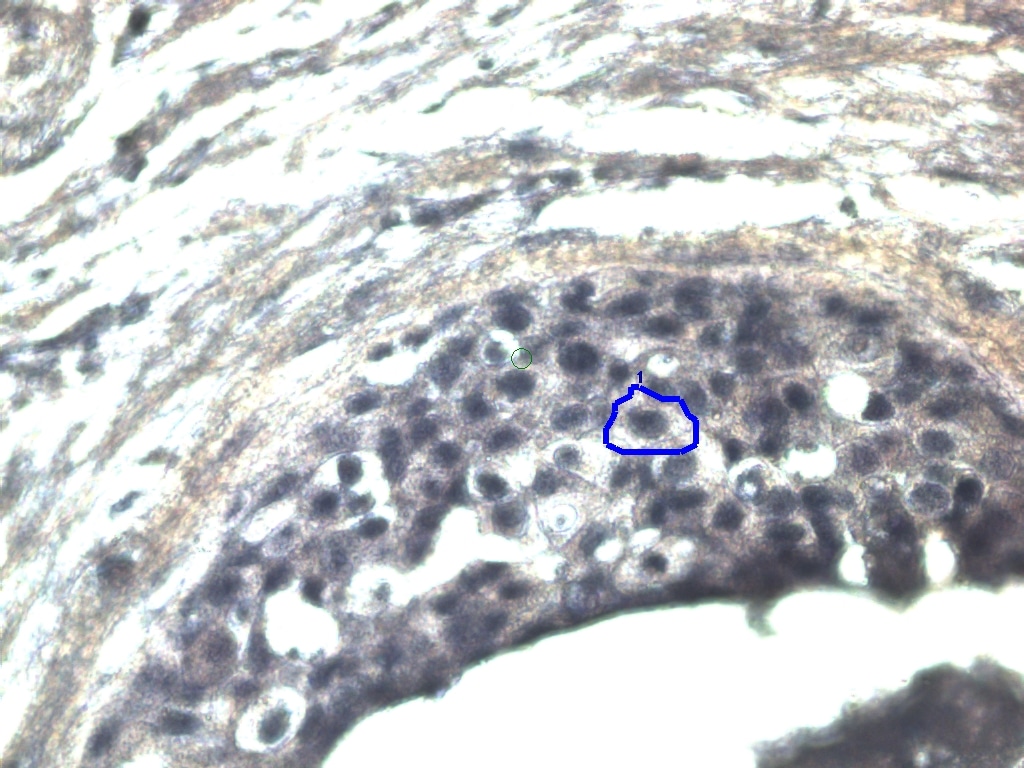

Supplement: Supplemental Material [file supp_gr.234807.118_Supplemental_File_4.zip › SINGLE CELL/DCIS single cell/DCIS-4 BEFORE.jpeg]

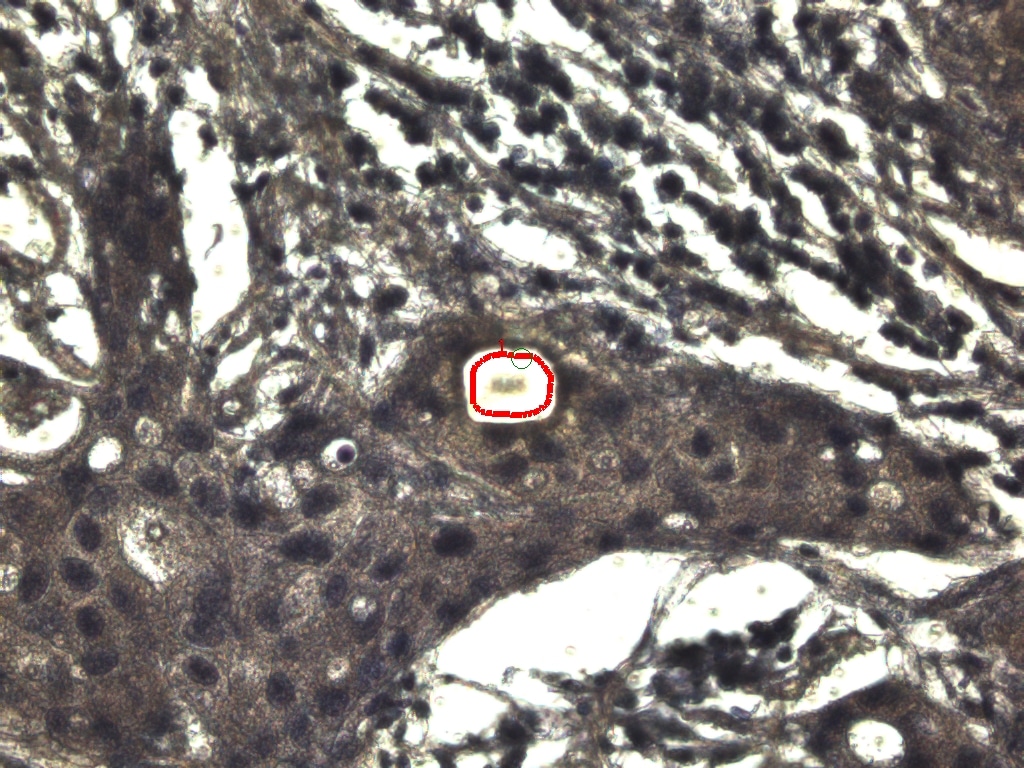

Supplement: Supplemental Material [file supp_gr.234807.118_Supplemental_File_4.zip › SINGLE CELL/DCIS single cell/DCIS-5 AFTER.jpeg]

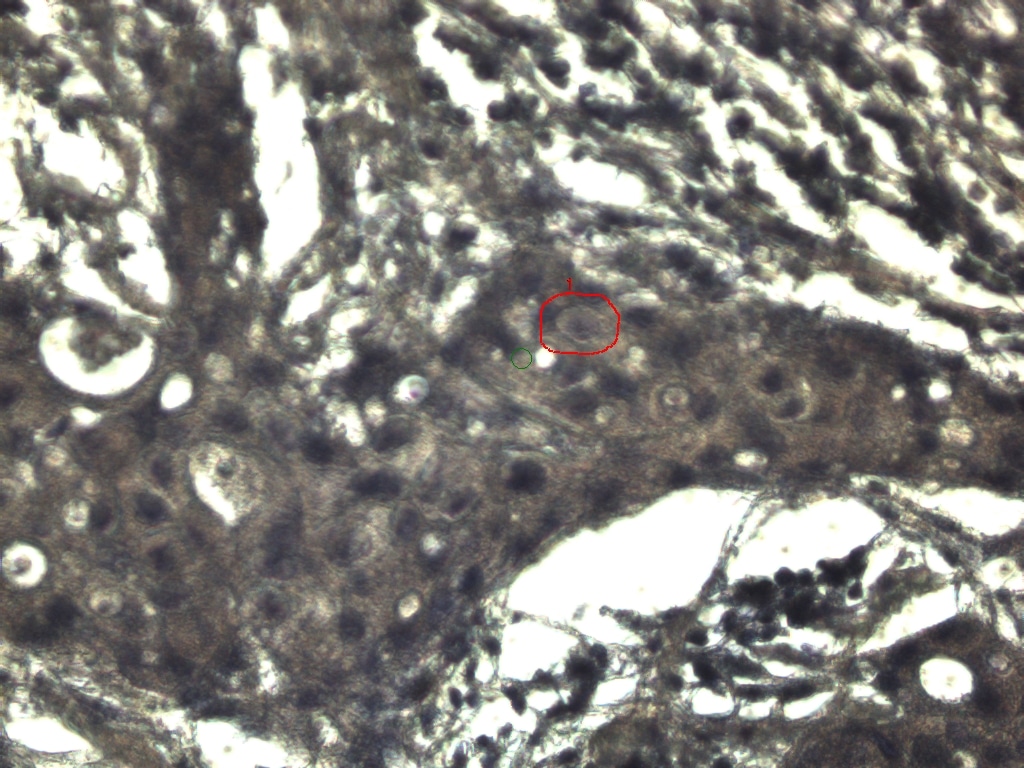

Supplement: Supplemental Material [file supp_gr.234807.118_Supplemental_File_4.zip › SINGLE CELL/DCIS single cell/DCIS-5 BEFORE.jpeg]

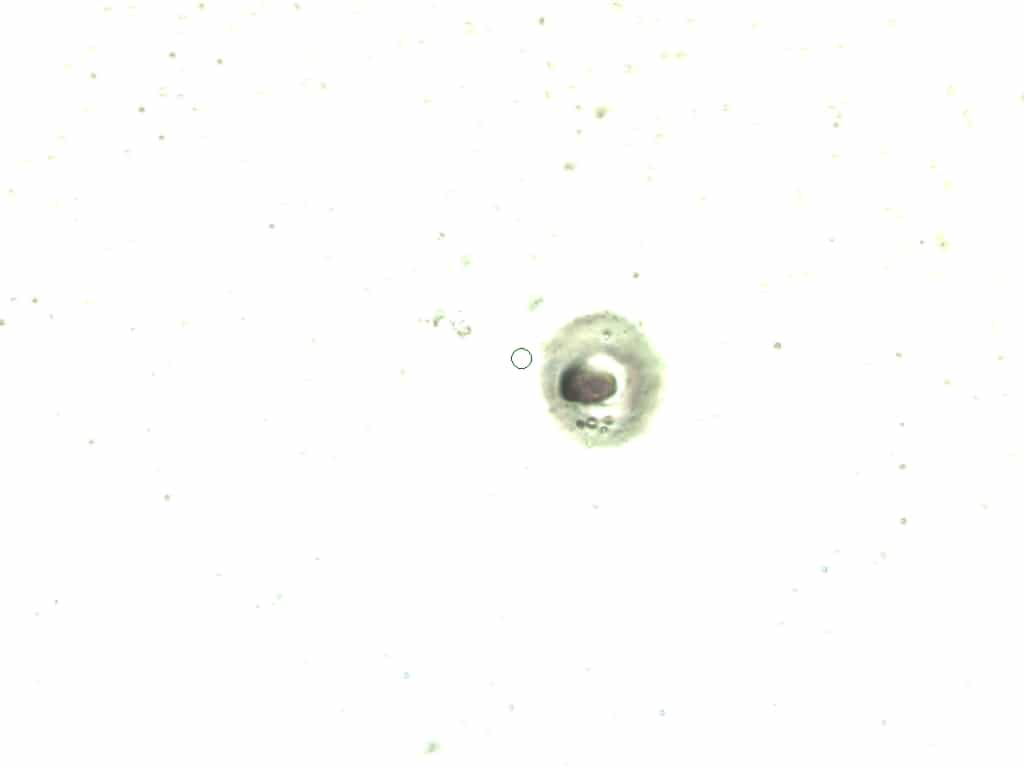

Supplement: Supplemental Material [file supp_gr.234807.118_Supplemental_File_4.zip › SINGLE CELL/DCIS single cell/DCIS-5.jpeg]

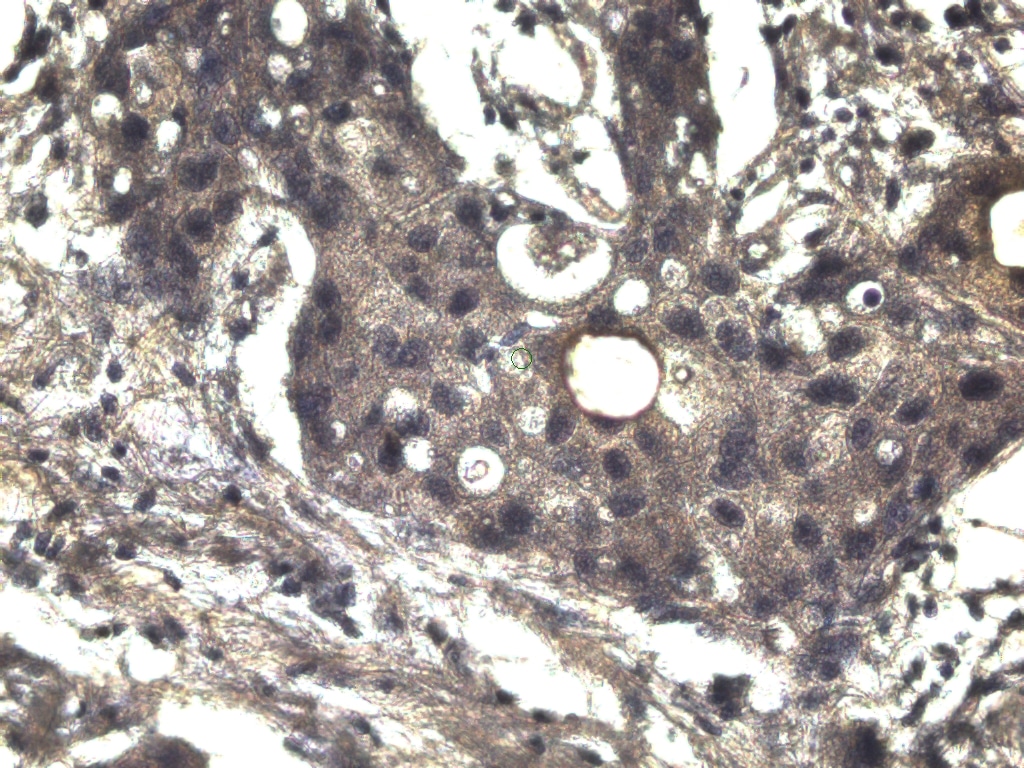

Supplement: Supplemental Material [file supp_gr.234807.118_Supplemental_File_4.zip › SINGLE CELL/DCIS single cell/DCIS-6 AFTER.jpeg]

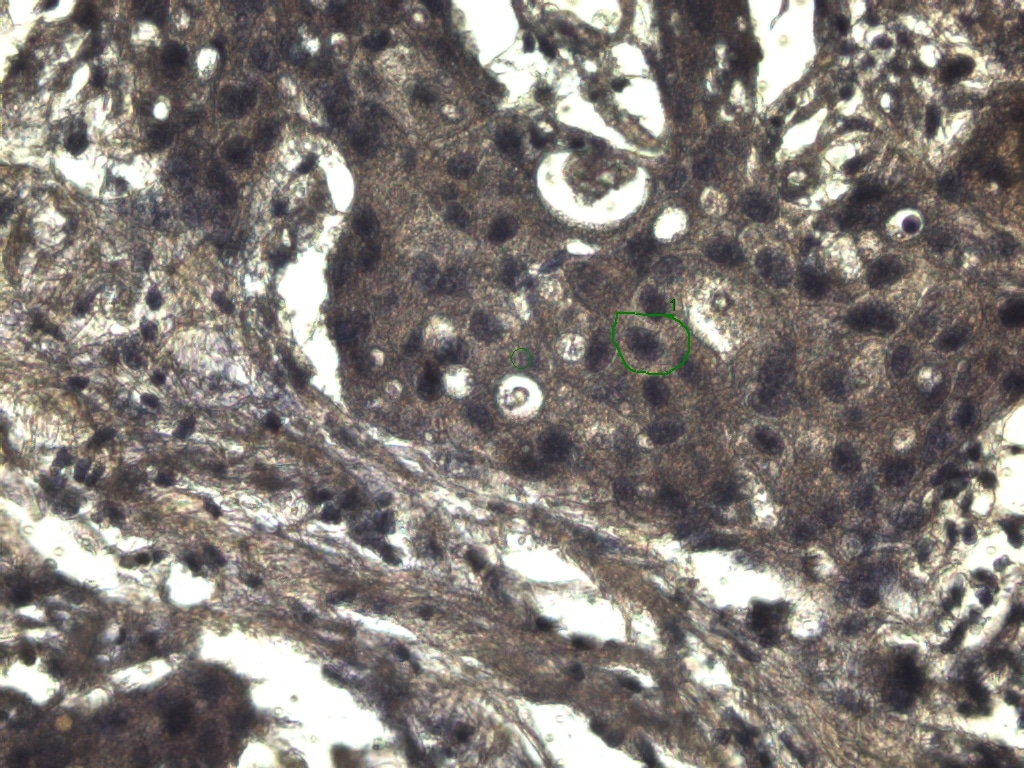

Supplement: Supplemental Material [file supp_gr.234807.118_Supplemental_File_4.zip › SINGLE CELL/DCIS single cell/DCIS-6 BEFORE.jpeg]

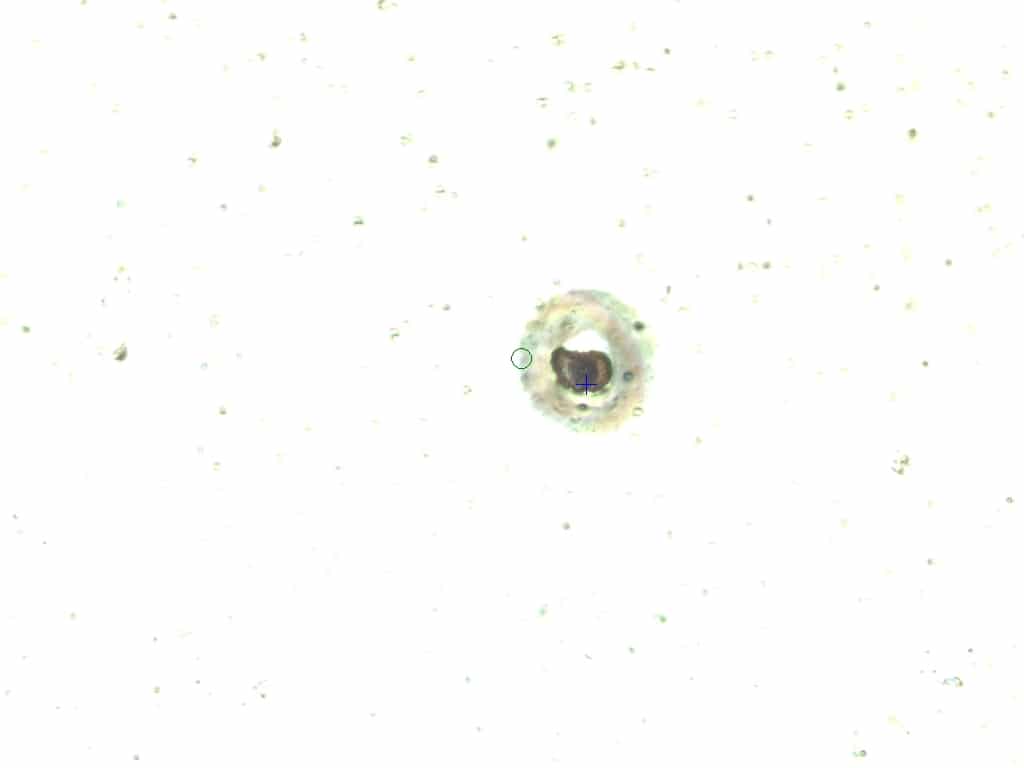

Supplement: Supplemental Material [file supp_gr.234807.118_Supplemental_File_4.zip › SINGLE CELL/DCIS single cell/DCIS-6.jpeg]

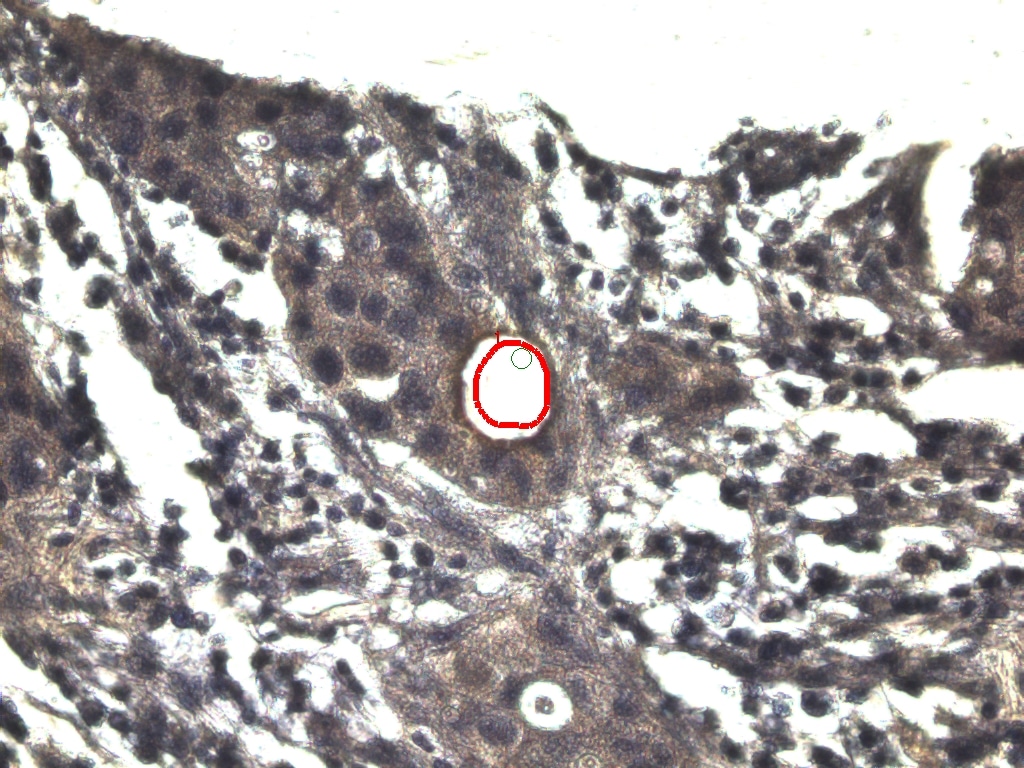

Supplement: Supplemental Material [file supp_gr.234807.118_Supplemental_File_4.zip › SINGLE CELL/DCIS single cell/DCIS-7 AFTER.jpeg]

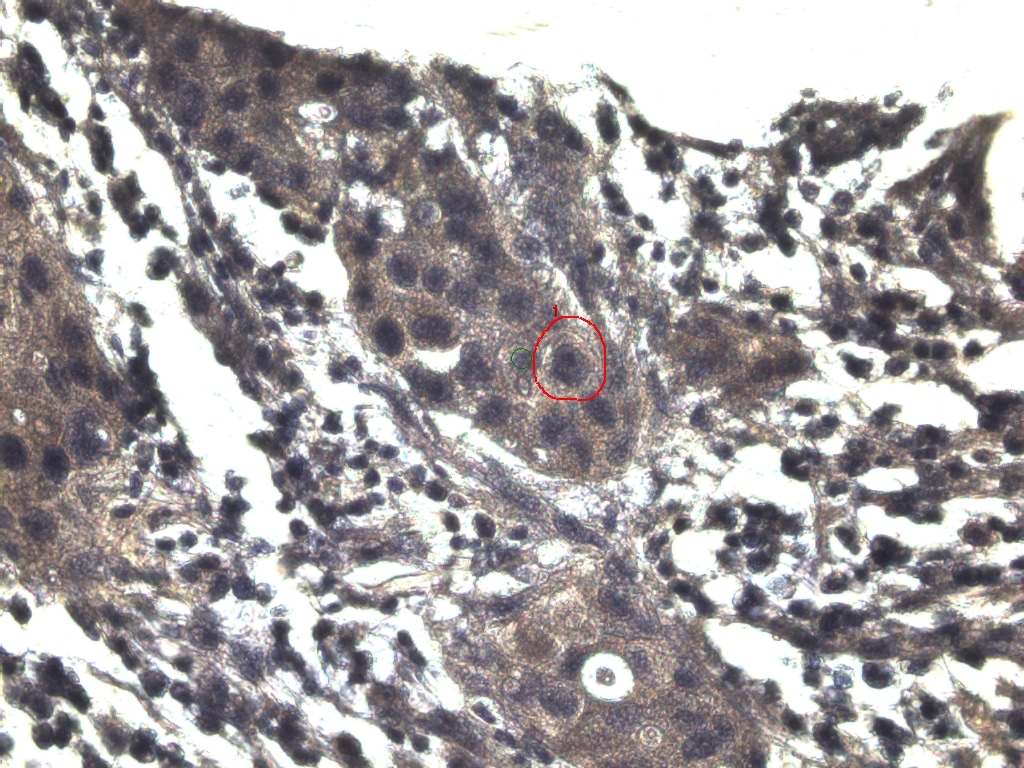

Supplement: Supplemental Material [file supp_gr.234807.118_Supplemental_File_4.zip › SINGLE CELL/DCIS single cell/DCIS-7 BEFORE.jpeg]

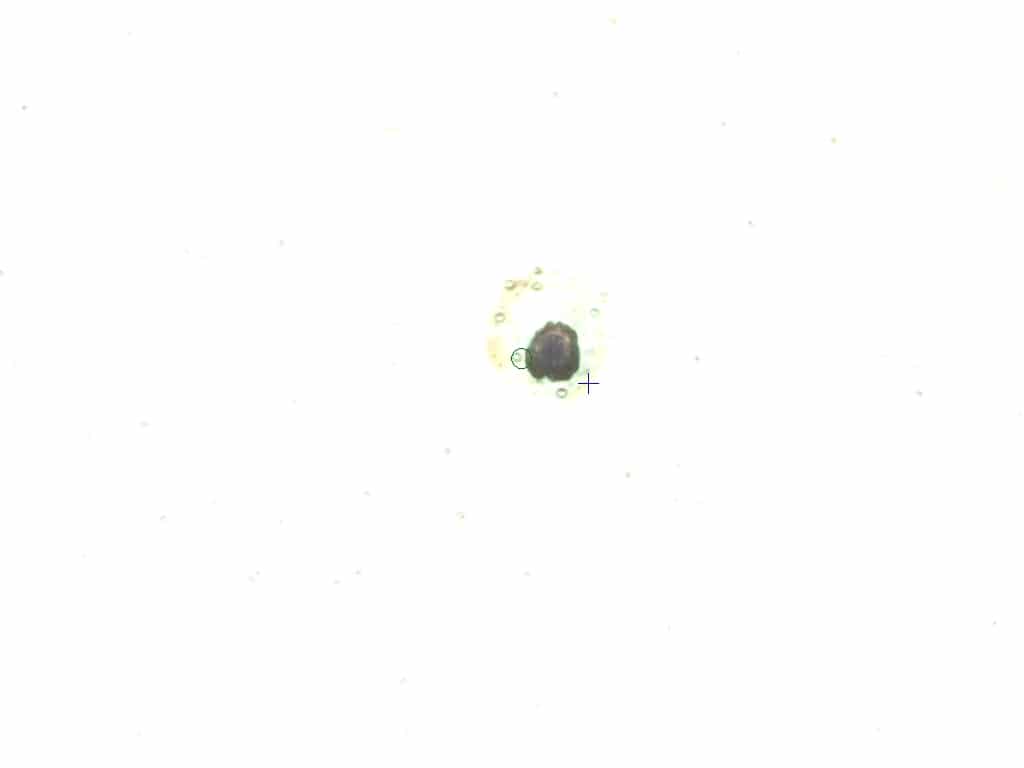

Supplement: Supplemental Material [file supp_gr.234807.118_Supplemental_File_4.zip › SINGLE CELL/DCIS single cell/DCIS-7.jpeg]

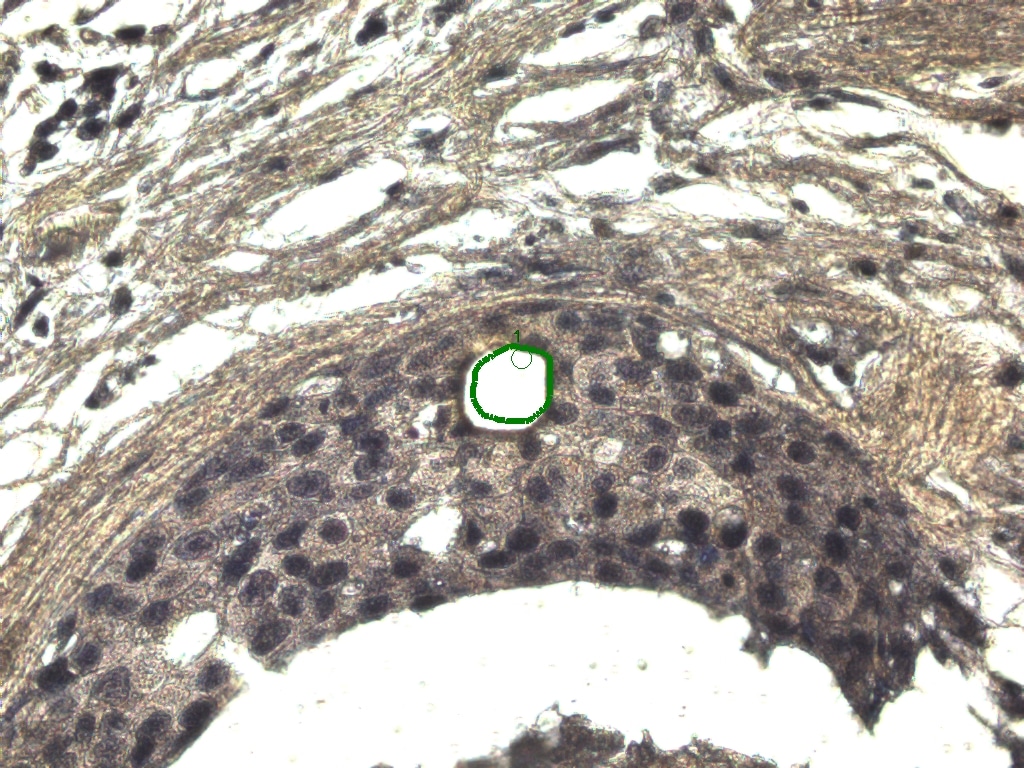

Supplement: Supplemental Material [file supp_gr.234807.118_Supplemental_File_4.zip › SINGLE CELL/DCIS single cell/DCIS-8 AFTER.jpeg]

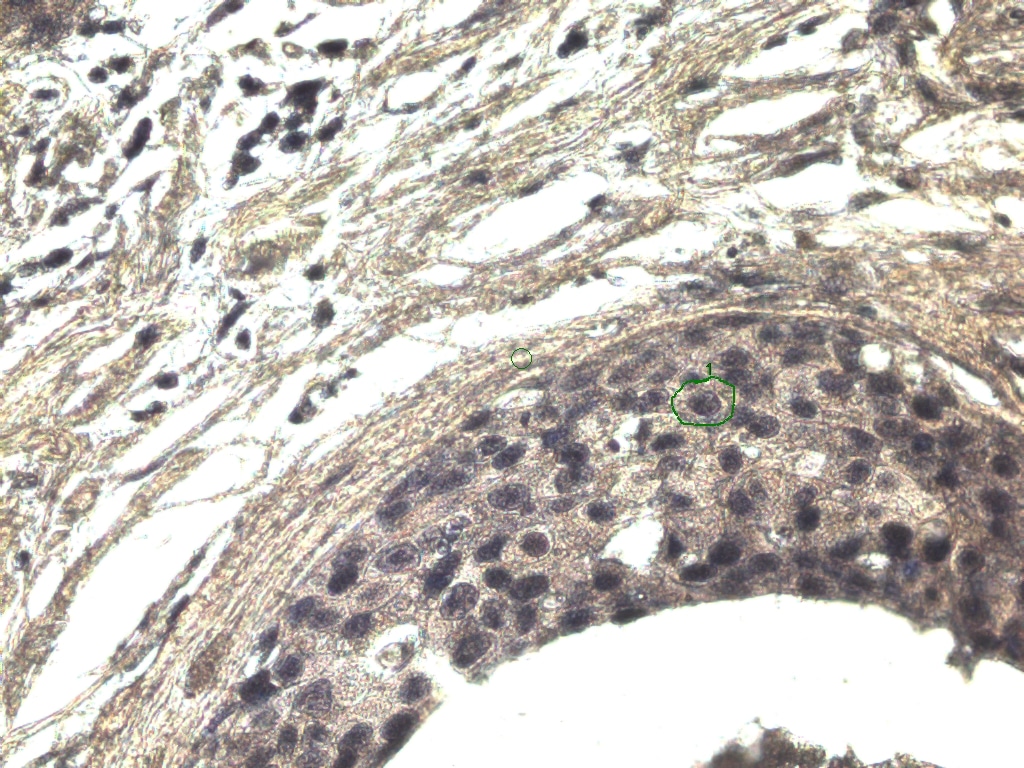

Supplement: Supplemental Material [file supp_gr.234807.118_Supplemental_File_4.zip › SINGLE CELL/DCIS single cell/DCIS-8 BEFORE.jpeg]

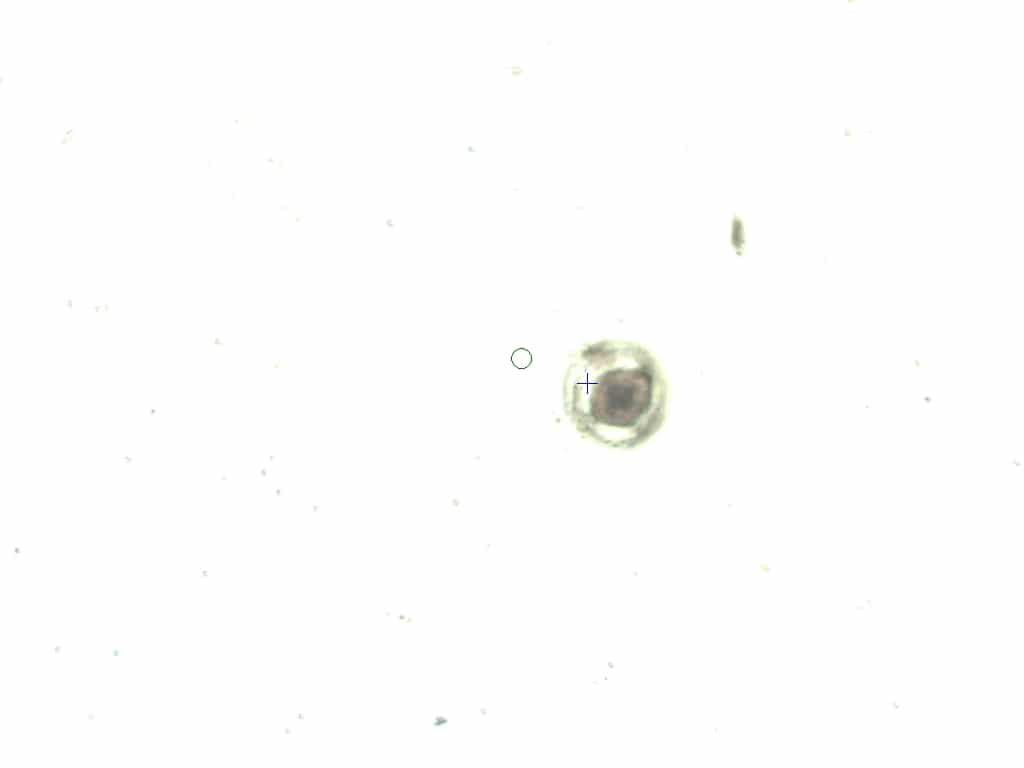

Supplement: Supplemental Material [file supp_gr.234807.118_Supplemental_File_4.zip › SINGLE CELL/DCIS single cell/DCIS-8.jpeg]

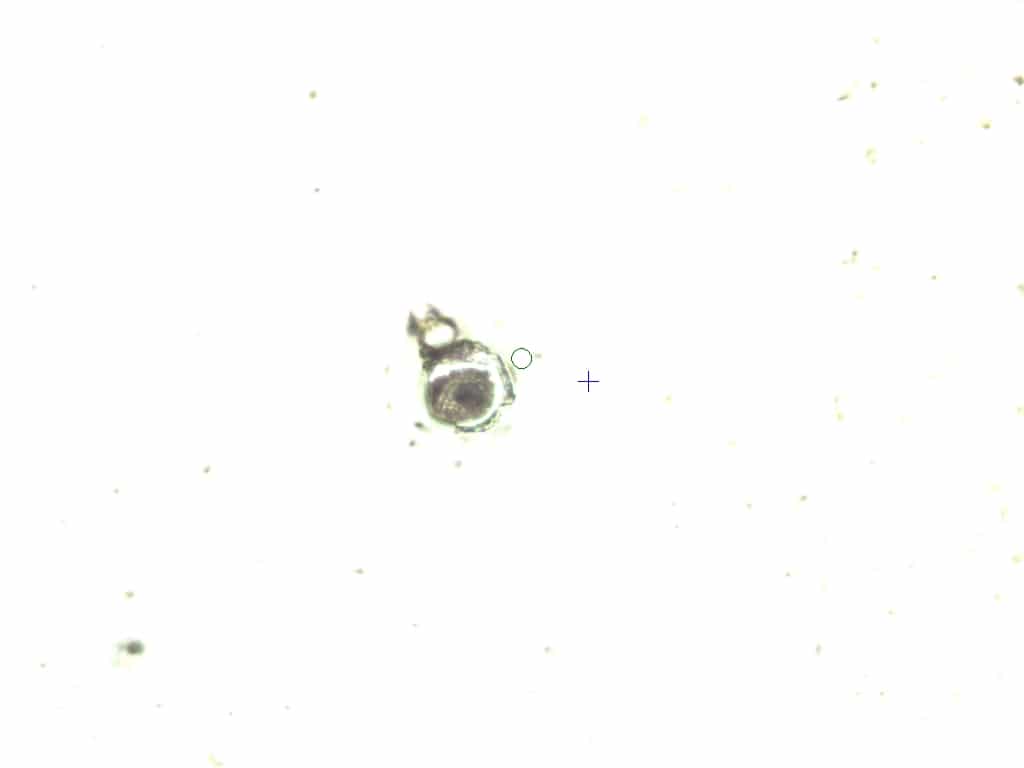

Supplement: Supplemental Material [file supp_gr.234807.118_Supplemental_File_4.zip › SINGLE CELL/DCIS single cell/DCIS-9 AFTER ABLATION.jpeg]

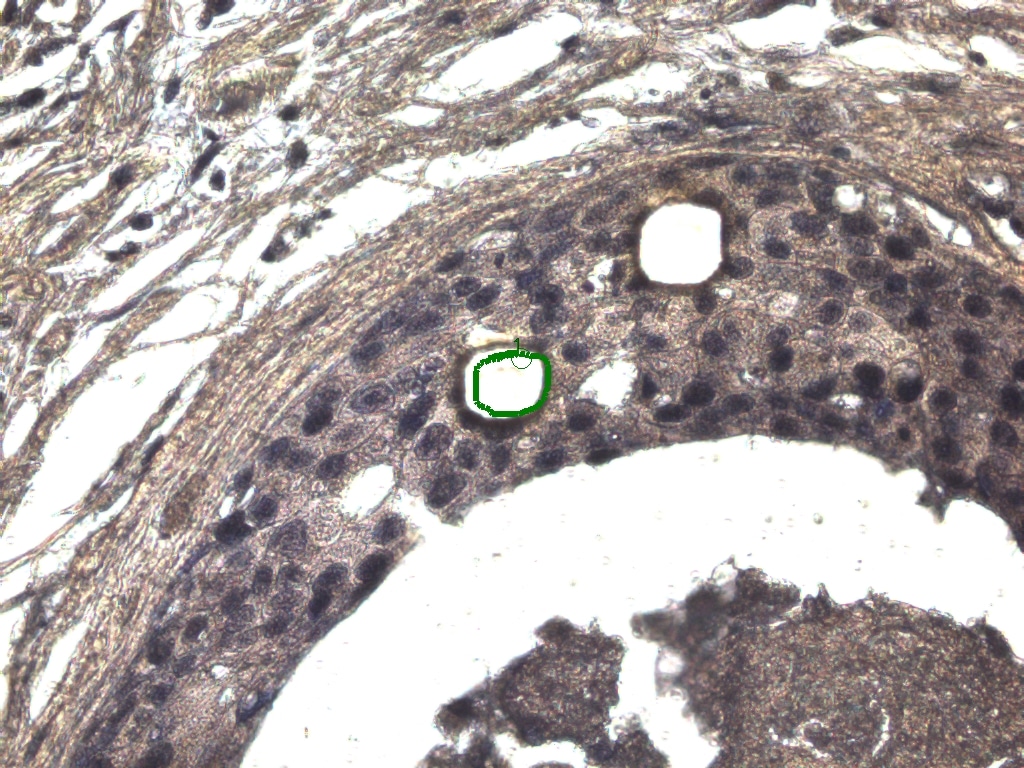

Supplement: Supplemental Material [file supp_gr.234807.118_Supplemental_File_4.zip › SINGLE CELL/DCIS single cell/DCIS-9 AFTER.jpeg]

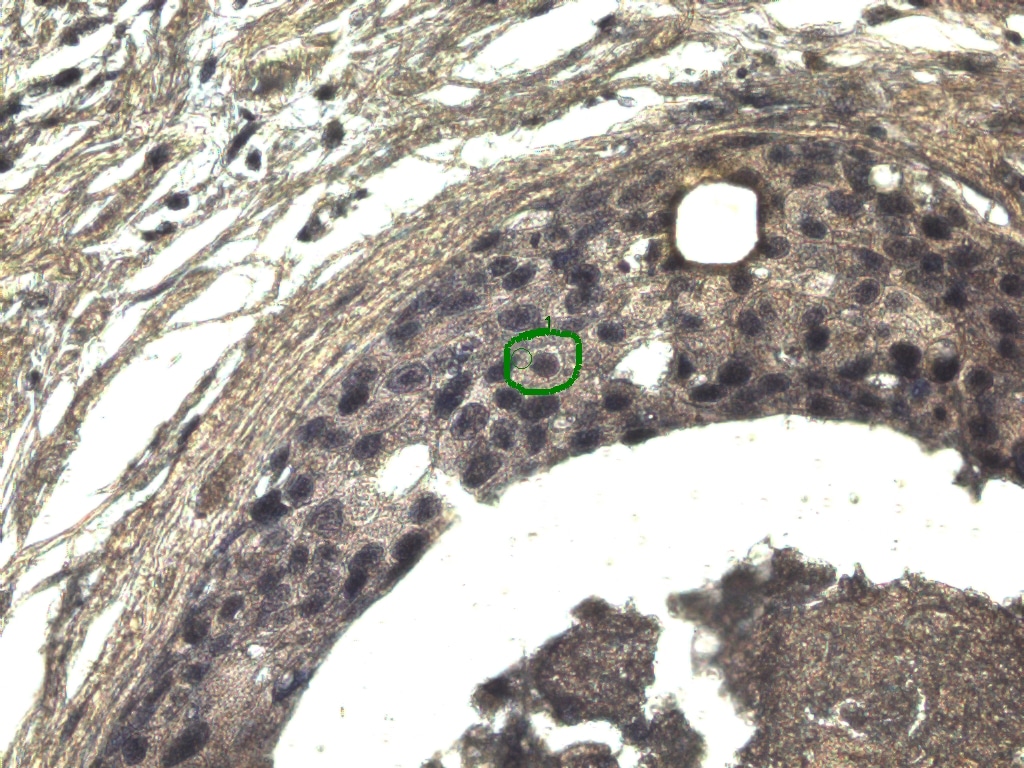

Supplement: Supplemental Material [file supp_gr.234807.118_Supplemental_File_4.zip › SINGLE CELL/DCIS single cell/DCIS-9 BEFORE.jpeg]

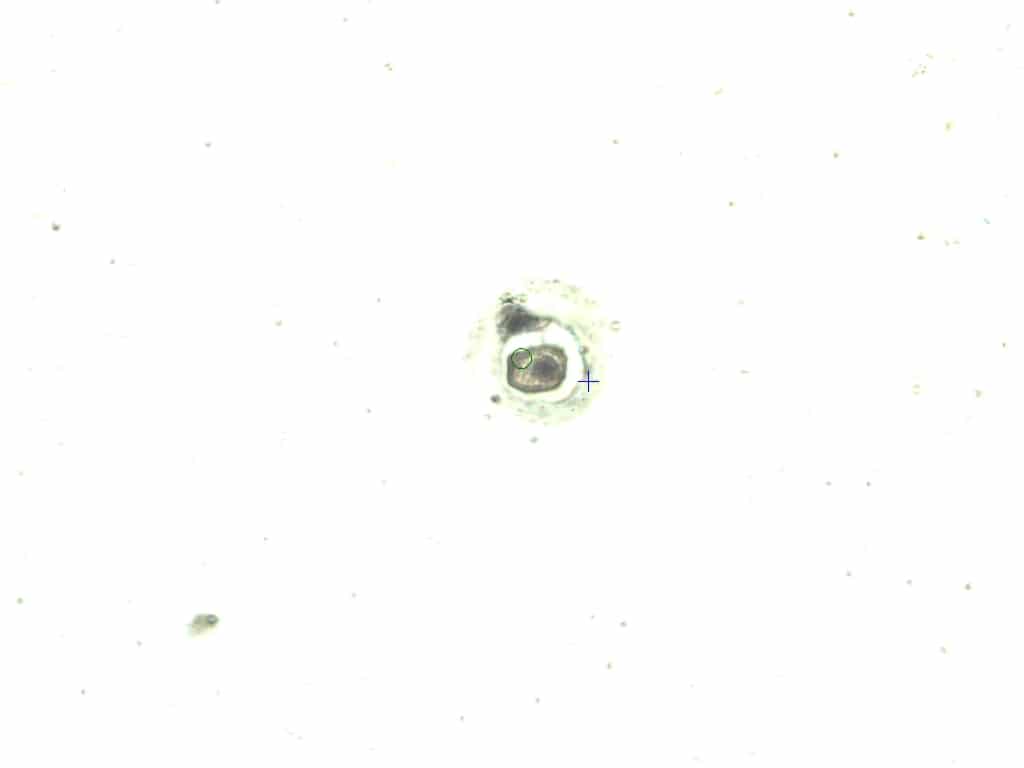

Supplement: Supplemental Material [file supp_gr.234807.118_Supplemental_File_4.zip › SINGLE CELL/DCIS single cell/DCIS-9.jpeg]

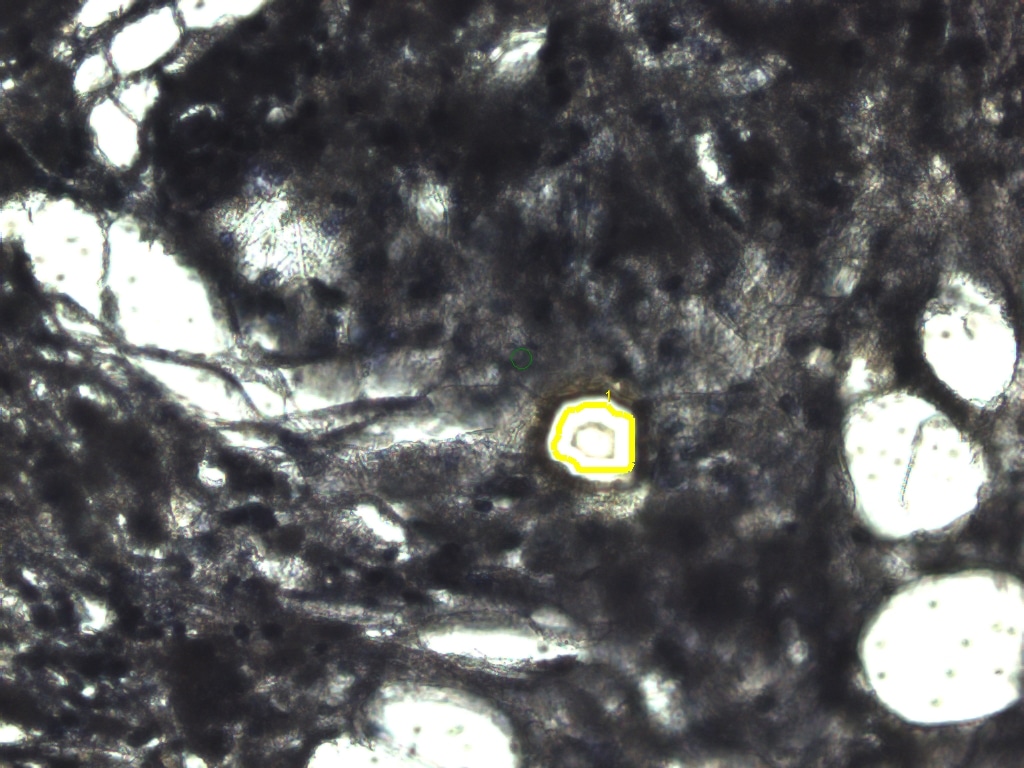

Supplement: Supplemental Material [file supp_gr.234807.118_Supplemental_File_4.zip › SINGLE CELL/Macrophage single cell/MAC1 AFTER.jpeg]

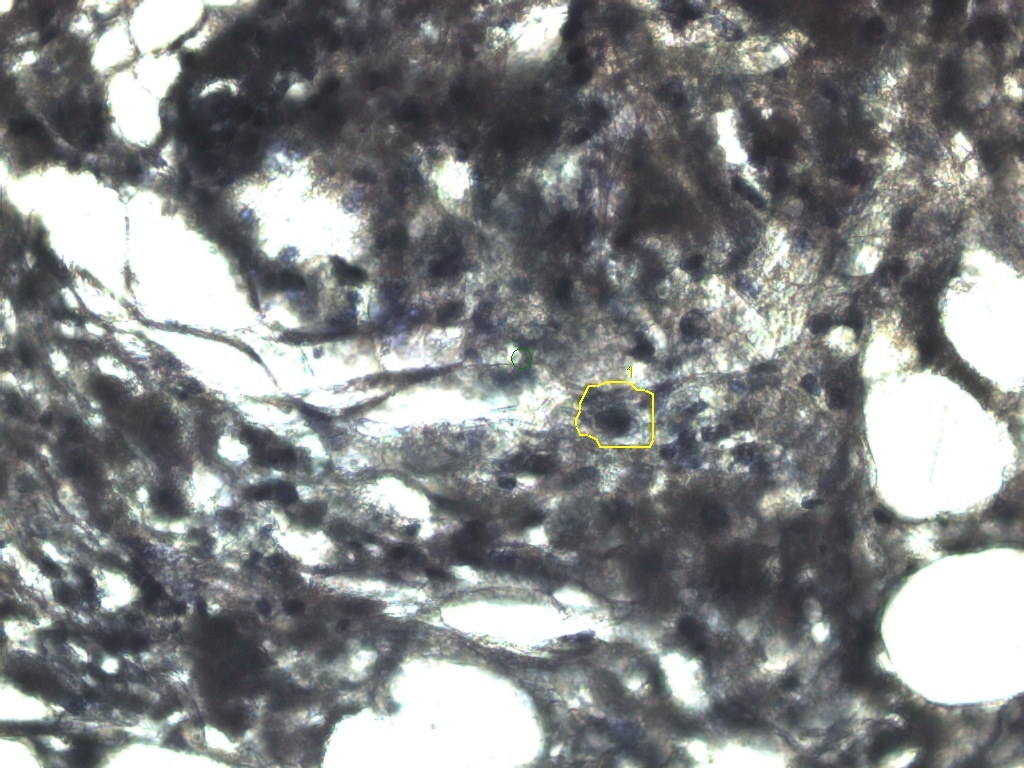

Supplement: Supplemental Material [file supp_gr.234807.118_Supplemental_File_4.zip › SINGLE CELL/Macrophage single cell/MAC1 BEFORE.jpeg]

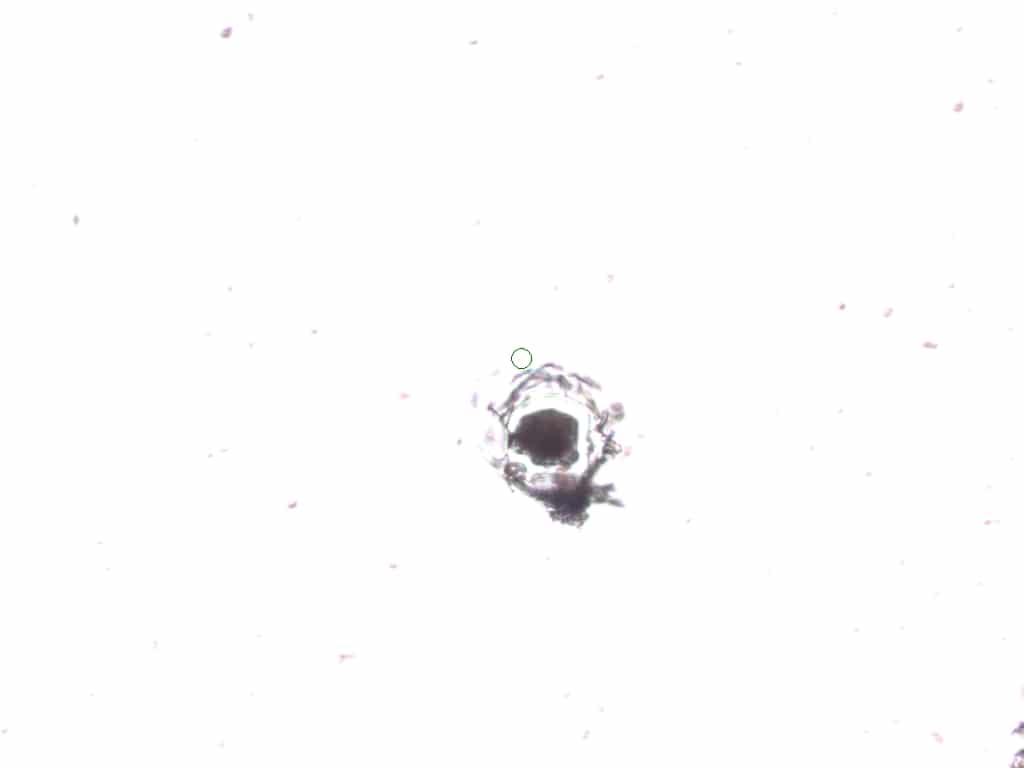

Supplement: Supplemental Material [file supp_gr.234807.118_Supplemental_File_4.zip › SINGLE CELL/Macrophage single cell/MAC1.jpeg]

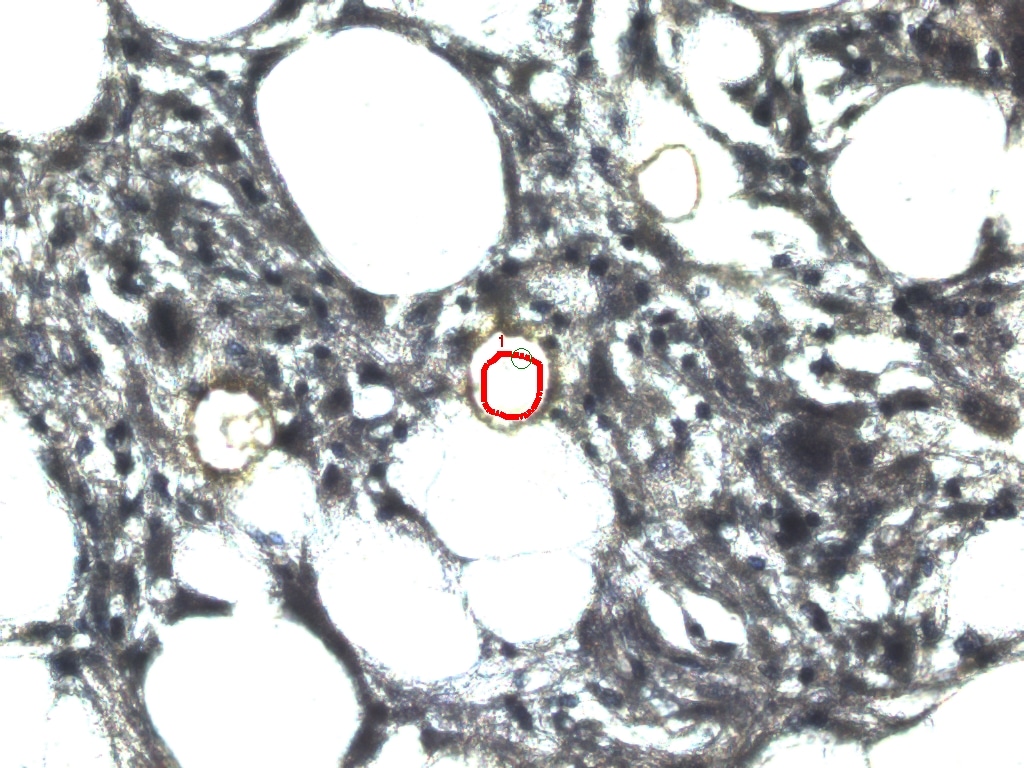

Supplement: Supplemental Material [file supp_gr.234807.118_Supplemental_File_4.zip › SINGLE CELL/Macrophage single cell/MAC10 AFTER.jpeg]

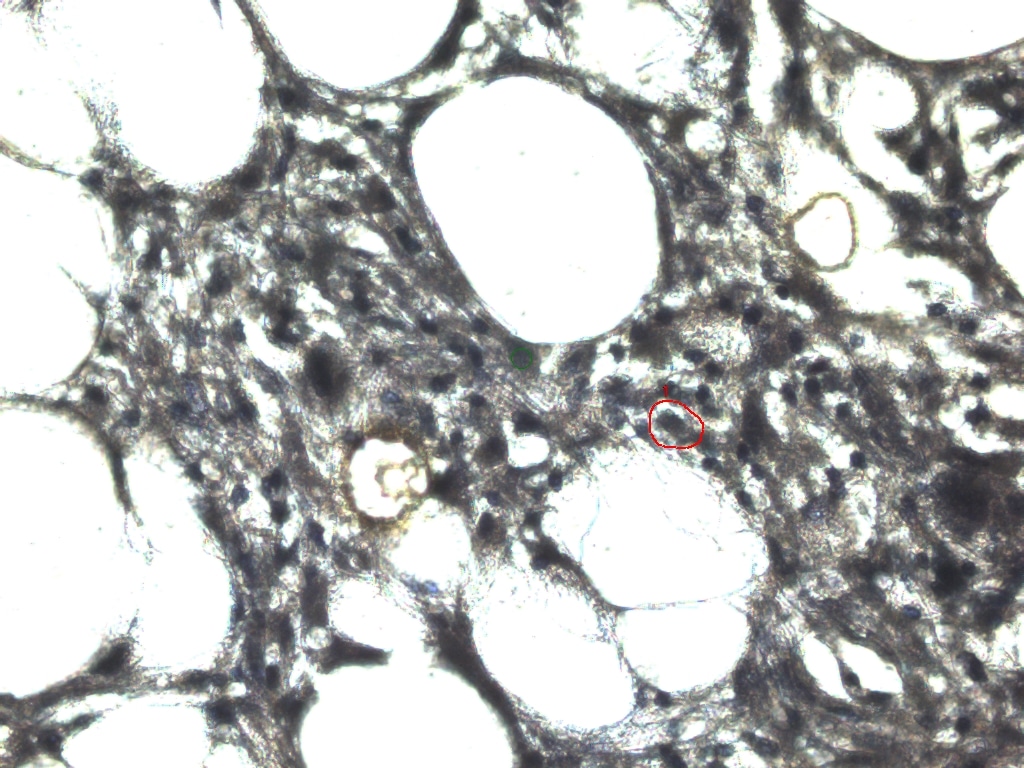

Supplement: Supplemental Material [file supp_gr.234807.118_Supplemental_File_4.zip › SINGLE CELL/Macrophage single cell/MAC10 BEFORE.jpeg]

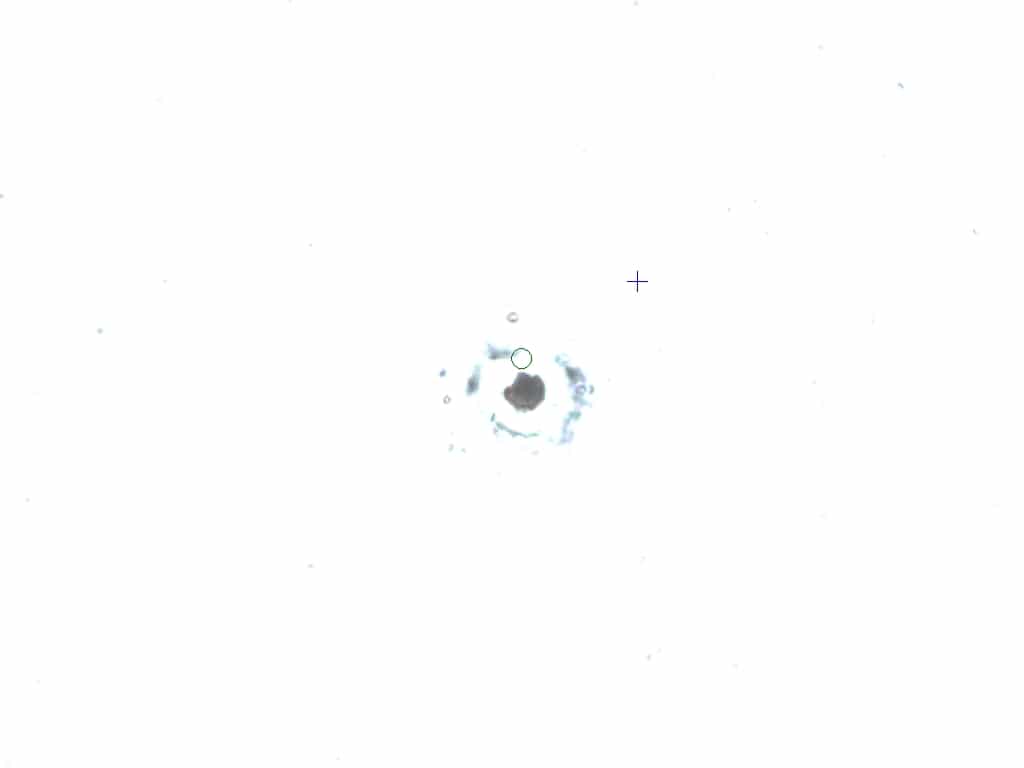

Supplement: Supplemental Material [file supp_gr.234807.118_Supplemental_File_4.zip › SINGLE CELL/Macrophage single cell/MAC10.jpeg]

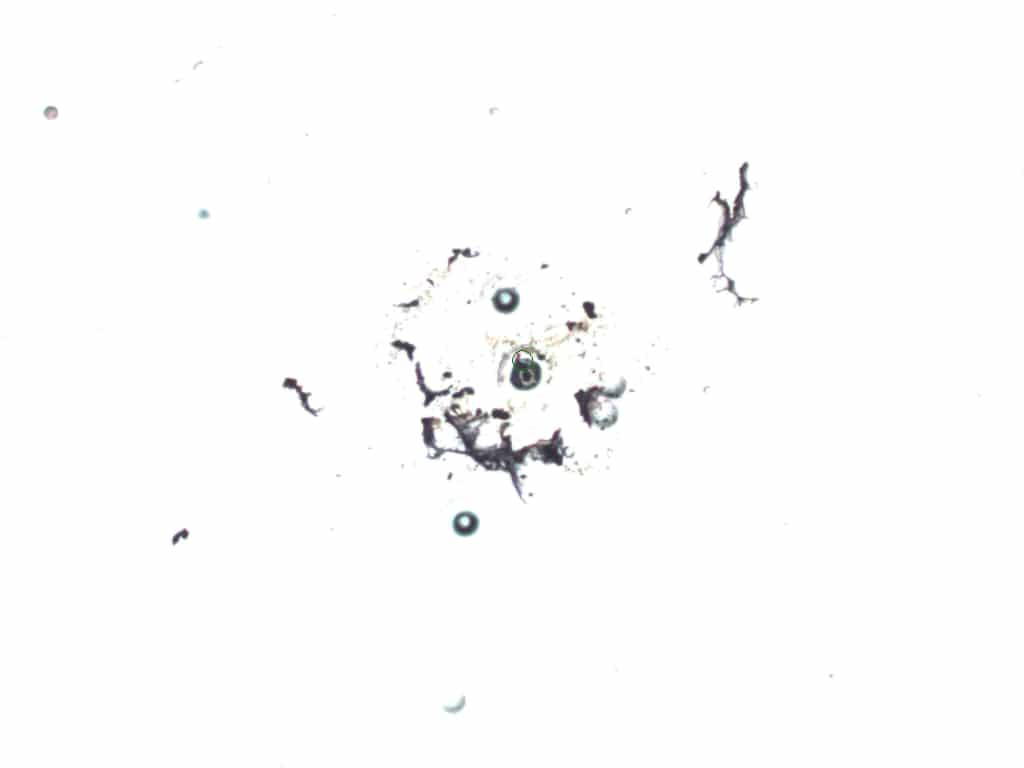

Supplement: Supplemental Material [file supp_gr.234807.118_Supplemental_File_4.zip › SINGLE CELL/Macrophage single cell/MAC2 AFTER ABLATION.jpeg]

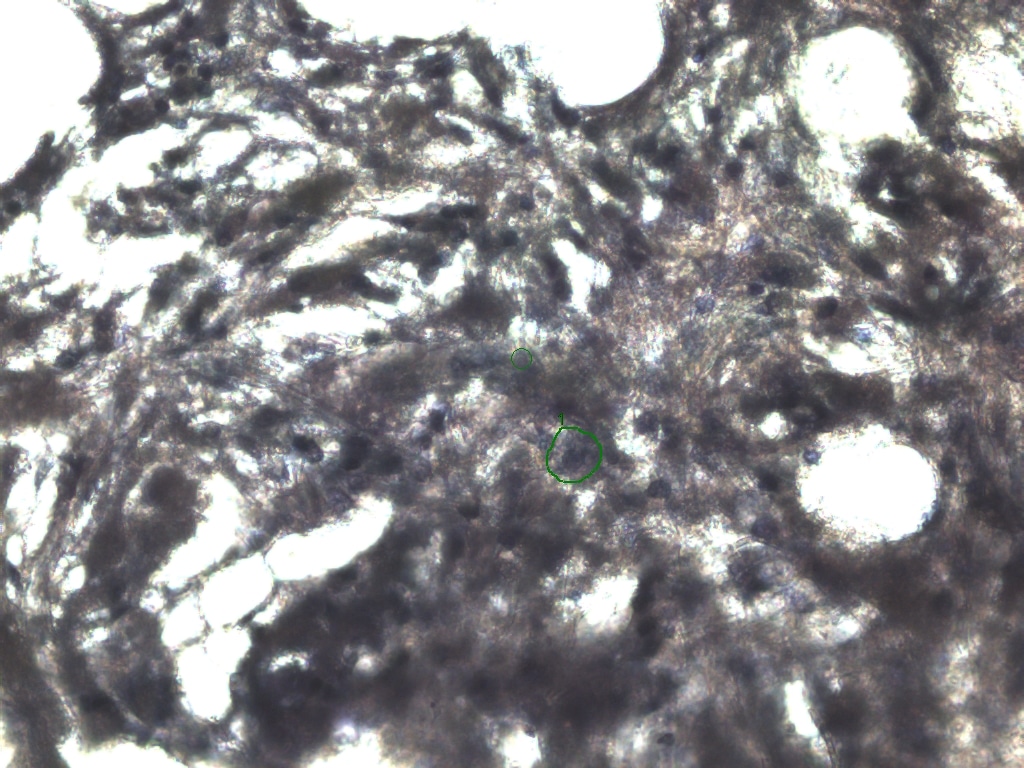

Supplement: Supplemental Material [file supp_gr.234807.118_Supplemental_File_4.zip › SINGLE CELL/Macrophage single cell/MAC2 BEFORE.jpeg]

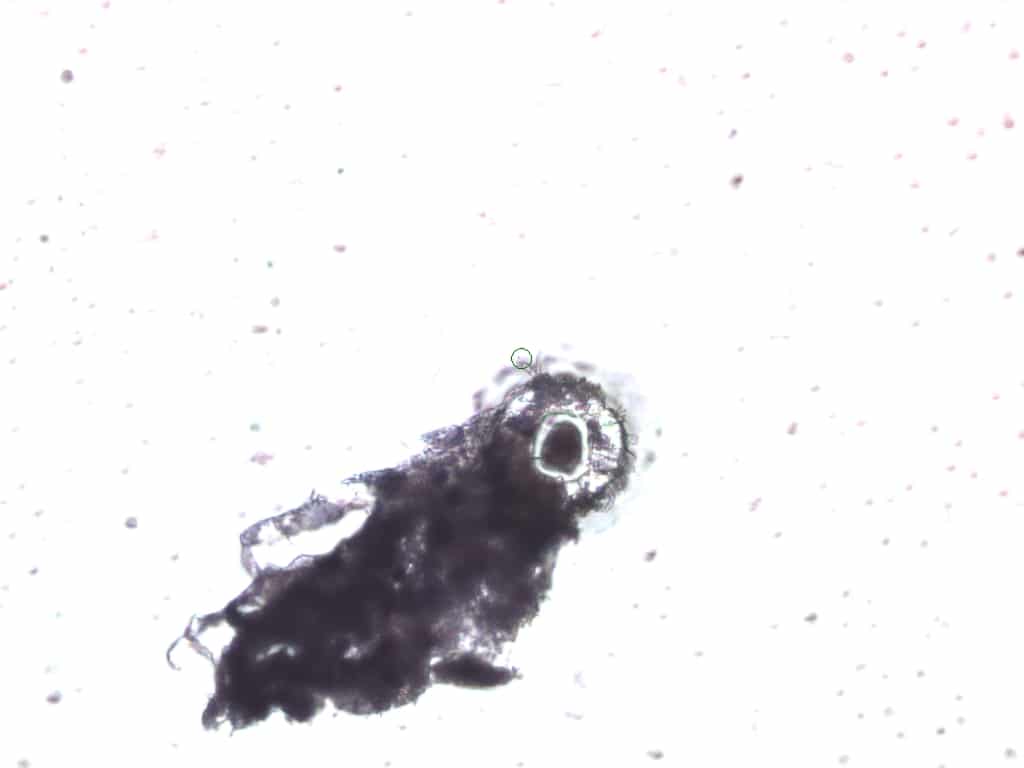

Supplement: Supplemental Material [file supp_gr.234807.118_Supplemental_File_4.zip › SINGLE CELL/Macrophage single cell/MAC2.jpeg]

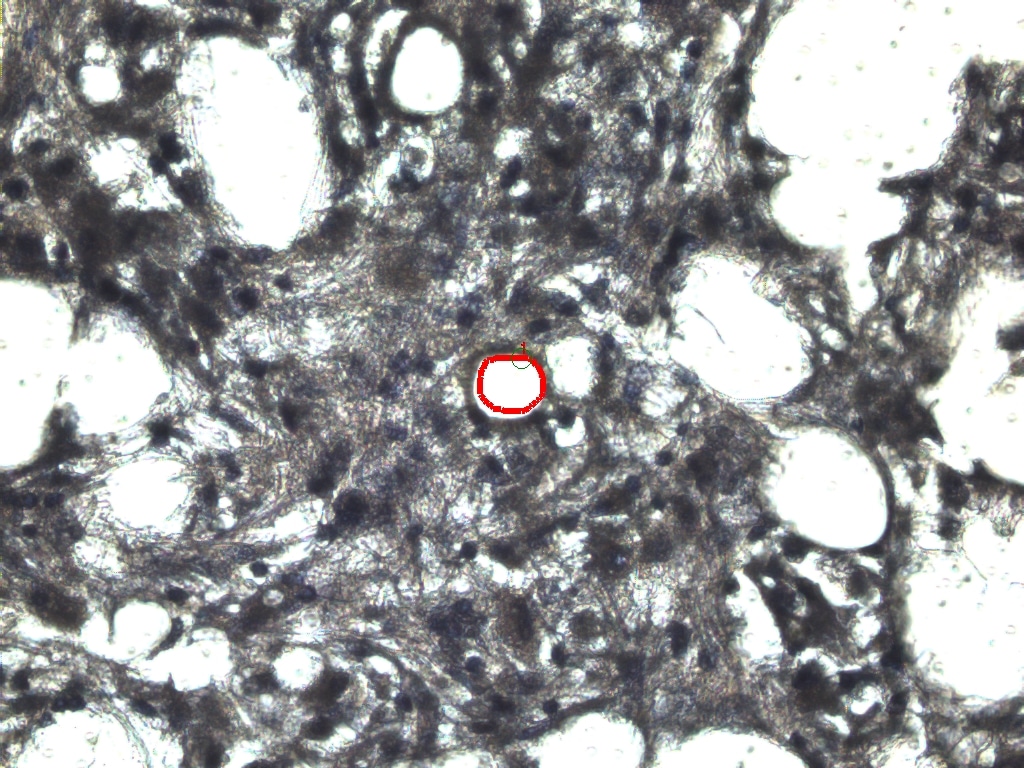

Supplement: Supplemental Material [file supp_gr.234807.118_Supplemental_File_4.zip › SINGLE CELL/Macrophage single cell/MAC3 AFTER.jpeg]

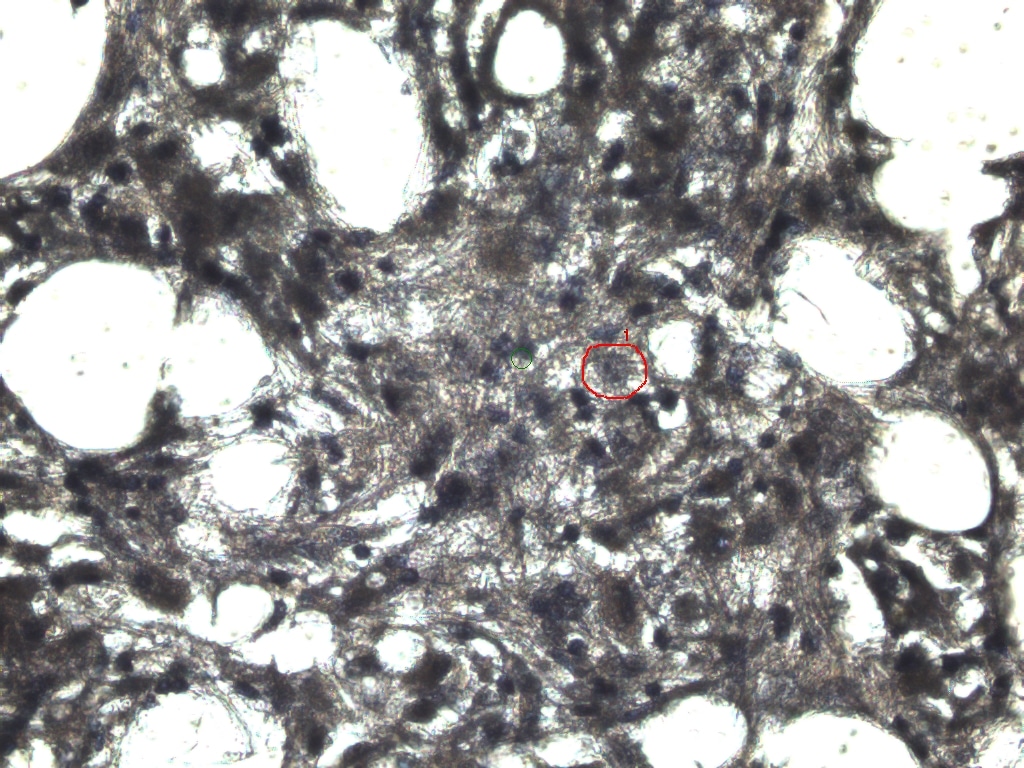

Supplement: Supplemental Material [file supp_gr.234807.118_Supplemental_File_4.zip › SINGLE CELL/Macrophage single cell/MAC3 BEFORE.jpeg]

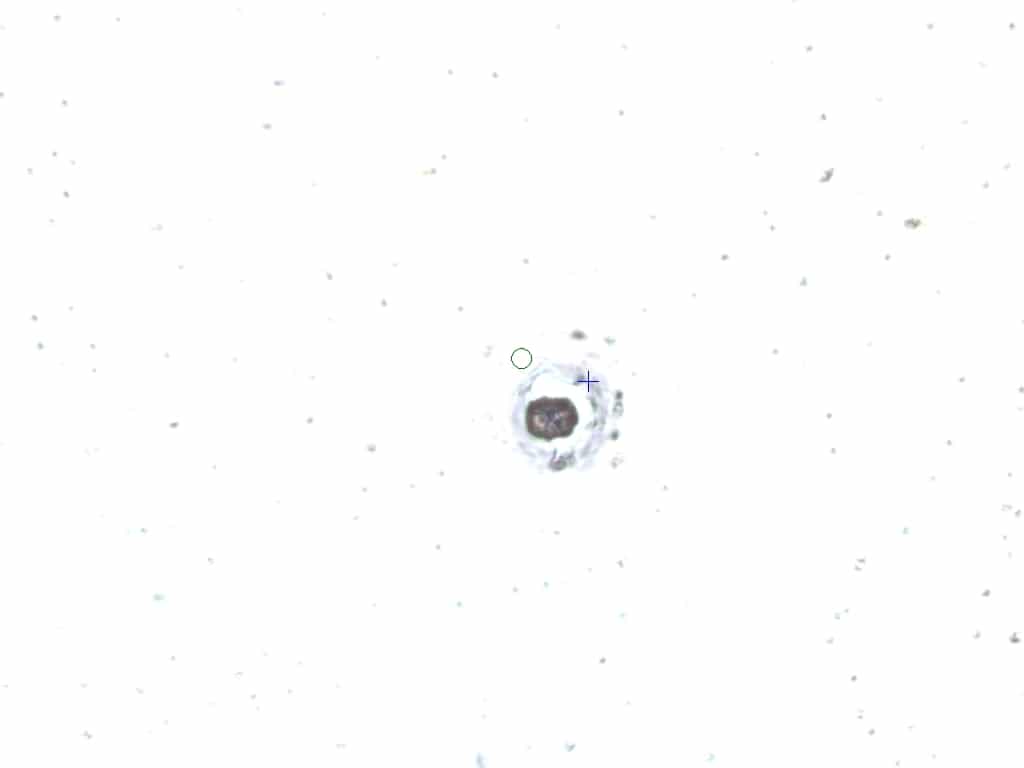

Supplement: Supplemental Material [file supp_gr.234807.118_Supplemental_File_4.zip › SINGLE CELL/Macrophage single cell/MAC3.jpeg]

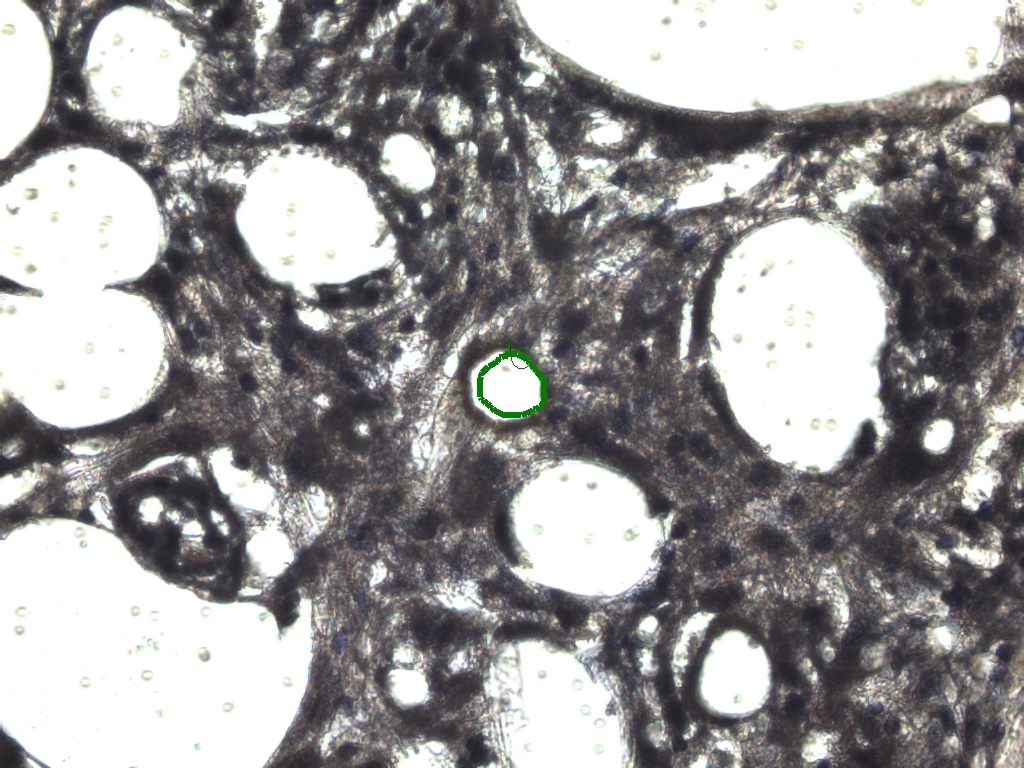

Supplement: Supplemental Material [file supp_gr.234807.118_Supplemental_File_4.zip › SINGLE CELL/Macrophage single cell/MAC4 AFTER.jpeg]

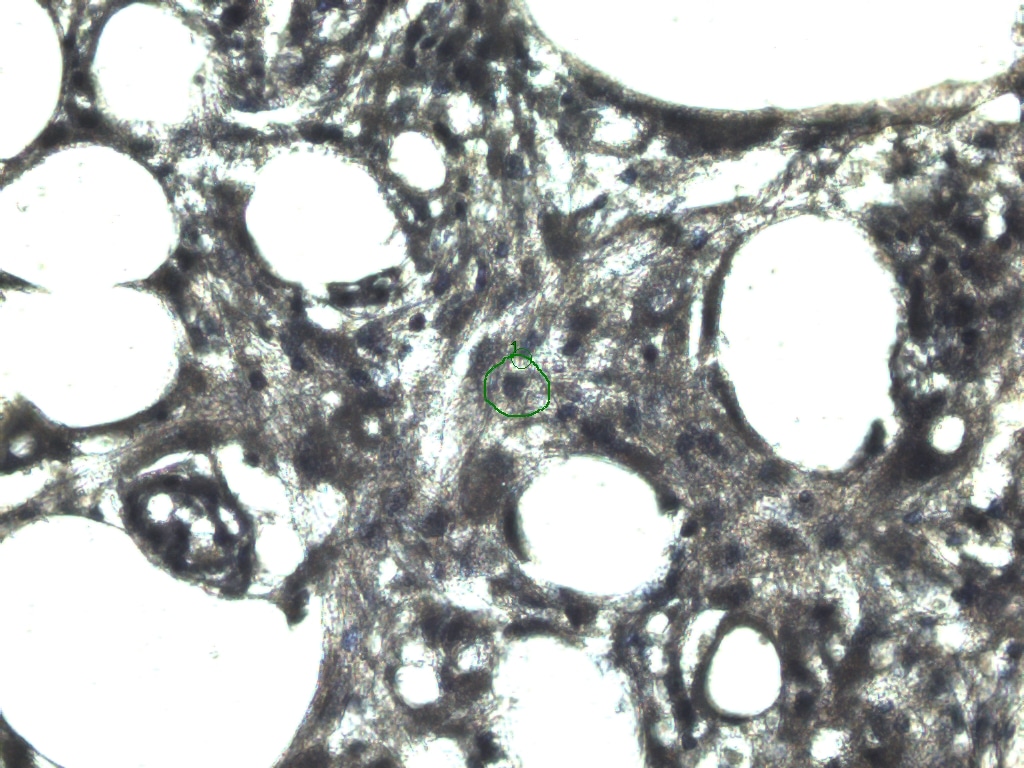

Supplement: Supplemental Material [file supp_gr.234807.118_Supplemental_File_4.zip › SINGLE CELL/Macrophage single cell/MAC4 BEFORE.jpeg]

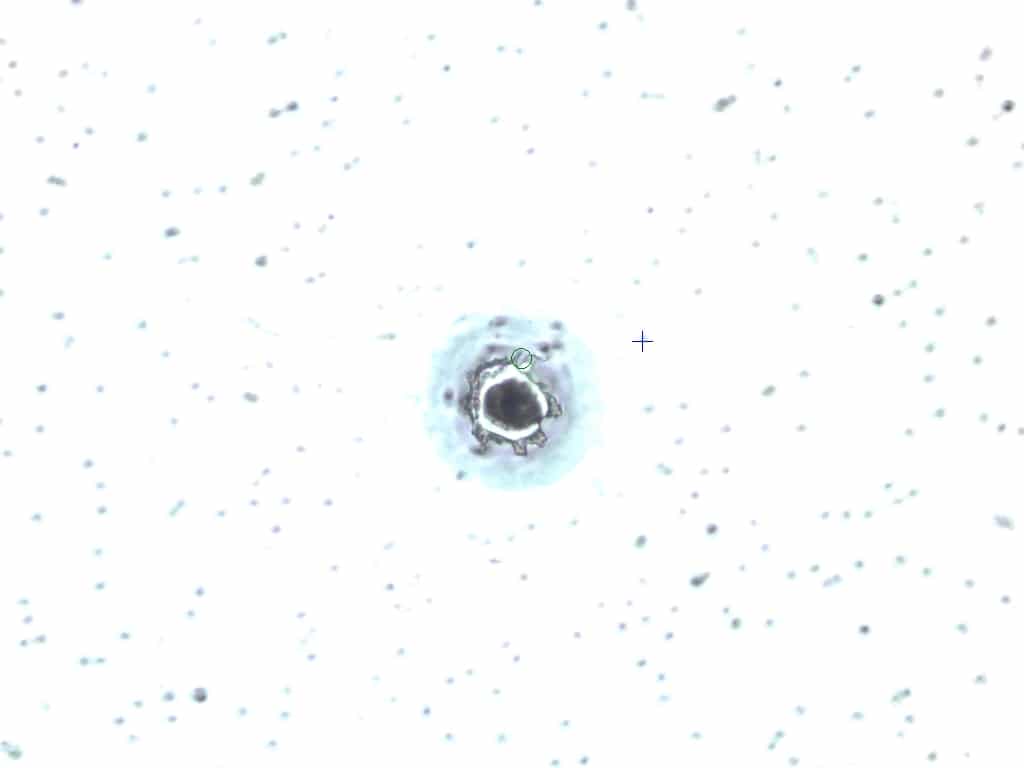

Supplement: Supplemental Material [file supp_gr.234807.118_Supplemental_File_4.zip › SINGLE CELL/Macrophage single cell/MAC4.jpeg]

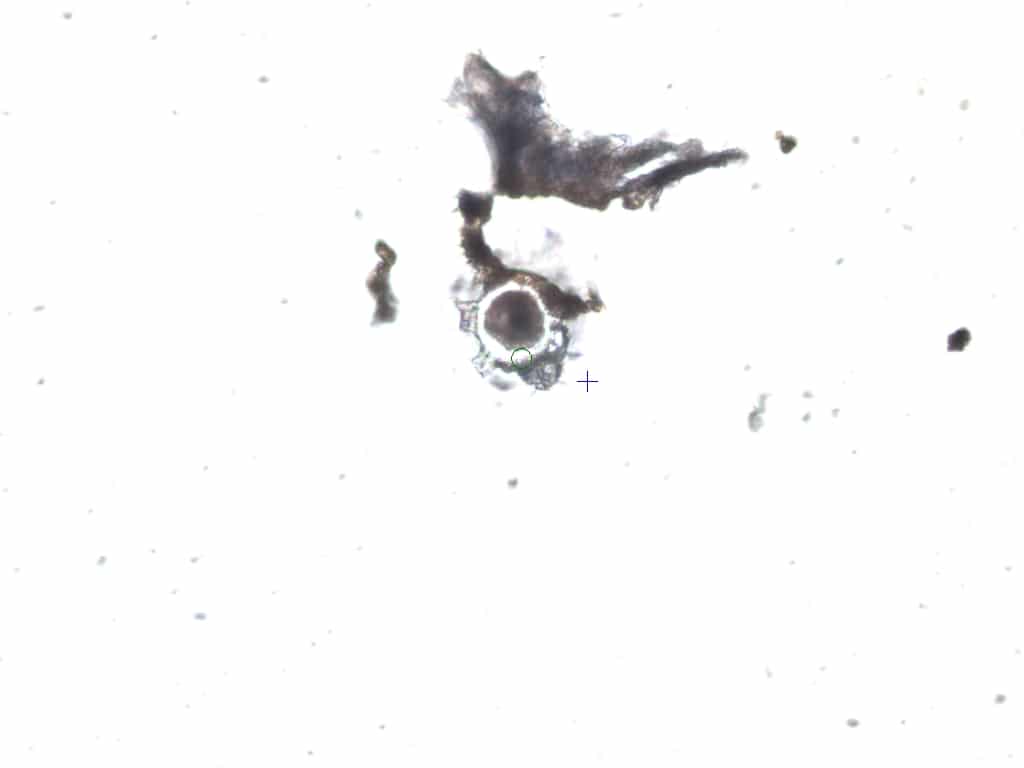

Supplement: Supplemental Material [file supp_gr.234807.118_Supplemental_File_4.zip › SINGLE CELL/Macrophage single cell/MAC5 AFTER ABLATION FINAL.jpeg]

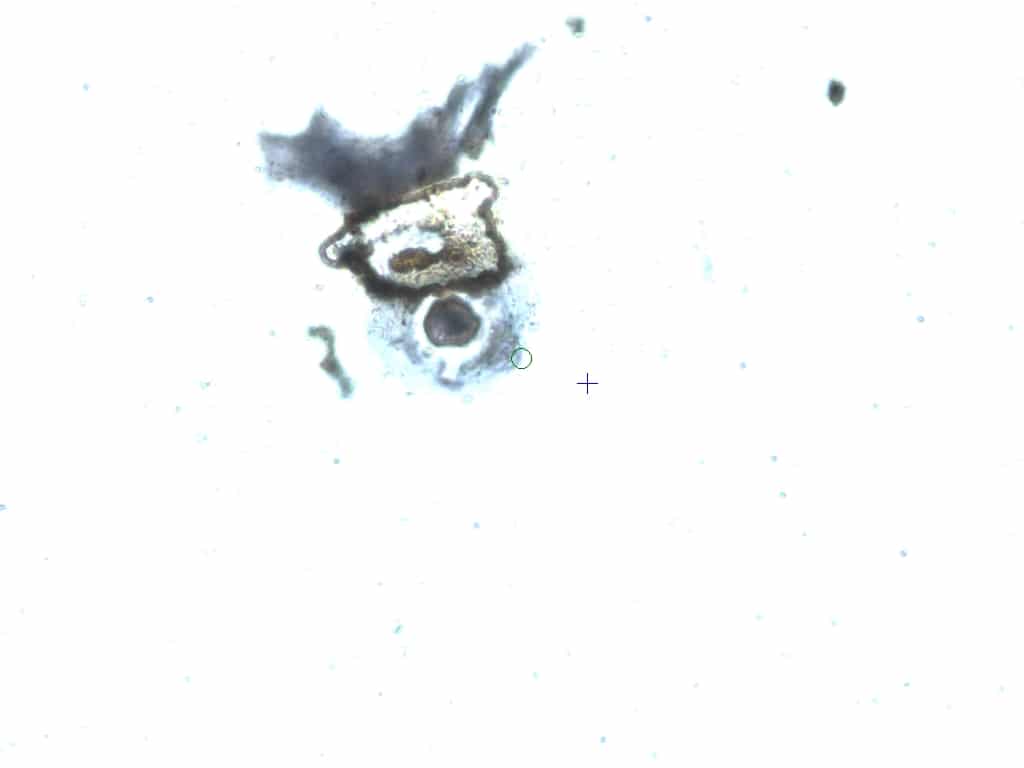

Supplement: Supplemental Material [file supp_gr.234807.118_Supplemental_File_4.zip › SINGLE CELL/Macrophage single cell/MAC5 AFTER ABLATION.jpeg]

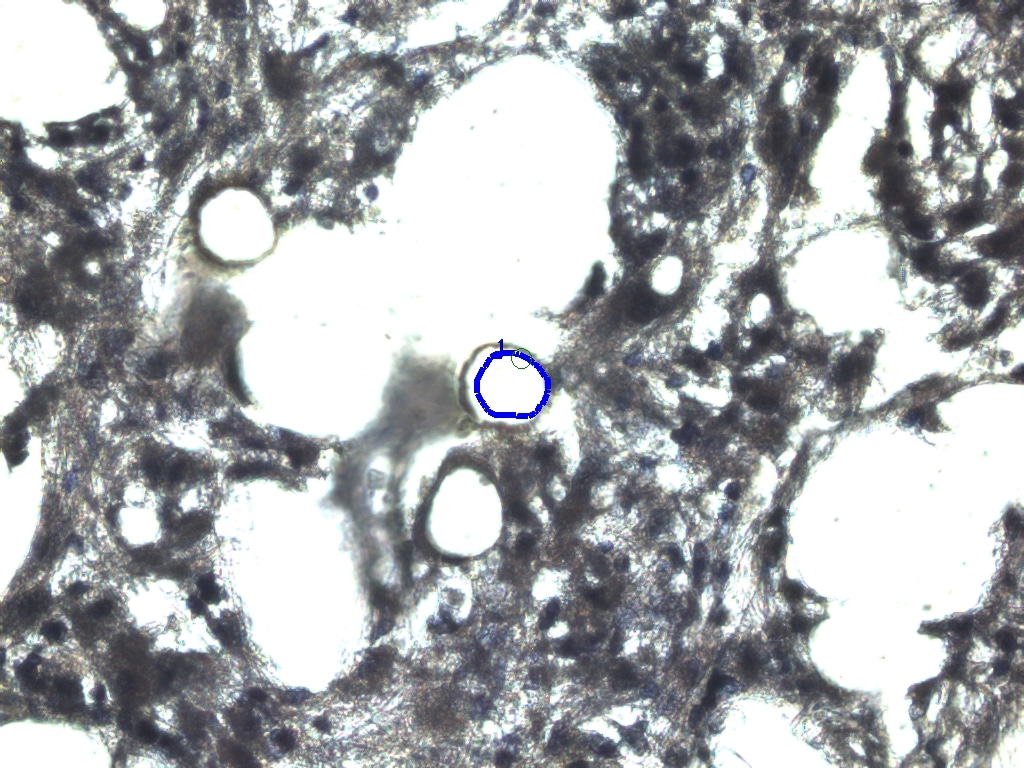

Supplement: Supplemental Material [file supp_gr.234807.118_Supplemental_File_4.zip › SINGLE CELL/Macrophage single cell/MAC5 AFTER.jpeg]

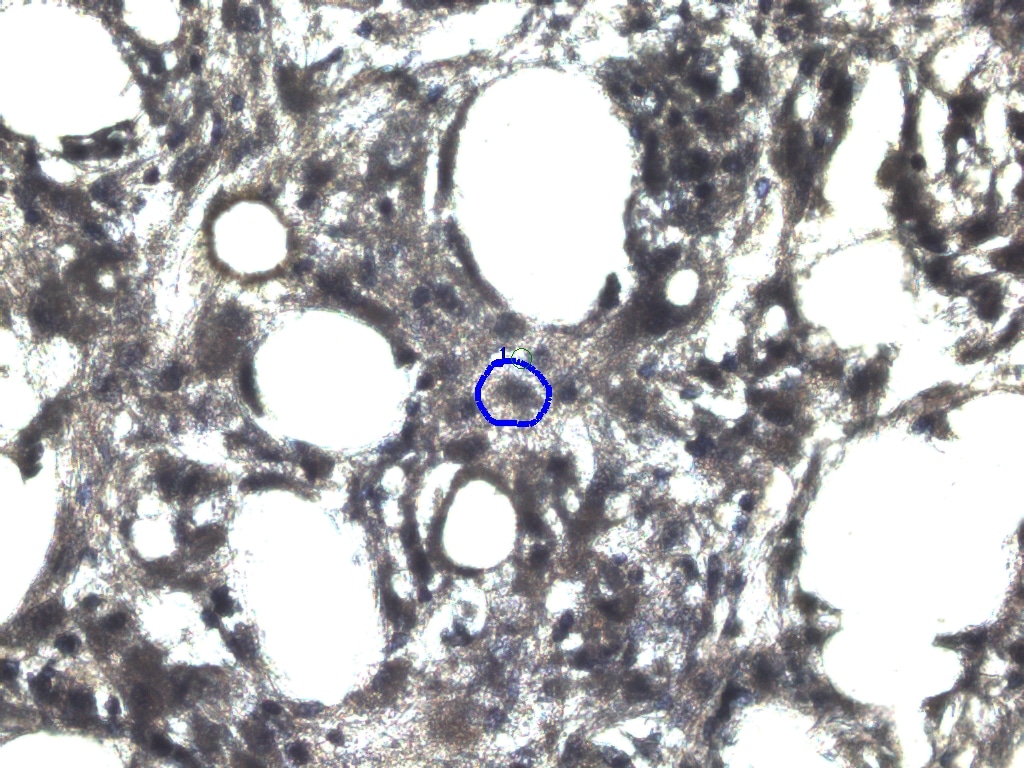

Supplement: Supplemental Material [file supp_gr.234807.118_Supplemental_File_4.zip › SINGLE CELL/Macrophage single cell/MAC5 BEFORE.jpeg]

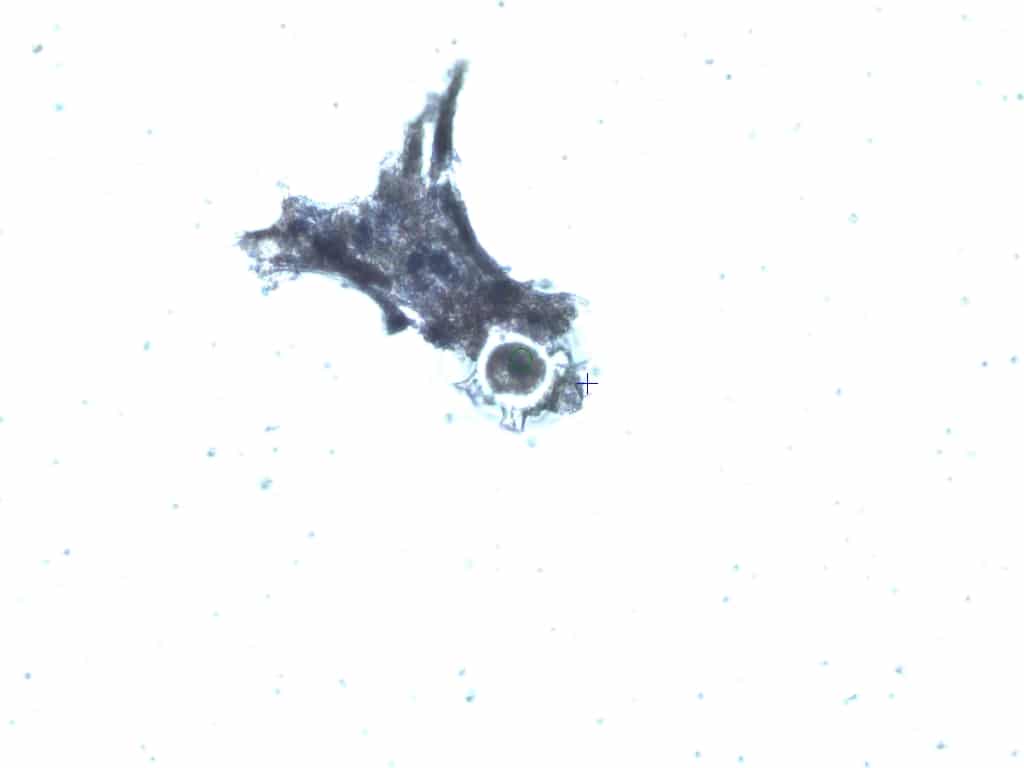

Supplement: Supplemental Material [file supp_gr.234807.118_Supplemental_File_4.zip › SINGLE CELL/Macrophage single cell/MAC5.jpeg]

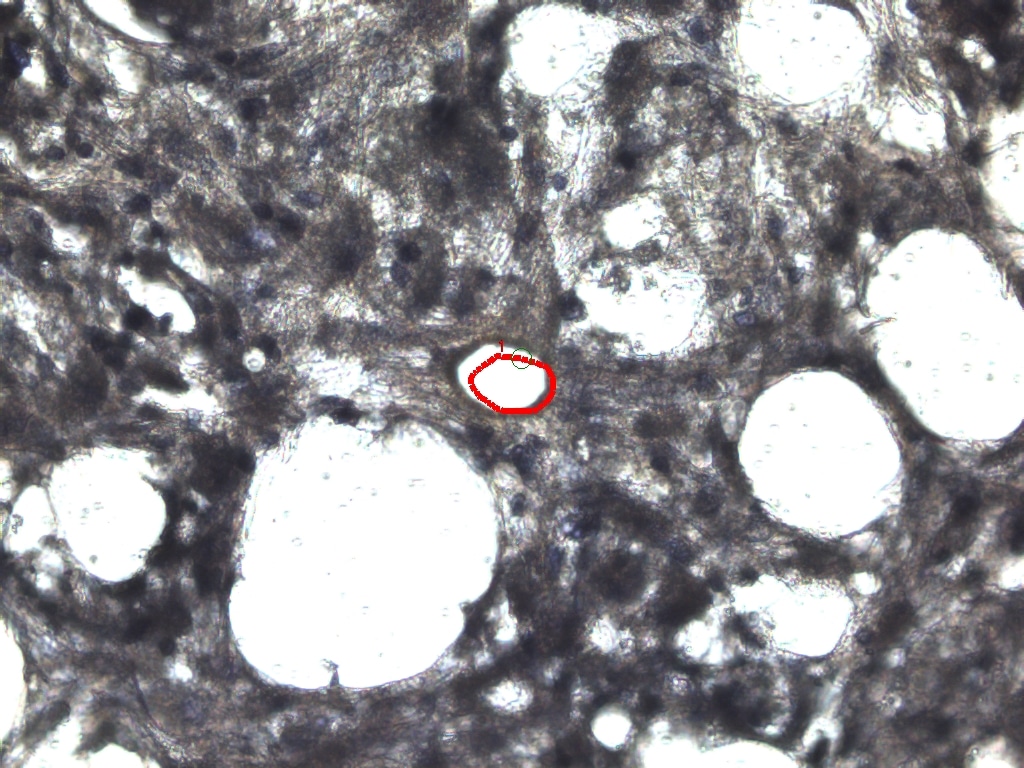

Supplement: Supplemental Material [file supp_gr.234807.118_Supplemental_File_4.zip › SINGLE CELL/Macrophage single cell/MAC6 AFTER.jpeg]

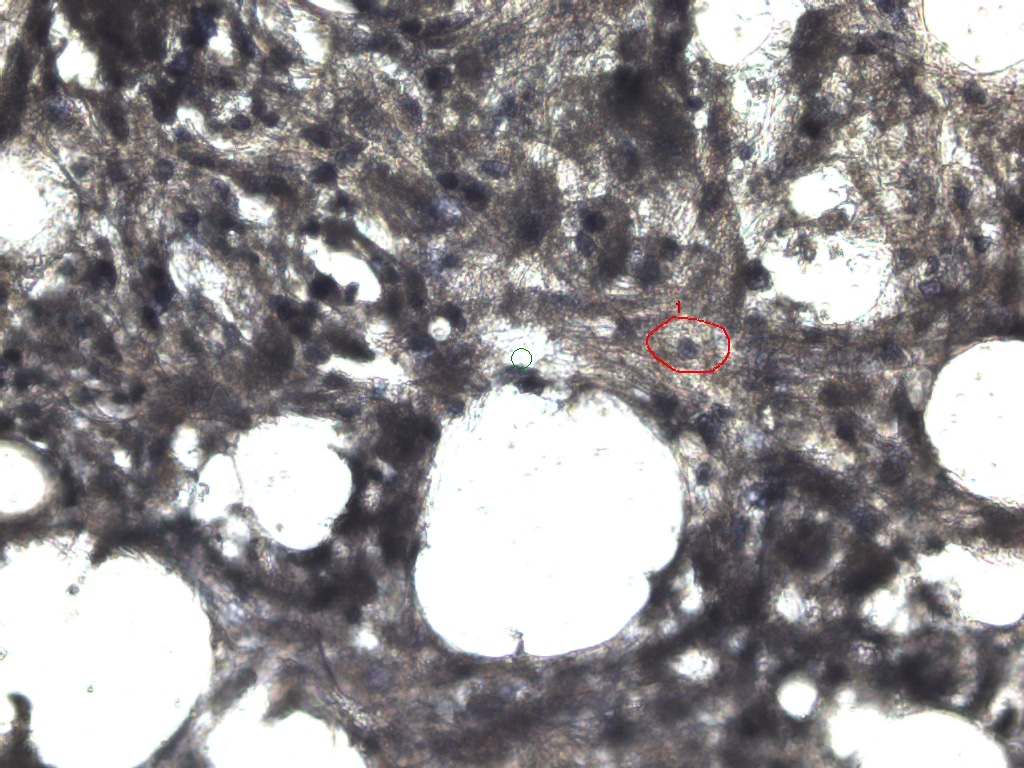

Supplement: Supplemental Material [file supp_gr.234807.118_Supplemental_File_4.zip › SINGLE CELL/Macrophage single cell/MAC6 BEFORE.jpeg]

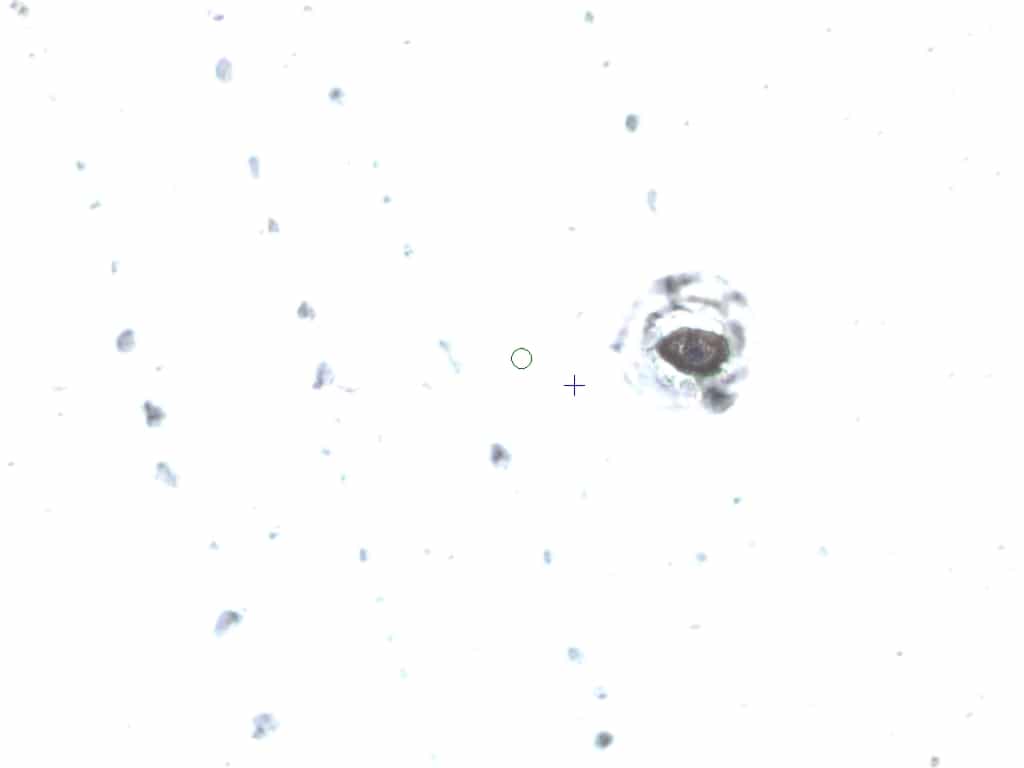

Supplement: Supplemental Material [file supp_gr.234807.118_Supplemental_File_4.zip › SINGLE CELL/Macrophage single cell/MAC6.jpeg]

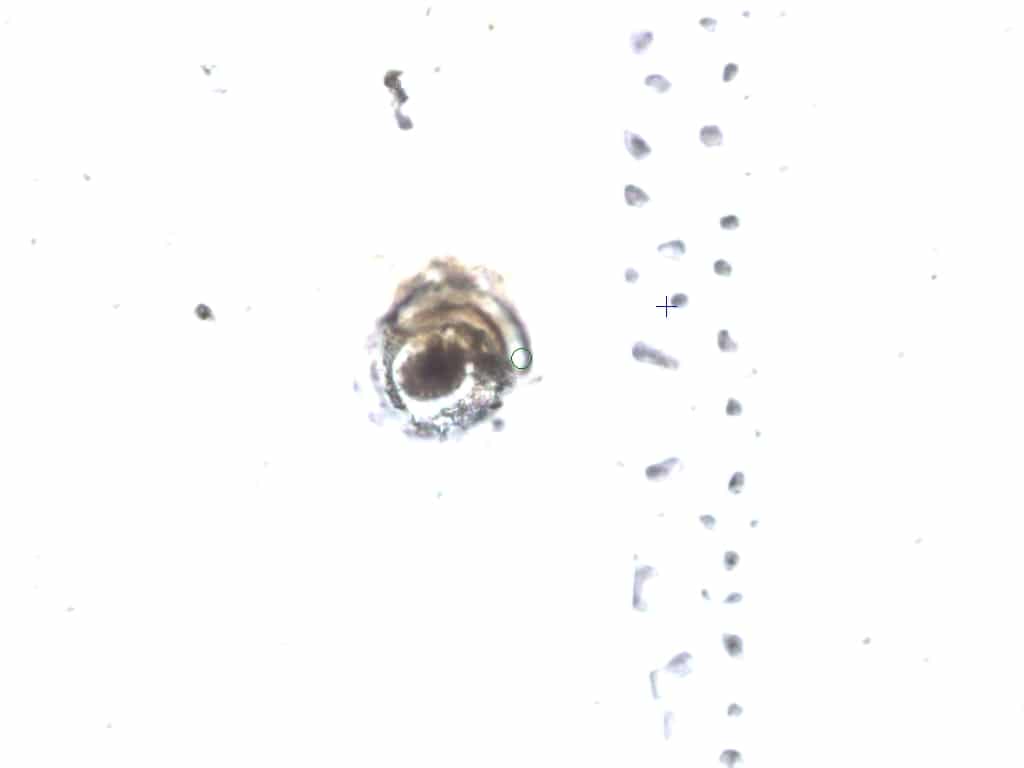

Supplement: Supplemental Material [file supp_gr.234807.118_Supplemental_File_4.zip › SINGLE CELL/Macrophage single cell/MAC7 AFTER ABLATION.jpeg]

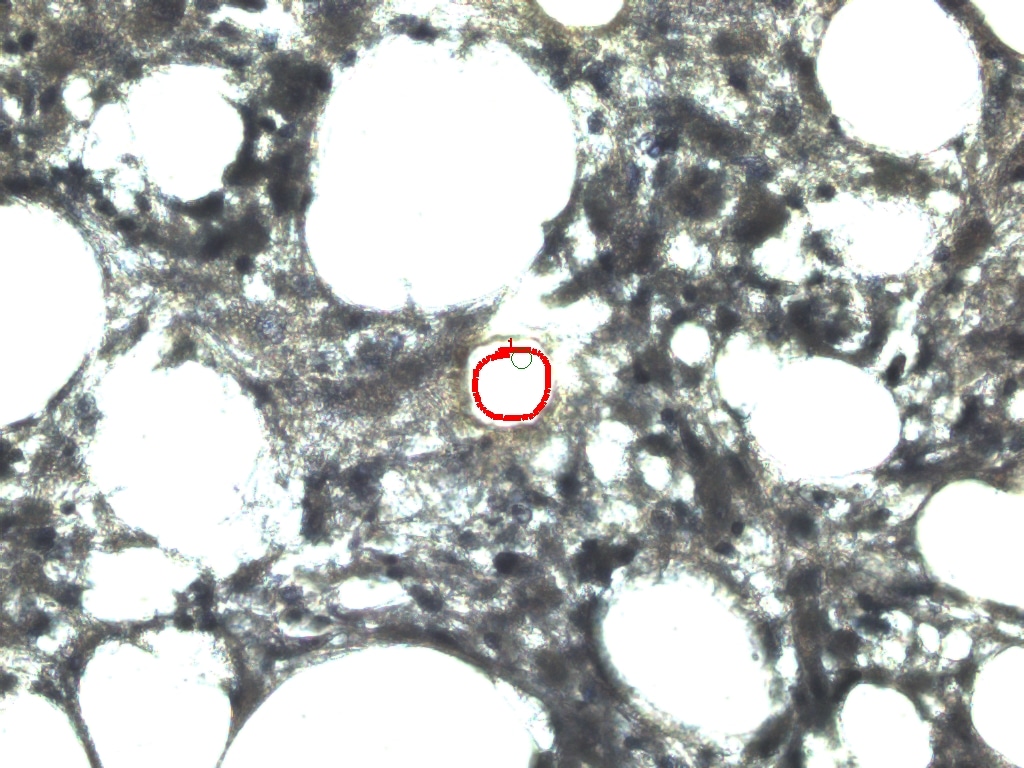

Supplement: Supplemental Material [file supp_gr.234807.118_Supplemental_File_4.zip › SINGLE CELL/Macrophage single cell/MAC7 AFTER.jpeg]

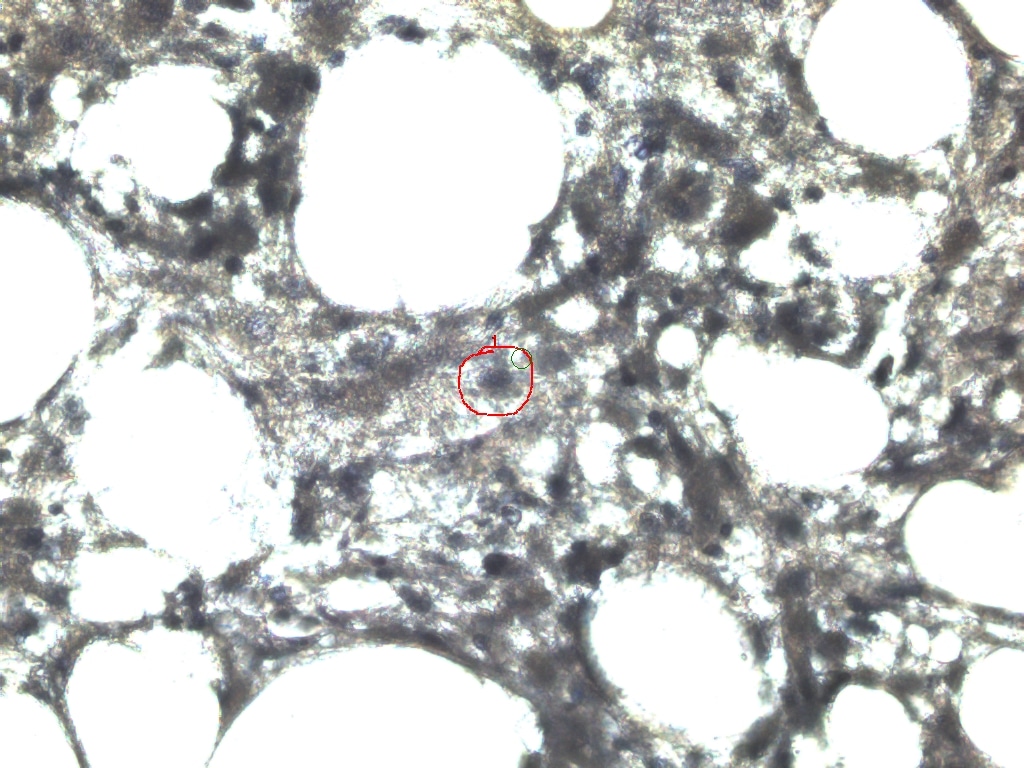

Supplement: Supplemental Material [file supp_gr.234807.118_Supplemental_File_4.zip › SINGLE CELL/Macrophage single cell/MAC7 BEFORE.jpeg]

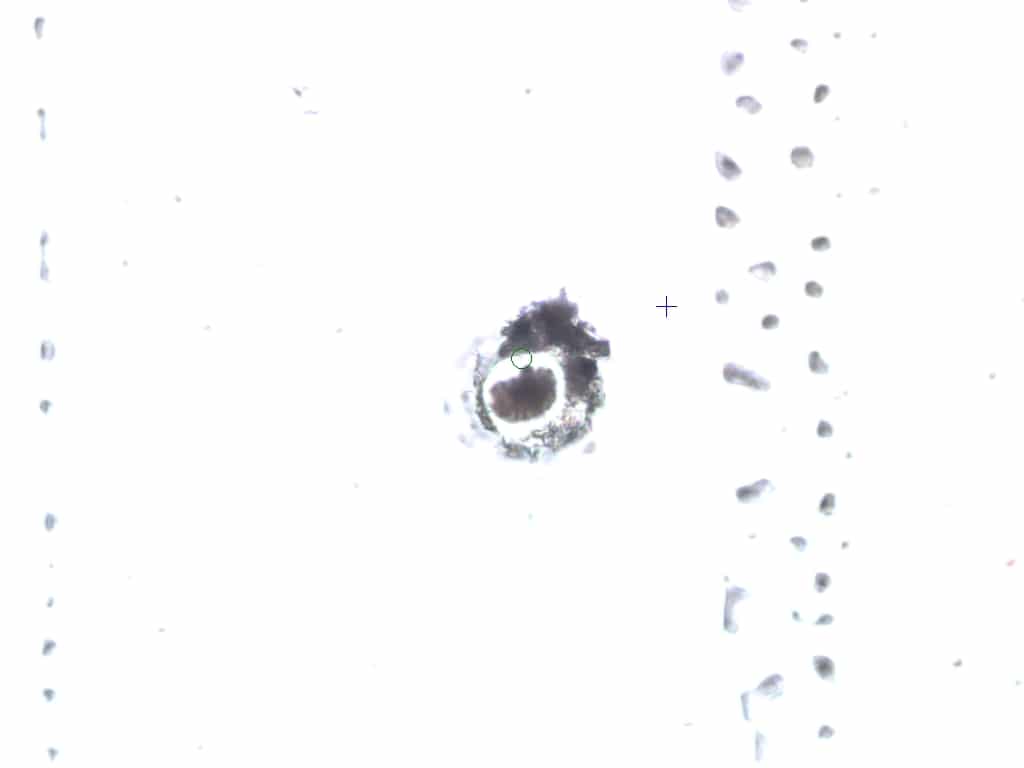

Supplement: Supplemental Material [file supp_gr.234807.118_Supplemental_File_4.zip › SINGLE CELL/Macrophage single cell/MAC7.jpeg]

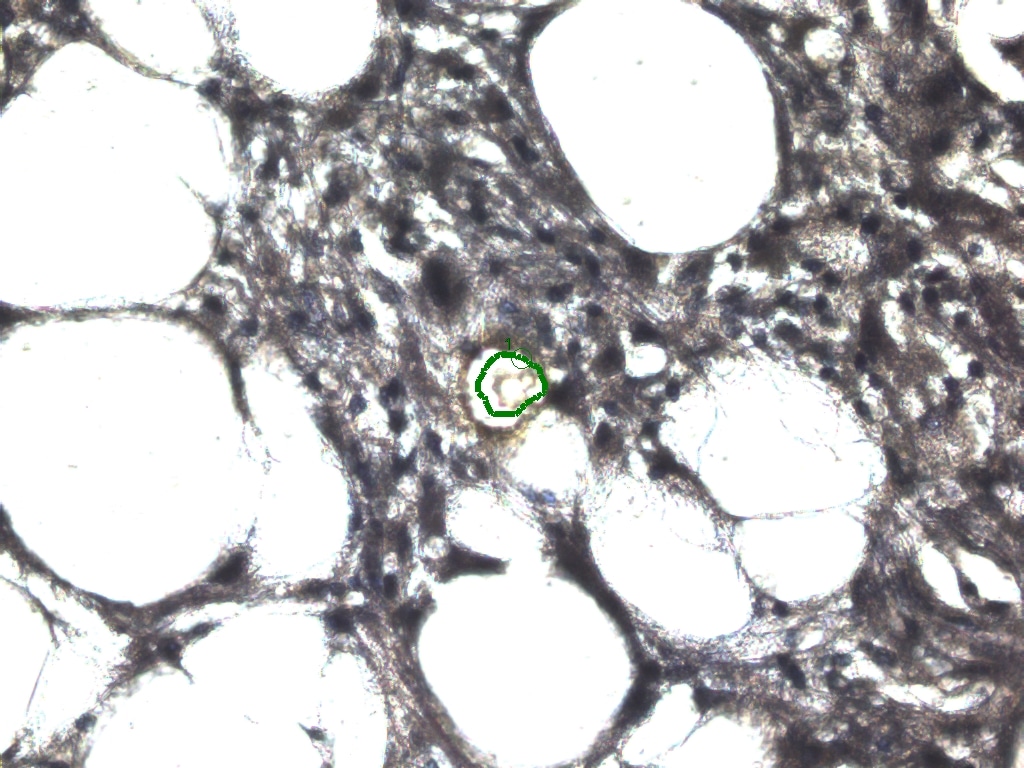

Supplement: Supplemental Material [file supp_gr.234807.118_Supplemental_File_4.zip › SINGLE CELL/Macrophage single cell/MAC8 AFTER.jpeg]

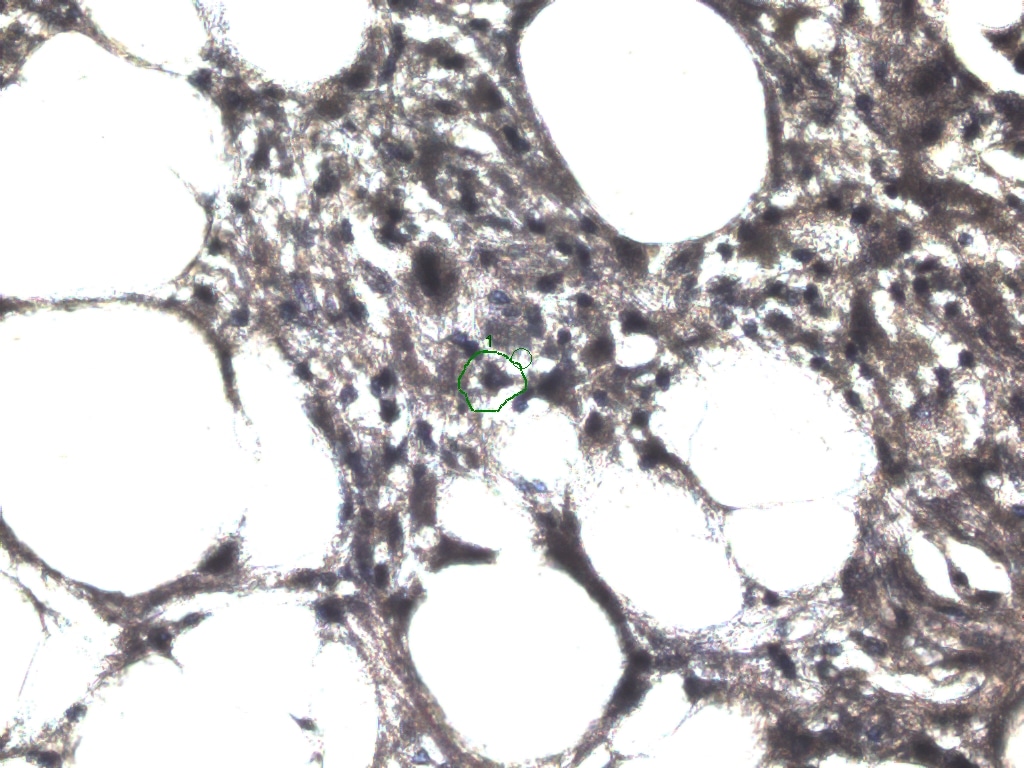

Supplement: Supplemental Material [file supp_gr.234807.118_Supplemental_File_4.zip › SINGLE CELL/Macrophage single cell/MAC8 BEFORE.jpeg]

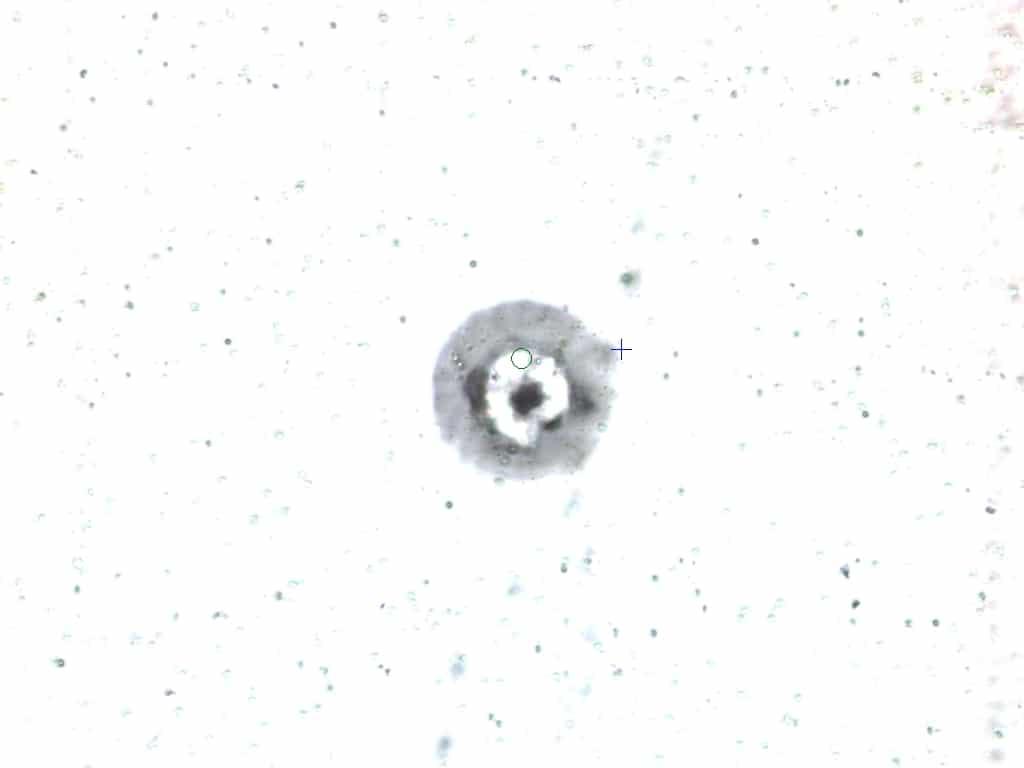

Supplement: Supplemental Material [file supp_gr.234807.118_Supplemental_File_4.zip › SINGLE CELL/Macrophage single cell/MAC8.jpeg]

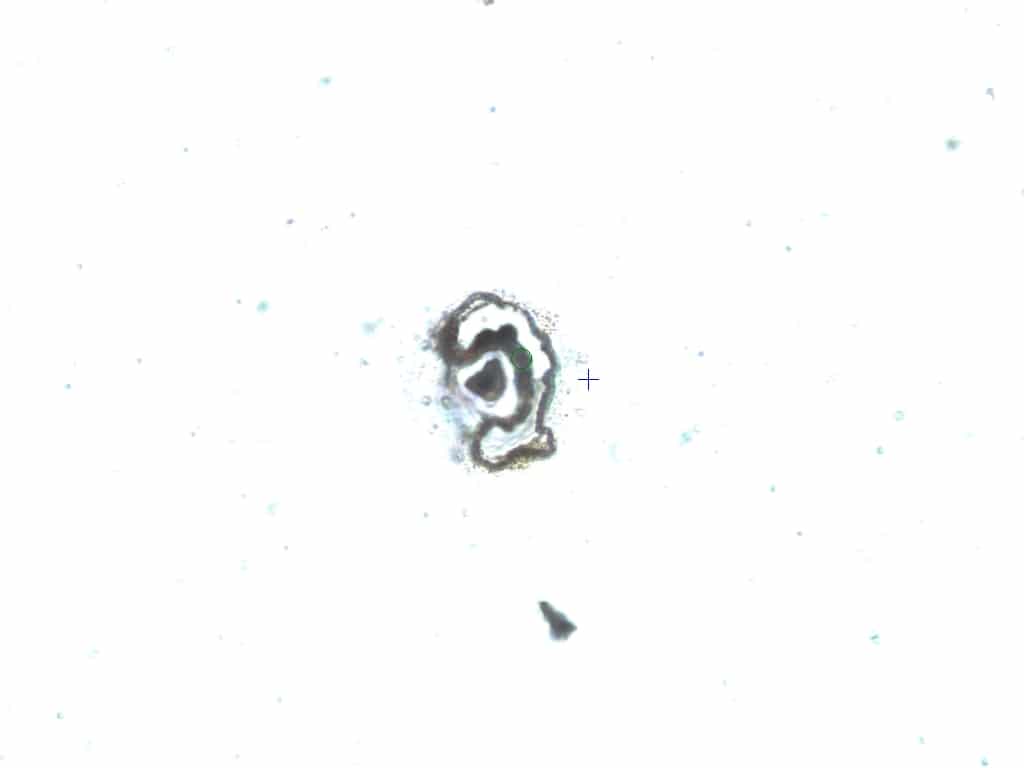

Supplement: Supplemental Material [file supp_gr.234807.118_Supplemental_File_4.zip › SINGLE CELL/Macrophage single cell/MAC9 AFTER ABLATION.jpeg]

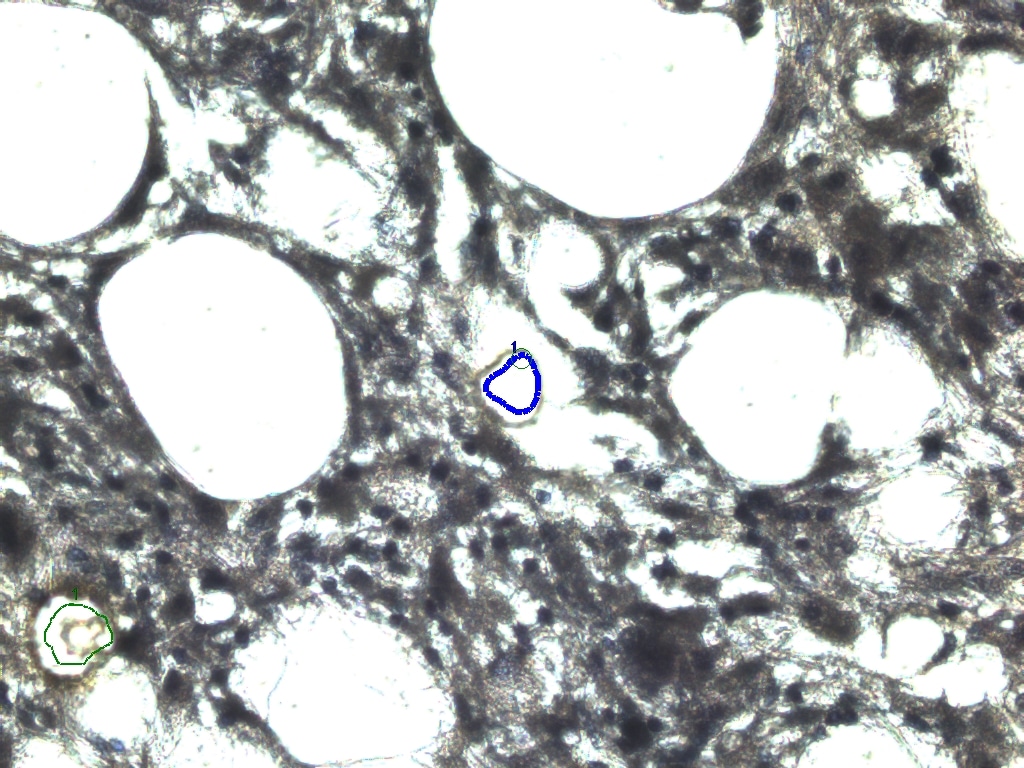

Supplement: Supplemental Material [file supp_gr.234807.118_Supplemental_File_4.zip › SINGLE CELL/Macrophage single cell/MAC9 AFTER.jpeg]

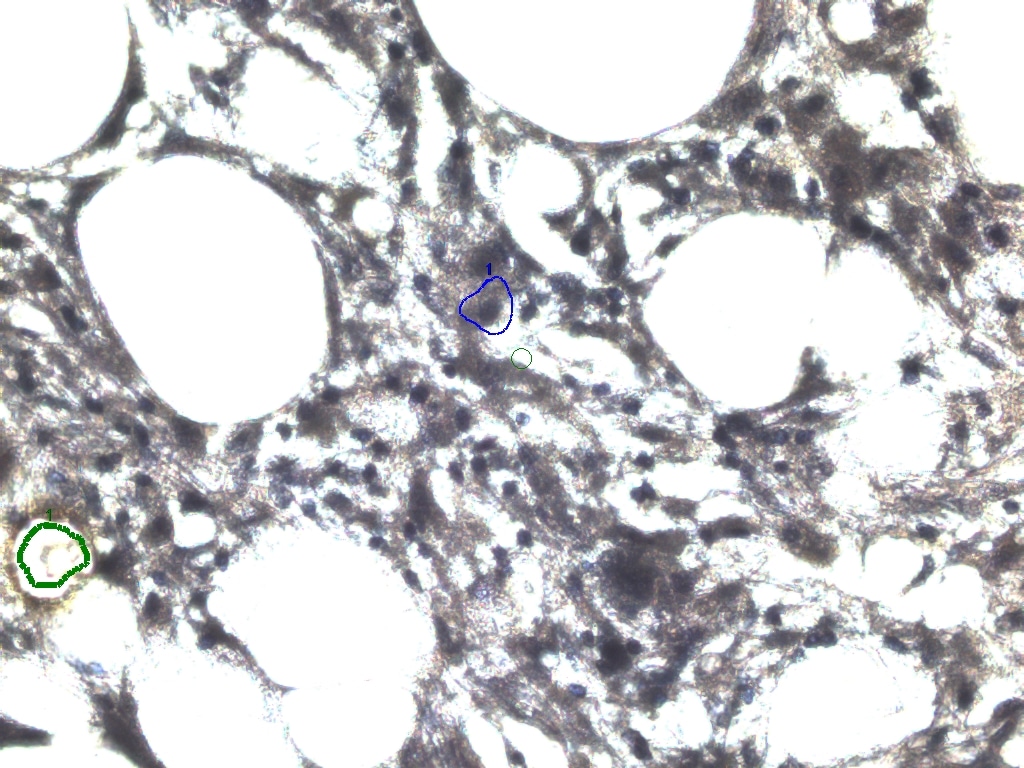

Supplement: Supplemental Material [file supp_gr.234807.118_Supplemental_File_4.zip › SINGLE CELL/Macrophage single cell/MAC9 BEFORE.jpeg]

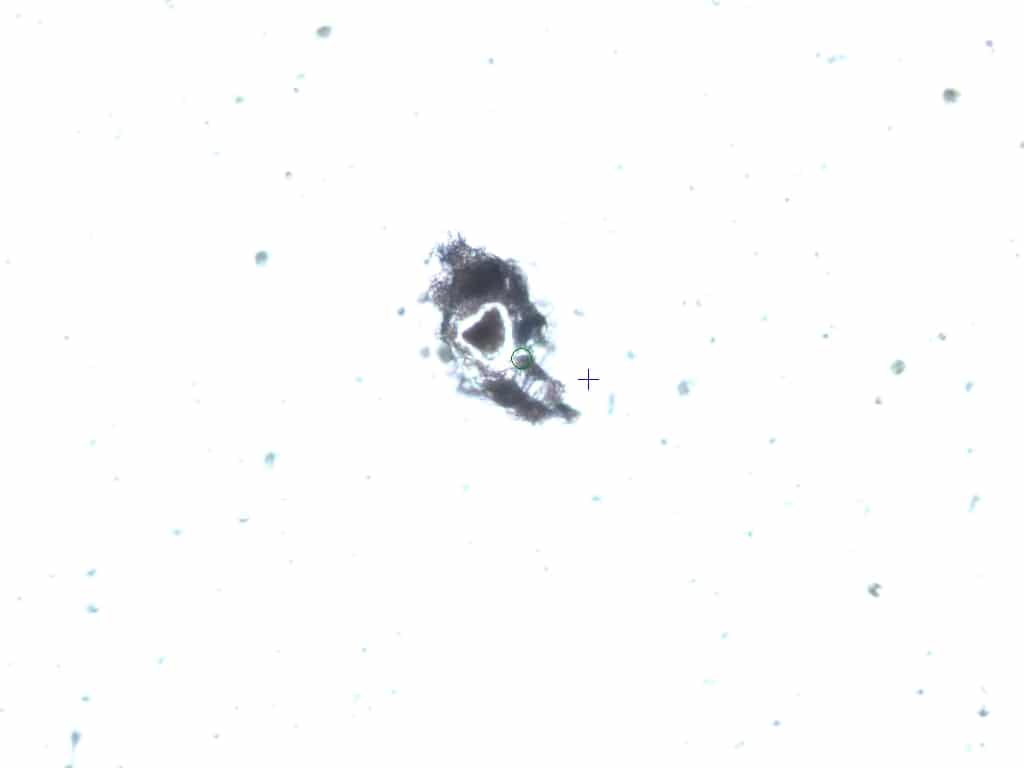

Supplement: Supplemental Material [file supp_gr.234807.118_Supplemental_File_4.zip › SINGLE CELL/Macrophage single cell/MAC9.jpeg]
