# Supplementary figures and images for: Gene expression profiling of single cells from archival tissue with laser-capture microdissection and Smart-3SEQ (part 1 of 2)
Source: Genome Res. 2019 Nov;29(11):1816–25. doi: 10.1101/gr.234807.118 (PMC6836736; doi:10.1101/gr.234807.118)

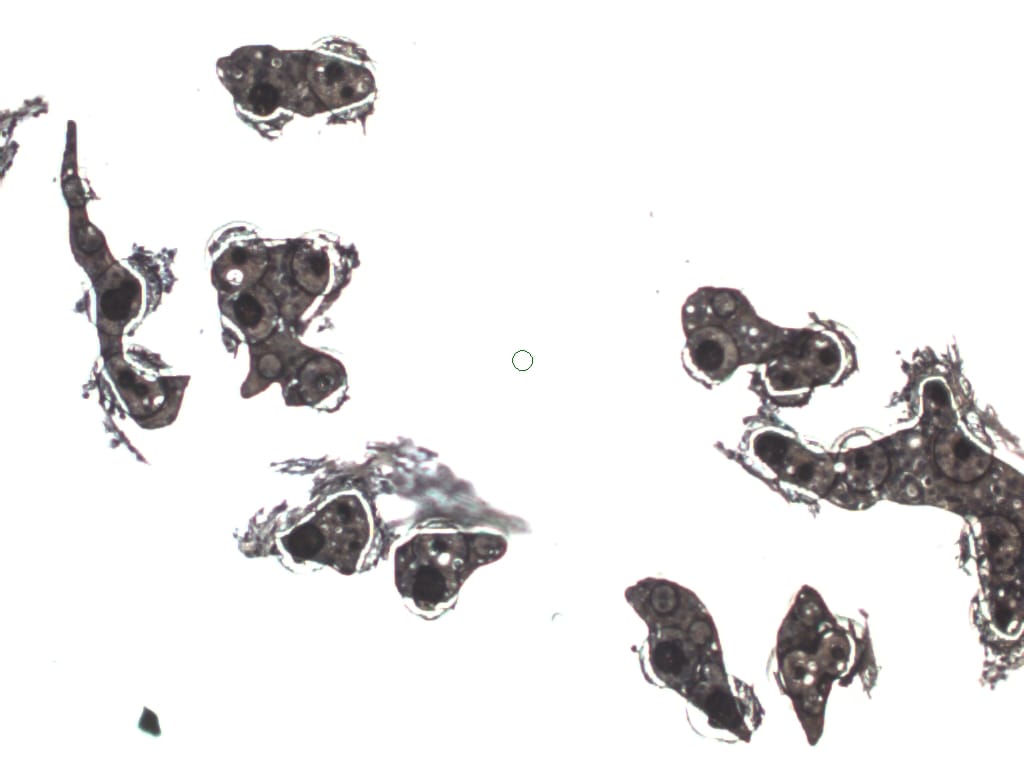

Supplement: Supplemental Material [file supp_gr.234807.118_Supplemental_File_4.zip › ABLATION/ABLATION FOR DCIS/DCIS1 .jpeg]

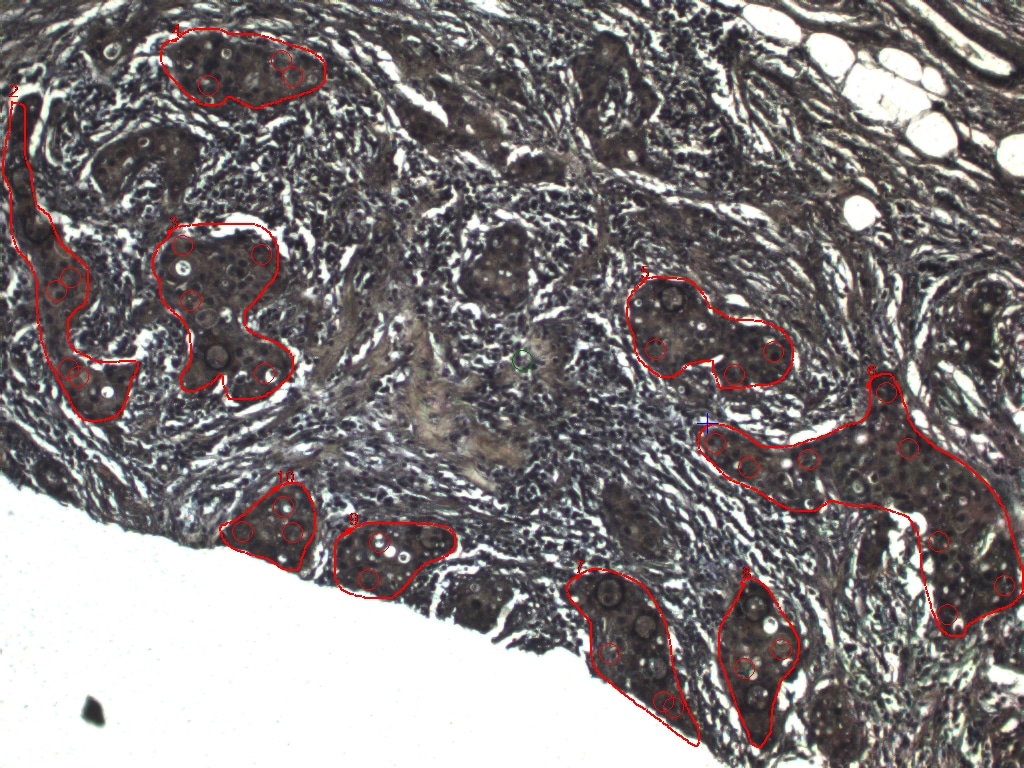

Supplement: Supplemental Material [file supp_gr.234807.118_Supplemental_File_4.zip › ABLATION/ABLATION FOR DCIS/DCIS1 BEFORE.jpeg]

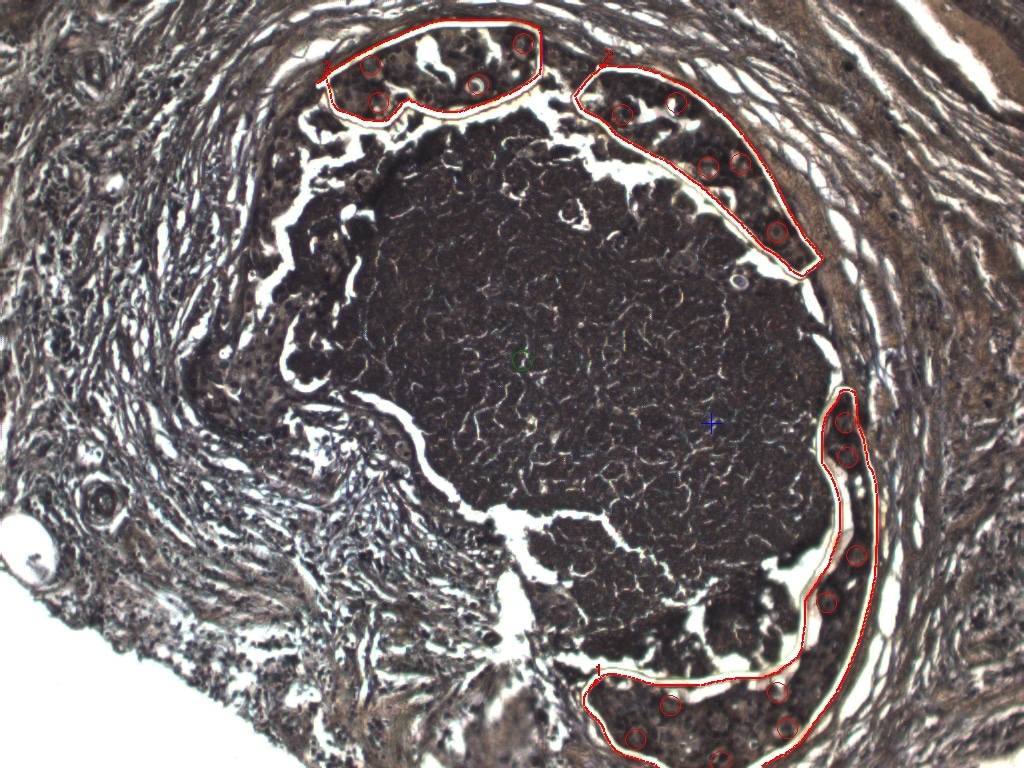

Supplement: Supplemental Material [file supp_gr.234807.118_Supplemental_File_4.zip › ABLATION/ABLATION FOR DCIS/DCIS1-2 BEFORE.jpeg]

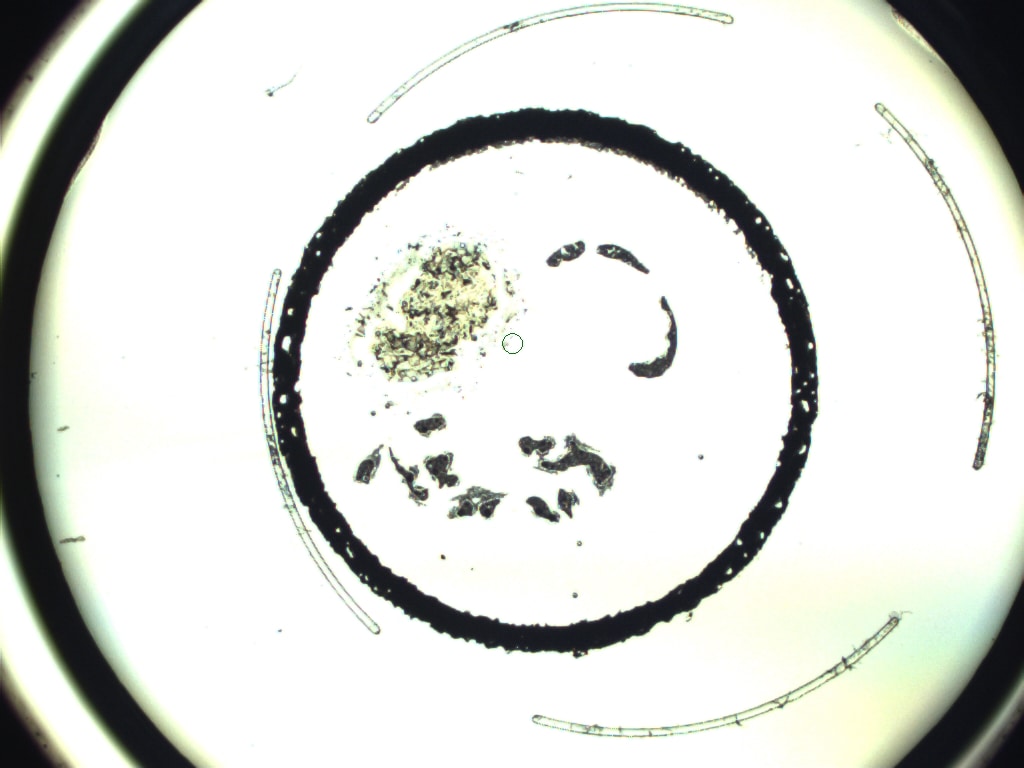

Supplement: Supplemental Material [file supp_gr.234807.118_Supplemental_File_4.zip › ABLATION/ABLATION FOR DCIS/DCIS1MAC1 ABLATION .jpeg]

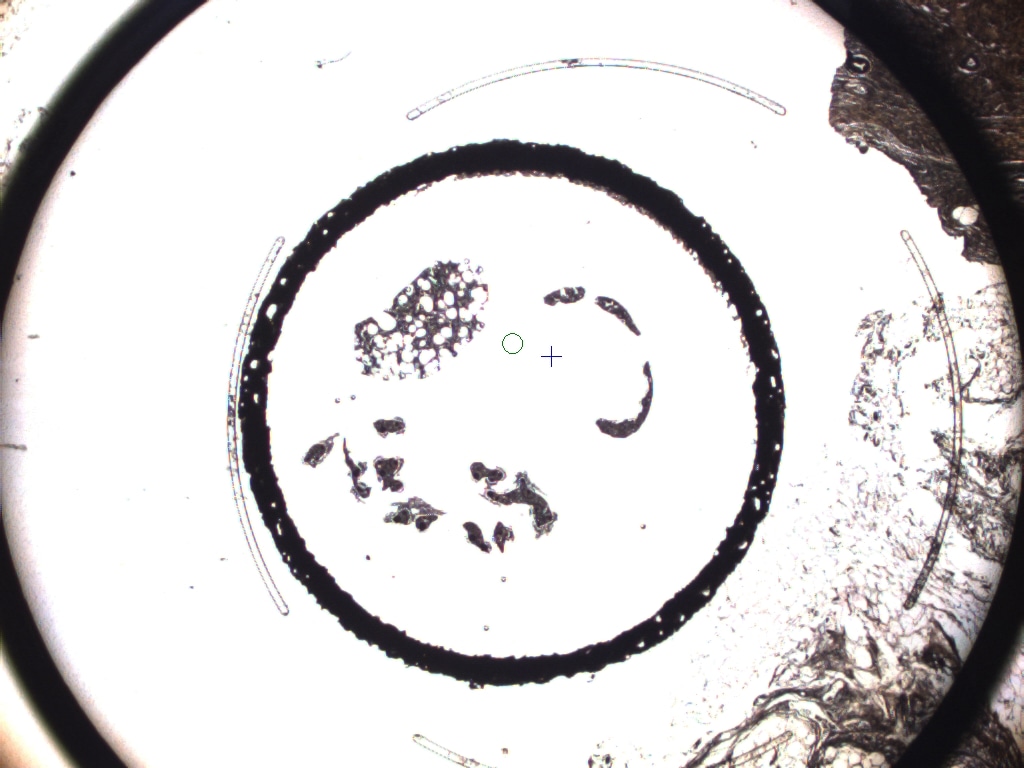

Supplement: Supplemental Material [file supp_gr.234807.118_Supplemental_File_4.zip › ABLATION/ABLATION FOR DCIS/DCIS1MAC1 BEFORE .jpeg]

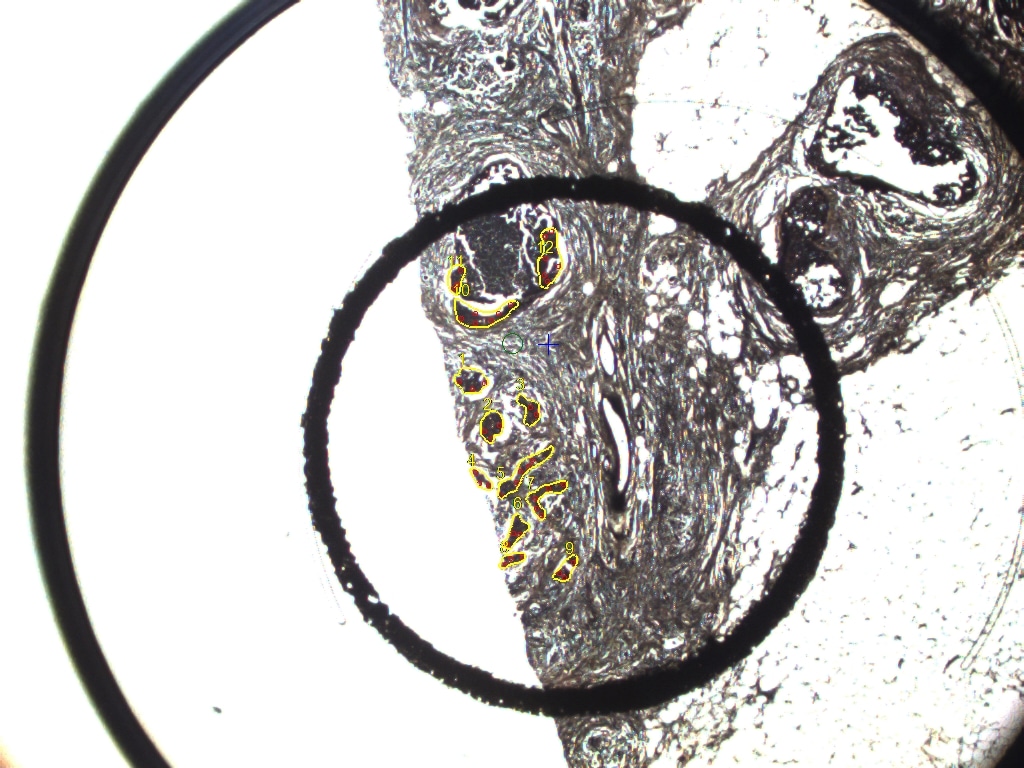

Supplement: Supplemental Material [file supp_gr.234807.118_Supplemental_File_4.zip › ABLATION/ABLATION FOR DCIS/DCIS2 BEFORE.jpeg]

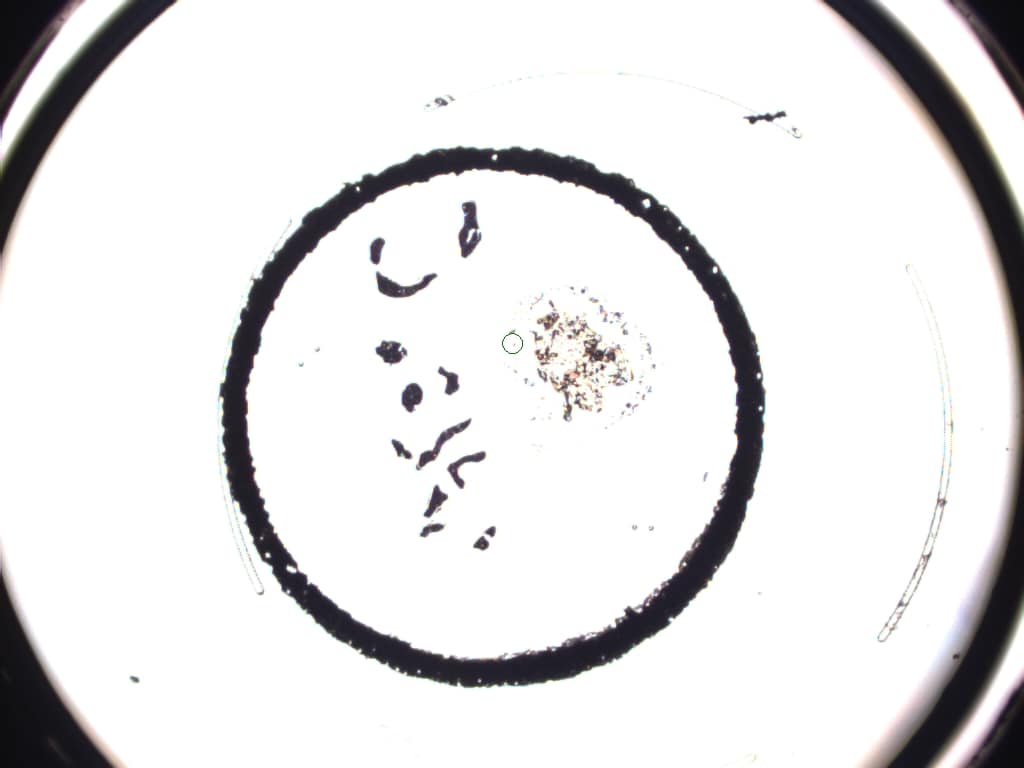

Supplement: Supplemental Material [file supp_gr.234807.118_Supplemental_File_4.zip › ABLATION/ABLATION FOR DCIS/DCIS2MAC2 ABLATION .jpeg]

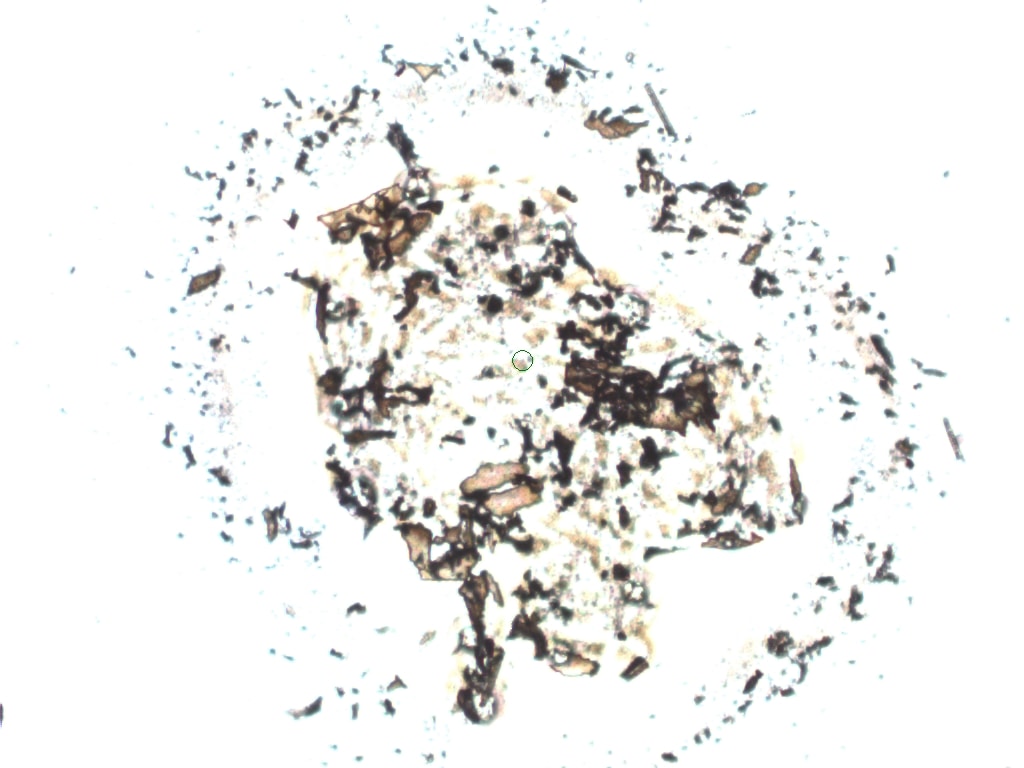

Supplement: Supplemental Material [file supp_gr.234807.118_Supplemental_File_4.zip › ABLATION/ABLATION FOR DCIS/DCIS2MAC2 ABLATION 10x .jpeg]

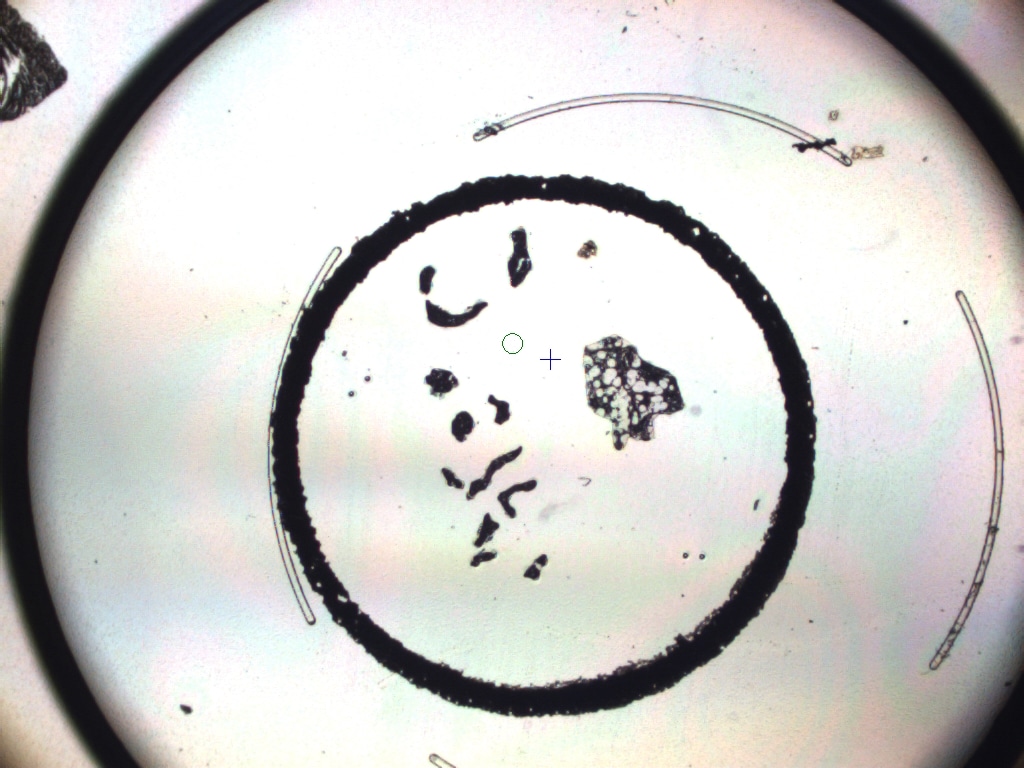

Supplement: Supplemental Material [file supp_gr.234807.118_Supplemental_File_4.zip › ABLATION/ABLATION FOR DCIS/DCIS2MAC2 BEFORE .jpeg]

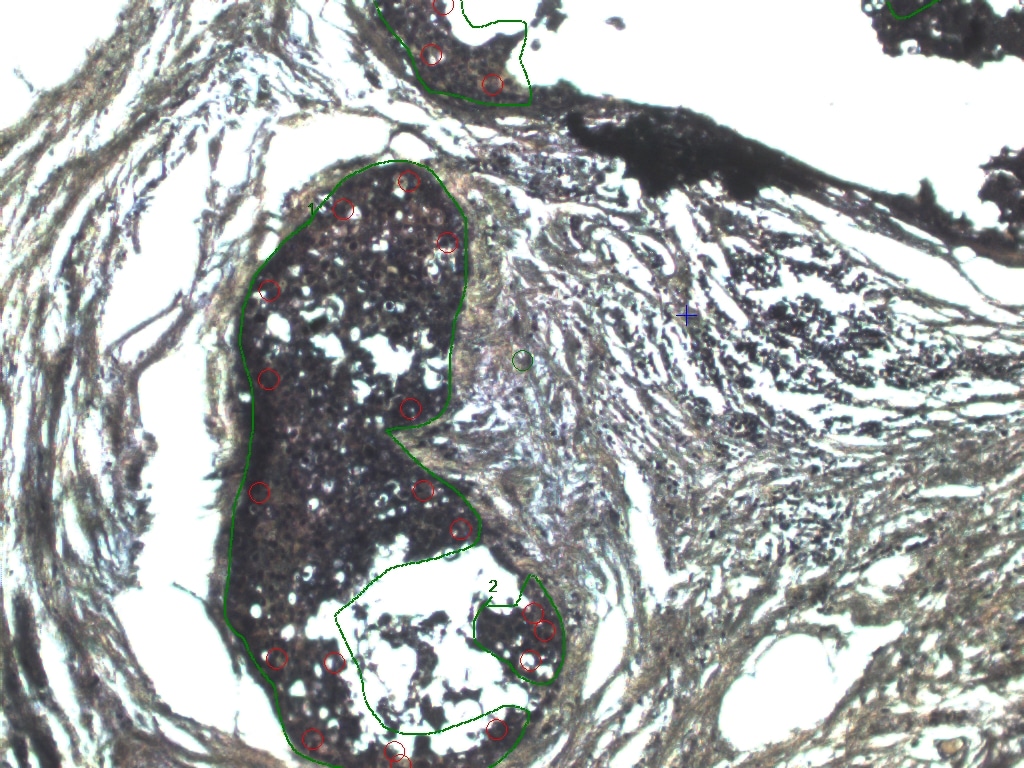

Supplement: Supplemental Material [file supp_gr.234807.118_Supplemental_File_4.zip › ABLATION/ABLATION FOR DCIS/DCIS3 BEFORE.jpeg]

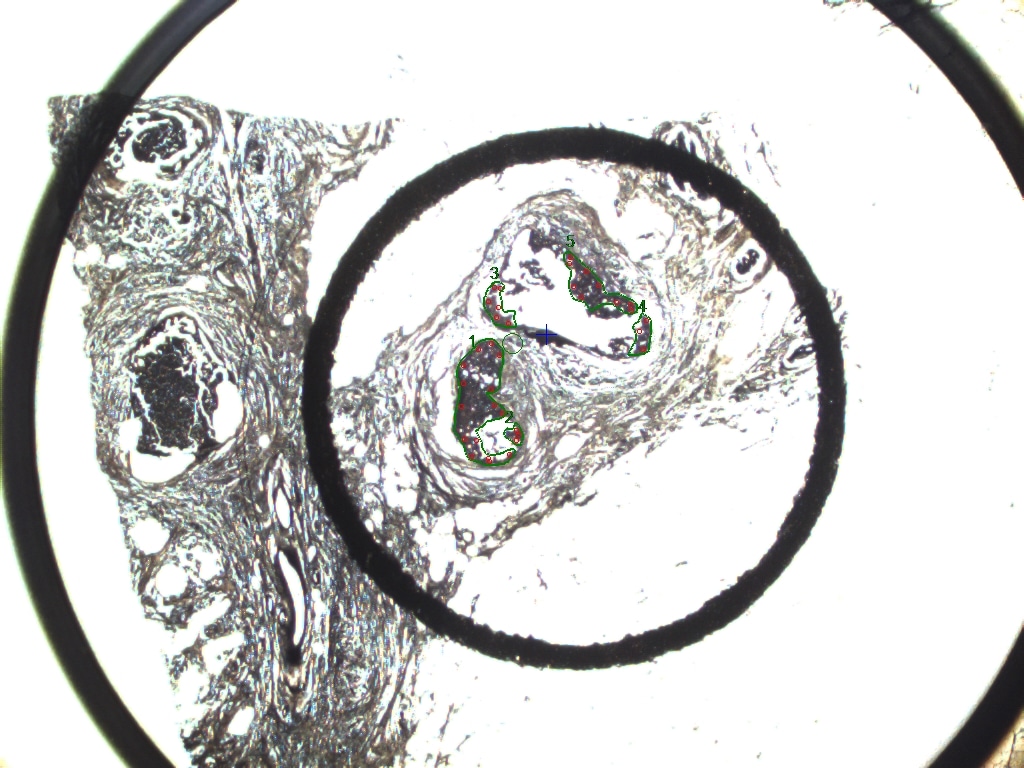

Supplement: Supplemental Material [file supp_gr.234807.118_Supplemental_File_4.zip › ABLATION/ABLATION FOR DCIS/DCIS3 BEFORE2X.jpeg]

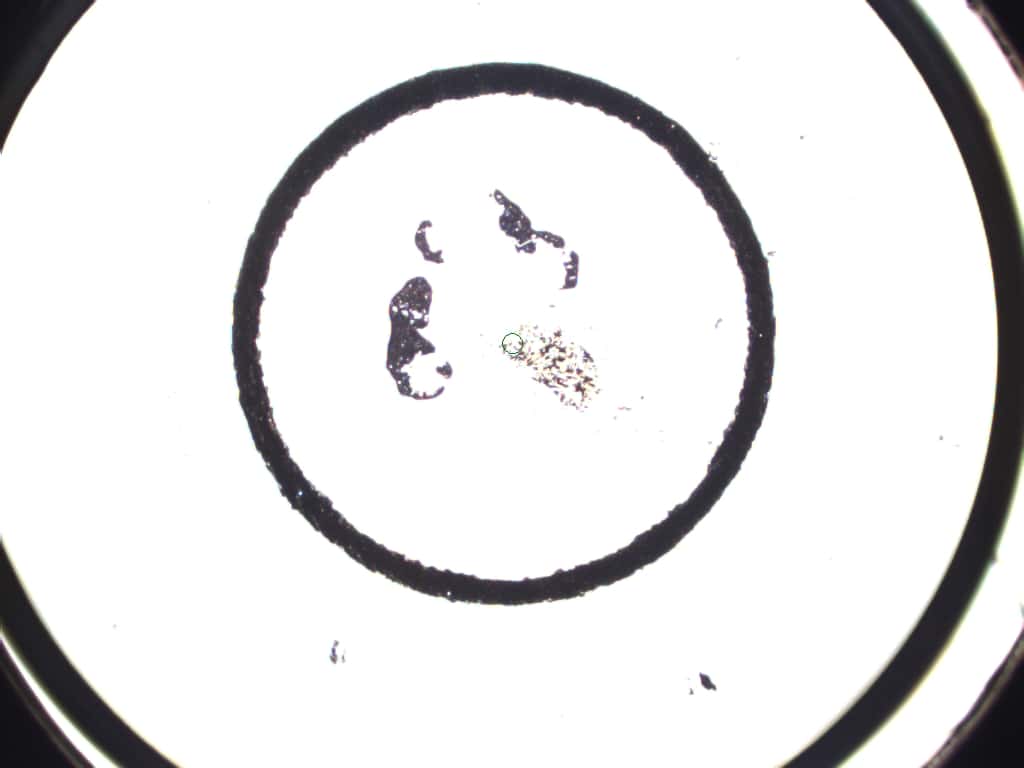

Supplement: Supplemental Material [file supp_gr.234807.118_Supplemental_File_4.zip › ABLATION/ABLATION FOR DCIS/DCIS3MAC3 ABLATION .jpeg]

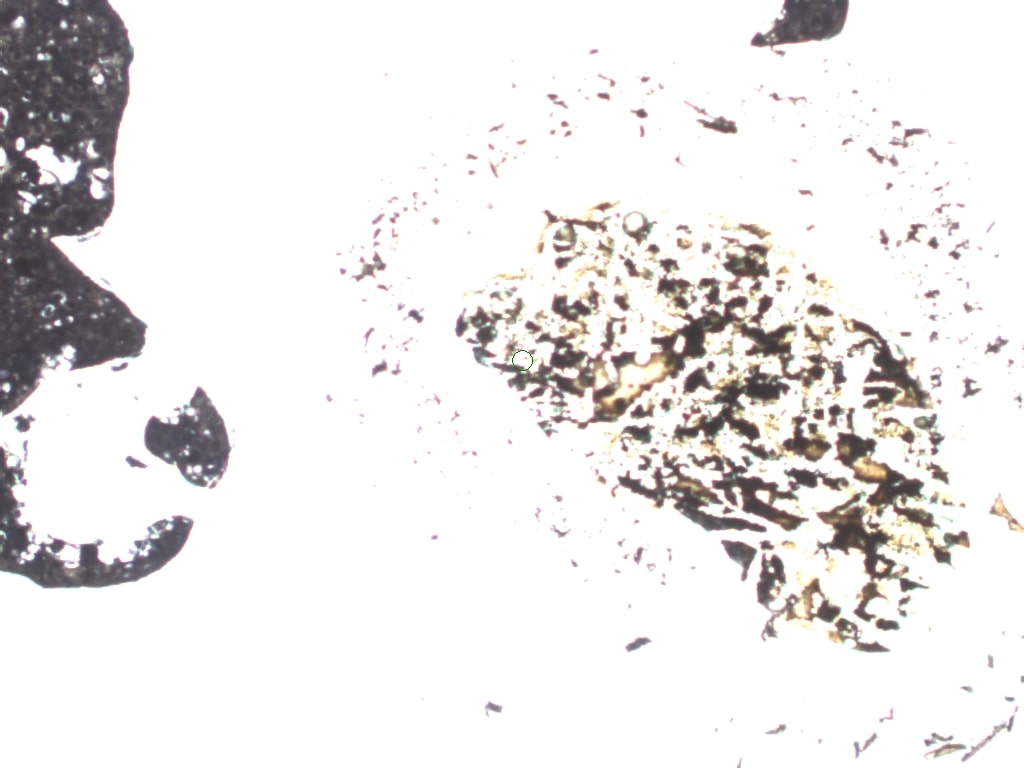

Supplement: Supplemental Material [file supp_gr.234807.118_Supplemental_File_4.zip › ABLATION/ABLATION FOR DCIS/DCIS3MAC3 ABLATION 10x .jpeg]

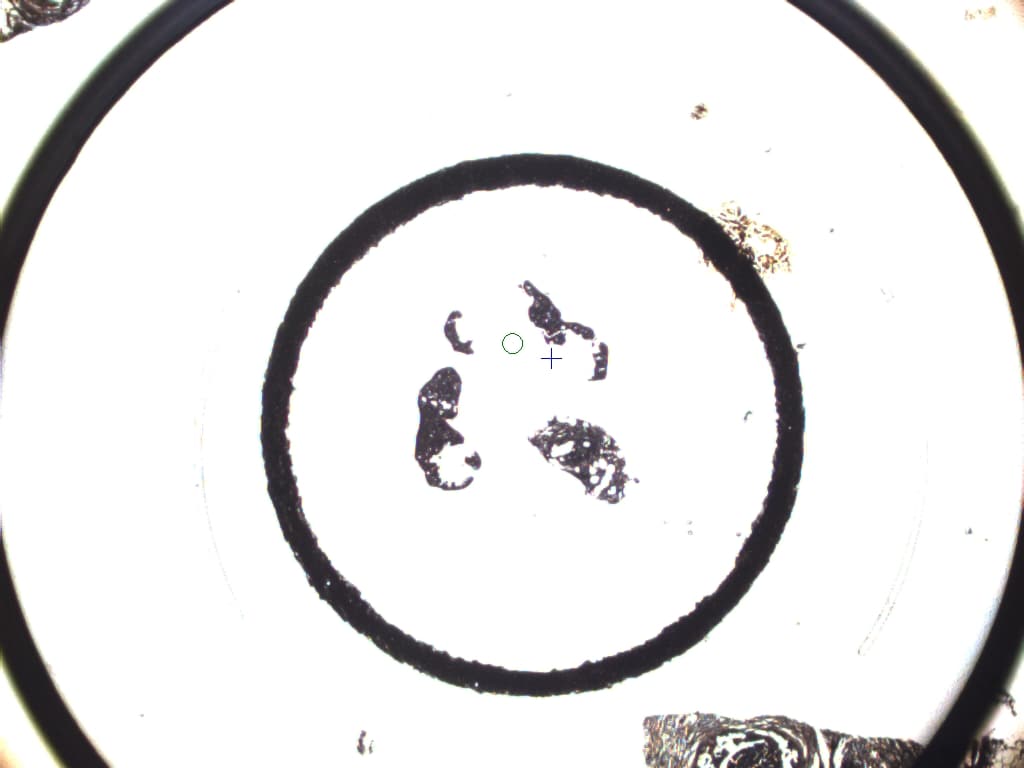

Supplement: Supplemental Material [file supp_gr.234807.118_Supplemental_File_4.zip › ABLATION/ABLATION FOR DCIS/DCIS3MAC3 BEFORE .jpeg]

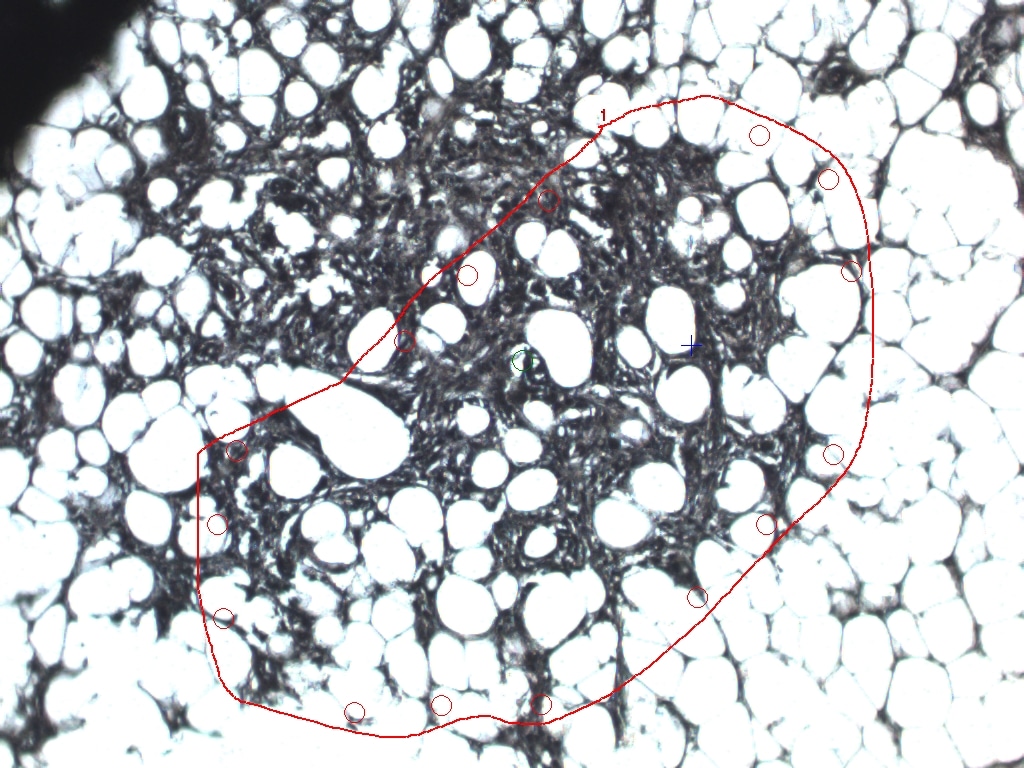

Supplement: Supplemental Material [file supp_gr.234807.118_Supplemental_File_4.zip › ABLATION/ABLATION FOR DCIS/MAC1 BEFORE.jpeg]

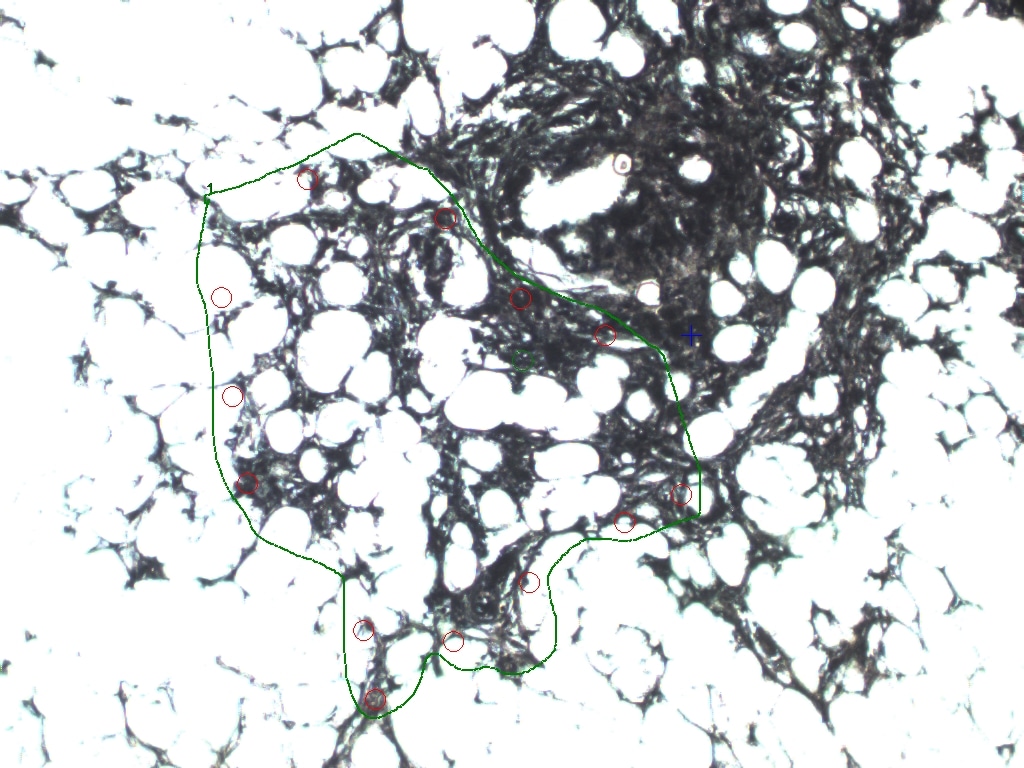

Supplement: Supplemental Material [file supp_gr.234807.118_Supplemental_File_4.zip › ABLATION/ABLATION FOR DCIS/MAC2 BEFORE.jpeg]

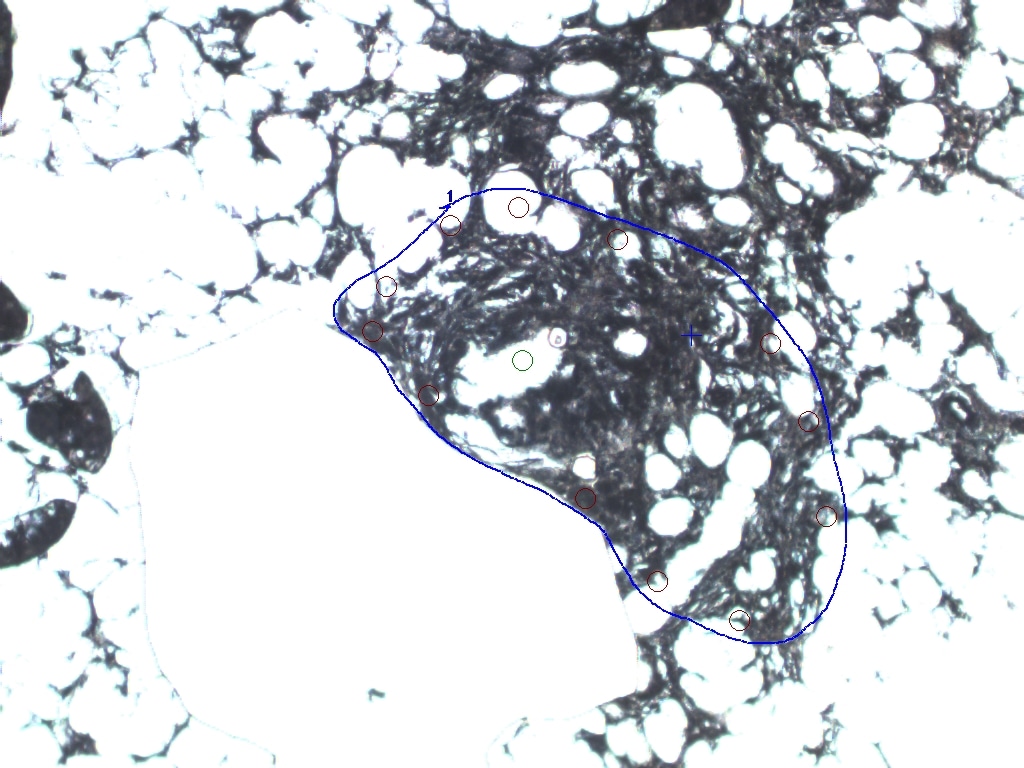

Supplement: Supplemental Material [file supp_gr.234807.118_Supplemental_File_4.zip › ABLATION/ABLATION FOR DCIS/MAC3 BEFORE.jpeg]

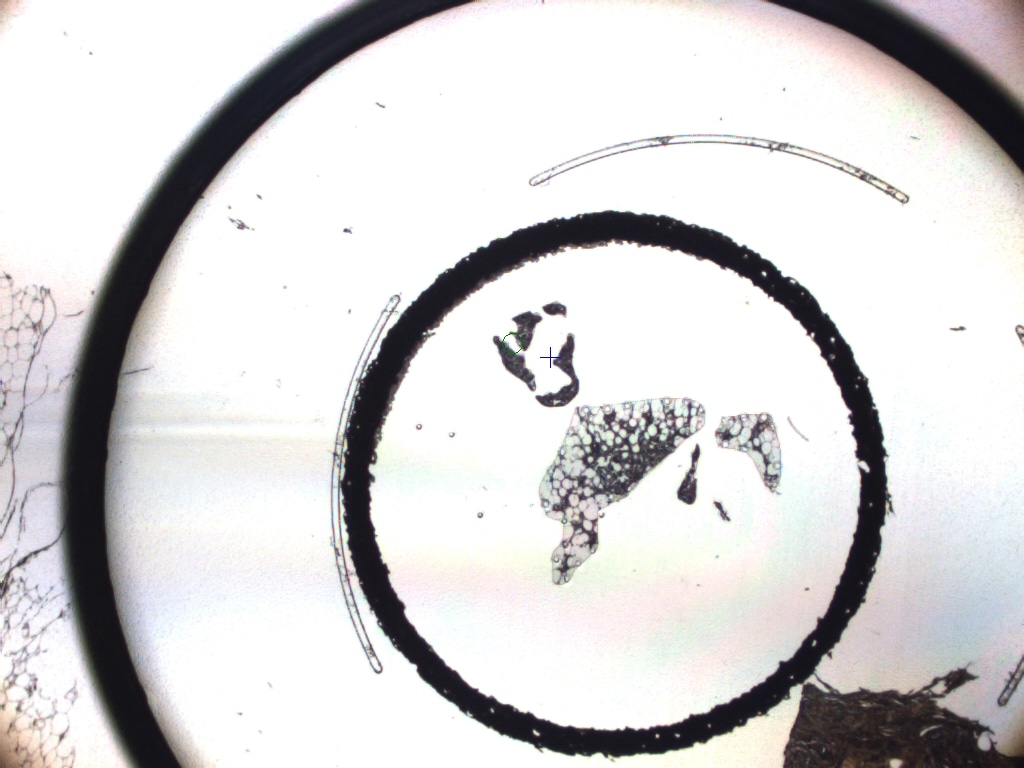

Supplement: Supplemental Material [file supp_gr.234807.118_Supplemental_File_4.zip › ABLATION/ABLATION FOR MAC/CAP1 BEFORE ABLATION.jpeg]

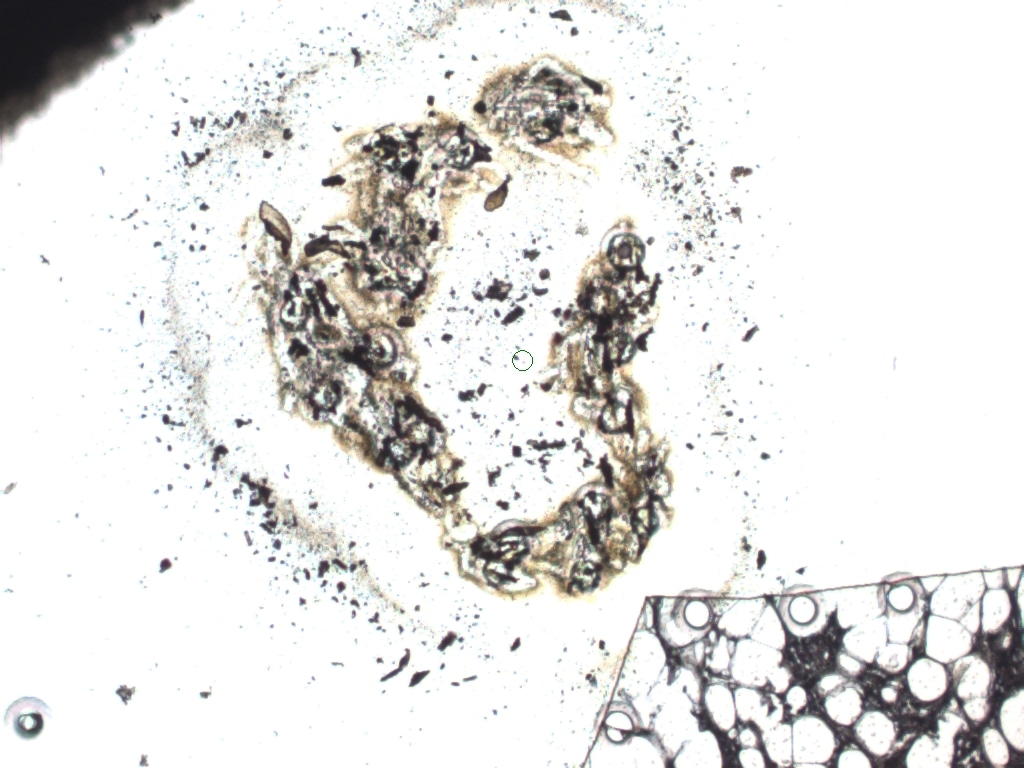

Supplement: Supplemental Material [file supp_gr.234807.118_Supplemental_File_4.zip › ABLATION/ABLATION FOR MAC/CAP1AFTERABLATION 10x.jpeg]

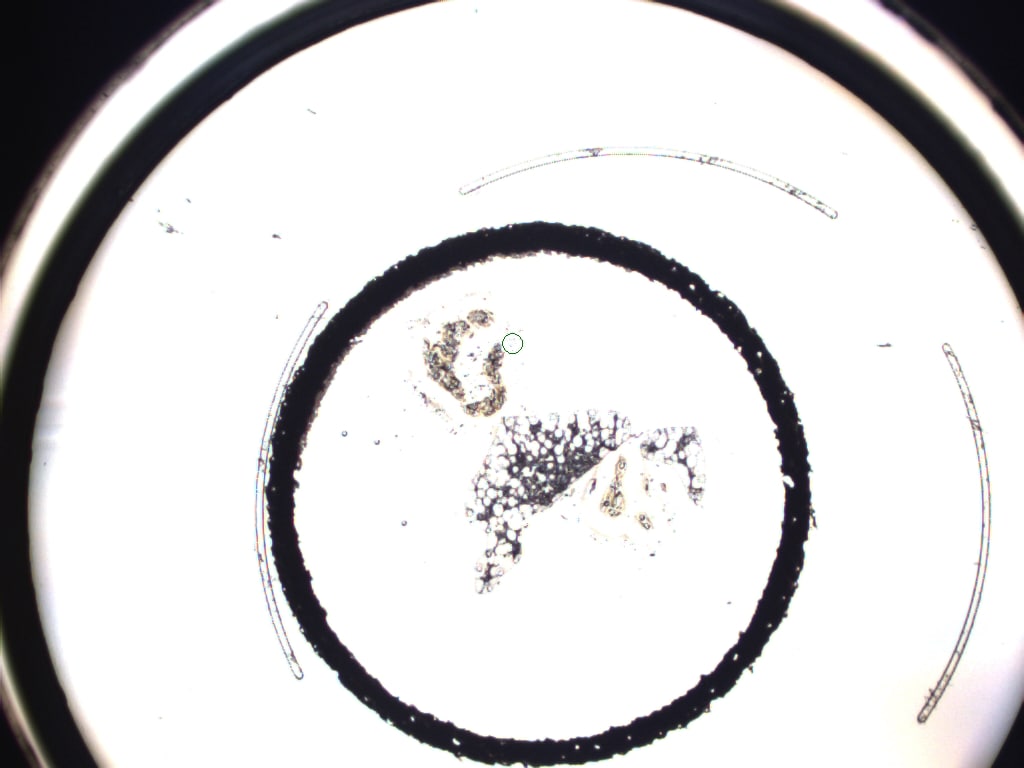

Supplement: Supplemental Material [file supp_gr.234807.118_Supplemental_File_4.zip › ABLATION/ABLATION FOR MAC/CAP1AFTERABLATION.jpeg]

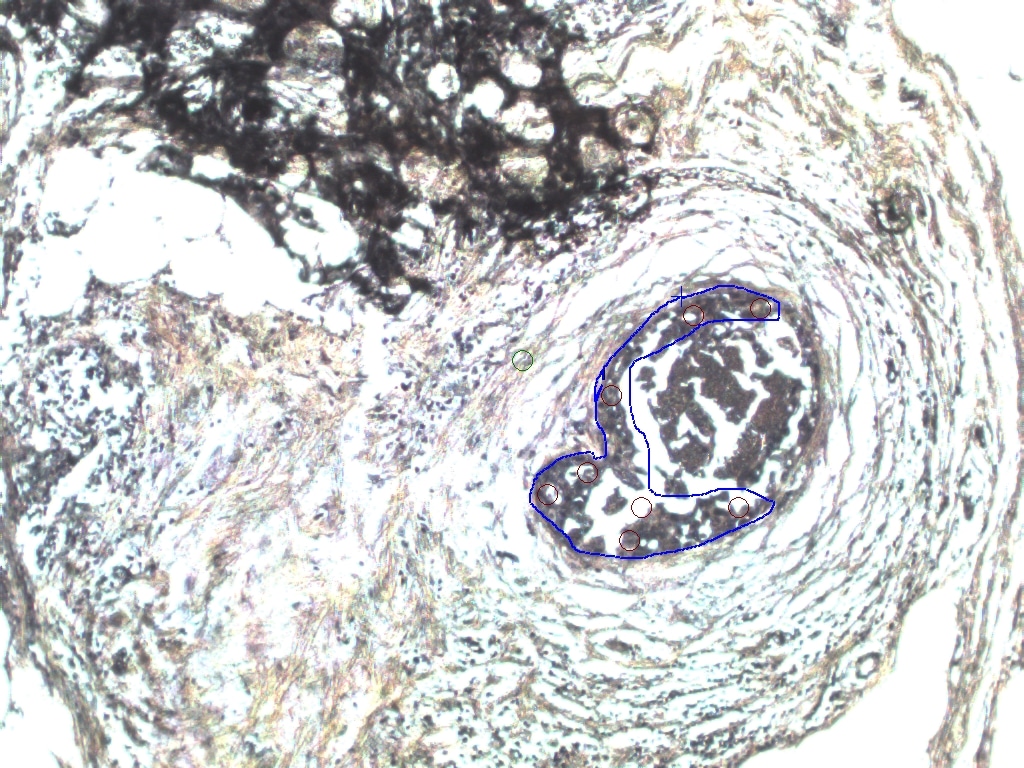

Supplement: Supplemental Material [file supp_gr.234807.118_Supplemental_File_4.zip › ABLATION/ABLATION FOR MAC/DCIS3-2 BEFORE 10x.jpeg]

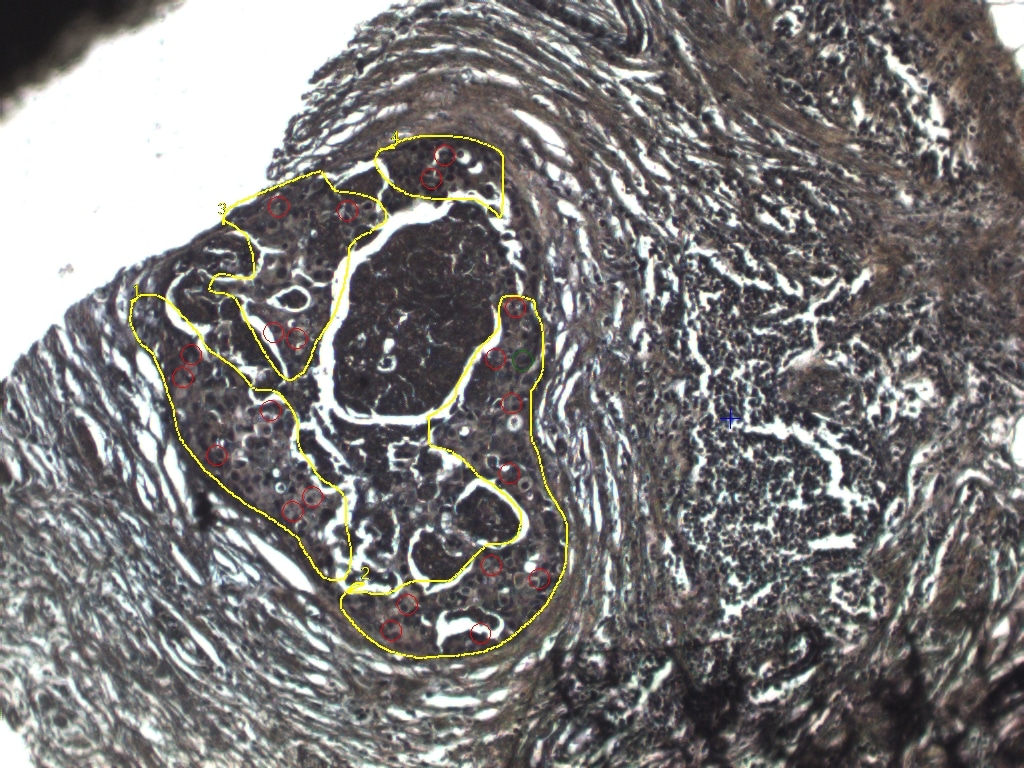

Supplement: Supplemental Material [file supp_gr.234807.118_Supplemental_File_4.zip › ABLATION/ABLATION FOR MAC/IDC1 BEFORE.jpeg]

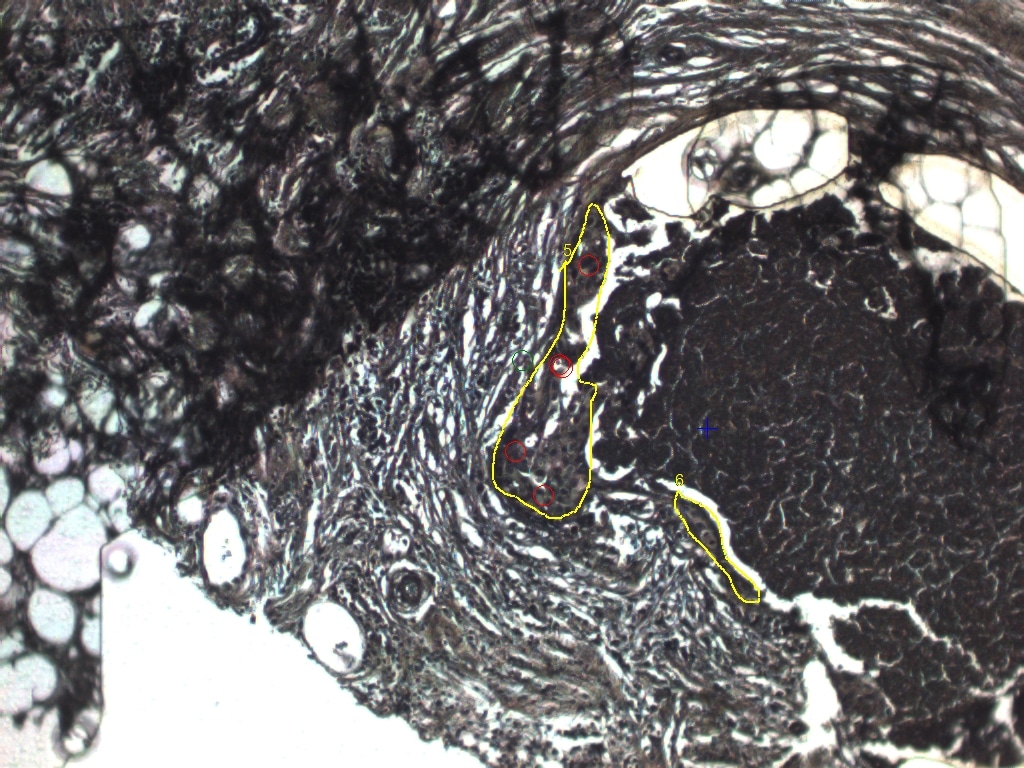

Supplement: Supplemental Material [file supp_gr.234807.118_Supplemental_File_4.zip › ABLATION/ABLATION FOR MAC/IDC1-2 BEFORE.jpeg]

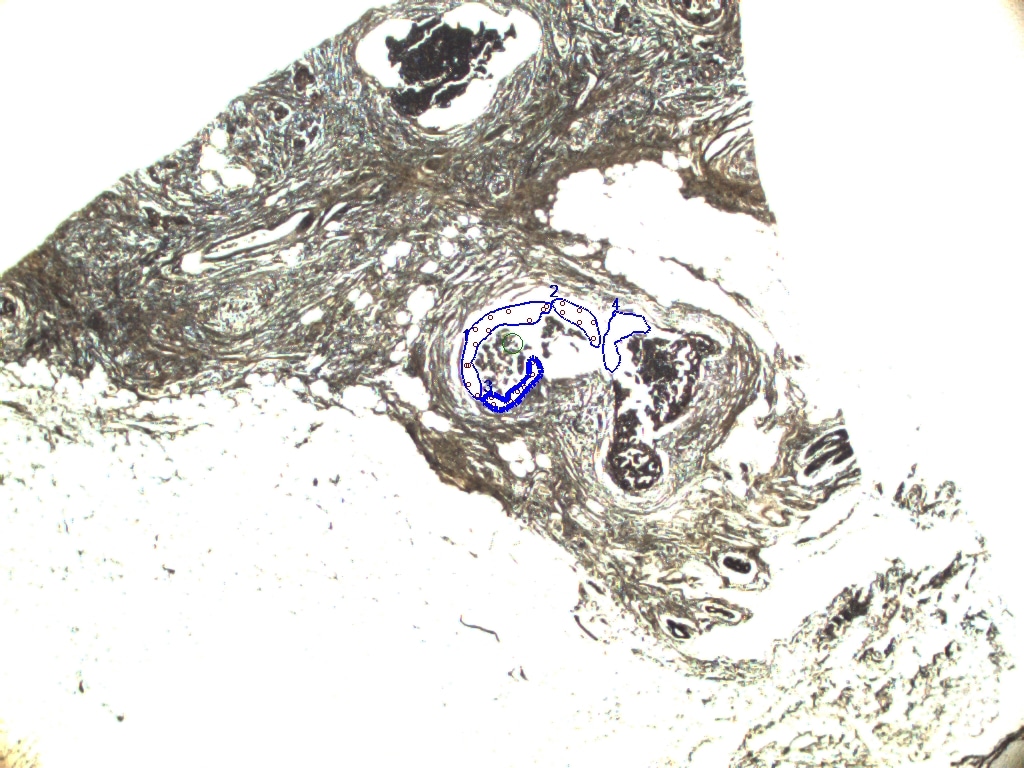

Supplement: Supplemental Material [file supp_gr.234807.118_Supplemental_File_4.zip › ABLATION/ABLATION FOR MAC/IDC1AFTER.jpeg]

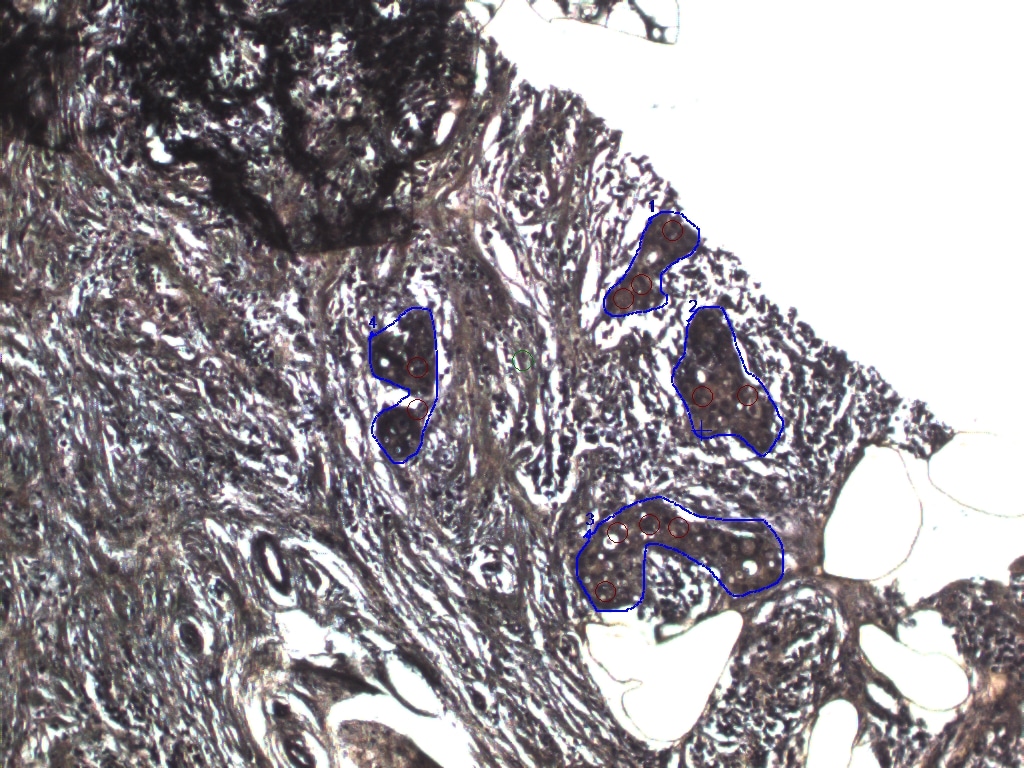

Supplement: Supplemental Material [file supp_gr.234807.118_Supplemental_File_4.zip › ABLATION/ABLATION FOR MAC/IDC2 BEFORE.jpeg]

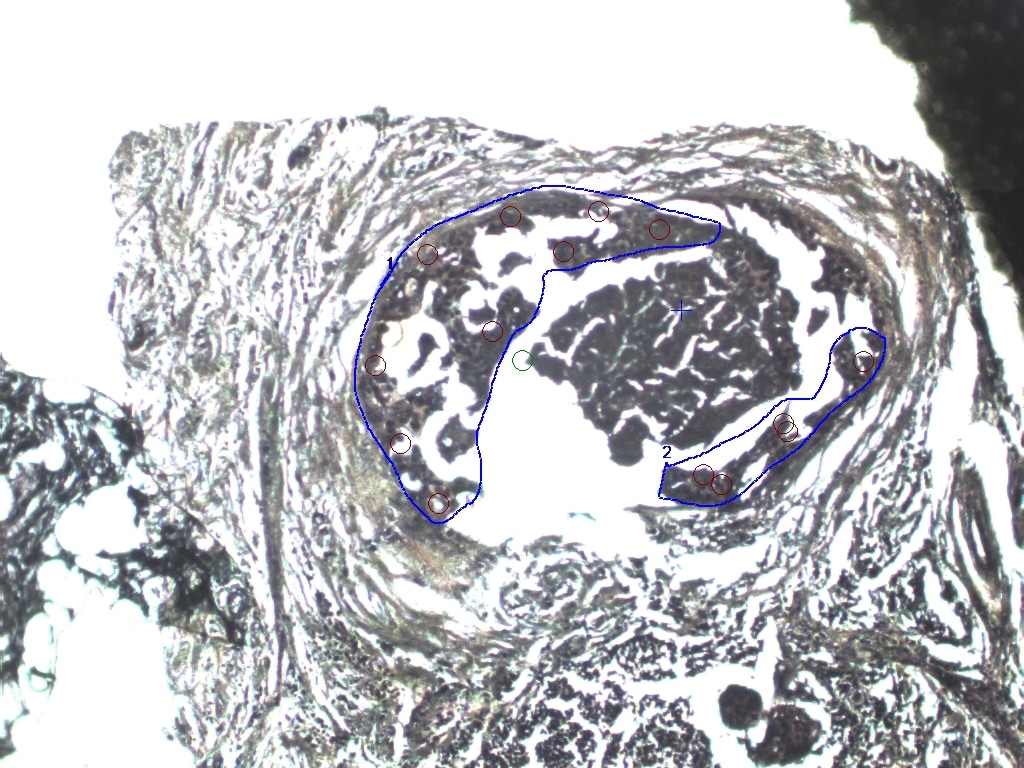

Supplement: Supplemental Material [file supp_gr.234807.118_Supplemental_File_4.zip › ABLATION/ABLATION FOR MAC/IDC2-2 BEFORE.jpeg]

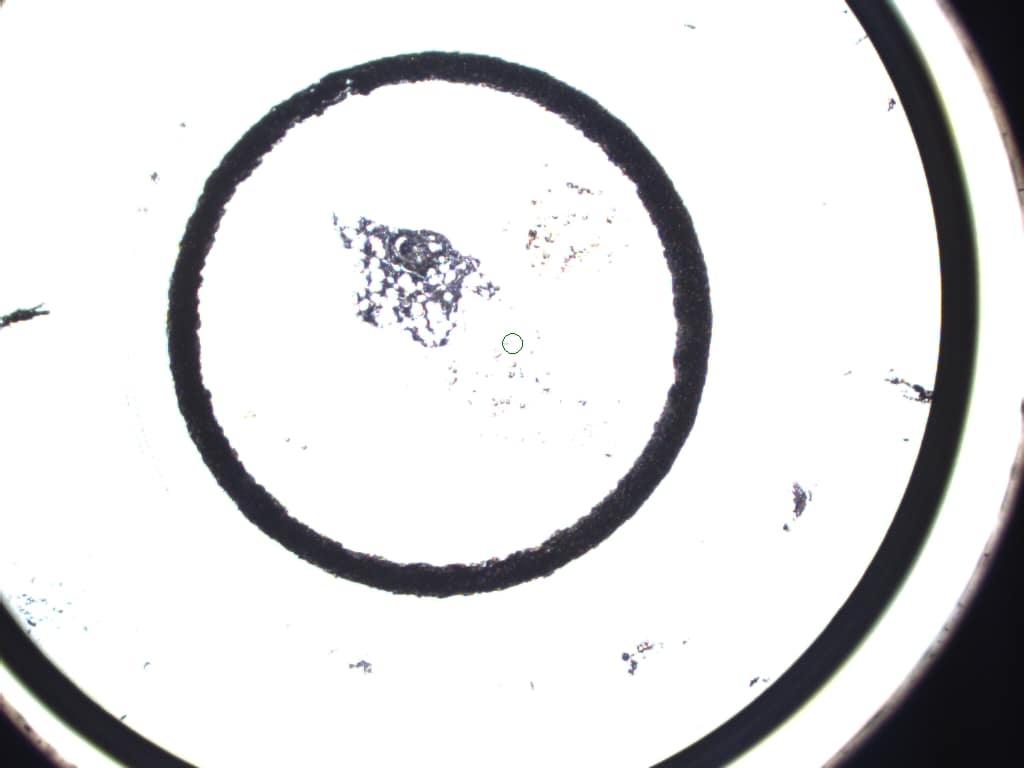

Supplement: Supplemental Material [file supp_gr.234807.118_Supplemental_File_4.zip › ABLATION/ABLATION FOR MAC/MAC2 AFTER ABLATION .jpeg]

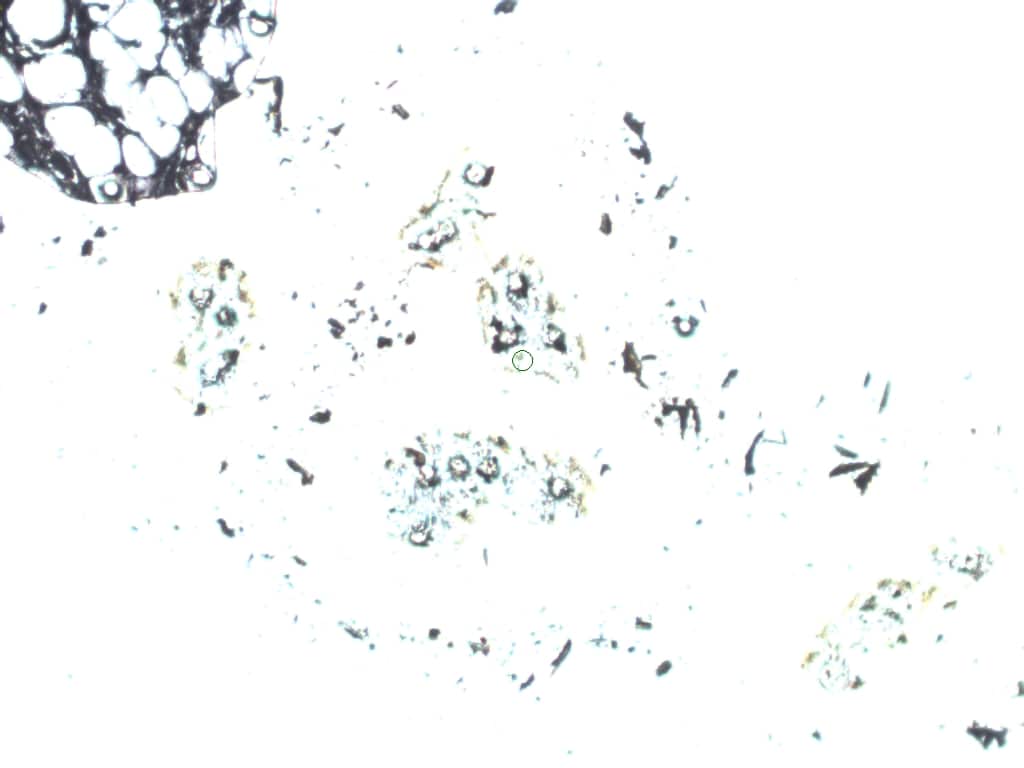

Supplement: Supplemental Material [file supp_gr.234807.118_Supplemental_File_4.zip › ABLATION/ABLATION FOR MAC/MAC2 AFTER ABLATION10x.jpeg]

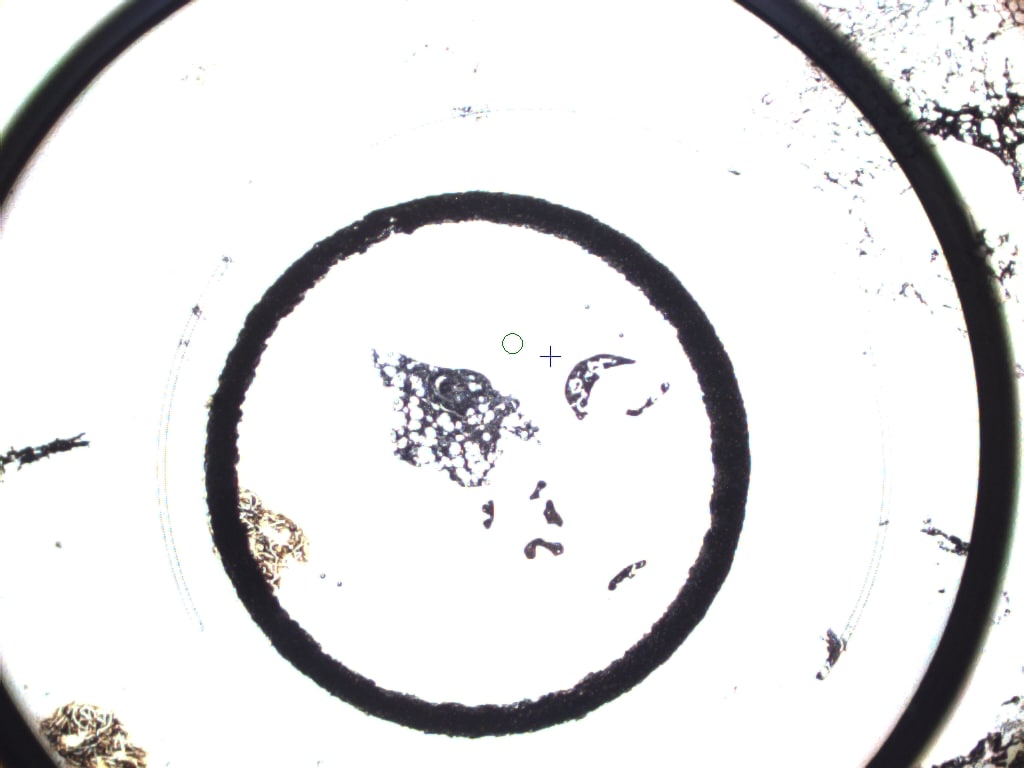

Supplement: Supplemental Material [file supp_gr.234807.118_Supplemental_File_4.zip › ABLATION/ABLATION FOR MAC/MAC2 BEFORE ABLATION.jpeg]

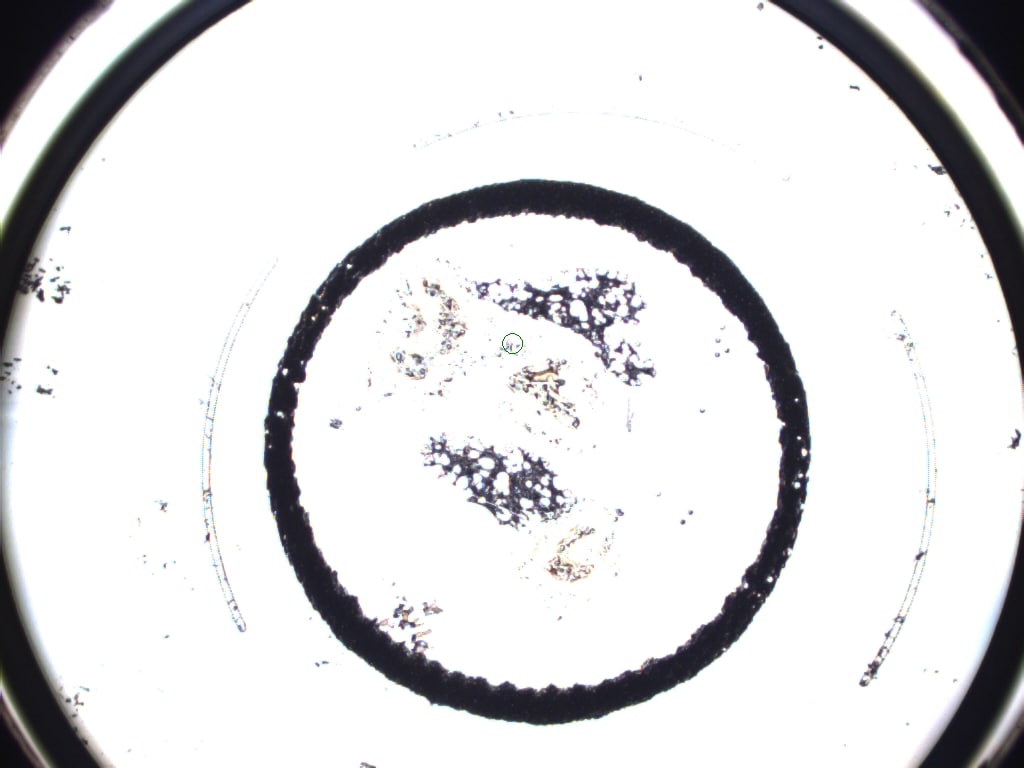

Supplement: Supplemental Material [file supp_gr.234807.118_Supplemental_File_4.zip › ABLATION/ABLATION FOR MAC/MAC3 AFTER ABLATION .jpeg]

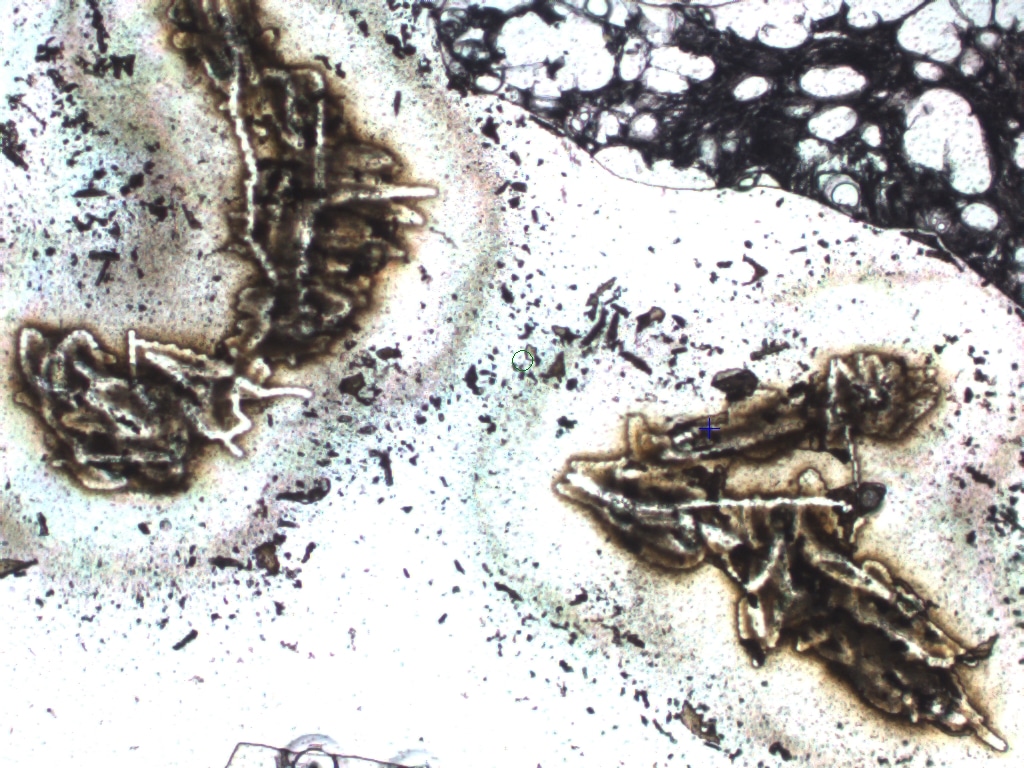

Supplement: Supplemental Material [file supp_gr.234807.118_Supplemental_File_4.zip › ABLATION/ABLATION FOR MAC/MAC3 AFTER ABLATION10x.jpeg]

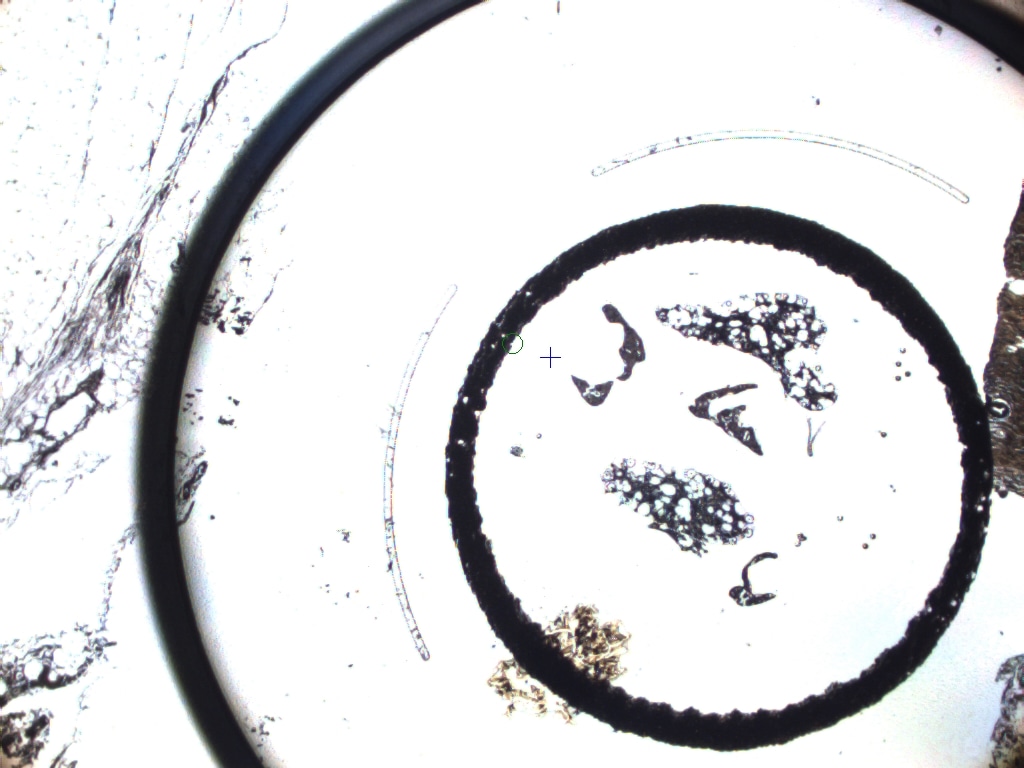

Supplement: Supplemental Material [file supp_gr.234807.118_Supplemental_File_4.zip › ABLATION/ABLATION FOR MAC/MAC3 BEFORE ABLATION.jpeg]

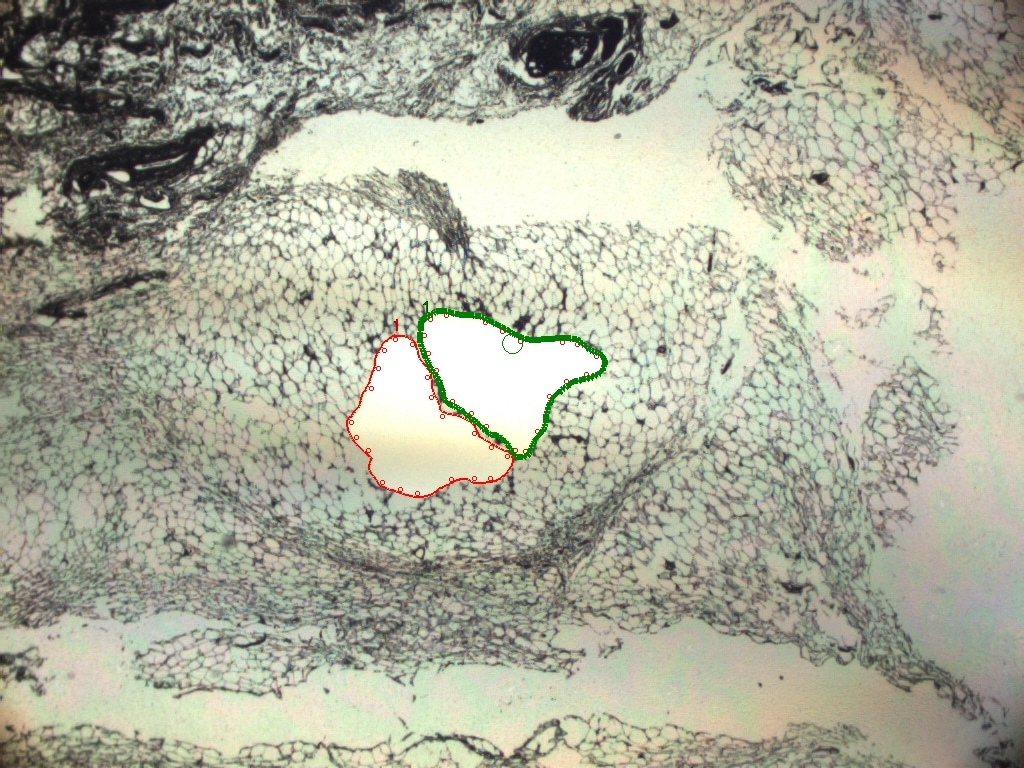

Supplement: Supplemental Material [file supp_gr.234807.118_Supplemental_File_4.zip › ABLATION/ABLATION FOR MAC/MACROPHAGE 1 AFTER.jpeg]

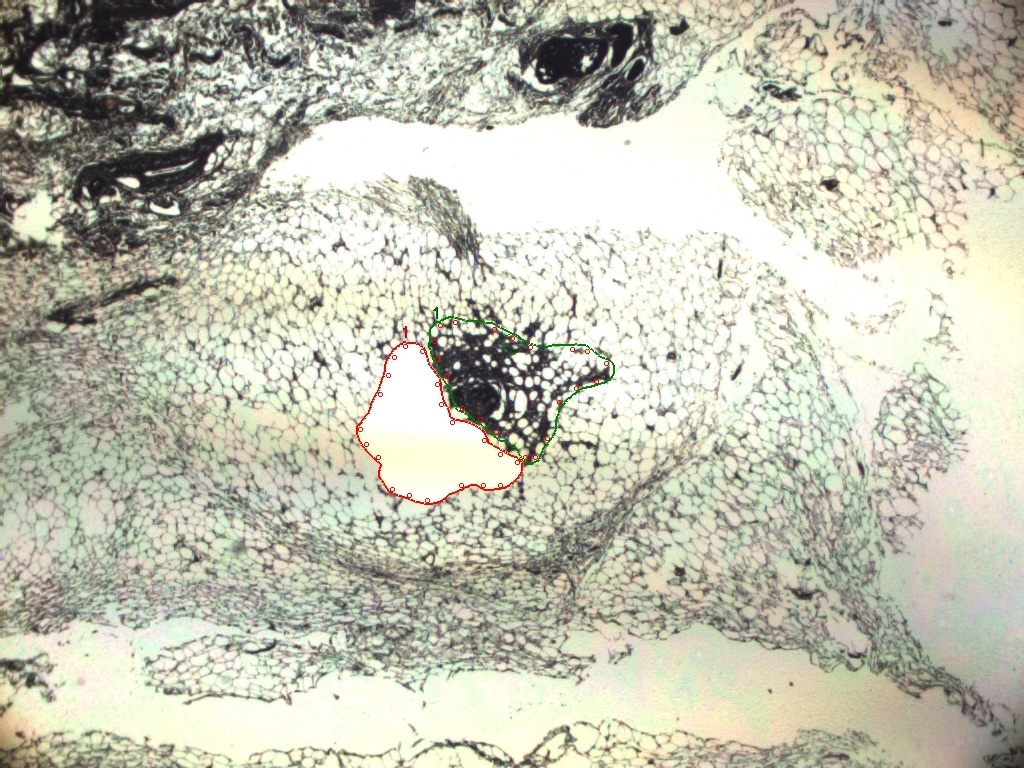

Supplement: Supplemental Material [file supp_gr.234807.118_Supplemental_File_4.zip › ABLATION/ABLATION FOR MAC/MACROPHAGE 1 BEFORE.jpeg]

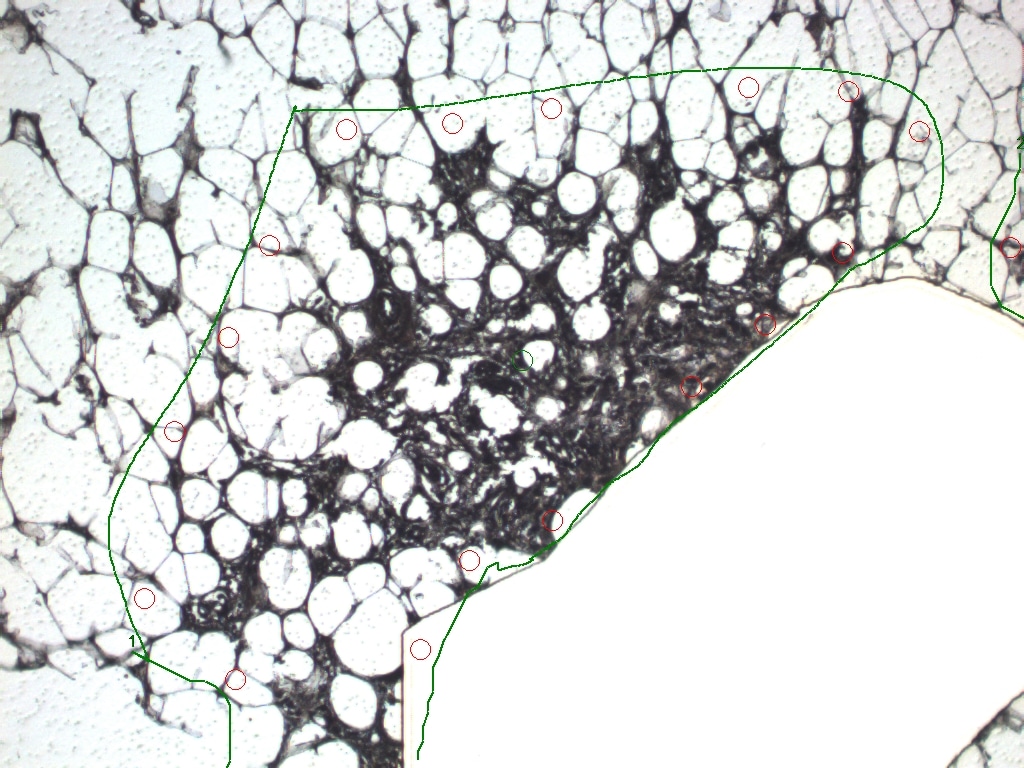

Supplement: Supplemental Material [file supp_gr.234807.118_Supplemental_File_4.zip › ABLATION/ABLATION FOR MAC/MACROPHAGE1 BEFORE 10x.jpeg]

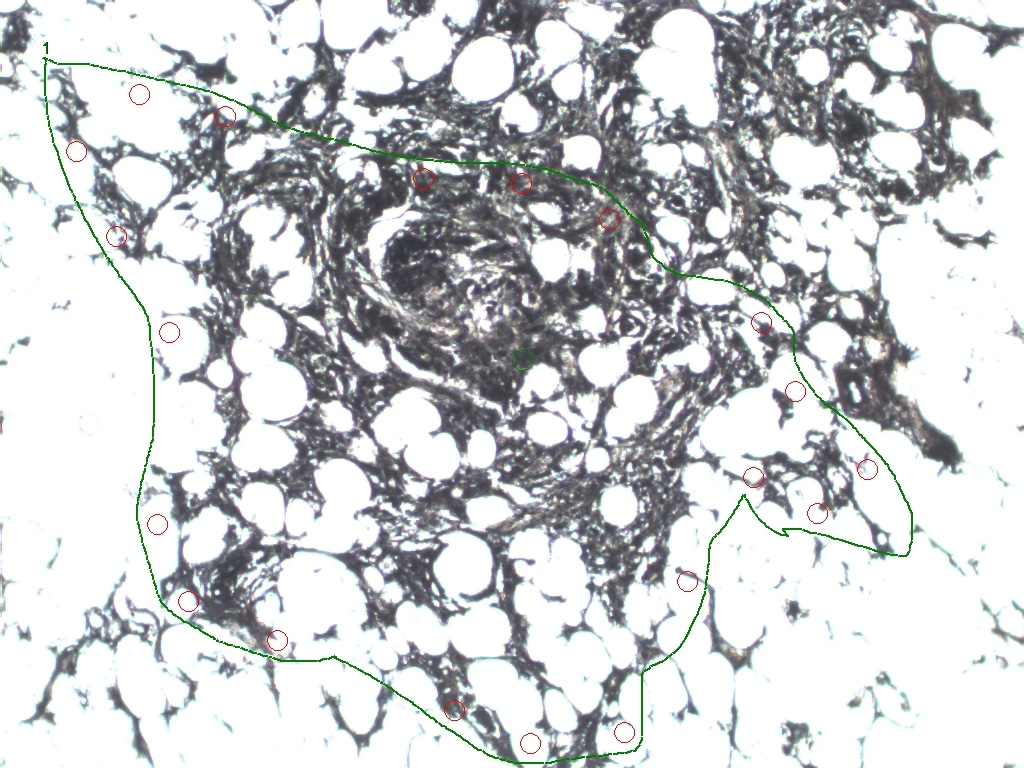

Supplement: Supplemental Material [file supp_gr.234807.118_Supplemental_File_4.zip › ABLATION/ABLATION FOR MAC/MACROPHAGE2 BEFORE 10x.jpeg]

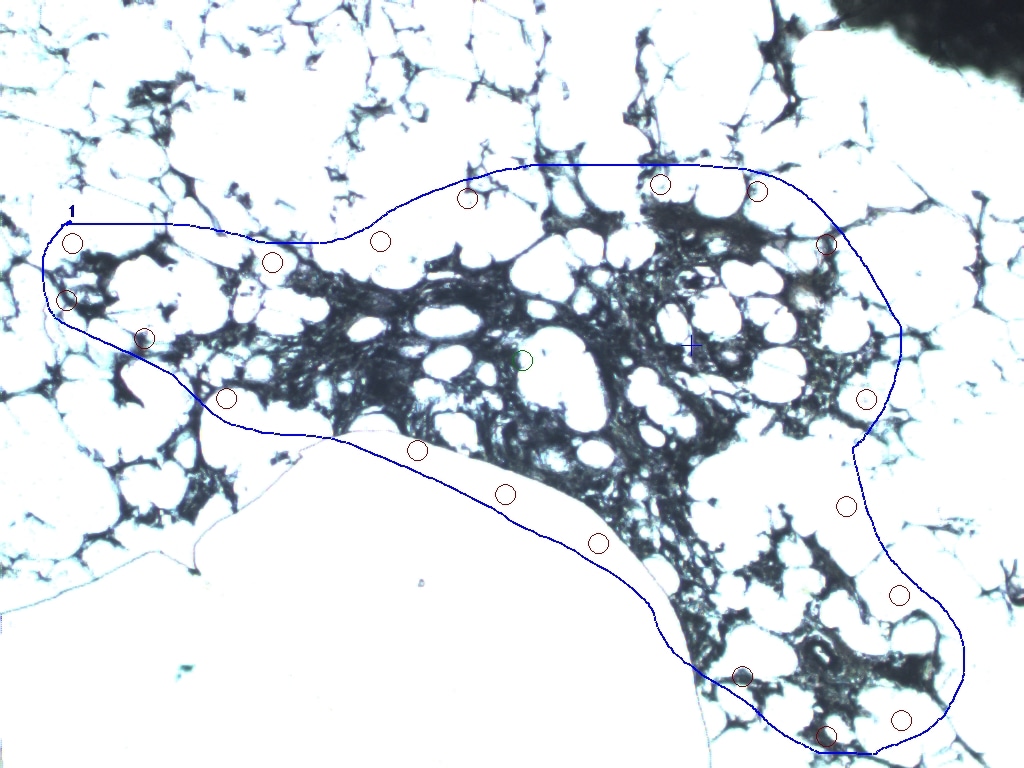

Supplement: Supplemental Material [file supp_gr.234807.118_Supplemental_File_4.zip › ABLATION/ABLATION FOR MAC/MACROPHAGE3 BEFORE 10x.jpeg]

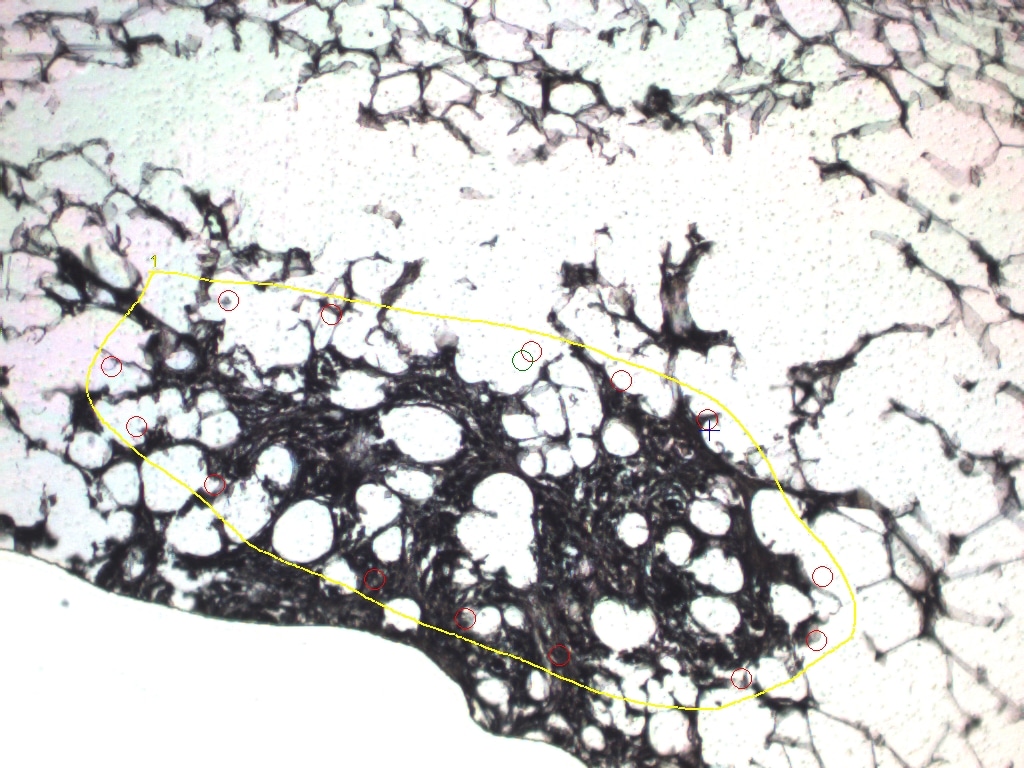

Supplement: Supplemental Material [file supp_gr.234807.118_Supplemental_File_4.zip › ABLATION/ABLATION FOR MAC/MACROPHAGE3-2 BEFORE 10x.jpeg]

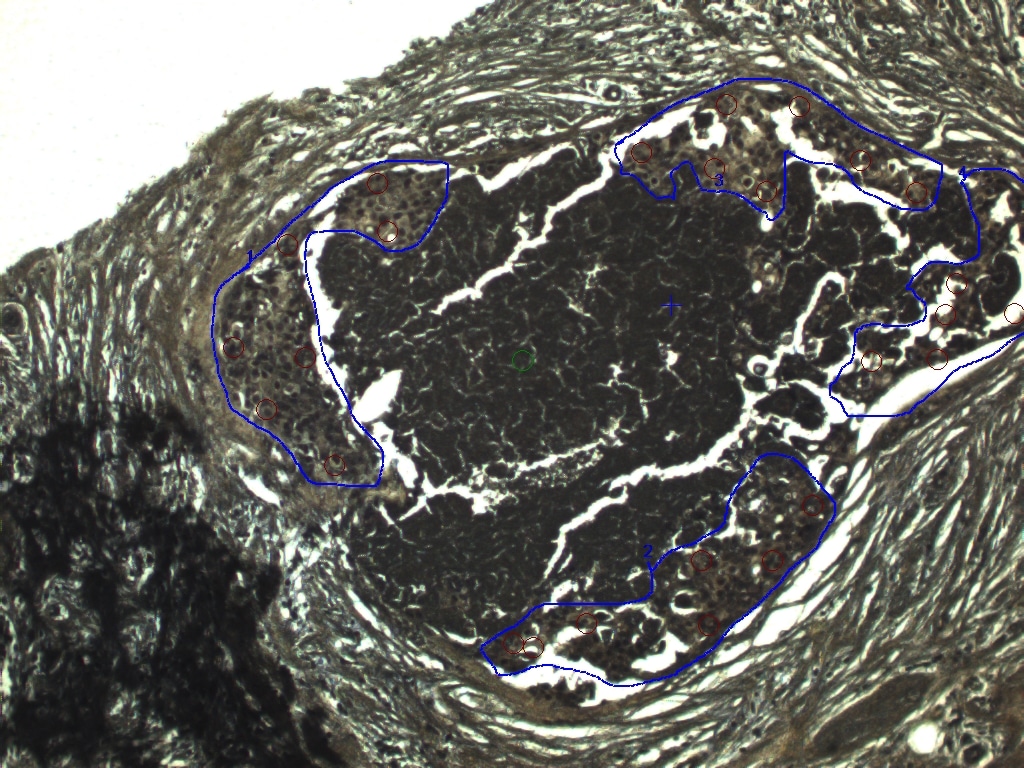

Supplement: Supplemental Material [file supp_gr.234807.118_Supplemental_File_4.zip › ABLATION/NO ABLATION/DCIS 1 BEFORE.jpeg]

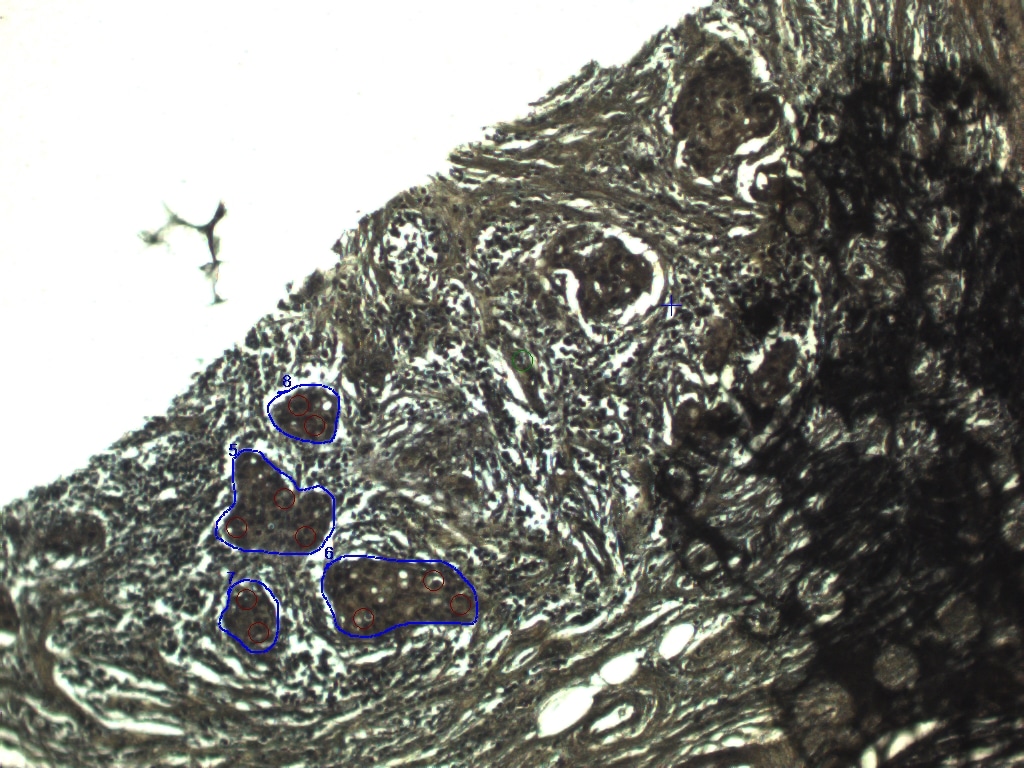

Supplement: Supplemental Material [file supp_gr.234807.118_Supplemental_File_4.zip › ABLATION/NO ABLATION/DCIS 1 BEFORE2.jpeg]

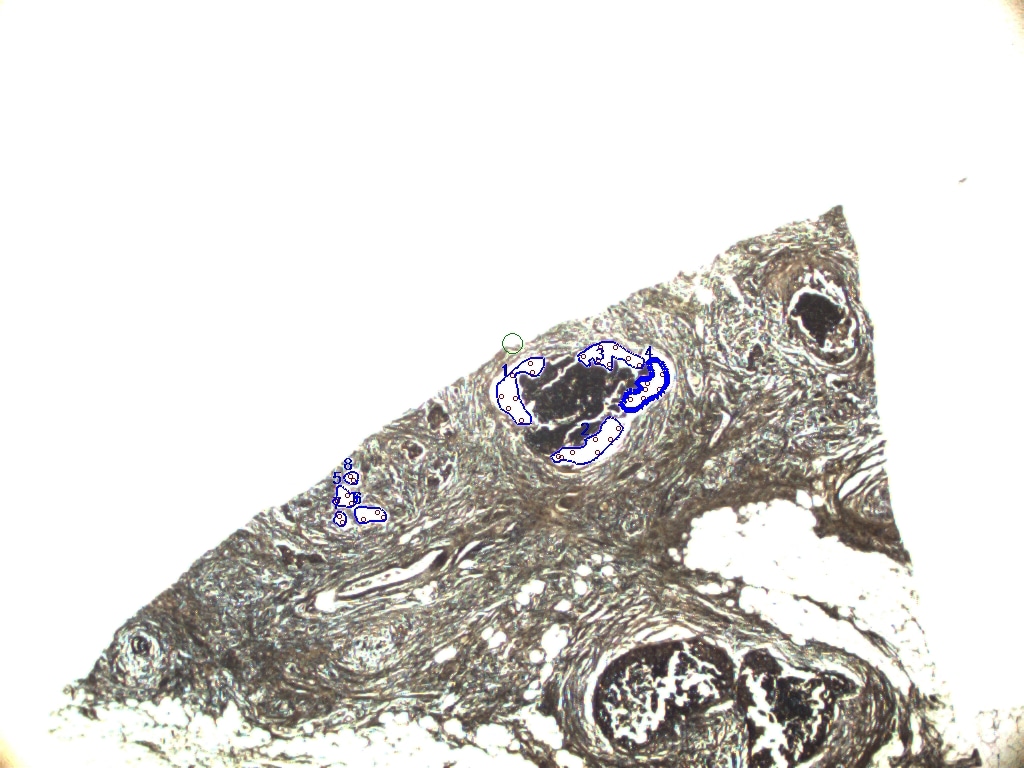

Supplement: Supplemental Material [file supp_gr.234807.118_Supplemental_File_4.zip › ABLATION/NO ABLATION/DCIS 1AFTER.jpeg]

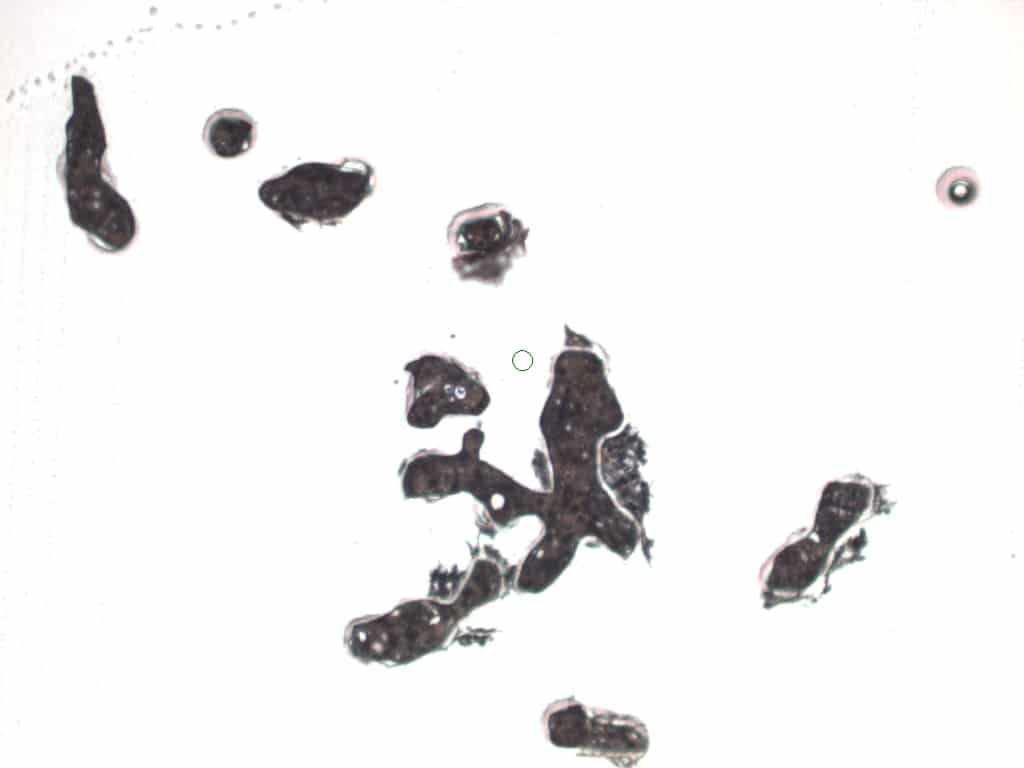

Supplement: Supplemental Material [file supp_gr.234807.118_Supplemental_File_4.zip › ABLATION/NO ABLATION/DCIS-7 .jpeg]

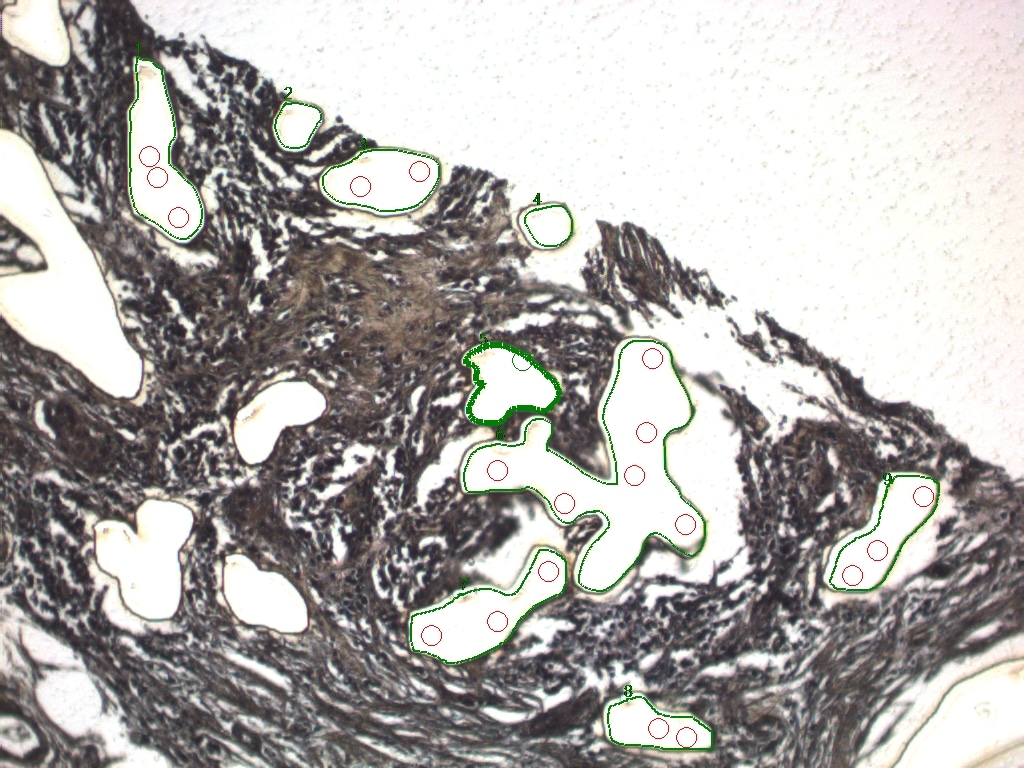

Supplement: Supplemental Material [file supp_gr.234807.118_Supplemental_File_4.zip › ABLATION/NO ABLATION/DCIS-7 AFTER.jpeg]

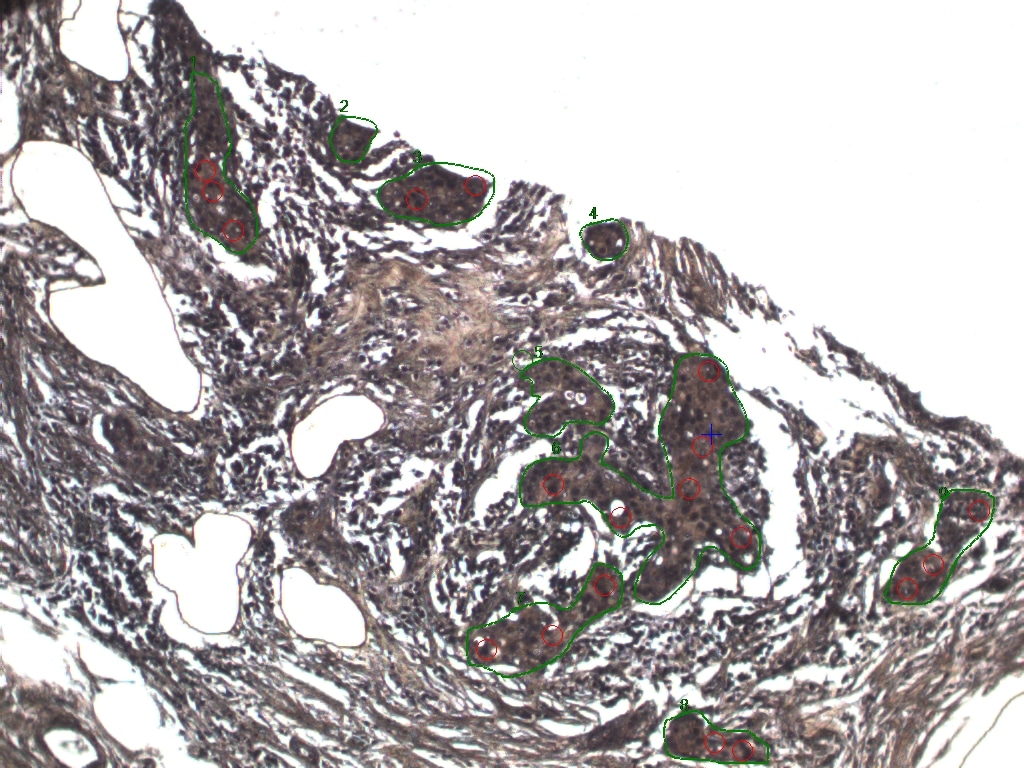

Supplement: Supplemental Material [file supp_gr.234807.118_Supplemental_File_4.zip › ABLATION/NO ABLATION/DCIS-7 BEFORE .jpeg]

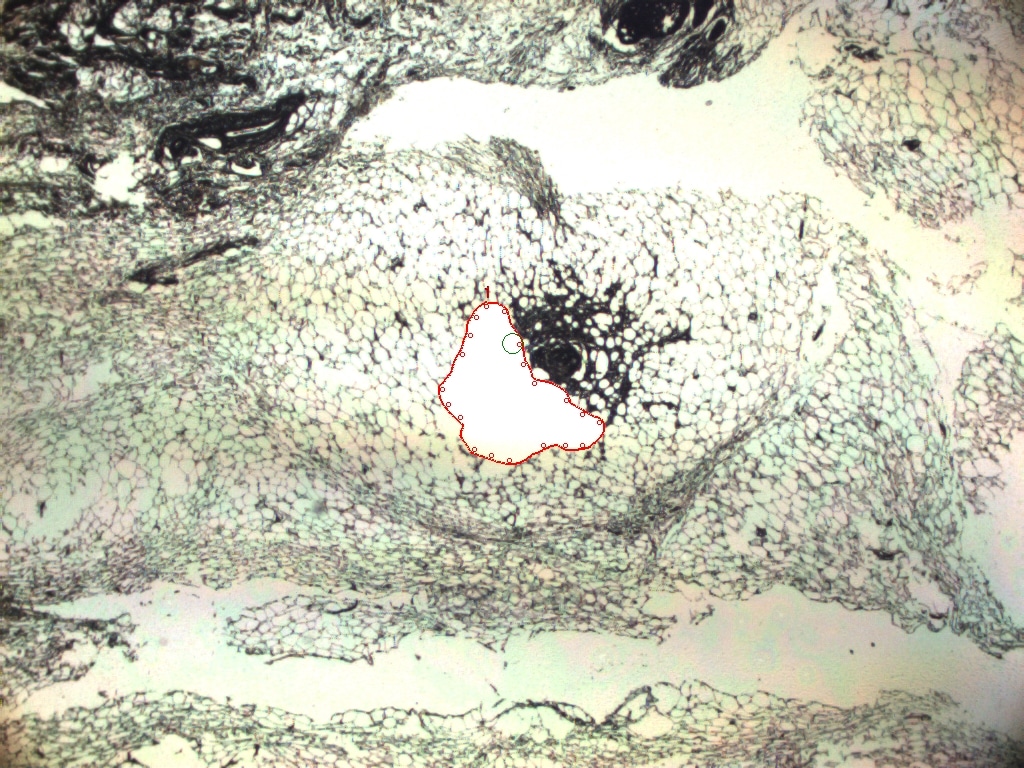

Supplement: Supplemental Material [file supp_gr.234807.118_Supplemental_File_4.zip › ABLATION/NO ABLATION/MACROPHAGE 1 AFTER.jpeg]

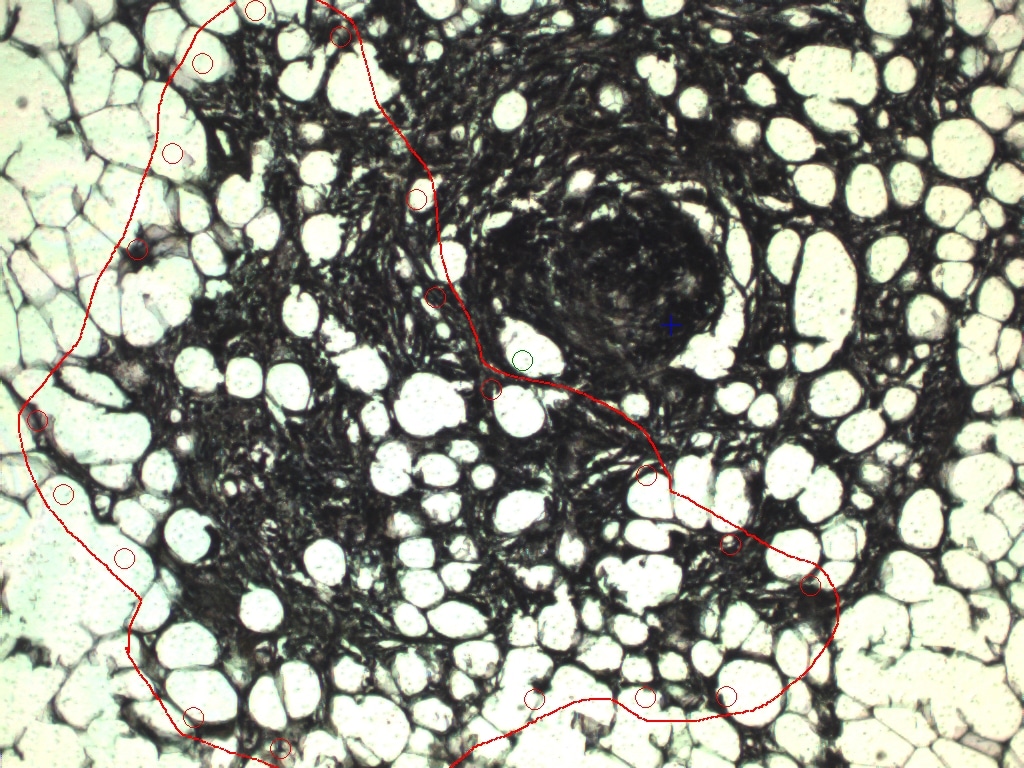

Supplement: Supplemental Material [file supp_gr.234807.118_Supplemental_File_4.zip › ABLATION/NO ABLATION/MACROPHAGE 1 BEFORE 10x.jpeg]

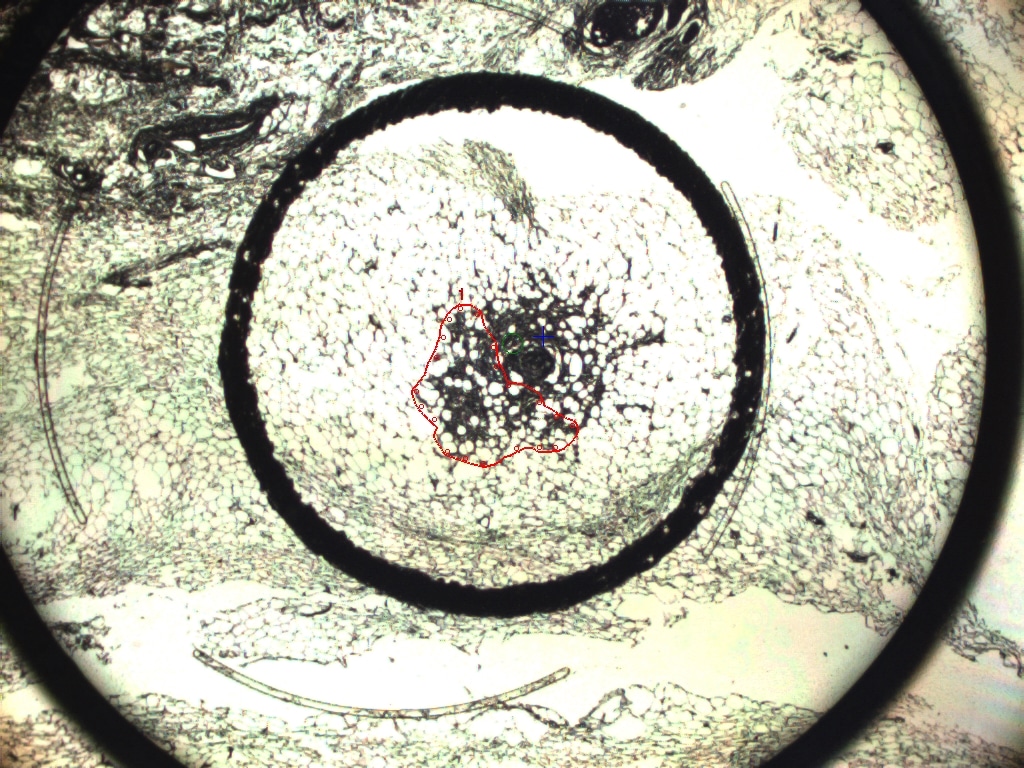

Supplement: Supplemental Material [file supp_gr.234807.118_Supplemental_File_4.zip › ABLATION/NO ABLATION/MACROPHAGE 1 BEFORE.jpeg]

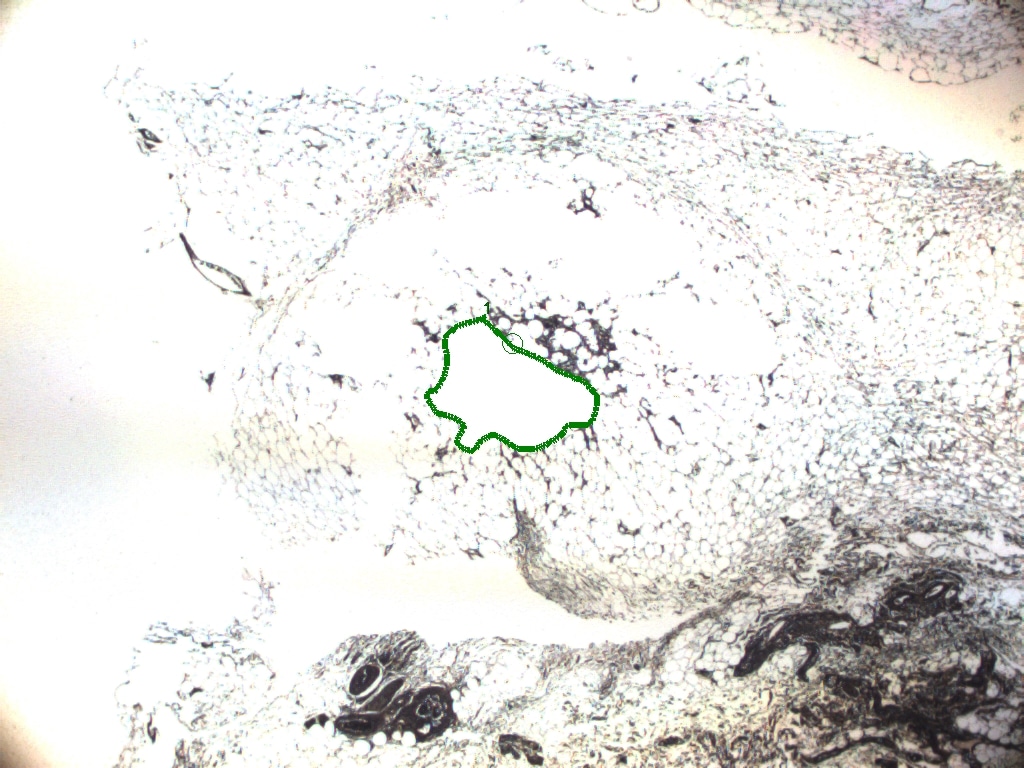

Supplement: Supplemental Material [file supp_gr.234807.118_Supplemental_File_4.zip › ABLATION/NO ABLATION/MACROPHAGE 2 AFTER.jpeg]

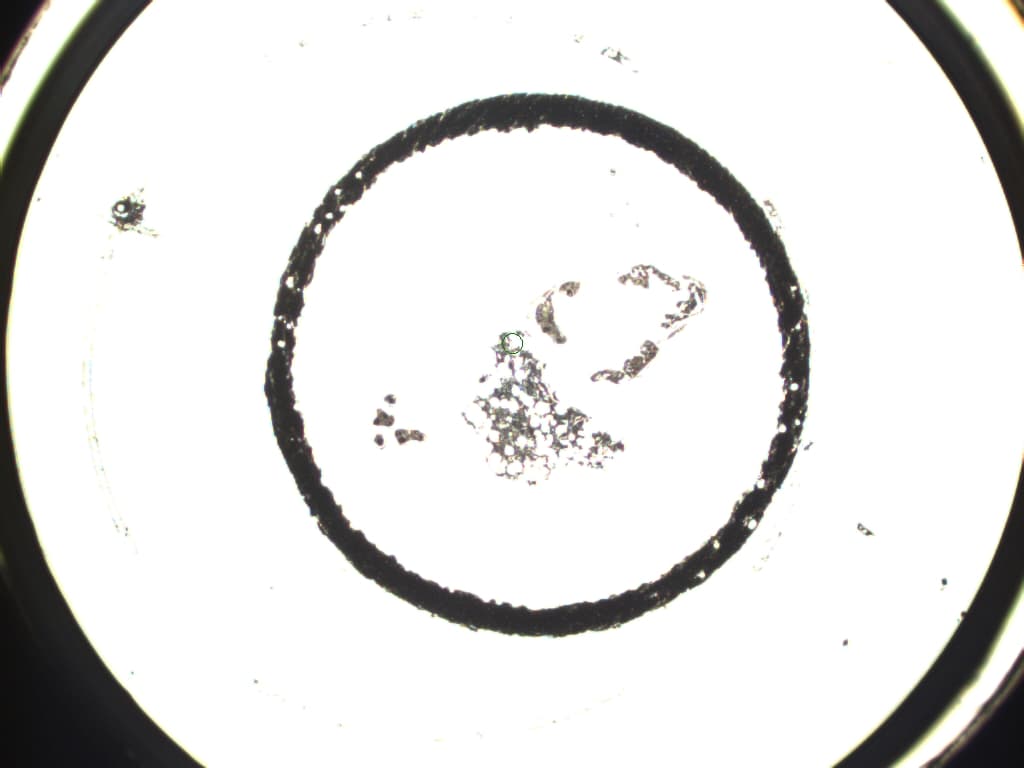

Supplement: Supplemental Material [file supp_gr.234807.118_Supplemental_File_4.zip › ABLATION/NO ABLATION/NO BLATION CAP1.jpeg]

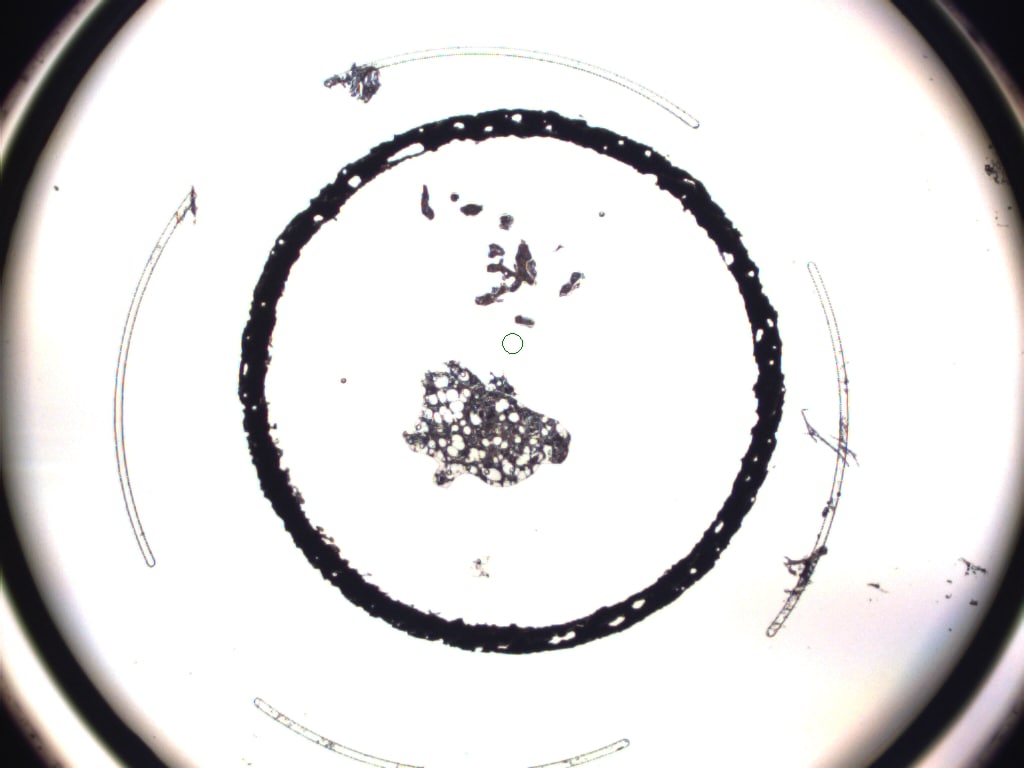

Supplement: Supplemental Material [file supp_gr.234807.118_Supplemental_File_4.zip › ABLATION/NO ABLATION/NO BLATION CAP2 DCIS7+MAC.jpeg]

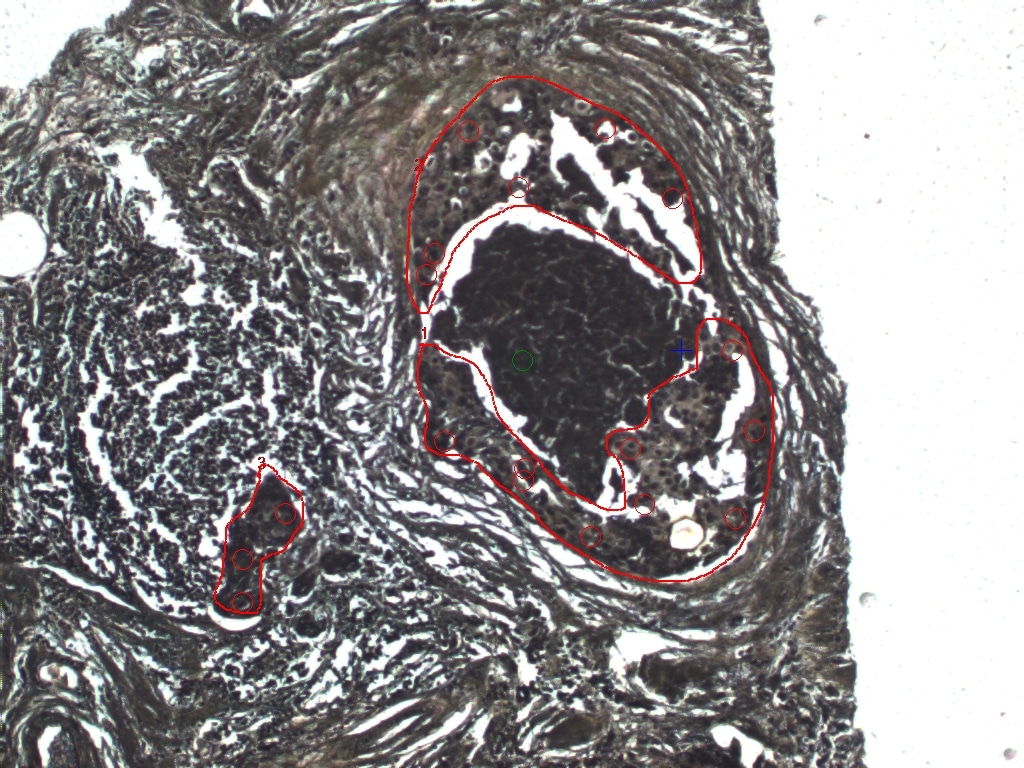

Supplement: Supplemental Material [file supp_gr.234807.118_Supplemental_File_4.zip › BULK/DCIS/DCIS-1 BEFORE.jpeg]

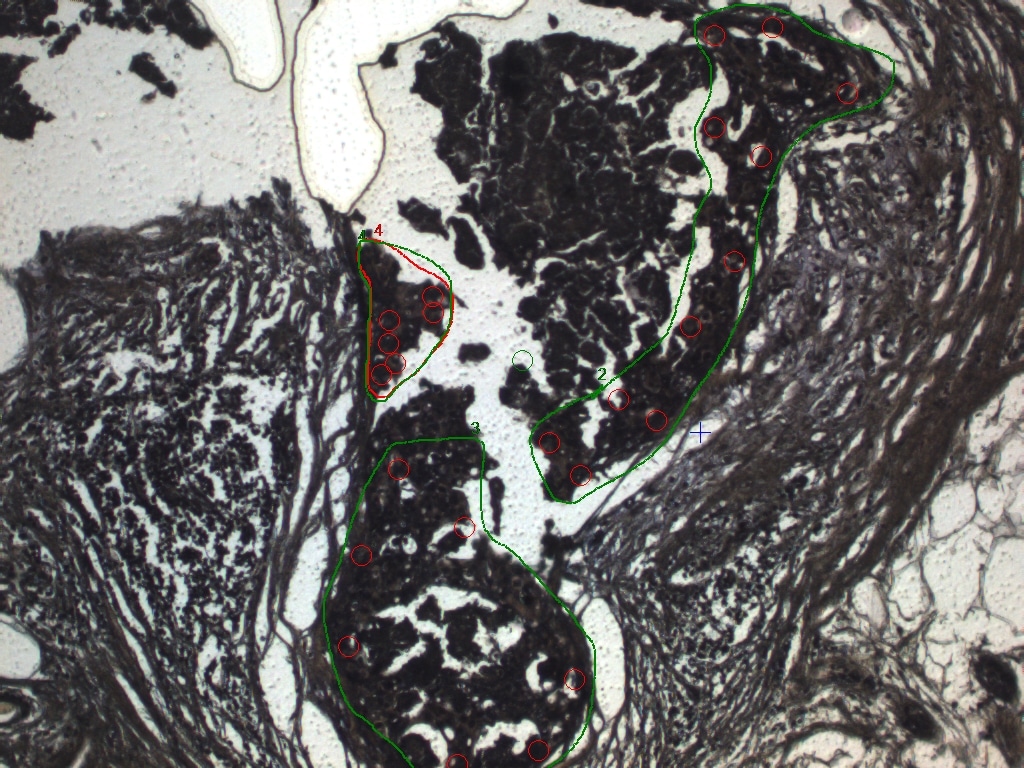

Supplement: Supplemental Material [file supp_gr.234807.118_Supplemental_File_4.zip › BULK/DCIS/DCIS-1-2 BEFORE.jpeg]

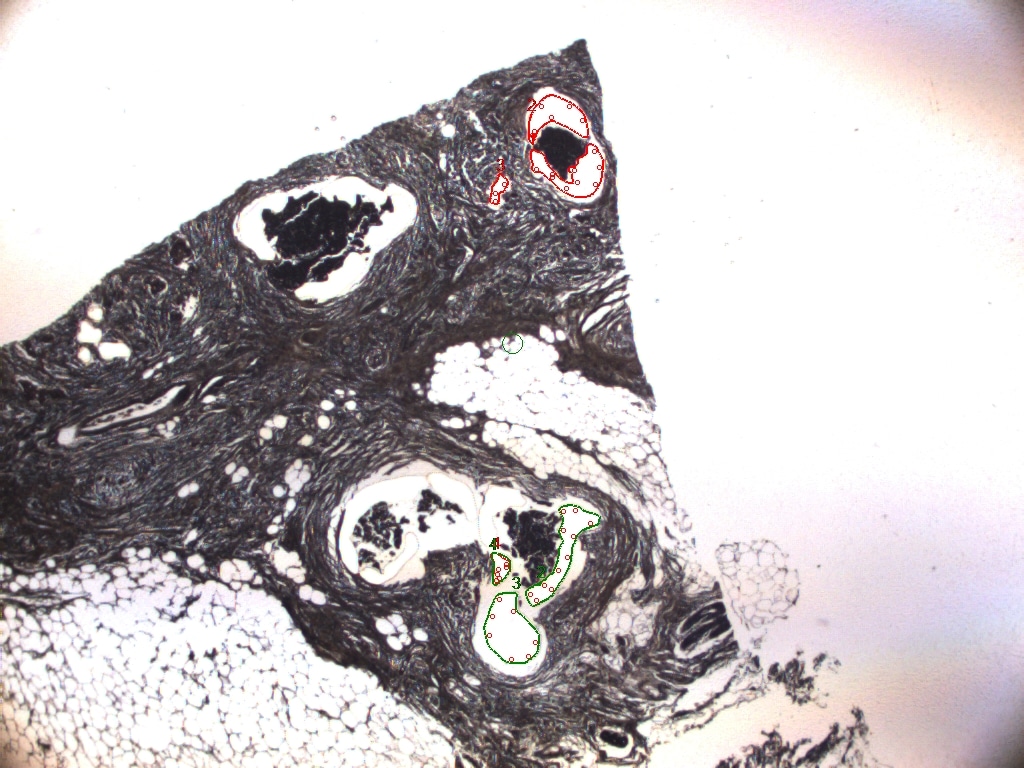

Supplement: Supplemental Material [file supp_gr.234807.118_Supplemental_File_4.zip › BULK/DCIS/DCIS-1-2AFTER.jpeg]

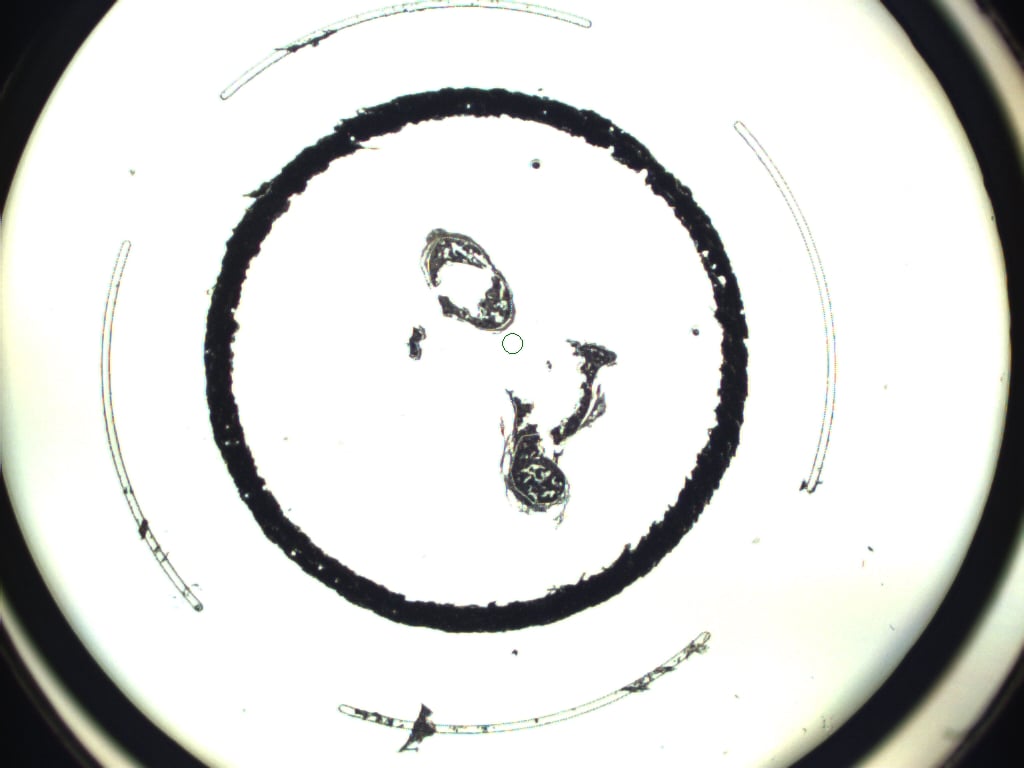

Supplement: Supplemental Material [file supp_gr.234807.118_Supplemental_File_4.zip › BULK/DCIS/DCIS-1.jpeg]

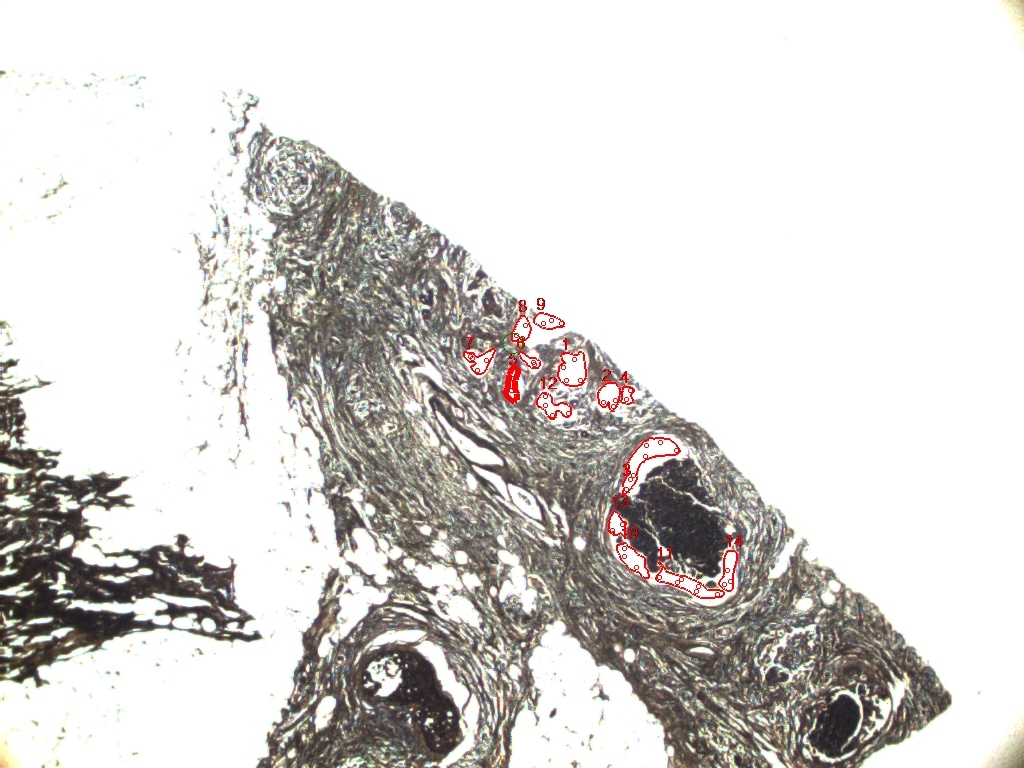

Supplement: Supplemental Material [file supp_gr.234807.118_Supplemental_File_4.zip › BULK/DCIS/DCIS-2 AFTER2X.jpeg]

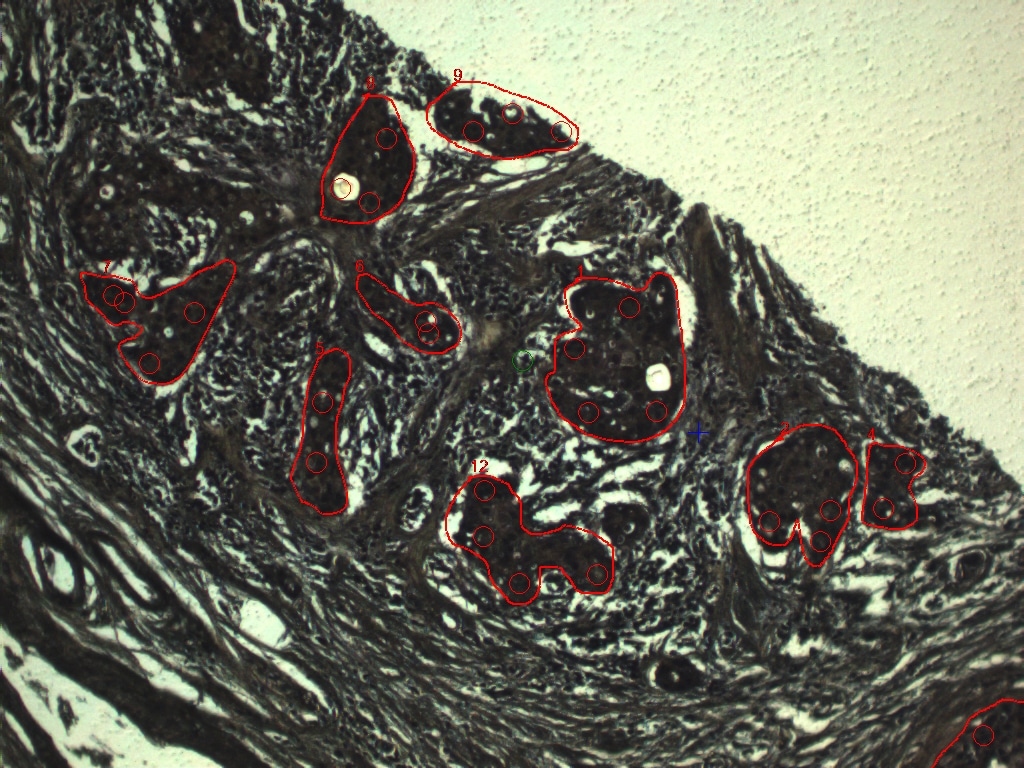

Supplement: Supplemental Material [file supp_gr.234807.118_Supplemental_File_4.zip › BULK/DCIS/DCIS-2 BEFORE.jpeg]

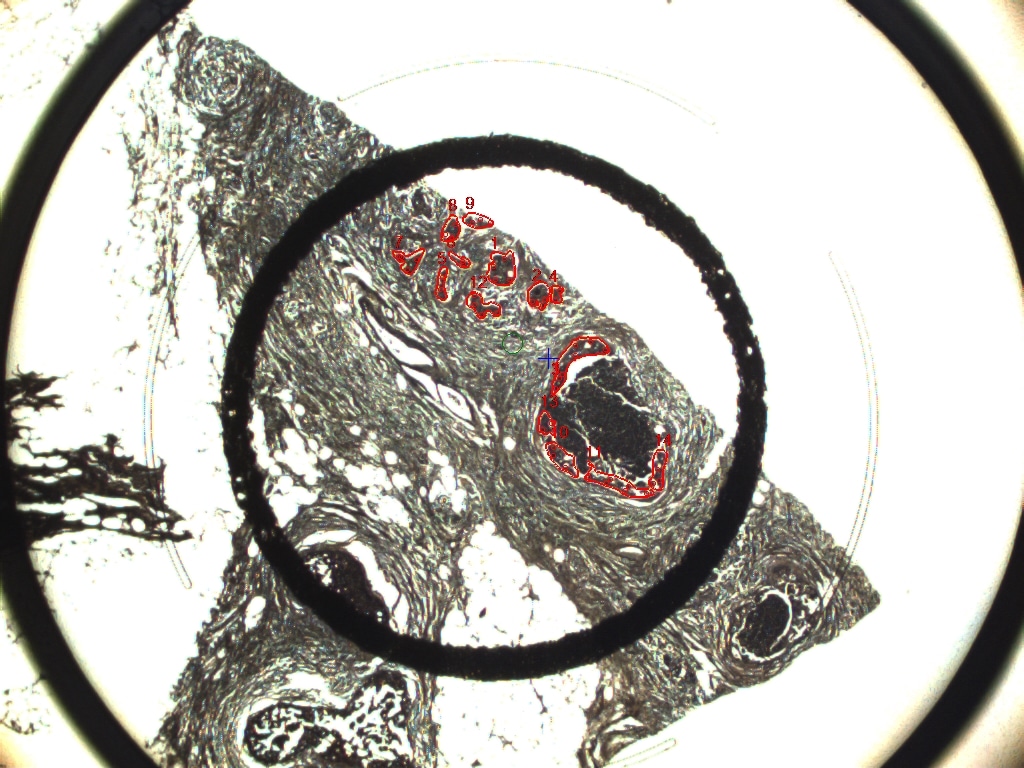

Supplement: Supplemental Material [file supp_gr.234807.118_Supplemental_File_4.zip › BULK/DCIS/DCIS-2 BEFORE2X.jpeg]

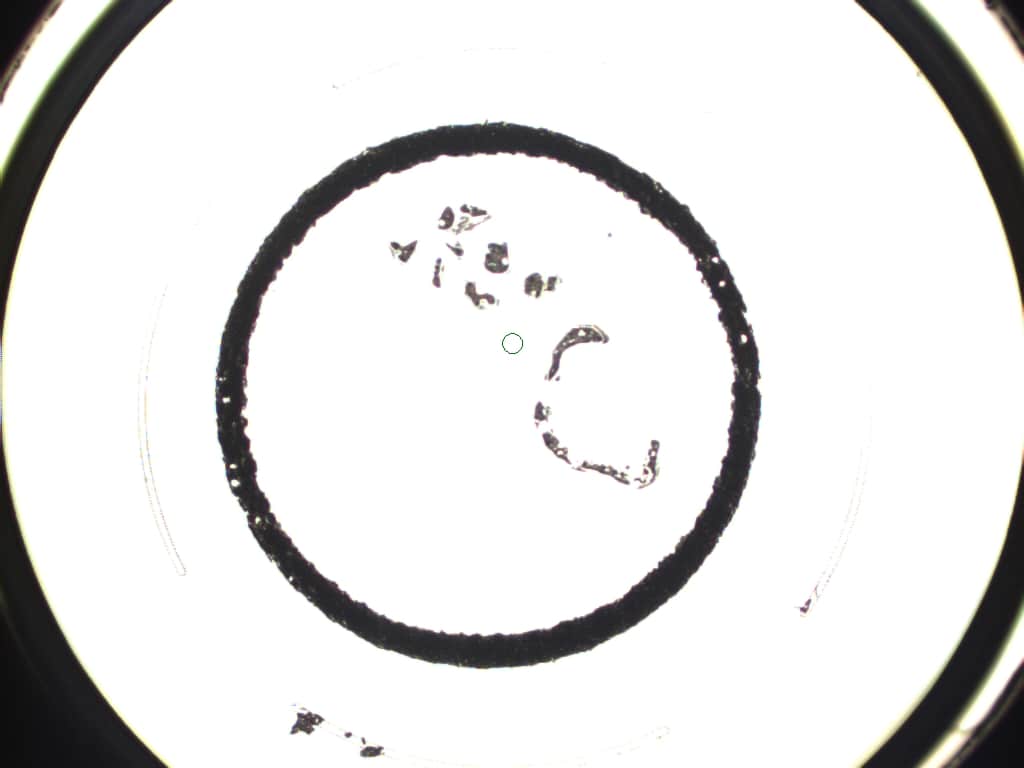

Supplement: Supplemental Material [file supp_gr.234807.118_Supplemental_File_4.zip › BULK/DCIS/DCIS-2.jpeg]

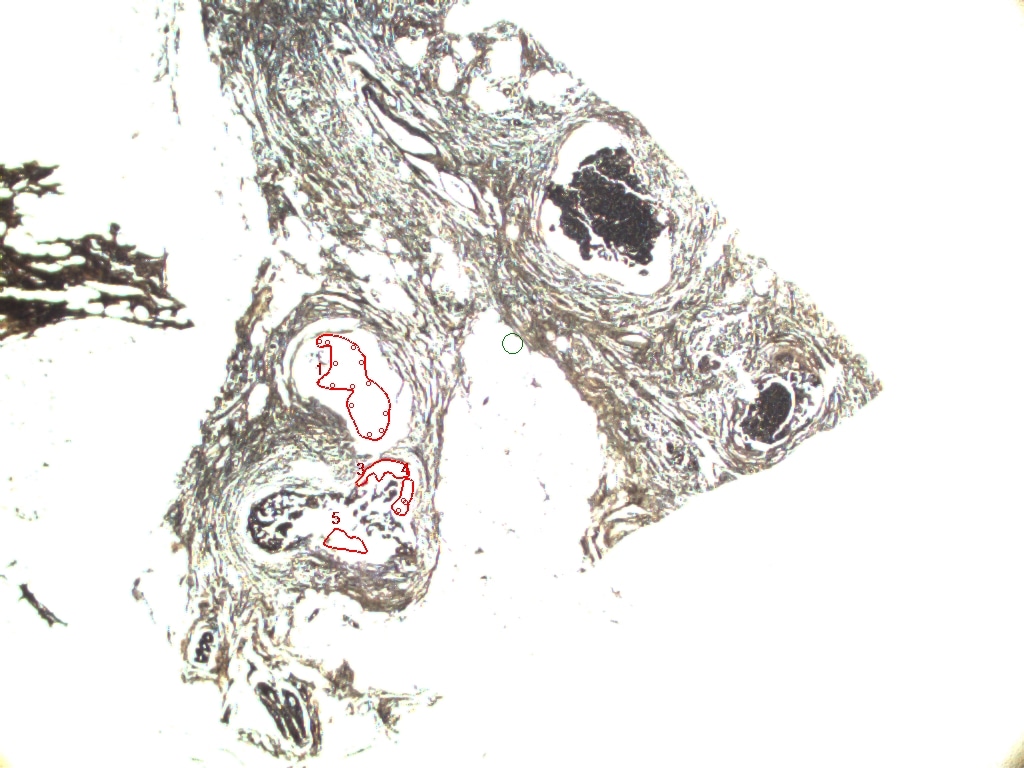

Supplement: Supplemental Material [file supp_gr.234807.118_Supplemental_File_4.zip › BULK/DCIS/DCIS-3 AFTER.jpeg]

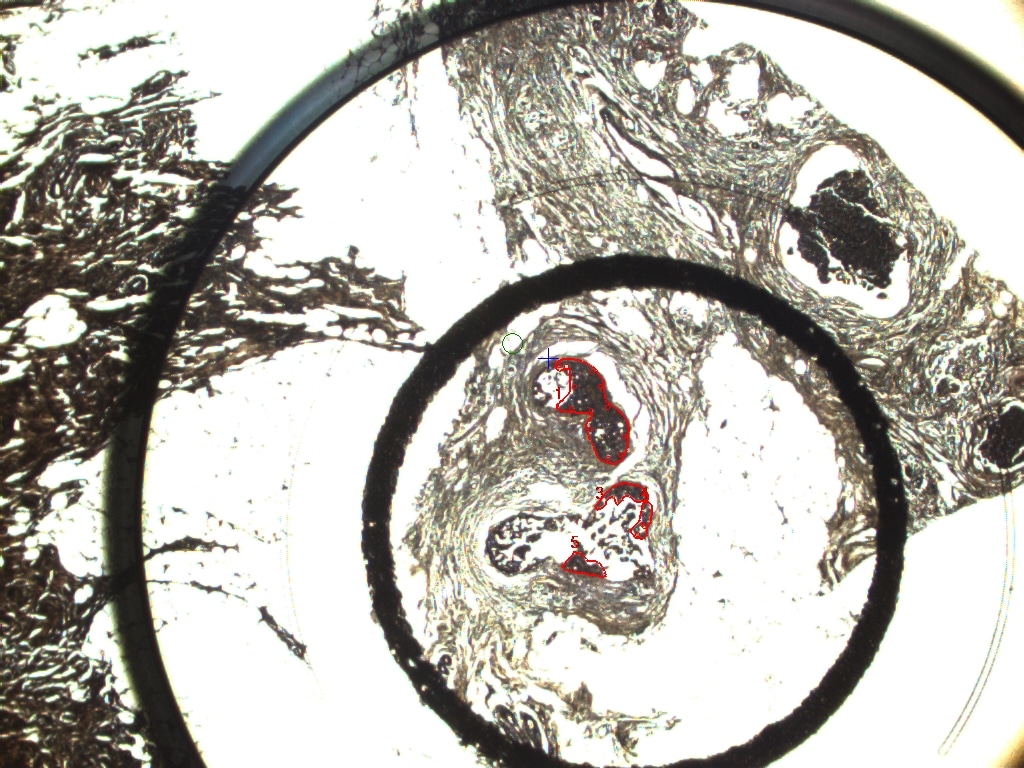

Supplement: Supplemental Material [file supp_gr.234807.118_Supplemental_File_4.zip › BULK/DCIS/DCIS-3 BEFORE.jpeg]

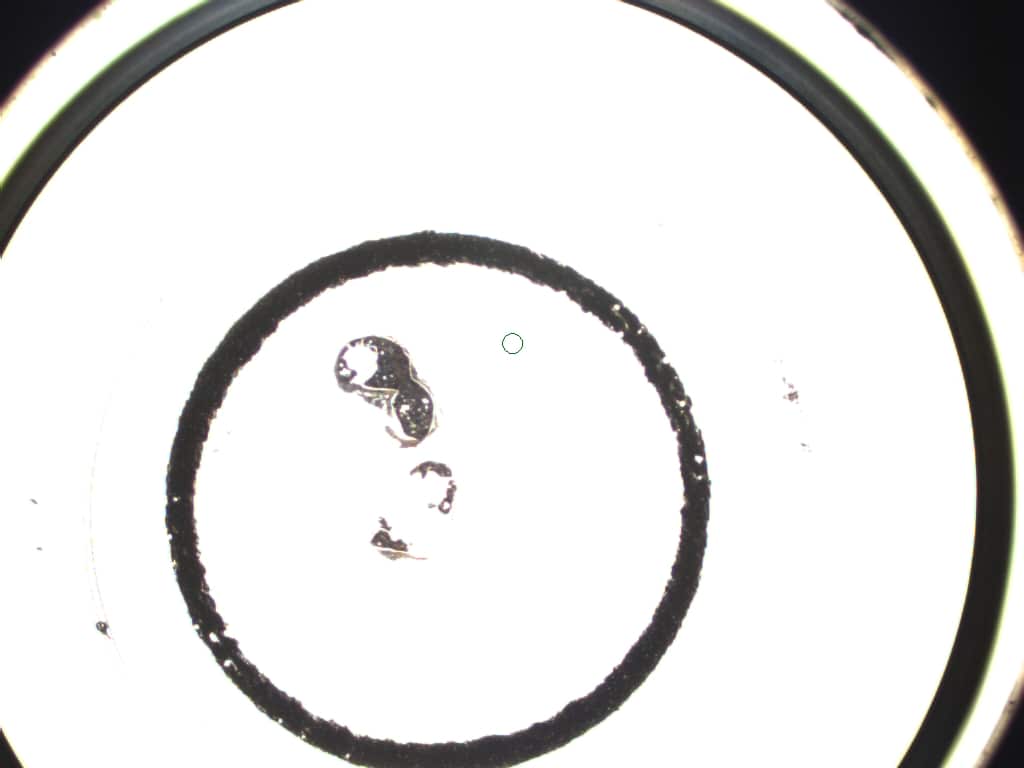

Supplement: Supplemental Material [file supp_gr.234807.118_Supplemental_File_4.zip › BULK/DCIS/DCIS-3.jpeg]

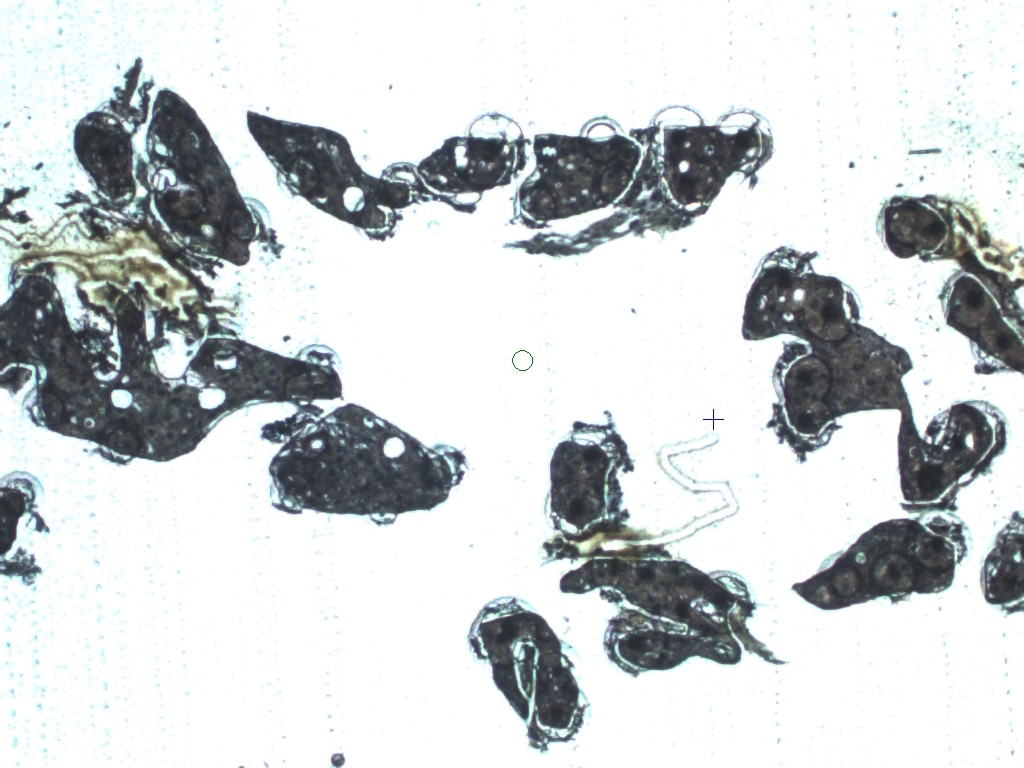

Supplement: Supplemental Material [file supp_gr.234807.118_Supplemental_File_4.zip › BULK/DCIS/DCIS-4 ABLATION.jpeg]

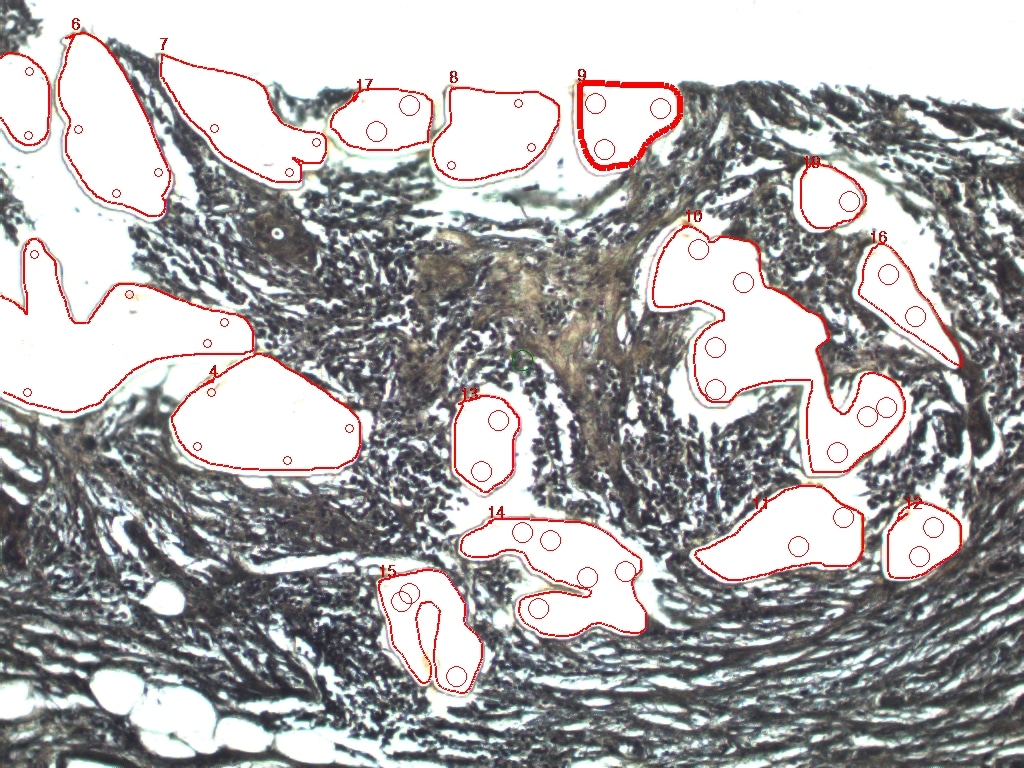

Supplement: Supplemental Material [file supp_gr.234807.118_Supplemental_File_4.zip › BULK/DCIS/DCIS-4 AFTER.jpeg]

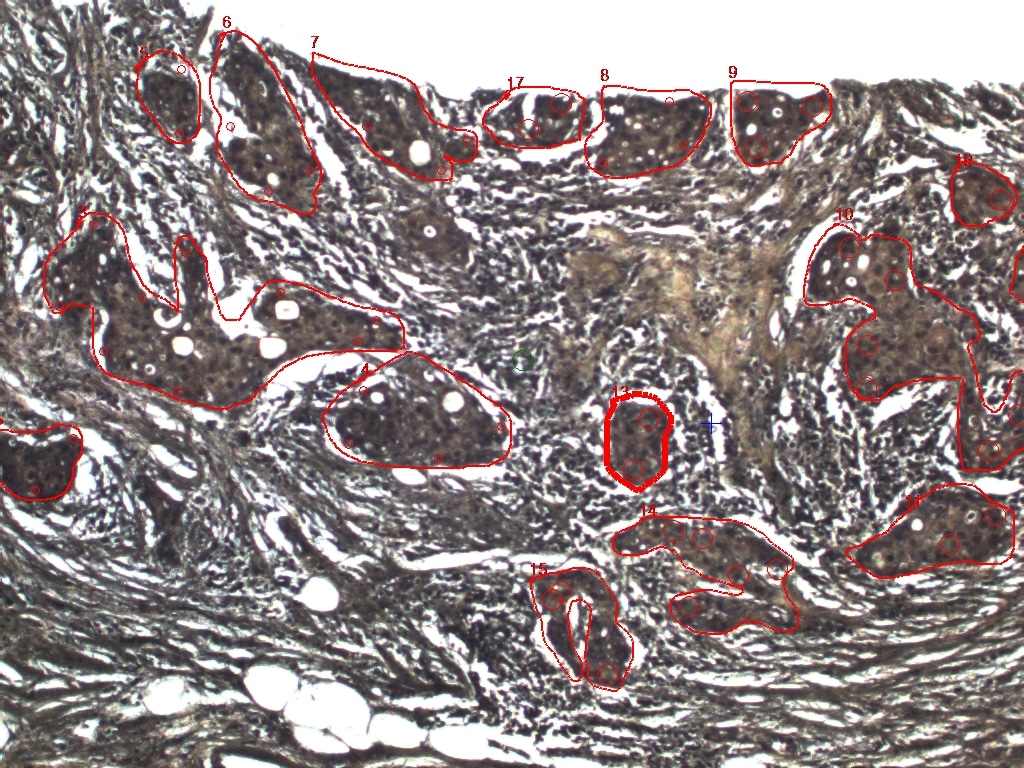

Supplement: Supplemental Material [file supp_gr.234807.118_Supplemental_File_4.zip › BULK/DCIS/DCIS-4 BEFORE .jpeg]

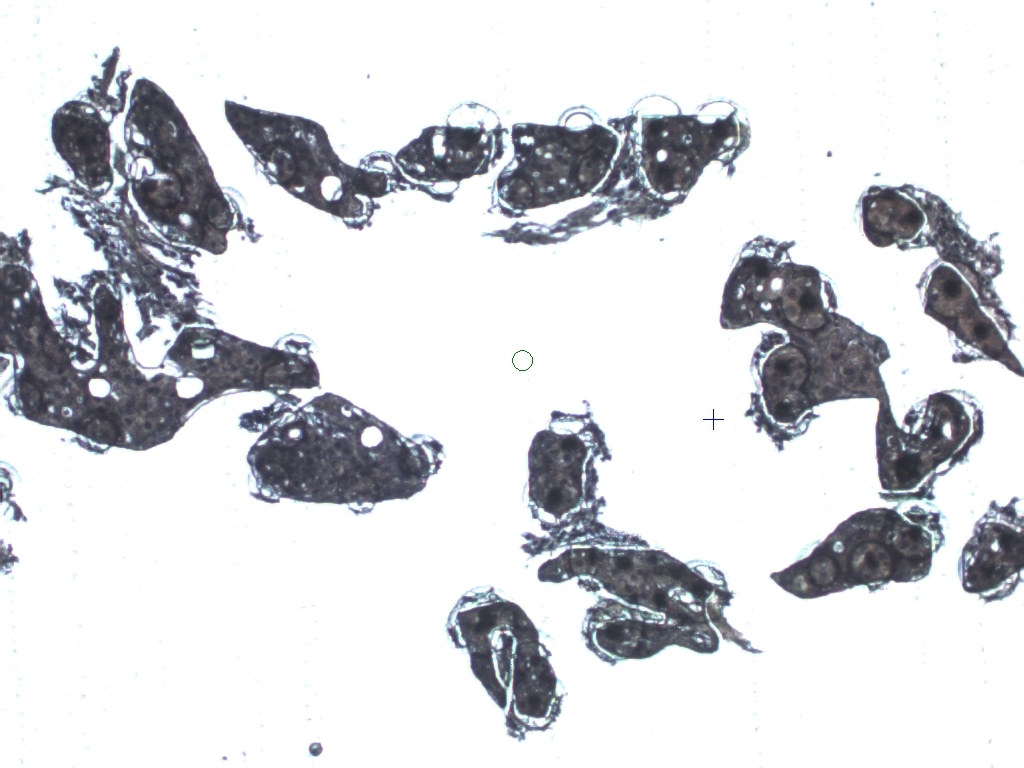

Supplement: Supplemental Material [file supp_gr.234807.118_Supplemental_File_4.zip › BULK/DCIS/DCIS-4.jpeg]

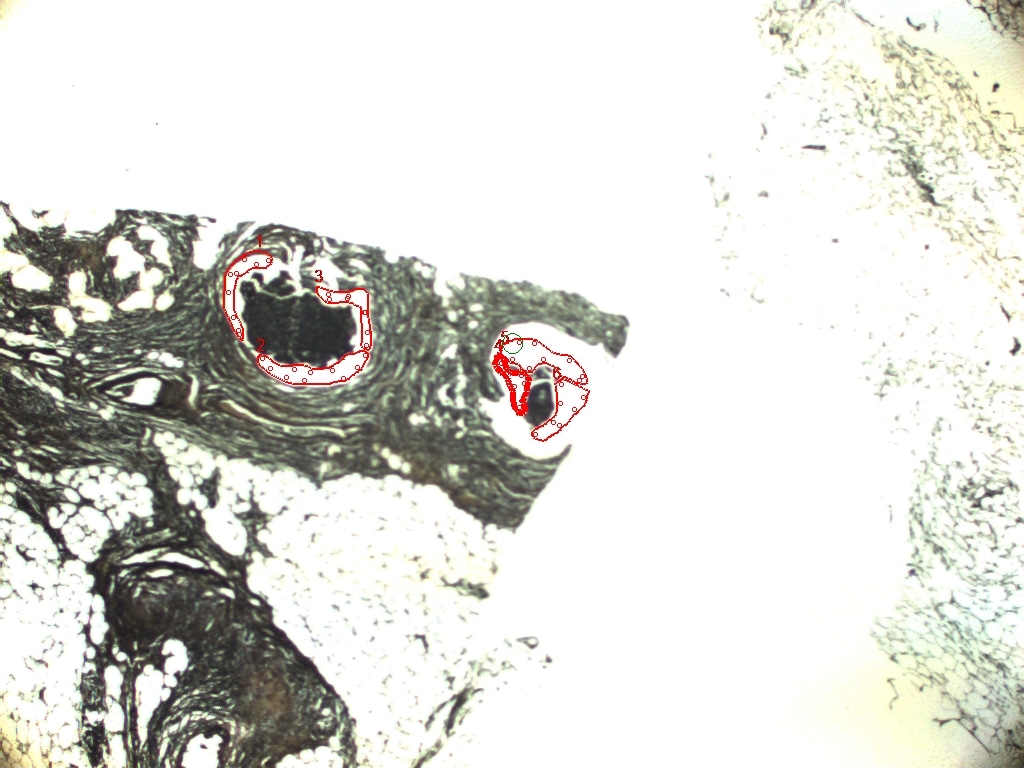

Supplement: Supplemental Material [file supp_gr.234807.118_Supplemental_File_4.zip › BULK/DCIS/DCIS-5 AFTER.jpeg]

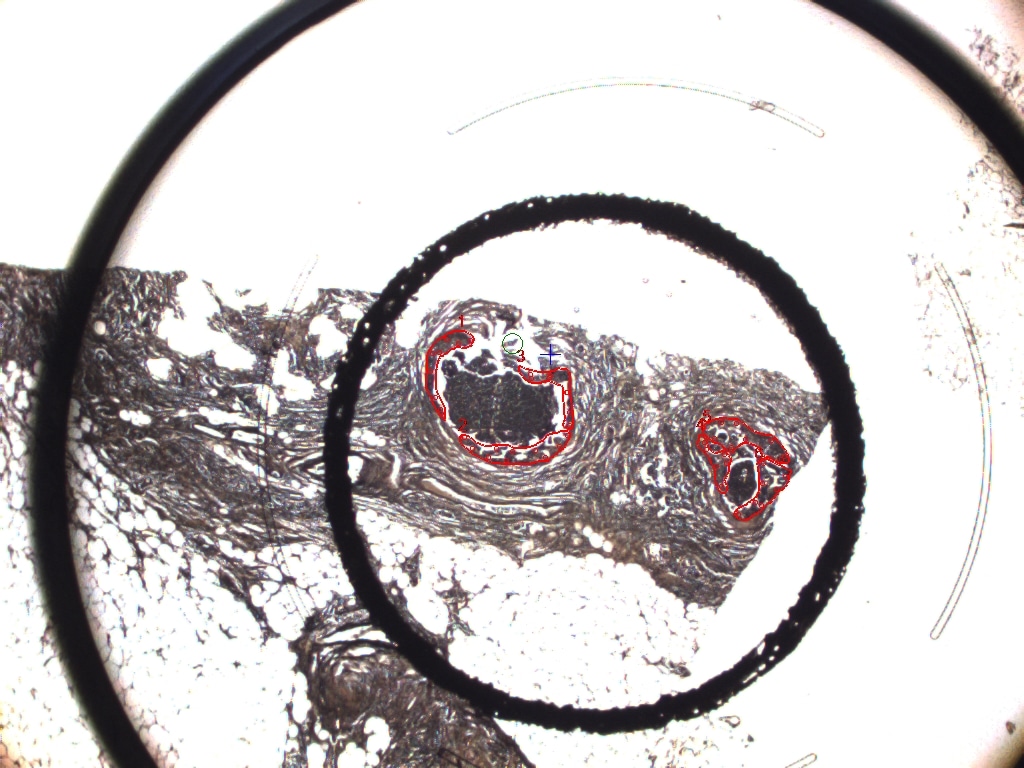

Supplement: Supplemental Material [file supp_gr.234807.118_Supplemental_File_4.zip › BULK/DCIS/DCIS-5 BEFORE .jpeg]

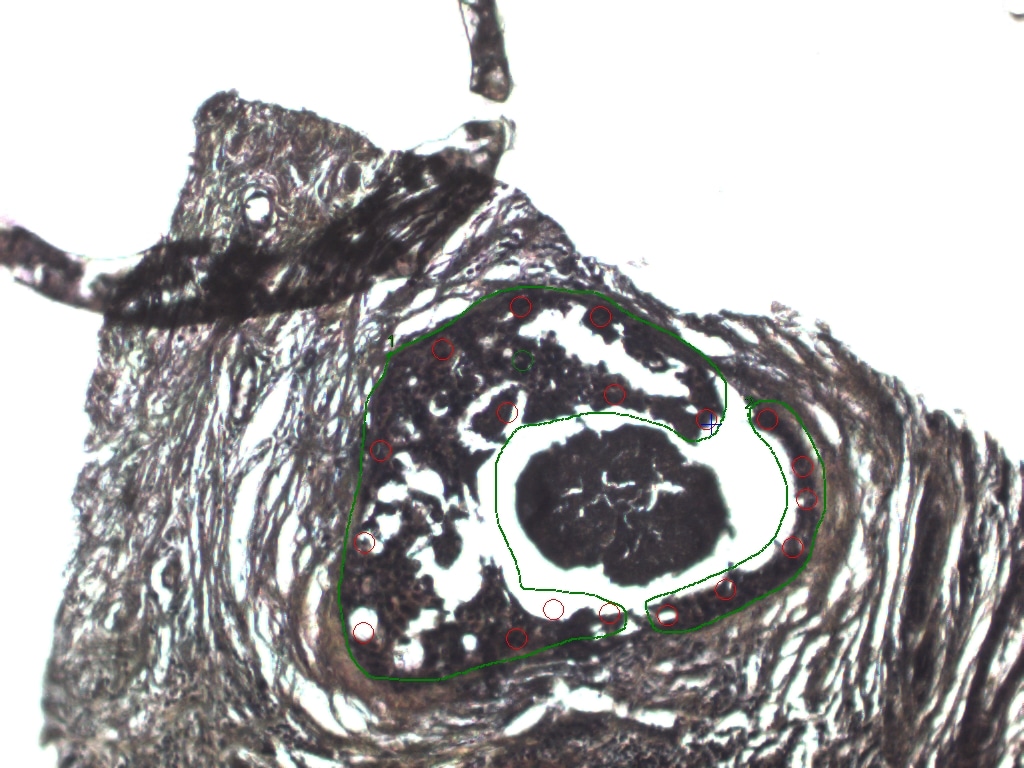

Supplement: Supplemental Material [file supp_gr.234807.118_Supplemental_File_4.zip › BULK/DCIS/DCIS-5 BEFORE2 .jpeg]

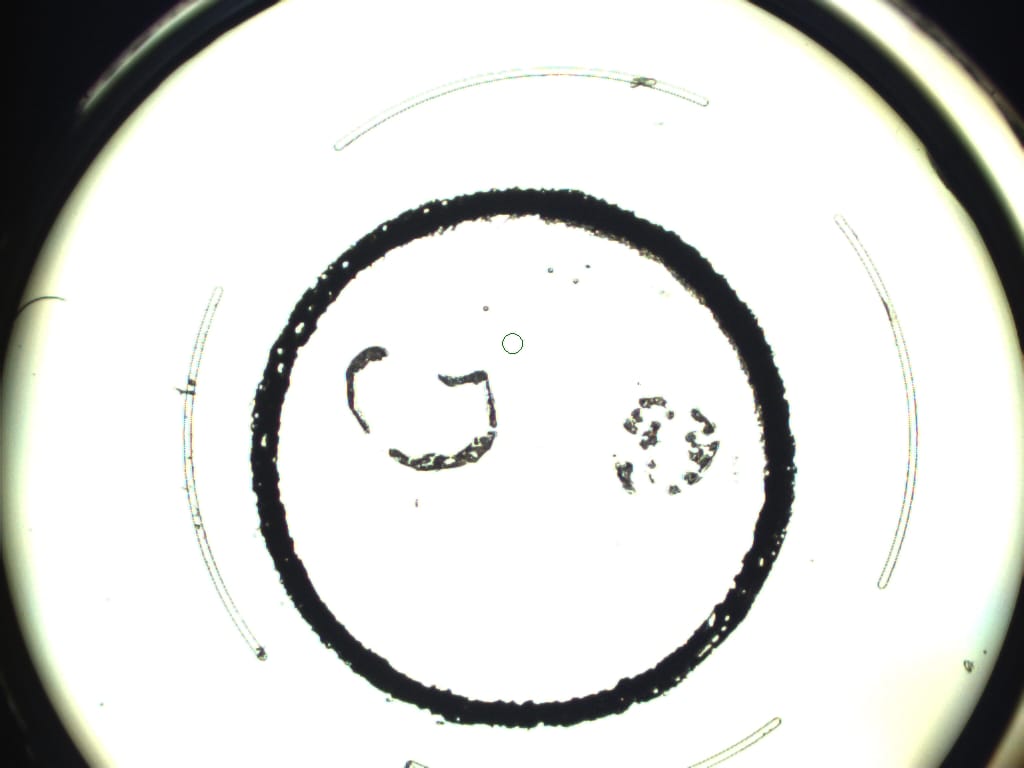

Supplement: Supplemental Material [file supp_gr.234807.118_Supplemental_File_4.zip › BULK/DCIS/DCIS-5.jpeg]

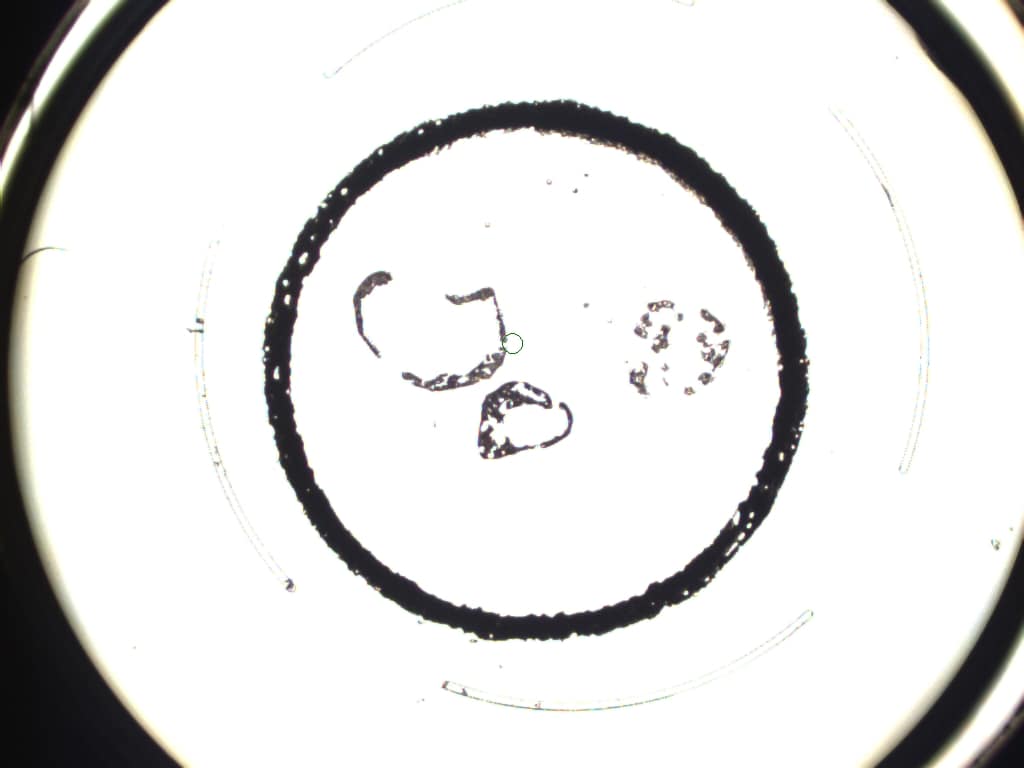

Supplement: Supplemental Material [file supp_gr.234807.118_Supplemental_File_4.zip › BULK/DCIS/DCIS-5FINAL.jpeg]

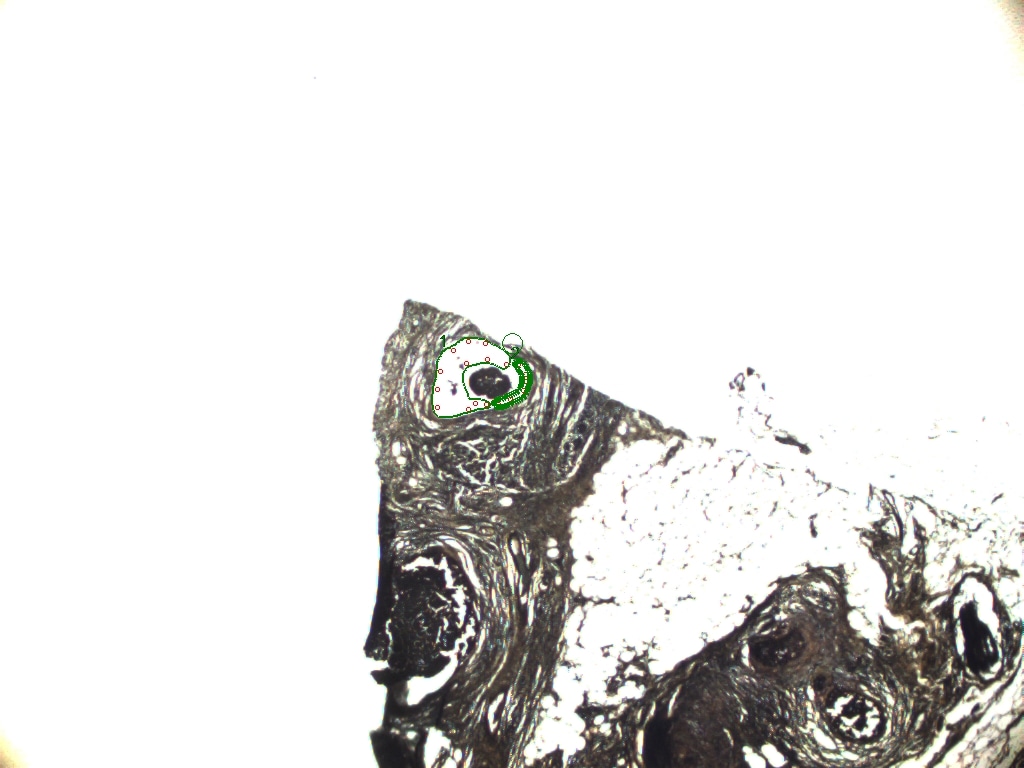

Supplement: Supplemental Material [file supp_gr.234807.118_Supplemental_File_4.zip › BULK/DCIS/DCIS-6 AFTER.jpeg]

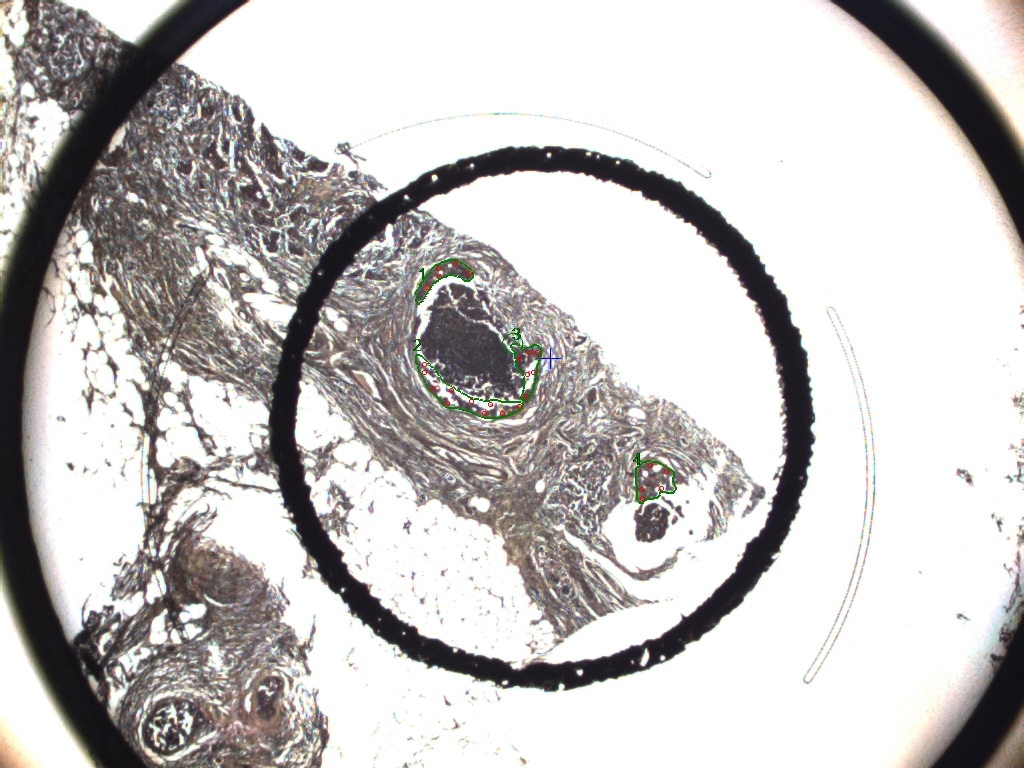

Supplement: Supplemental Material [file supp_gr.234807.118_Supplemental_File_4.zip › BULK/DCIS/DCIS-6 BEFORE .jpeg]

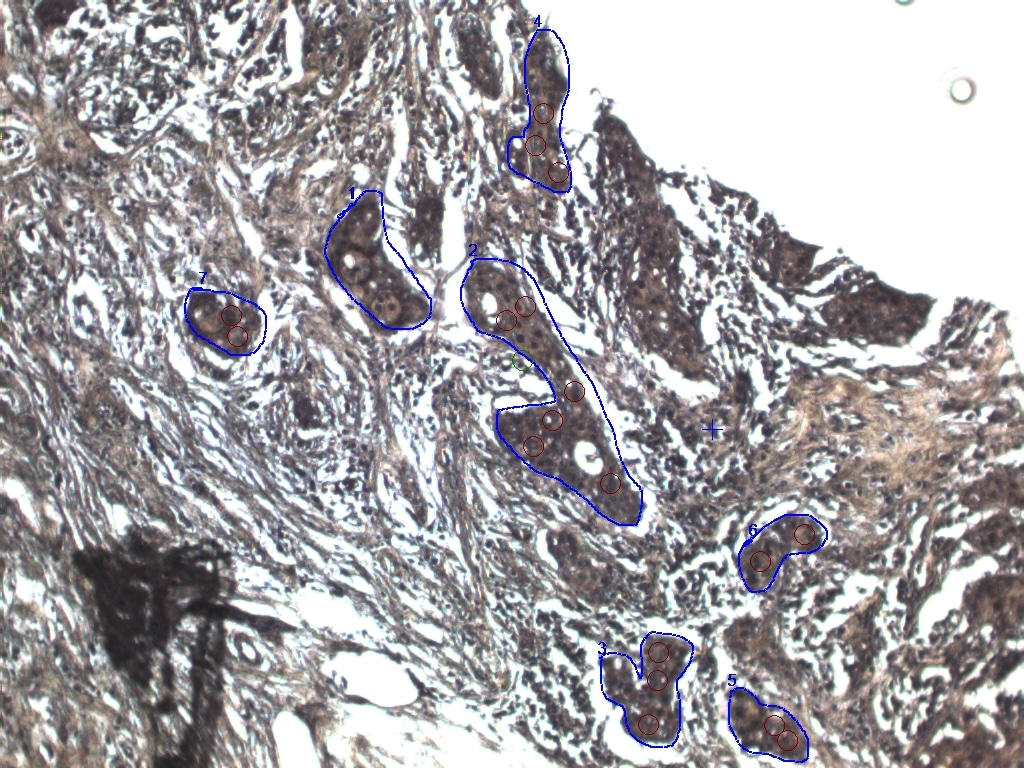

Supplement: Supplemental Material [file supp_gr.234807.118_Supplemental_File_4.zip › BULK/DCIS/DCIS-6 BEFORE2 .jpeg]

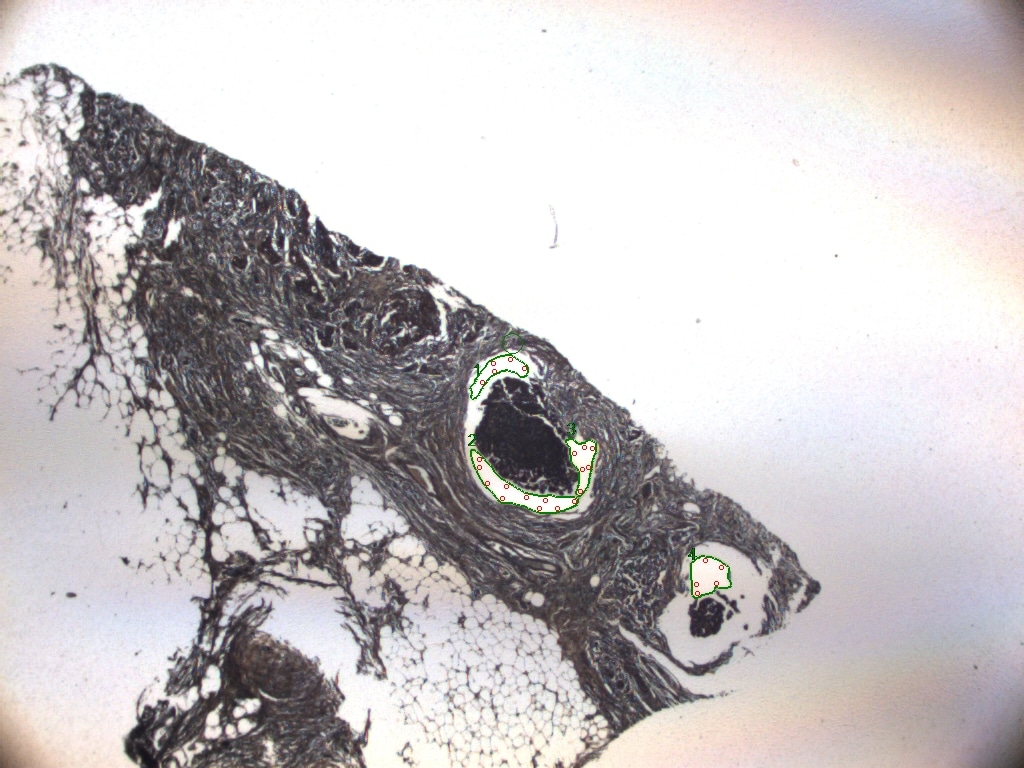

Supplement: Supplemental Material [file supp_gr.234807.118_Supplemental_File_4.zip › BULK/DCIS/DCIS-6 RIGHTAFTER.jpeg]

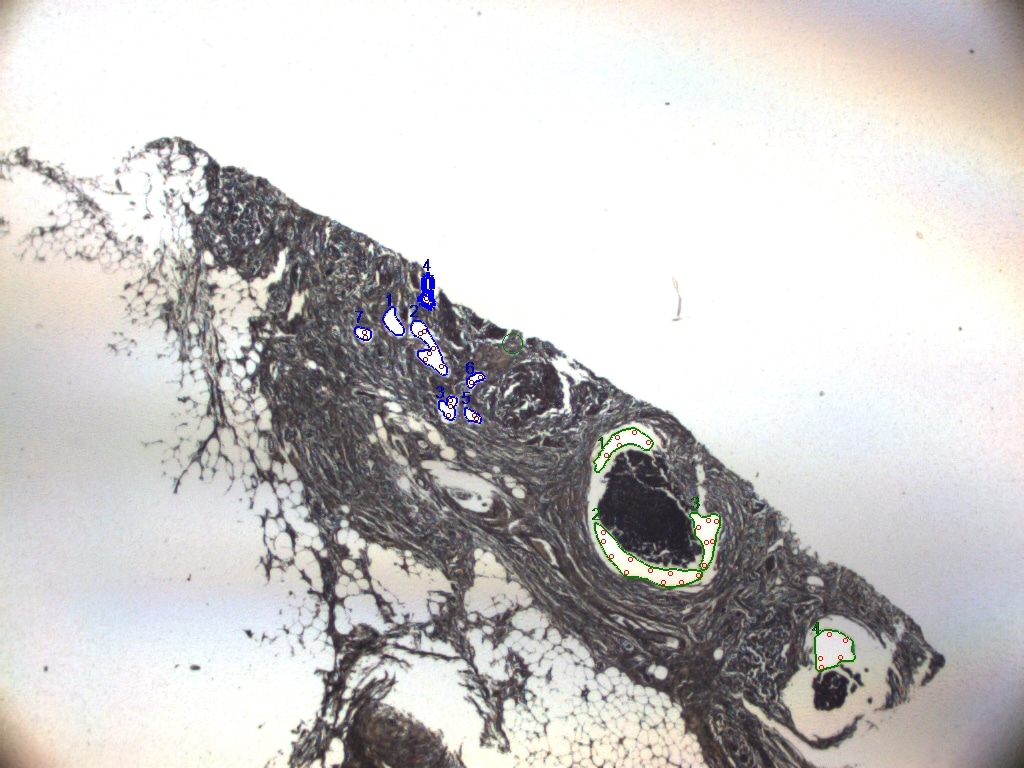

Supplement: Supplemental Material [file supp_gr.234807.118_Supplemental_File_4.zip › BULK/DCIS/DCIS-6 RIGHTAFTER2.jpeg]

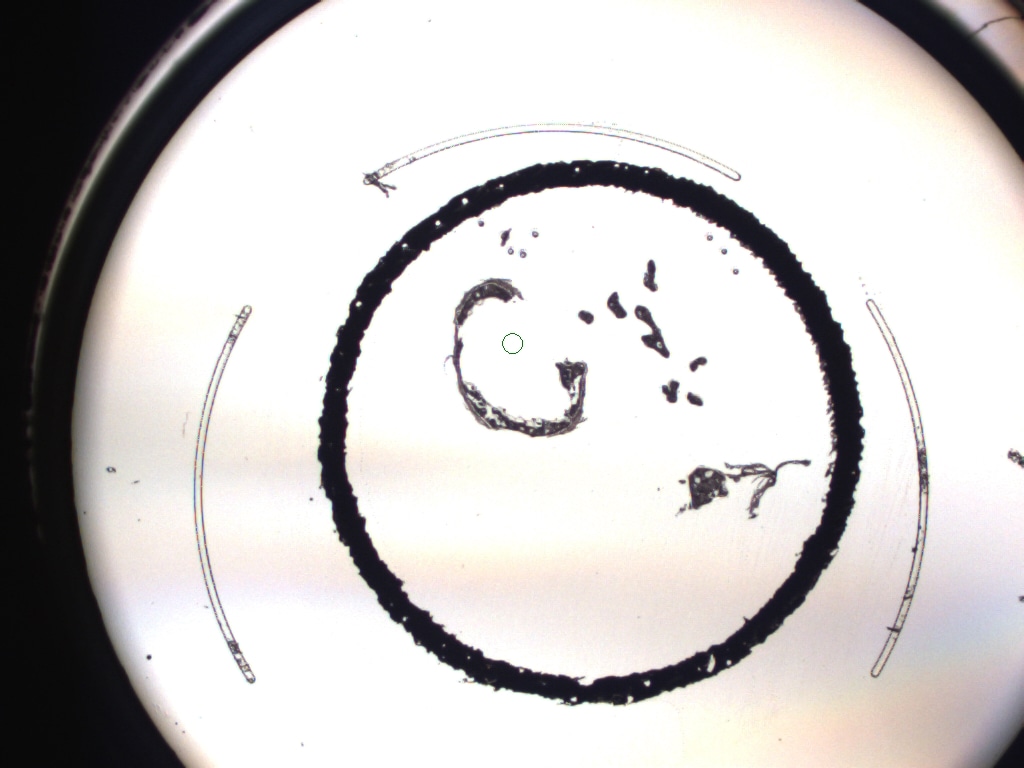

Supplement: Supplemental Material [file supp_gr.234807.118_Supplemental_File_4.zip › BULK/DCIS/DCIS-6FINAL.jpeg]

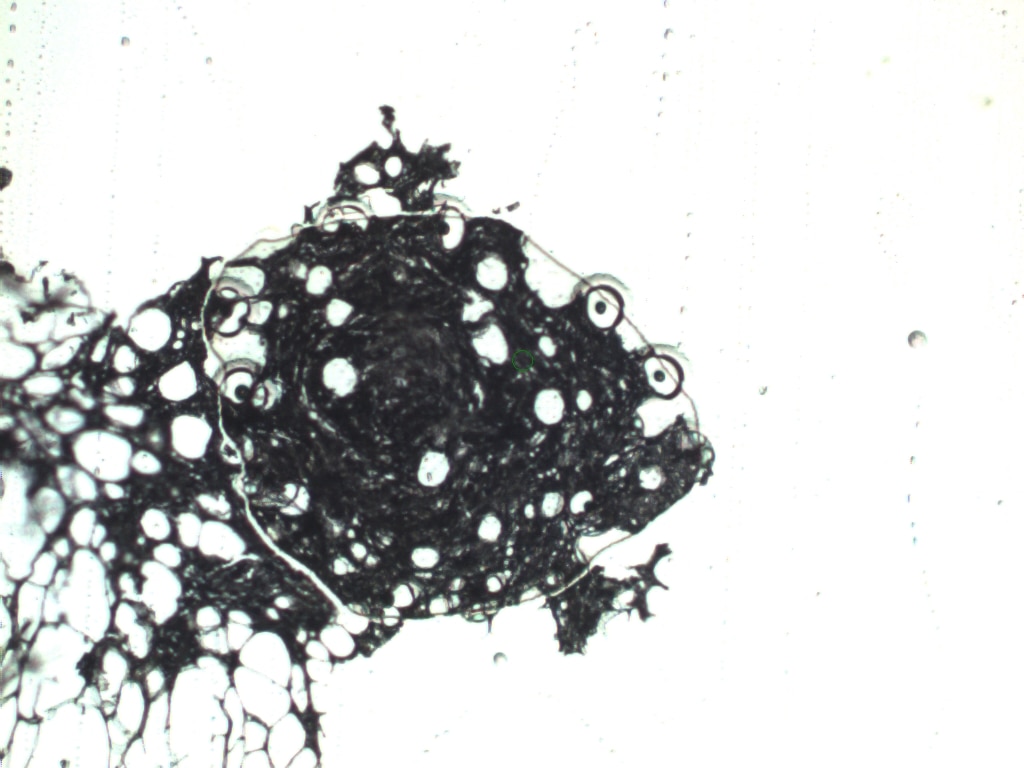

Supplement: Supplemental Material [file supp_gr.234807.118_Supplemental_File_4.zip › BULK/MAC/MAC1 .jpeg]

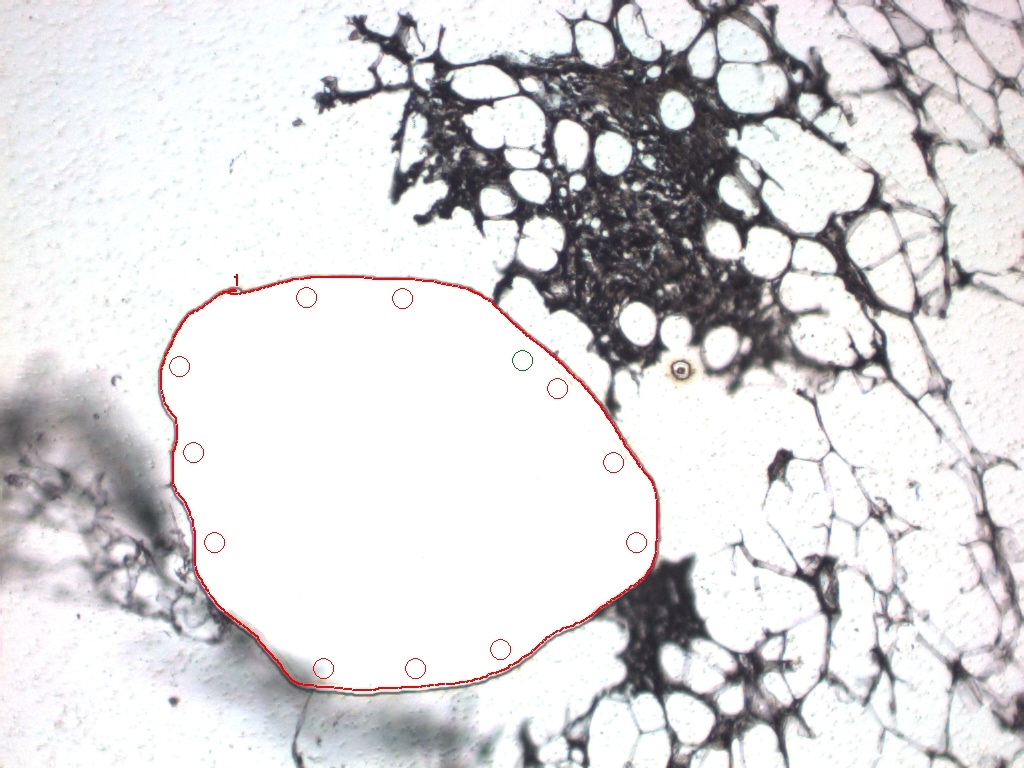

Supplement: Supplemental Material [file supp_gr.234807.118_Supplemental_File_4.zip › BULK/MAC/MAC1 AFTER.jpeg]

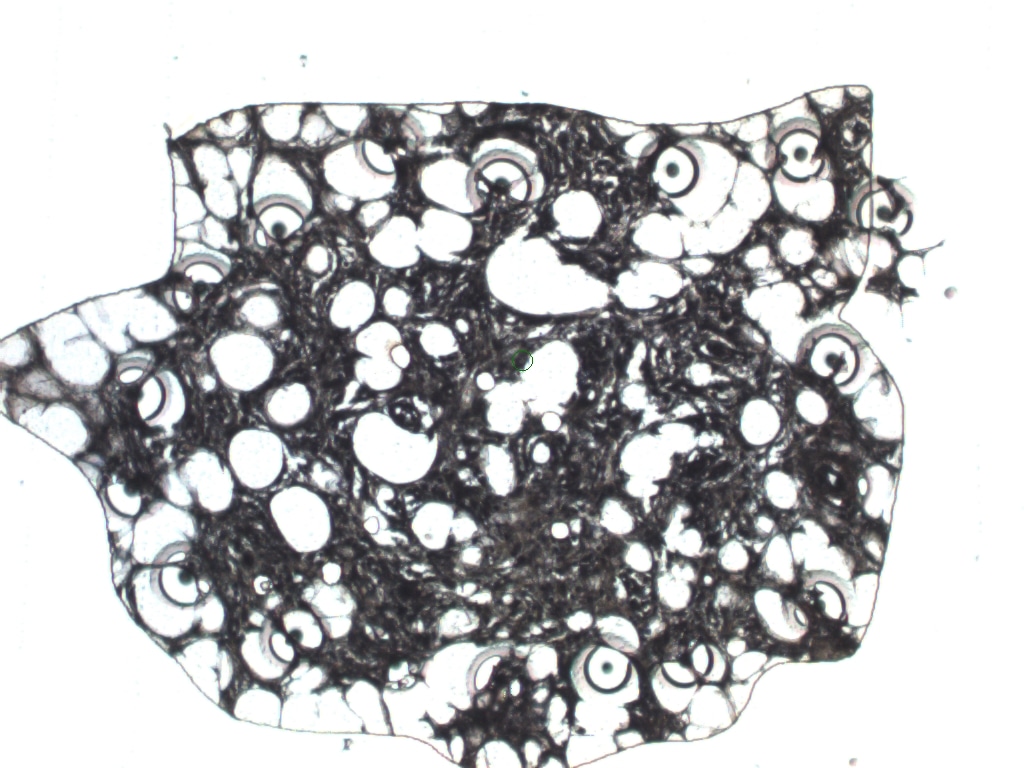

Supplement: Supplemental Material [file supp_gr.234807.118_Supplemental_File_4.zip › BULK/MAC/MAC2 .jpeg]

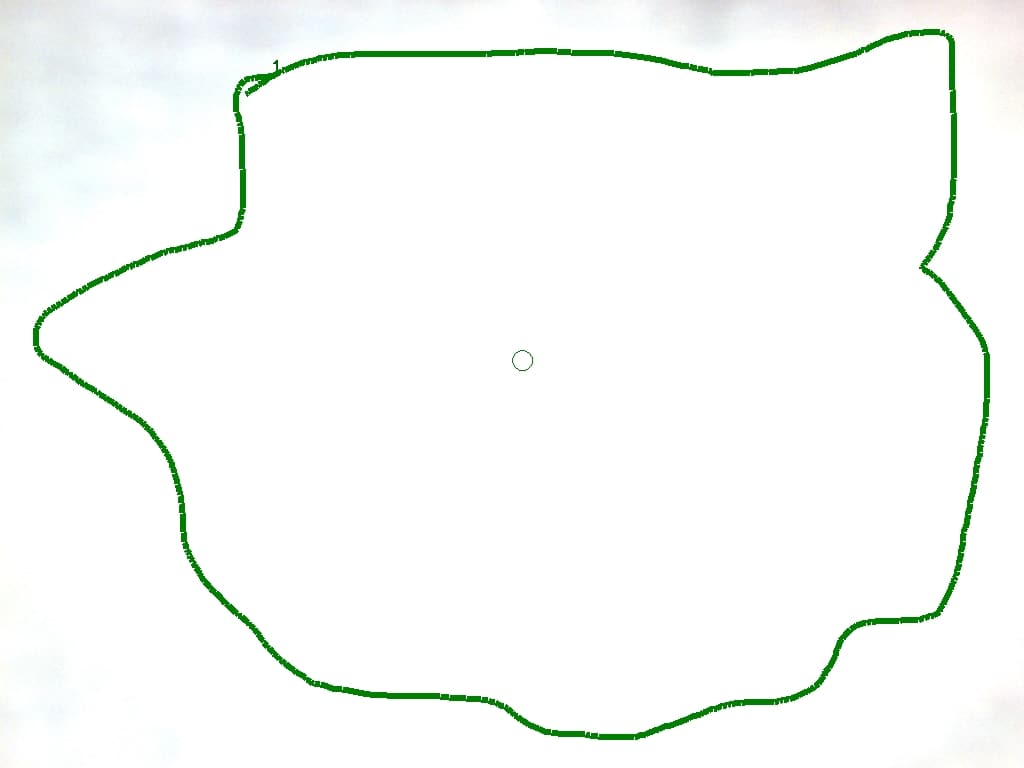

Supplement: Supplemental Material [file supp_gr.234807.118_Supplemental_File_4.zip › BULK/MAC/MAC2AFTER.jpeg]

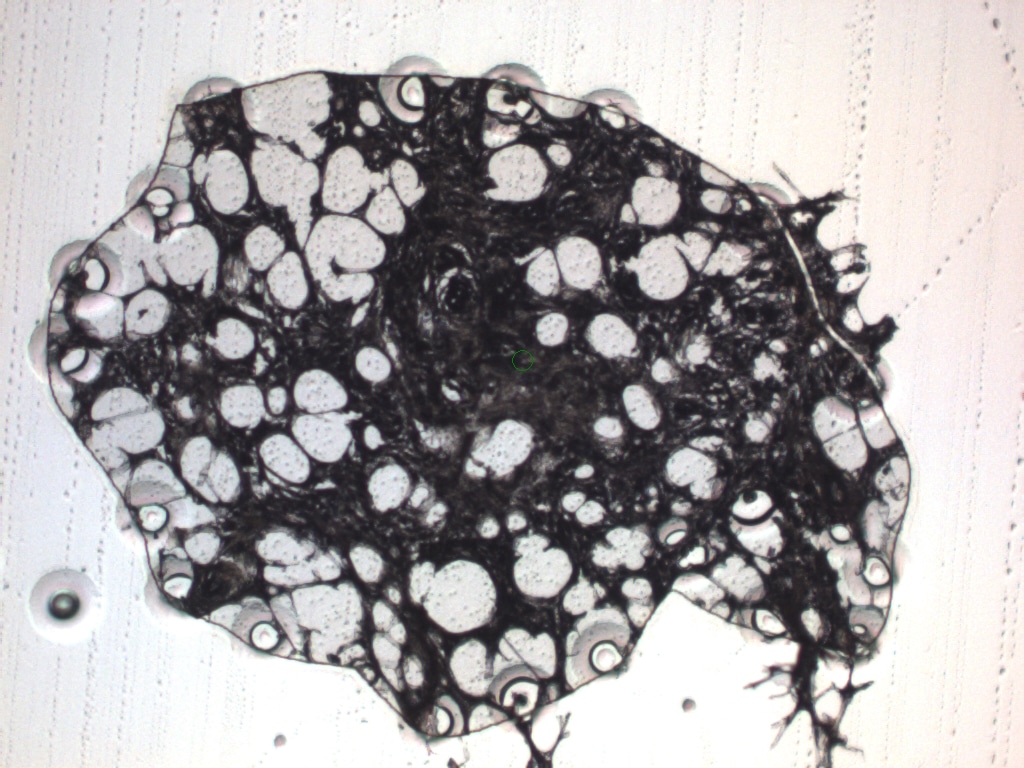

Supplement: Supplemental Material [file supp_gr.234807.118_Supplemental_File_4.zip › BULK/MAC/MAC3 AFTER.jpeg]

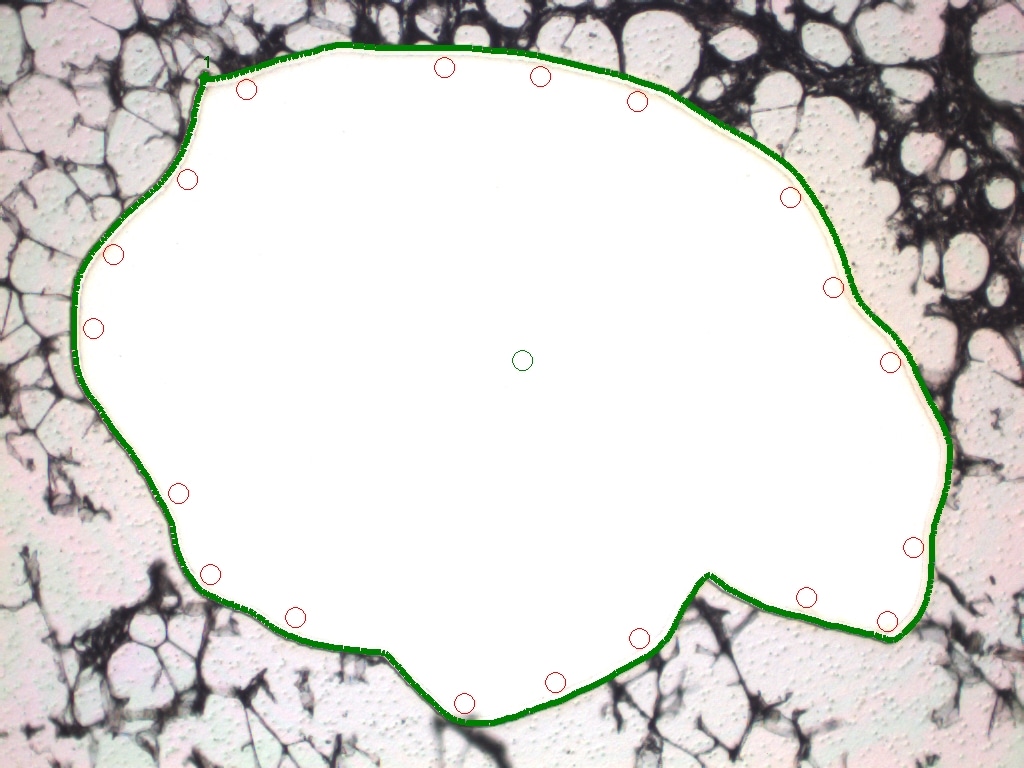

Supplement: Supplemental Material [file supp_gr.234807.118_Supplemental_File_4.zip › BULK/MAC/MAC3AFTER RIGHT.jpeg]

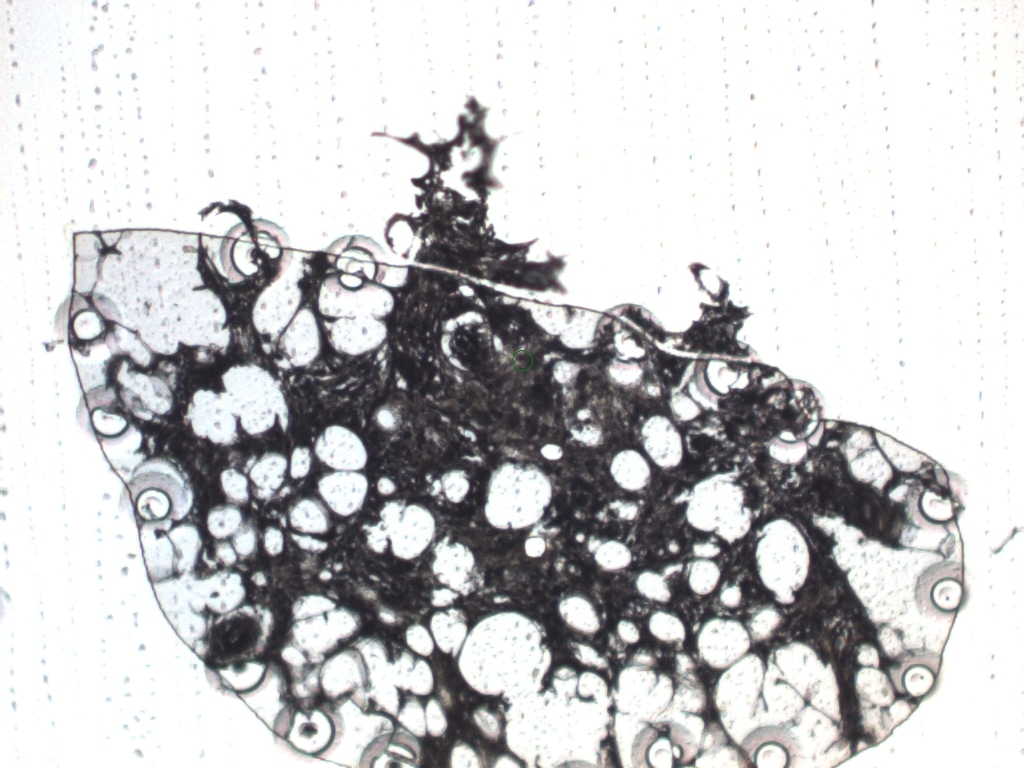

Supplement: Supplemental Material [file supp_gr.234807.118_Supplemental_File_4.zip › BULK/MAC/MAC4 .jpeg]

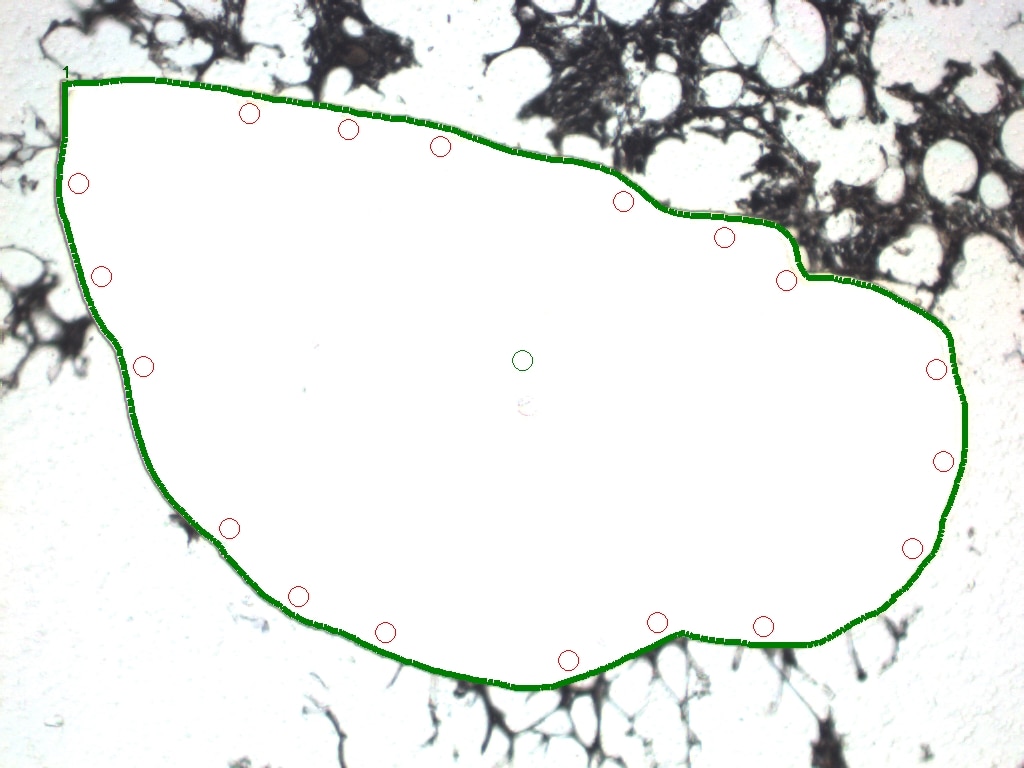

Supplement: Supplemental Material [file supp_gr.234807.118_Supplemental_File_4.zip › BULK/MAC/MAC4 AFTER.jpeg]

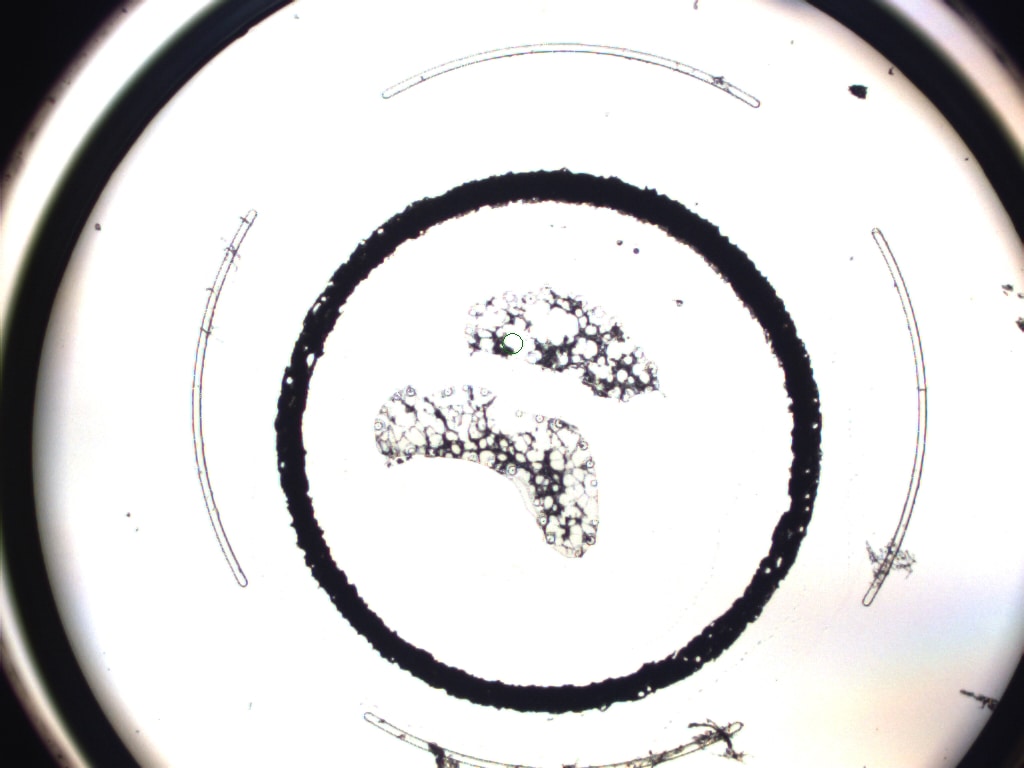

Supplement: Supplemental Material [file supp_gr.234807.118_Supplemental_File_4.zip › BULK/MAC/MAC5 .jpeg]

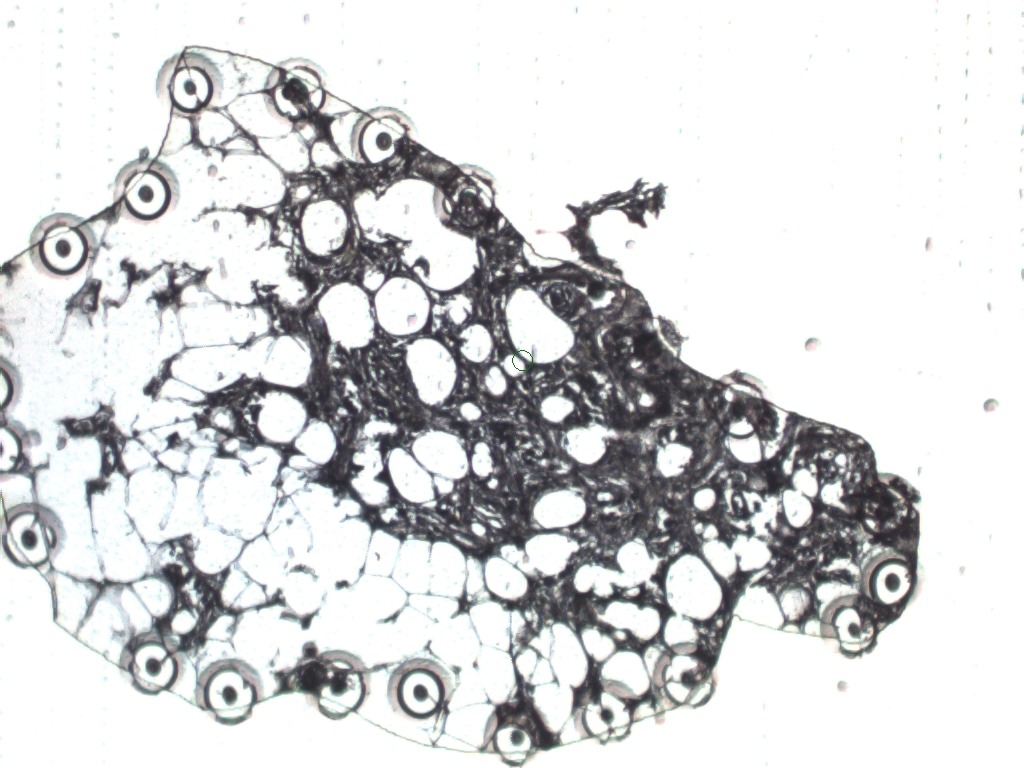

Supplement: Supplemental Material [file supp_gr.234807.118_Supplemental_File_4.zip › BULK/MAC/MAC6 .jpeg]

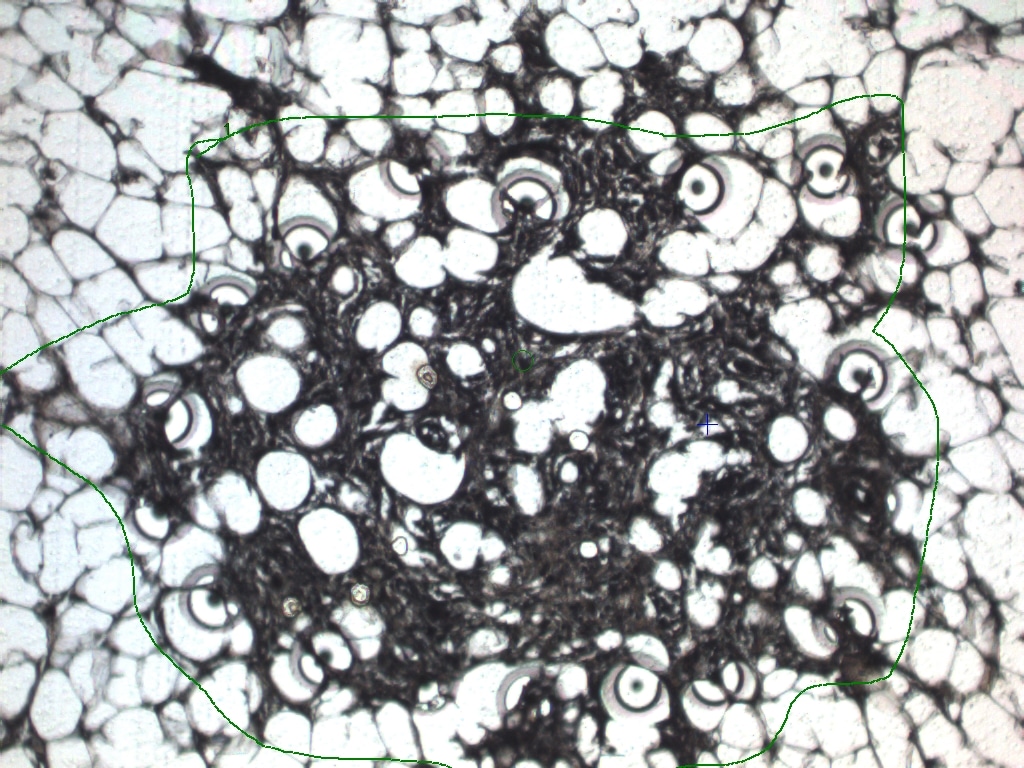

Supplement: Supplemental Material [file supp_gr.234807.118_Supplemental_File_4.zip › BULK/MAC/MACROPHAGE2 BEFORE 10x.jpeg]

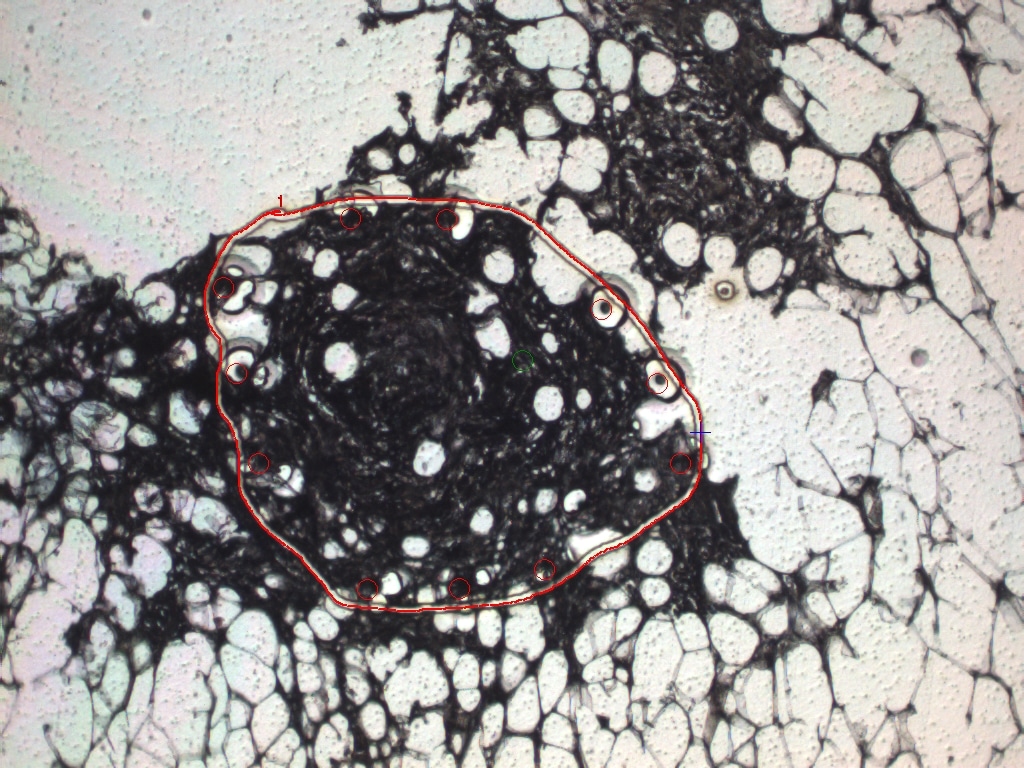

Supplement: Supplemental Material [file supp_gr.234807.118_Supplemental_File_4.zip › BULK/MAC/MACROPHAGE2BEFORE 10x.jpeg]

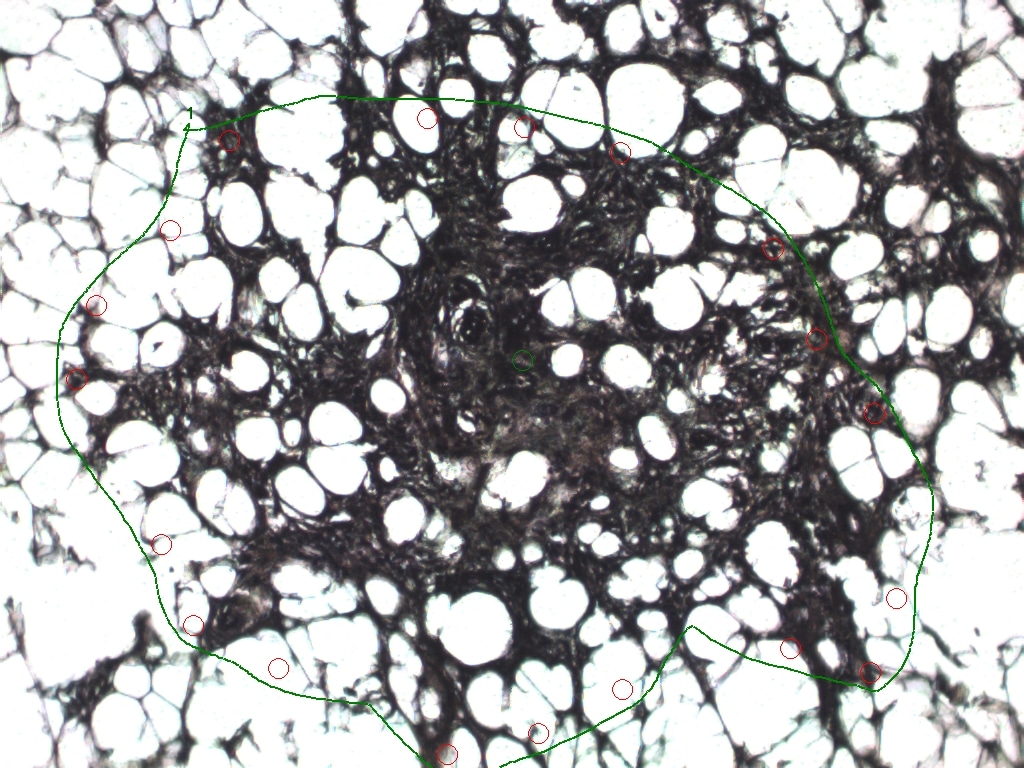

Supplement: Supplemental Material [file supp_gr.234807.118_Supplemental_File_4.zip › BULK/MAC/MACROPHAGE3 BEFORE 10x.jpeg]

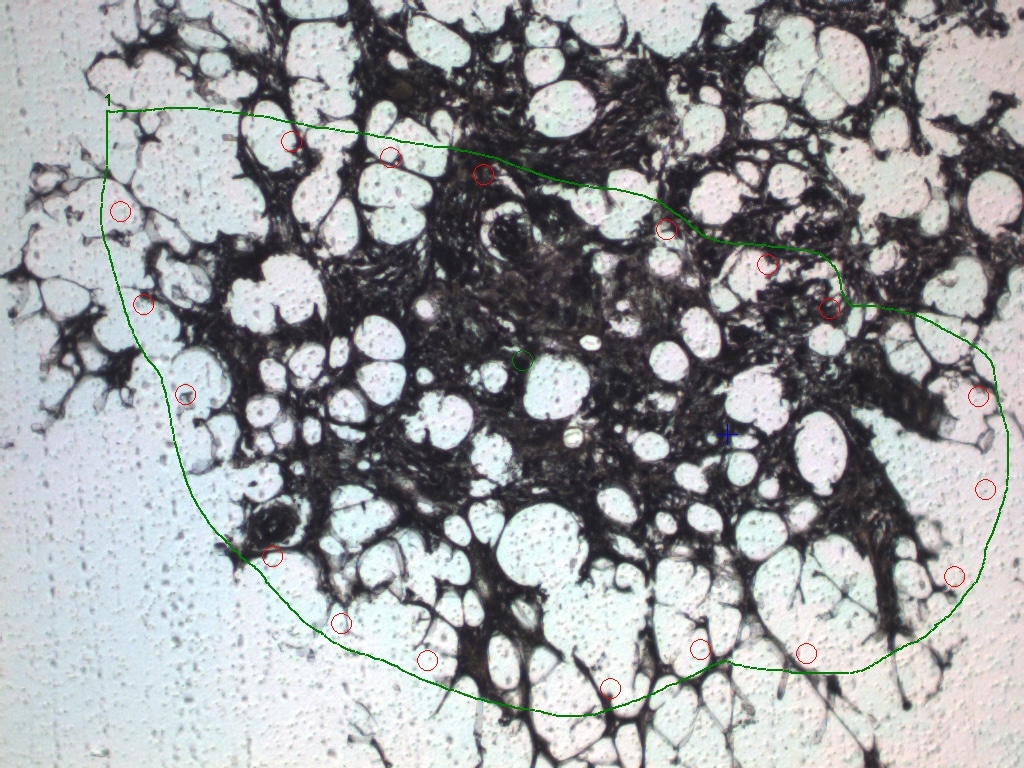

Supplement: Supplemental Material [file supp_gr.234807.118_Supplemental_File_4.zip › BULK/MAC/MACROPHAGE4 BEFORE 10x.jpeg]

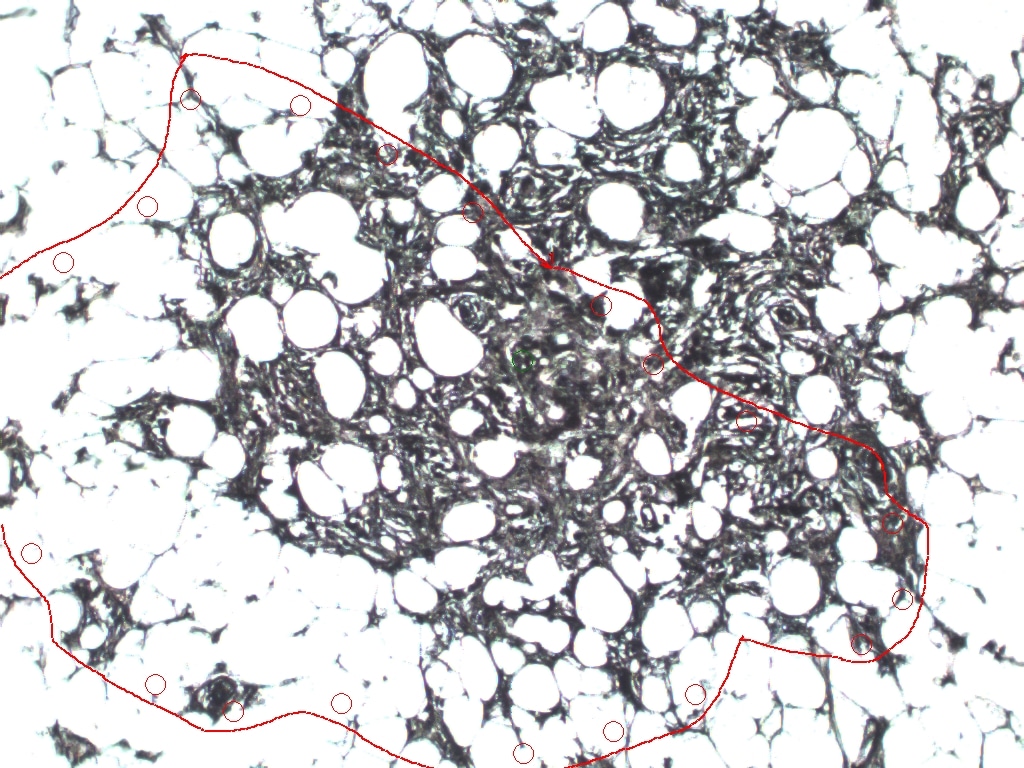

Supplement: Supplemental Material [file supp_gr.234807.118_Supplemental_File_4.zip › BULK/MAC/MACROPHAGE6 BEFORE 10x.jpeg]

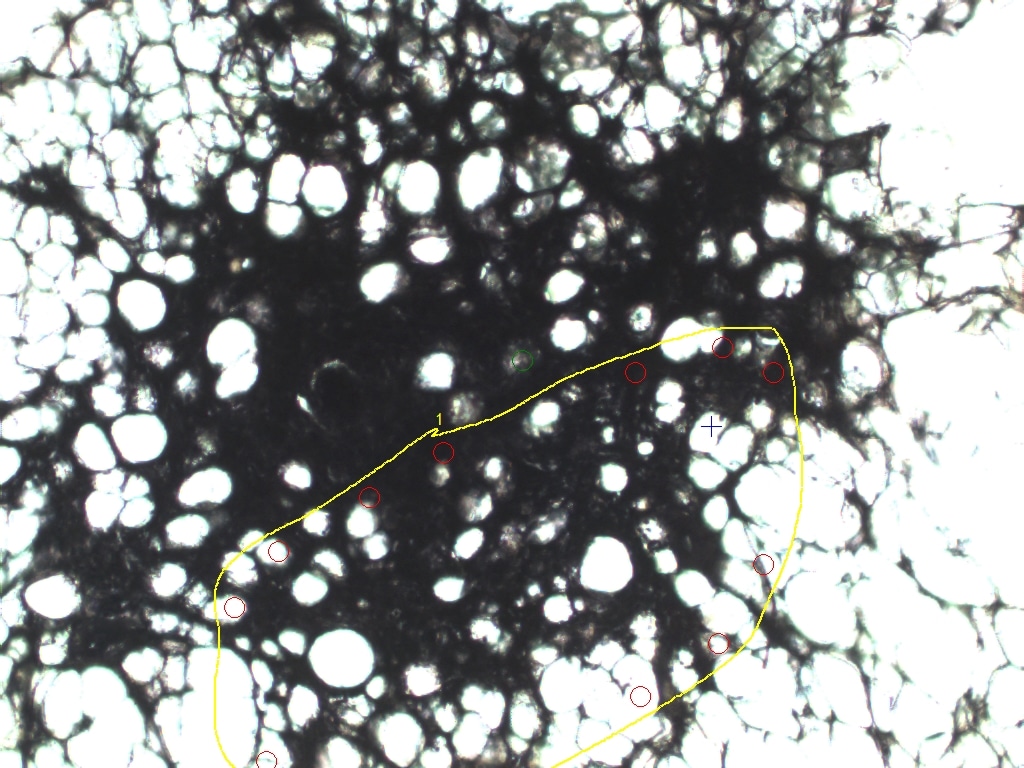

Supplement: Supplemental Material [file supp_gr.234807.118_Supplemental_File_4.zip › BULK/MAC/MACROPHAGE6 BEFORE 2.jpeg]

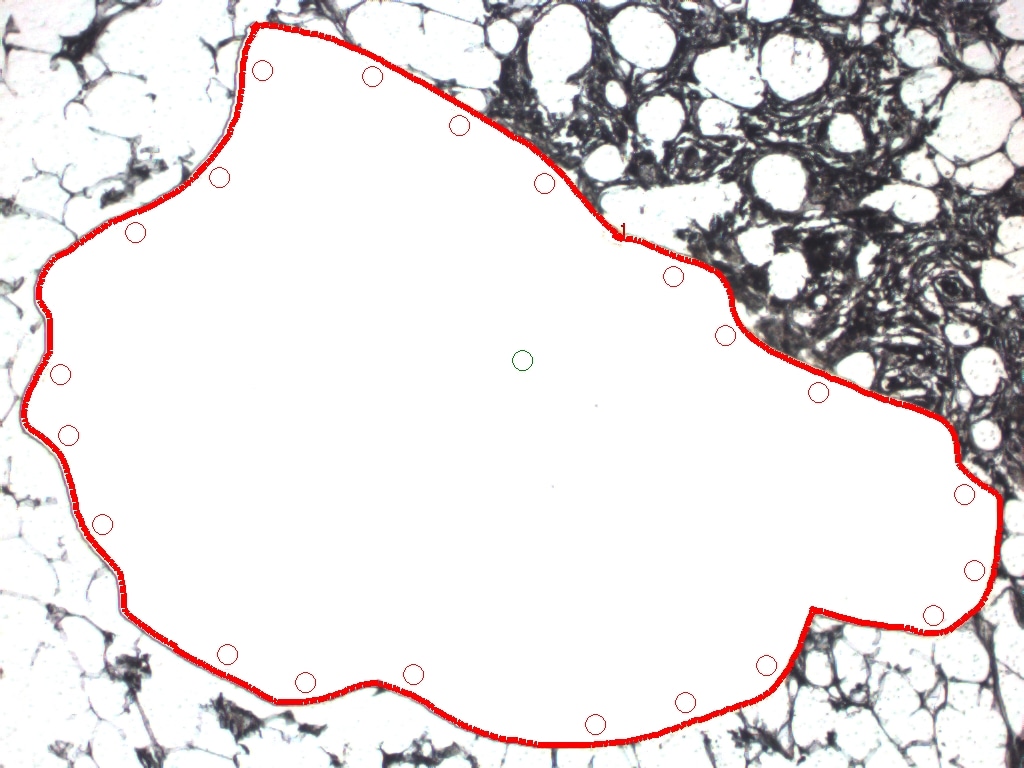

Supplement: Supplemental Material [file supp_gr.234807.118_Supplemental_File_4.zip › BULK/MAC/MACROPHAGE6 AFTER.jpeg]

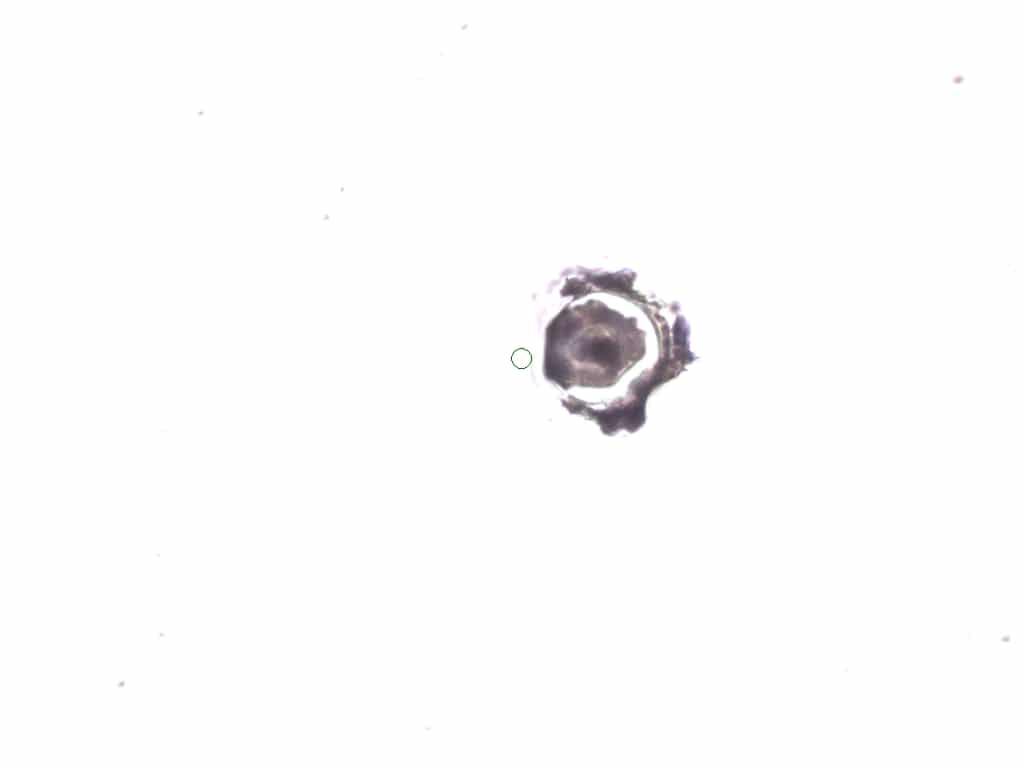

Supplement: Supplemental Material [file supp_gr.234807.118_Supplemental_File_4.zip › SINGLE CELL/DCIS single cell/DCIS-1 40x.jpeg]

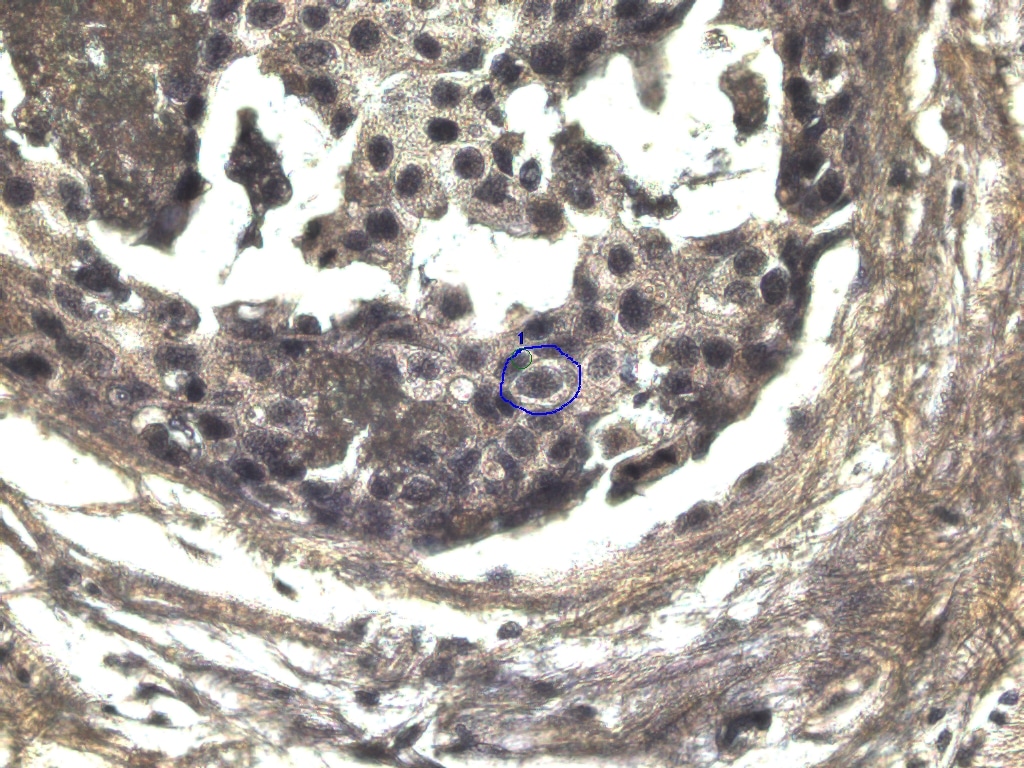

Supplement: Supplemental Material [file supp_gr.234807.118_Supplemental_File_4.zip › SINGLE CELL/DCIS single cell/DCIS-1 BEFORE.jpeg]

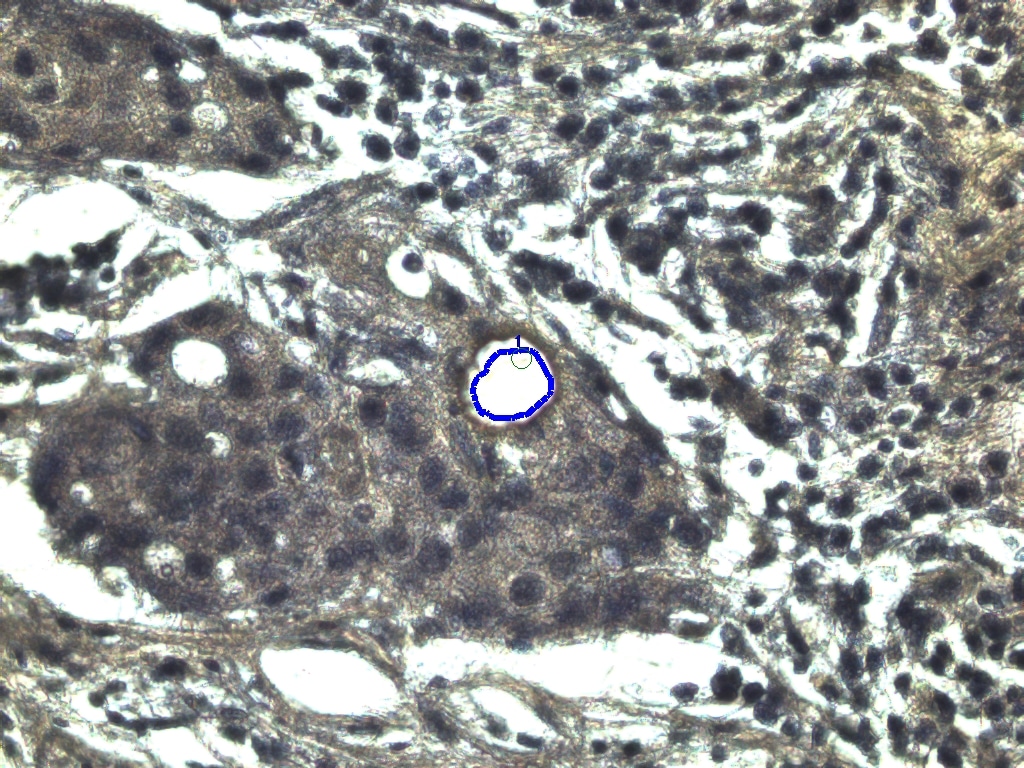

Supplement: Supplemental Material [file supp_gr.234807.118_Supplemental_File_4.zip › SINGLE CELL/DCIS single cell/DCIS-10 AFTER.jpeg]

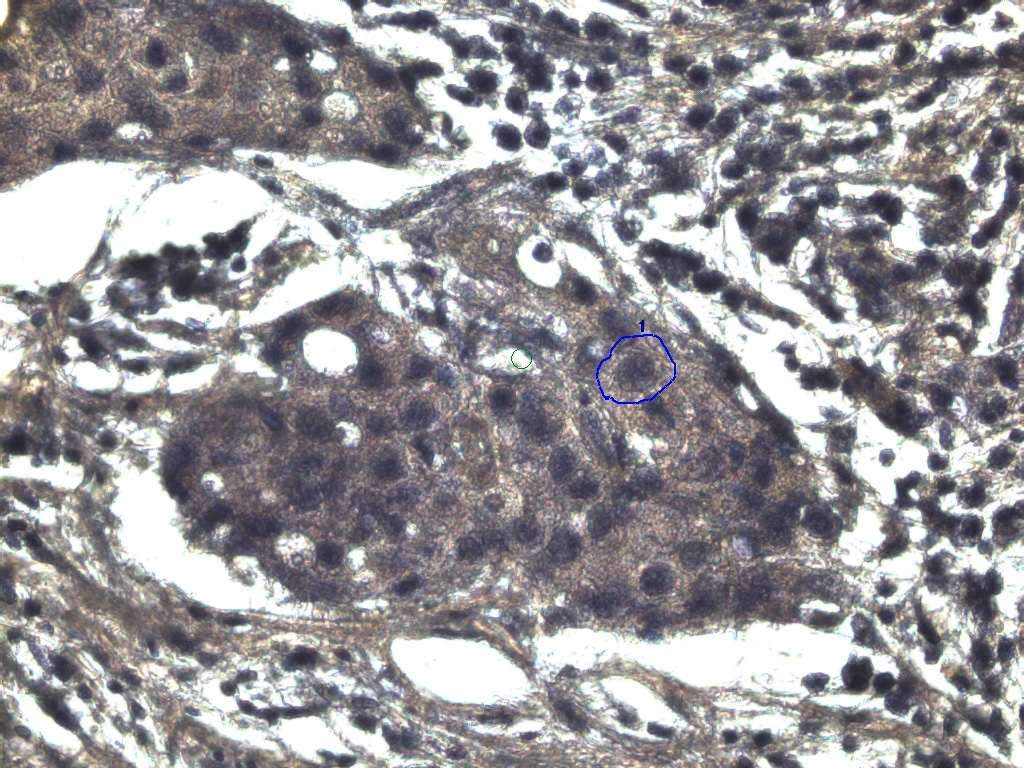

Supplement: Supplemental Material [file supp_gr.234807.118_Supplemental_File_4.zip › SINGLE CELL/DCIS single cell/DCIS-10 BEFORE.jpeg]

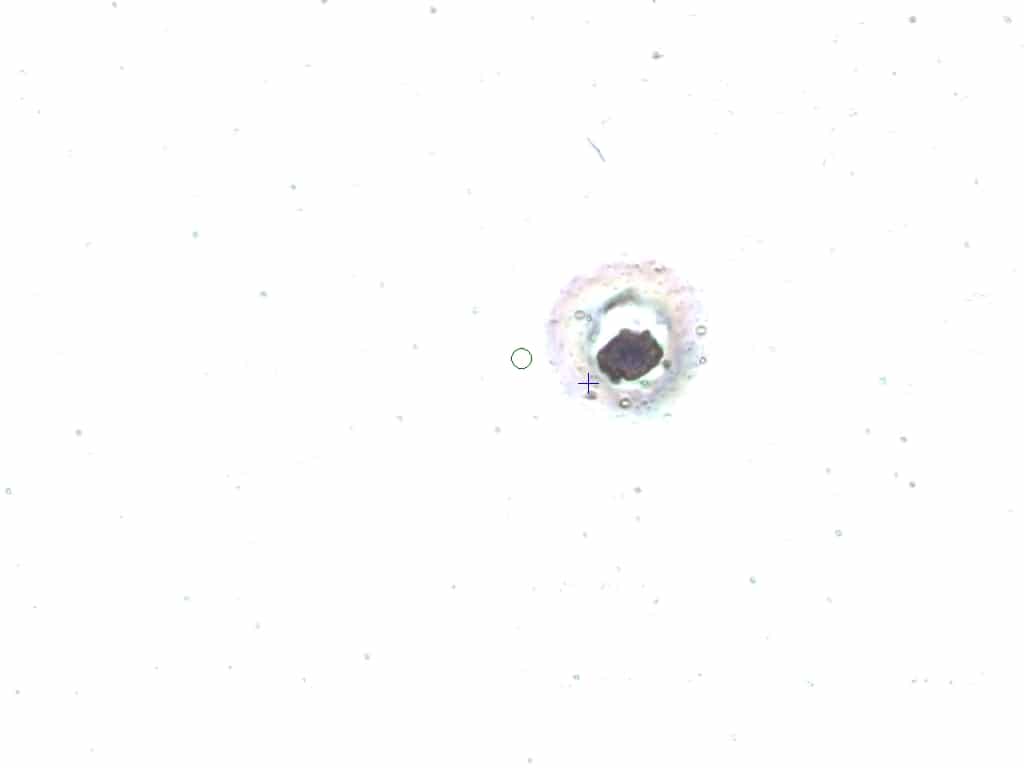

Supplement: Supplemental Material [file supp_gr.234807.118_Supplemental_File_4.zip › SINGLE CELL/DCIS single cell/DCIS-10.jpeg]

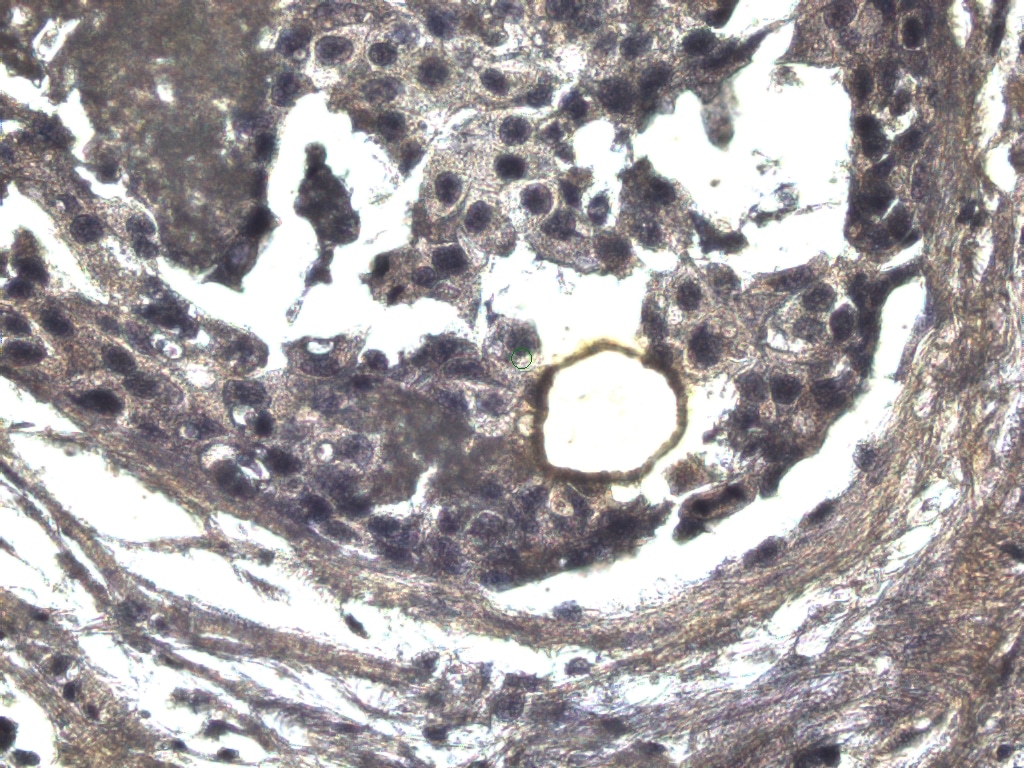

Supplement: Supplemental Material [file supp_gr.234807.118_Supplemental_File_4.zip › SINGLE CELL/DCIS single cell/DCIS-1AFTER40x.jpeg]

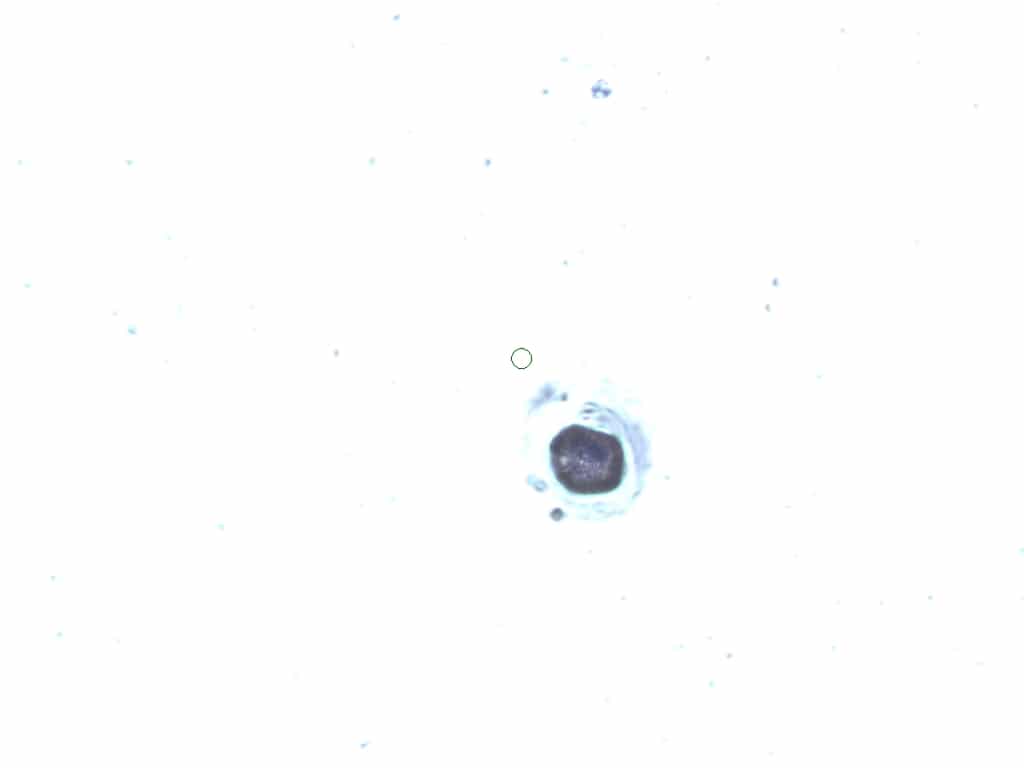

Supplement: Supplemental Material [file supp_gr.234807.118_Supplemental_File_4.zip › SINGLE CELL/DCIS single cell/DCIS-2 40x.jpeg]
