# Supplementary material for: Gene expression profiling of single cells from archival tissue with laser-capture microdissection and Smart-3SEQ
Source: Genome Res. 2019 Nov;29(11):1816–25. doi: 10.1101/gr.234807.118 (PMC6836736; doi:10.1101/gr.234807.118)
Supplement: Supplemental Material [file supp_gr.234807.118_Supplemental_Code.zip › Supplemental_Code.tar!/fig/depth_ref-rna.pdf]

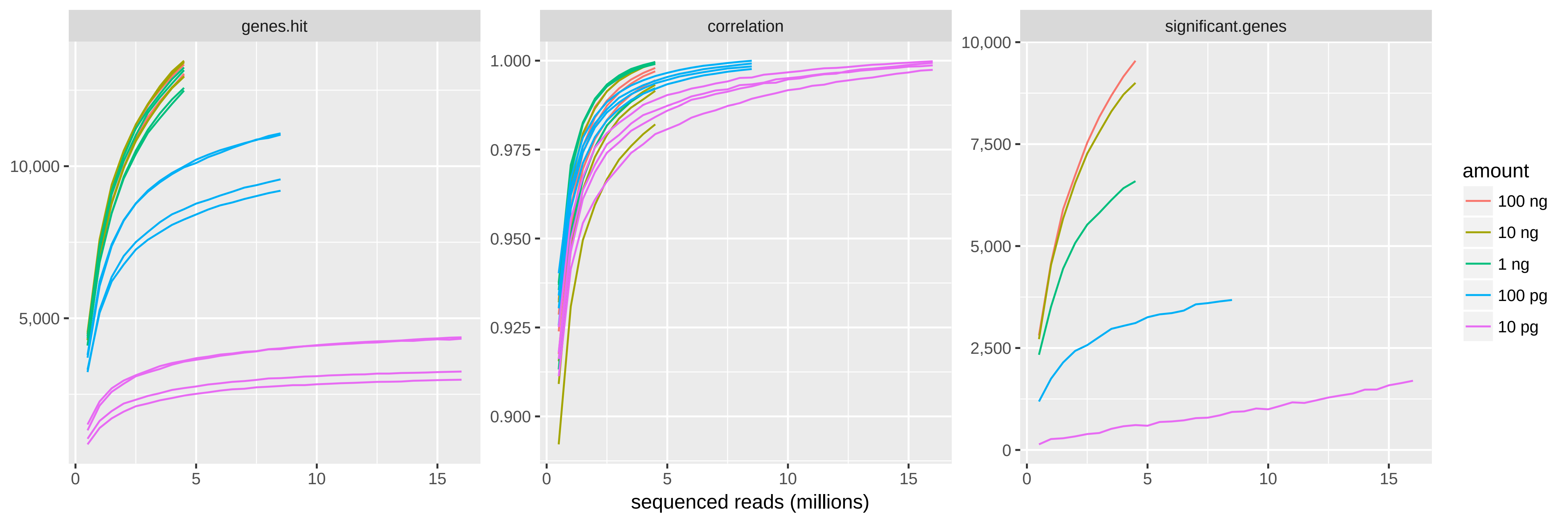
genes.hit
correlation
significant.genes
10,000
5,000
1.000
0.975
0.950
0.925
0.900
10,000
7,500
5,000
2,500
0
0
5
10
15
sequenced reads (millions)
amount
100 ng
10 ng
1 ng
100 pg
10 pg
