# Supplementary material for: Gene expression profiling of single cells from archival tissue with laser-capture microdissection and Smart-3SEQ
Source: Genome Res. 2019 Nov;29(11):1816–25. doi: 10.1101/gr.234807.118 (PMC6836736; doi:10.1101/gr.234807.118)
Supplement: Supplemental Material [file supp_gr.234807.118_Supplemental_Code.zip › Supplemental_Code.tar!/fig/alignability_ffpe-vs-frozen.pdf]

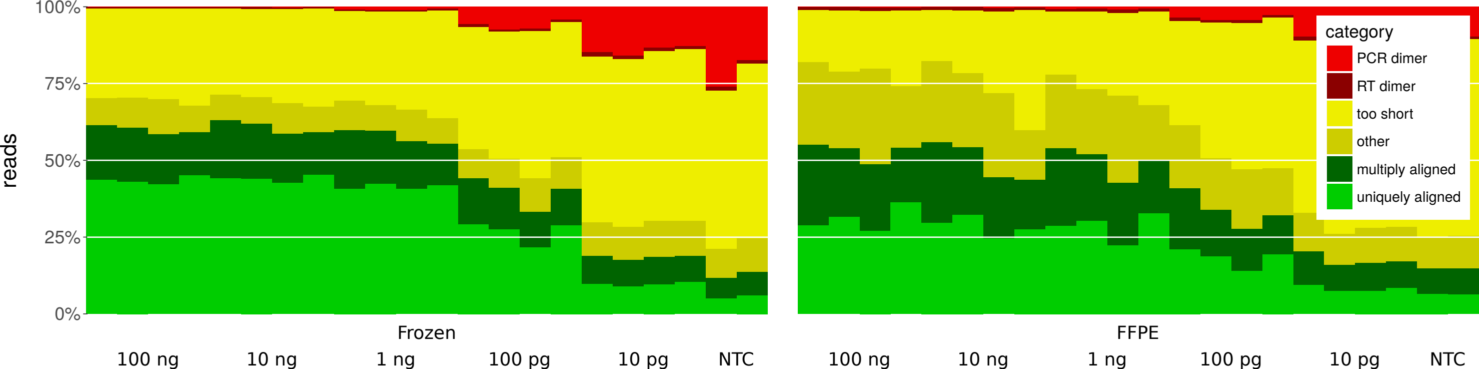

reads
100%
75%
50%
25%
0%
category
PCR dimer
RT dimer
too short
other
multiply aligned
uniquely aligned
Frozen
FFPE
100 ng
10 ng
1 ng
100 pg
10 pg
NTC
