# Supplementary material for: Gene expression profiling of single cells from archival tissue with laser-capture microdissection and Smart-3SEQ
Source: Genome Res. 2019 Nov;29(11):1816–25. doi: 10.1101/gr.234807.118 (PMC6836736; doi:10.1101/gr.234807.118)
Supplement: Supplemental Material [file supp_gr.234807.118_Supplemental_Code.zip › Supplemental_Code.tar!/fig/depth_ffpe-lcm.pdf]

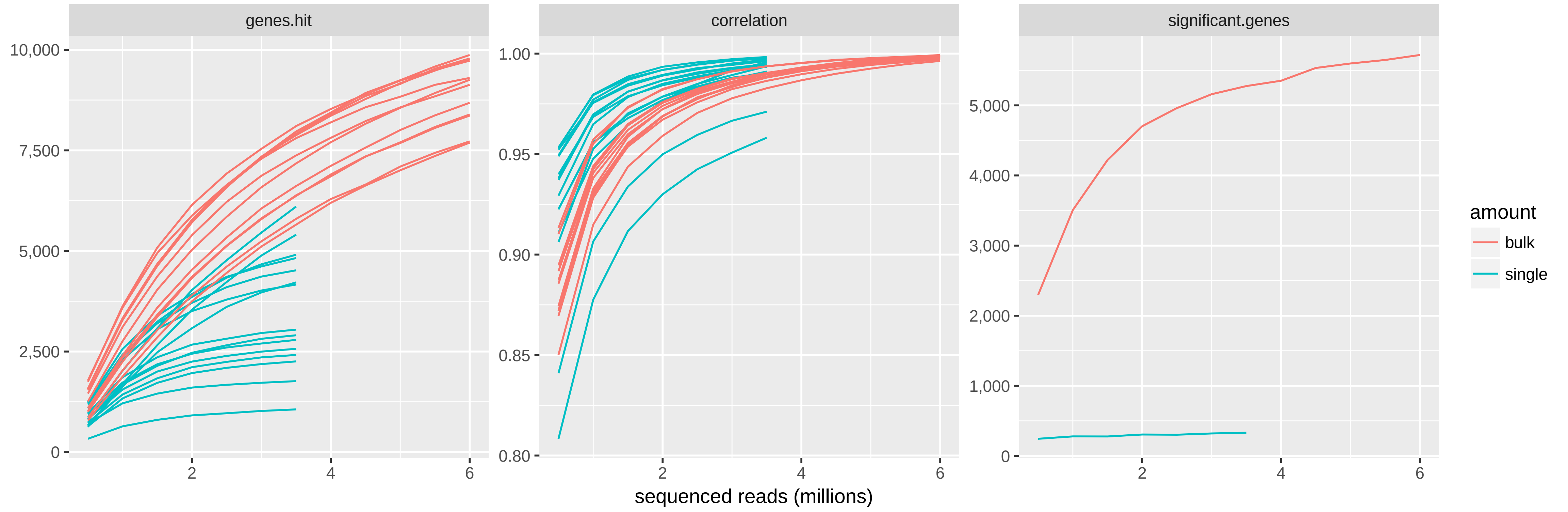

genes.hit
correlation
significant.genes
10,000
7,500
5,000
2,500
0
1.00
0.95
0.90
0.85
0.80
5,000
4,000
3,000
2,000
1,000
0
2
4
6
sequenced reads (millions)
amount
bulk
single
