# Supplementary material for: Gene expression profiling of single cells from archival tissue with laser-capture microdissection and Smart-3SEQ
Source: Genome Res. 2019 Nov;29(11):1816–25. doi: 10.1101/gr.234807.118 (PMC6836736; doi:10.1101/gr.234807.118)
Supplement: Supplemental Material [file supp_gr.234807.118_Supplemental_Code.zip › Supplemental_Code.tar!/fig/igv_snapshot_annotated.pdf]

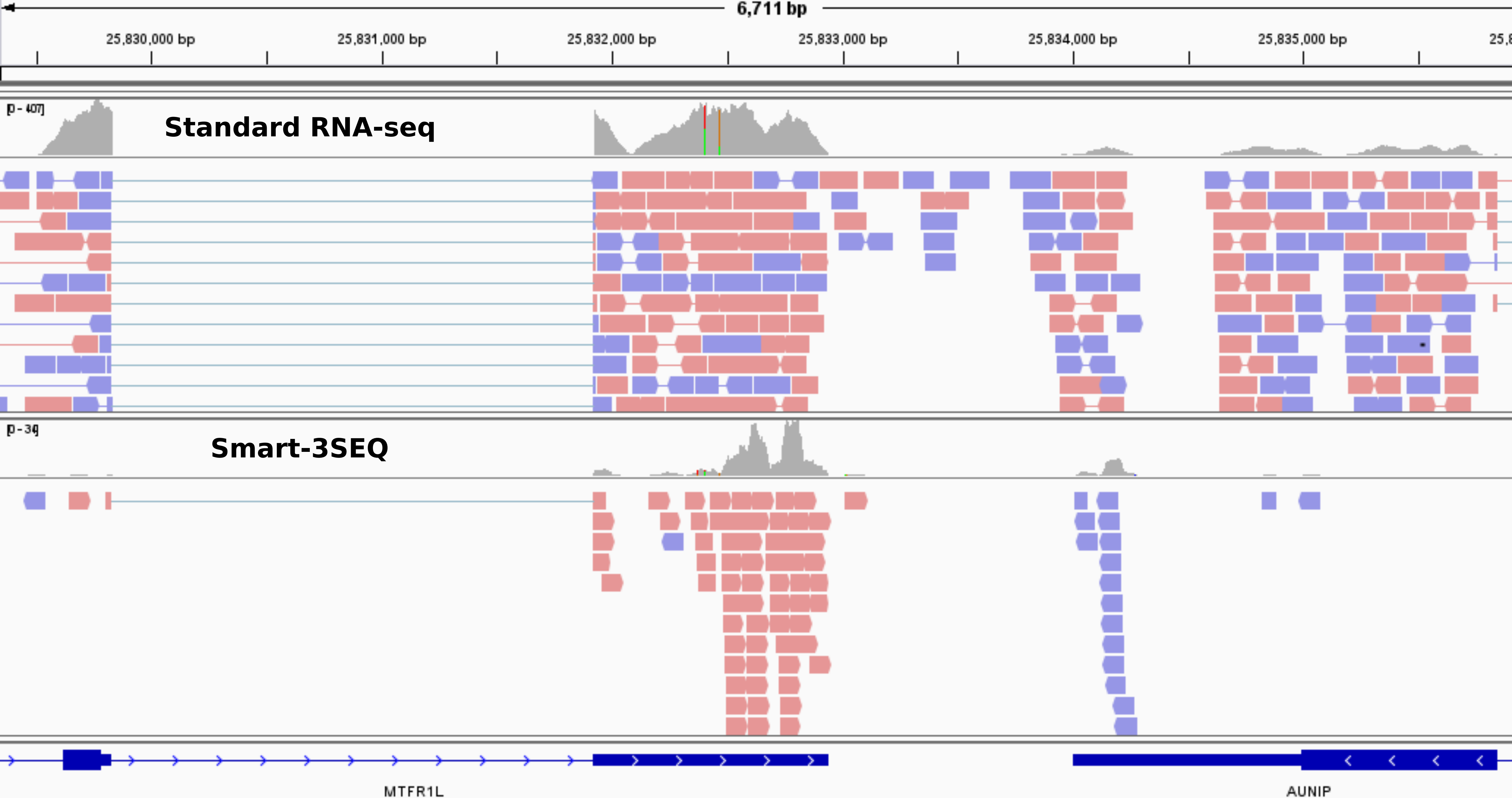

6,711 bp
25,830,000 bp
25,831,000 bp
25,832,000 bp
25,833,000 bp
25,834,000 bp
25,835,000 bp
[0 - 407]
Standard RNA-seq
[0 - 34]
Smart-3SEQ
MTFR1L
AUNIP
