# Supplementary material for: Gene expression profiling of single cells from archival tissue with laser-capture microdissection and Smart-3SEQ
Source: Genome Res. 2019 Nov;29(11):1816–25. doi: 10.1101/gr.234807.118 (PMC6836736; doi:10.1101/gr.234807.118)
Supplement: Supplemental Material [file supp_gr.234807.118_Supplemental_Code.zip › Supplemental_Code.tar!/fig/sensitivity_3seq.pdf]

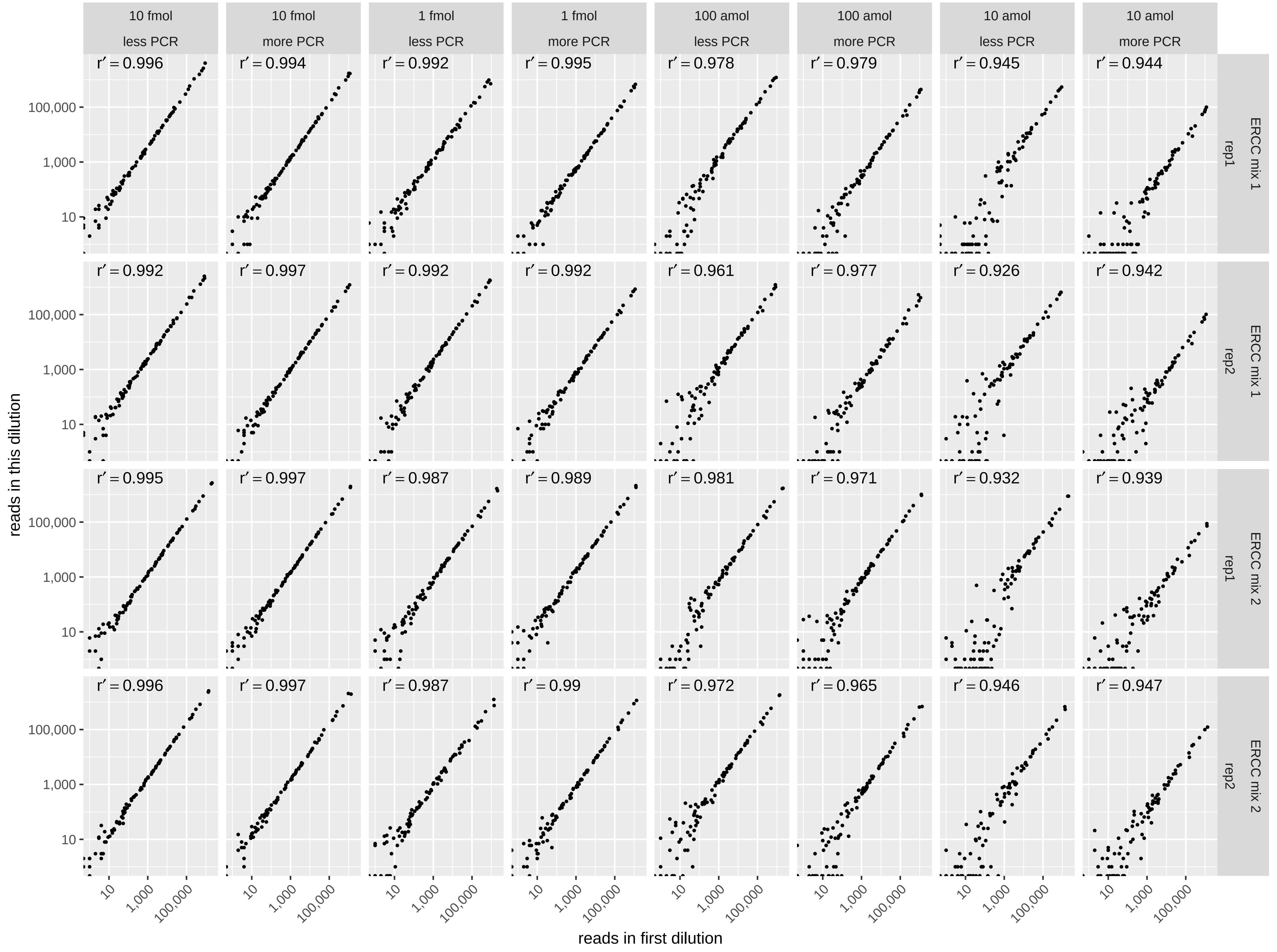

10 fmol less PCR
10 fmol more PCR
1 fmol less PCR
1 fmol more PCR
100 amol less PCR
100 amol more PCR
10 amol less PCR
10 amol more PCR
ERCC mix 1 rep1
ERCC mix 1 rep2
ERCC mix 2 rep1
ERCC mix 2 rep2
r′ = 0.996
r′ = 0.994
r′ = 0.992
r′ = 0.995
r′ = 0.978
r′ = 0.979
r′ = 0.945
r′ = 0.944
r′ = 0.992
r′ = 0.997
r′ = 0.992
r′ = 0.992
r′ = 0.961
r′ = 0.977
r′ = 0.926
r′ = 0.942
r′ = 0.995
r′ = 0.997
r′ = 0.987
r′ = 0.989
r′ = 0.981
r′ = 0.971
r′ = 0.932
r′ = 0.939
r′ = 0.996
r′ = 0.997
r′ = 0.987
r′ = 0.99
r′ = 0.972
r′ = 0.965
r′ = 0.946
r′ = 0.947
10
1,000
100,000
reads in this dilution
reads in first dilution
