# Supplementary material for: Gene expression profiling of single cells from archival tissue with laser-capture microdissection and Smart-3SEQ
Source: Genome Res. 2019 Nov;29(11):1816–25. doi: 10.1101/gr.234807.118 (PMC6836736; doi:10.1101/gr.234807.118)
Supplement: Supplemental Material [file supp_gr.234807.118_Supplemental_Code.zip › Supplemental_Code.tar!/fig/compare_3seq_sybr.pdf]

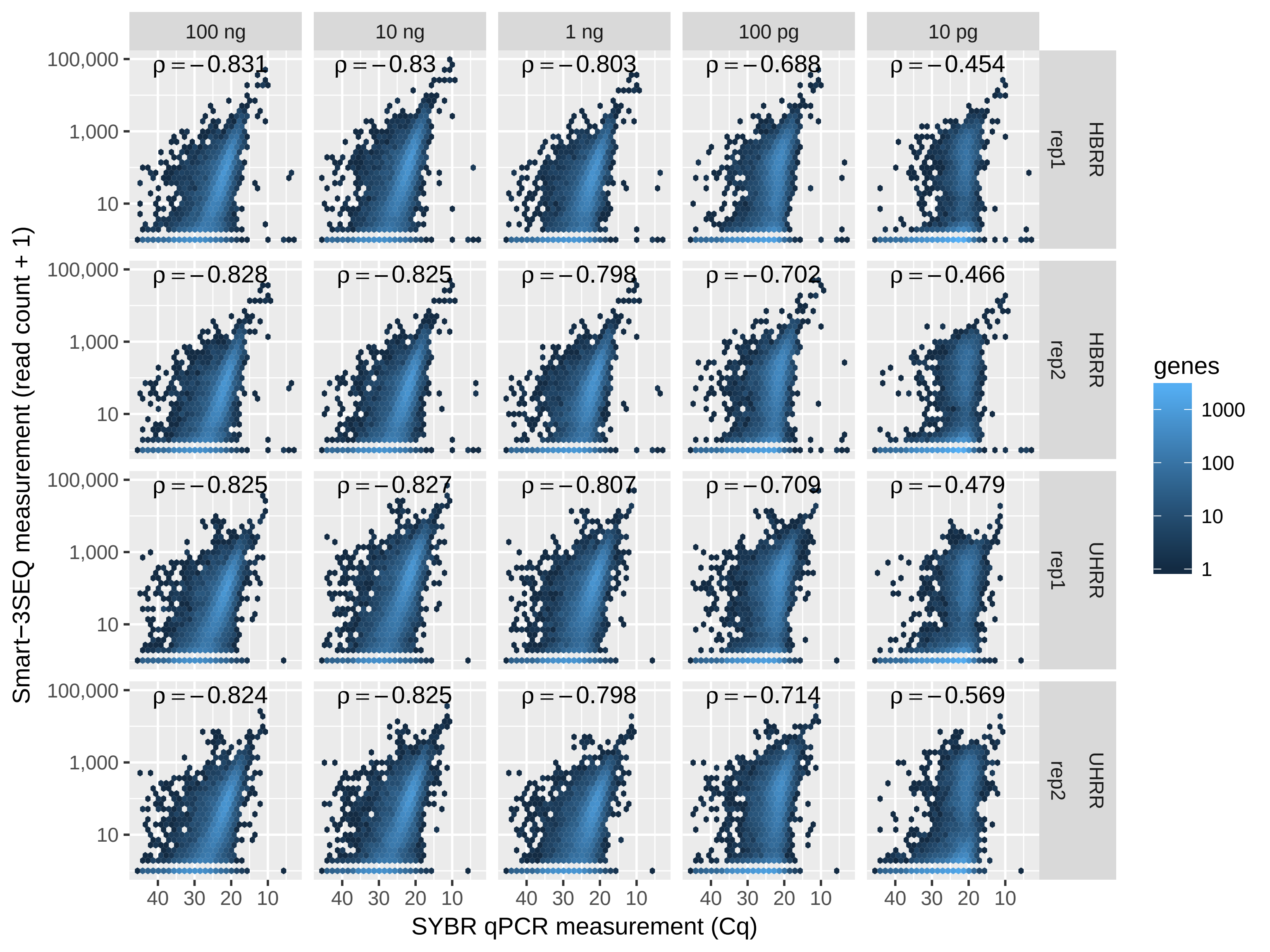
100 ng
10 ng
1 ng
100 pg
10 pg
HBRR rep1
HBRR rep2
UHRR rep1
UHRR rep2
ρ = −0.831
ρ = −0.83
ρ = −0.803
ρ = −0.688
ρ = −0.454
ρ = −0.828
ρ = −0.825
ρ = −0.798
ρ = −0.702
ρ = −0.466
ρ = −0.825
ρ = −0.827
ρ = −0.807
ρ = −0.709
ρ = −0.479
ρ = −0.824
ρ = −0.825
ρ = −0.798
ρ = −0.714
ρ = −0.569
100,000
1,000
10
40
30
20
10
Smart−3SEQ measurement (read count + 1)
SYBR qPCR measurement (Cq)
genes
1000
100
10
1
