# Supplementary material for: Gene expression profiling of single cells from archival tissue with laser-capture microdissection and Smart-3SEQ
Source: Genome Res. 2019 Nov;29(11):1816–25. doi: 10.1101/gr.234807.118 (PMC6836736; doi:10.1101/gr.234807.118)
Supplement: Supplemental Material [file supp_gr.234807.118_Supplemental_Code.zip › Supplemental_Code.tar!/fig/alignability_ercc.pdf]

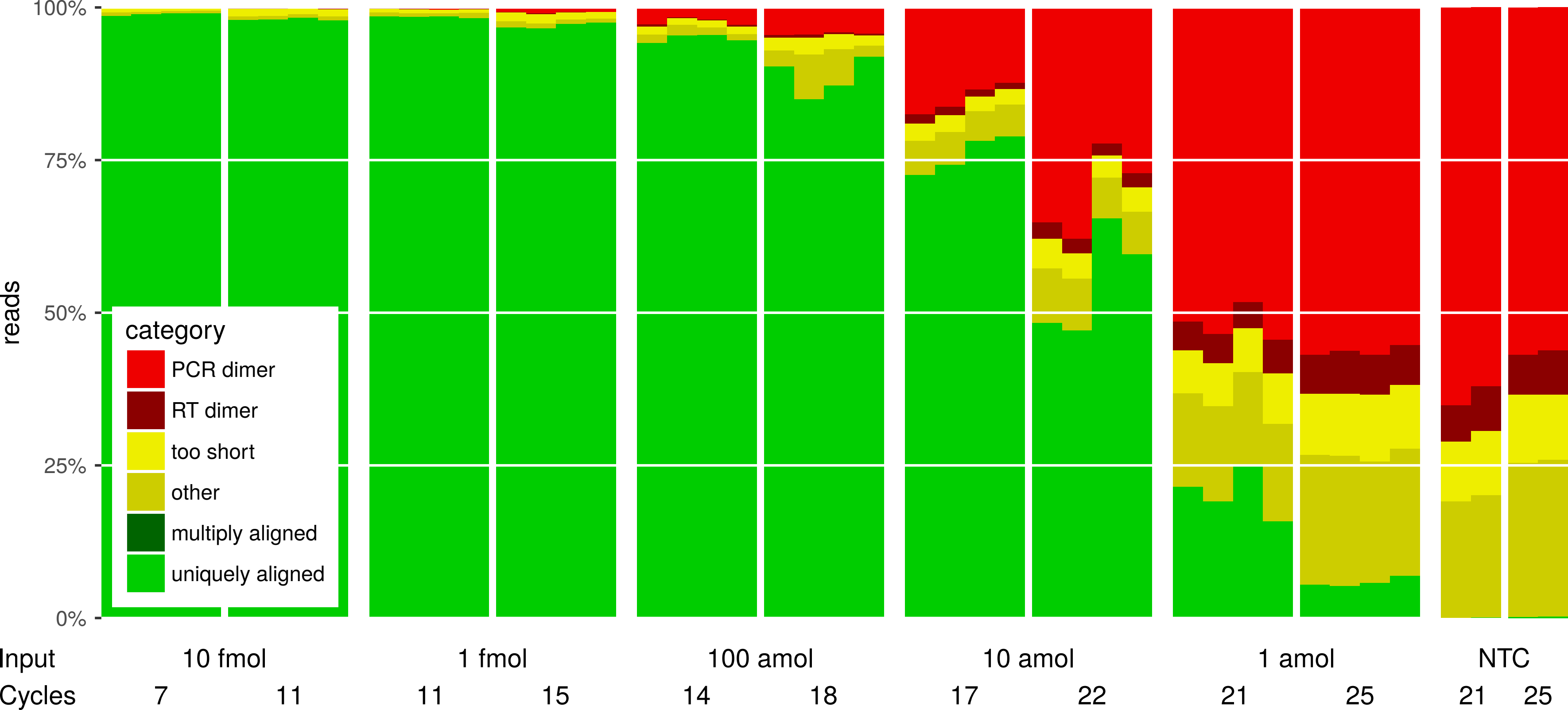
100%
75%
50%
25%
0%
reads
category
PCR dimer
RT dimer
too short
other
multiply aligned
uniquely aligned
Input
10 fmol
1 fmol
100 amol
10 amol
1 amol
NTC
Cycles
7
11
11
15
14
18
17
22
21
25
21
25
