# Supplementary material for: Gene expression profiling of single cells from archival tissue with laser-capture microdissection and Smart-3SEQ
Source: Genome Res. 2019 Nov;29(11):1816–25. doi: 10.1101/gr.234807.118 (PMC6836736; doi:10.1101/gr.234807.118)
Supplement: Supplemental Material [file supp_gr.234807.118_Supplemental_Code.zip › Supplemental_Code.tar!/fig/ffpe-lcm_heatmap_by_chr.pdf]

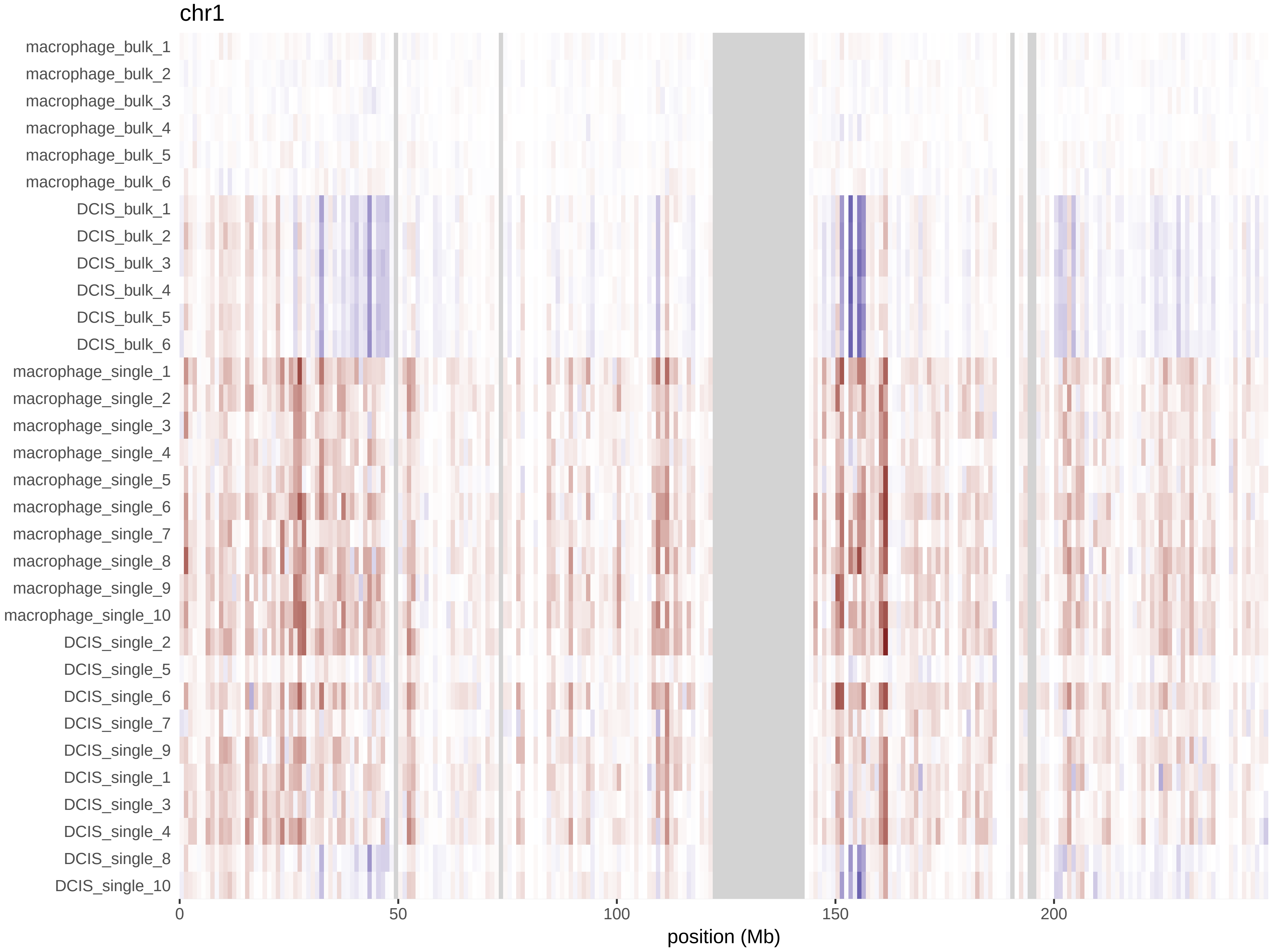
chr1
macrophage_bulk_1
macrophage_bulk_2
macrophage_bulk_3
macrophage_bulk_4
macrophage_bulk_5
macrophage_bulk_6
DCIS_bulk_1
DCIS_bulk_2
DCIS_bulk_3
DCIS_bulk_4
DCIS_bulk_5
DCIS_bulk_6
macrophage_single_1
macrophage_single_2
macrophage_single_3
macrophage_single_4
macrophage_single_5
macrophage_single_6
macrophage_single_7
macrophage_single_8
macrophage_single_9
macrophage_single_10
DCIS_single_2
DCIS_single_5
DCIS_single_6
DCIS_single_7
DCIS_single_9
DCIS_single_1
DCIS_single_3
DCIS_single_4
DCIS_single_8
DCIS_single_10
0
50
100
150
200
position (Mb)

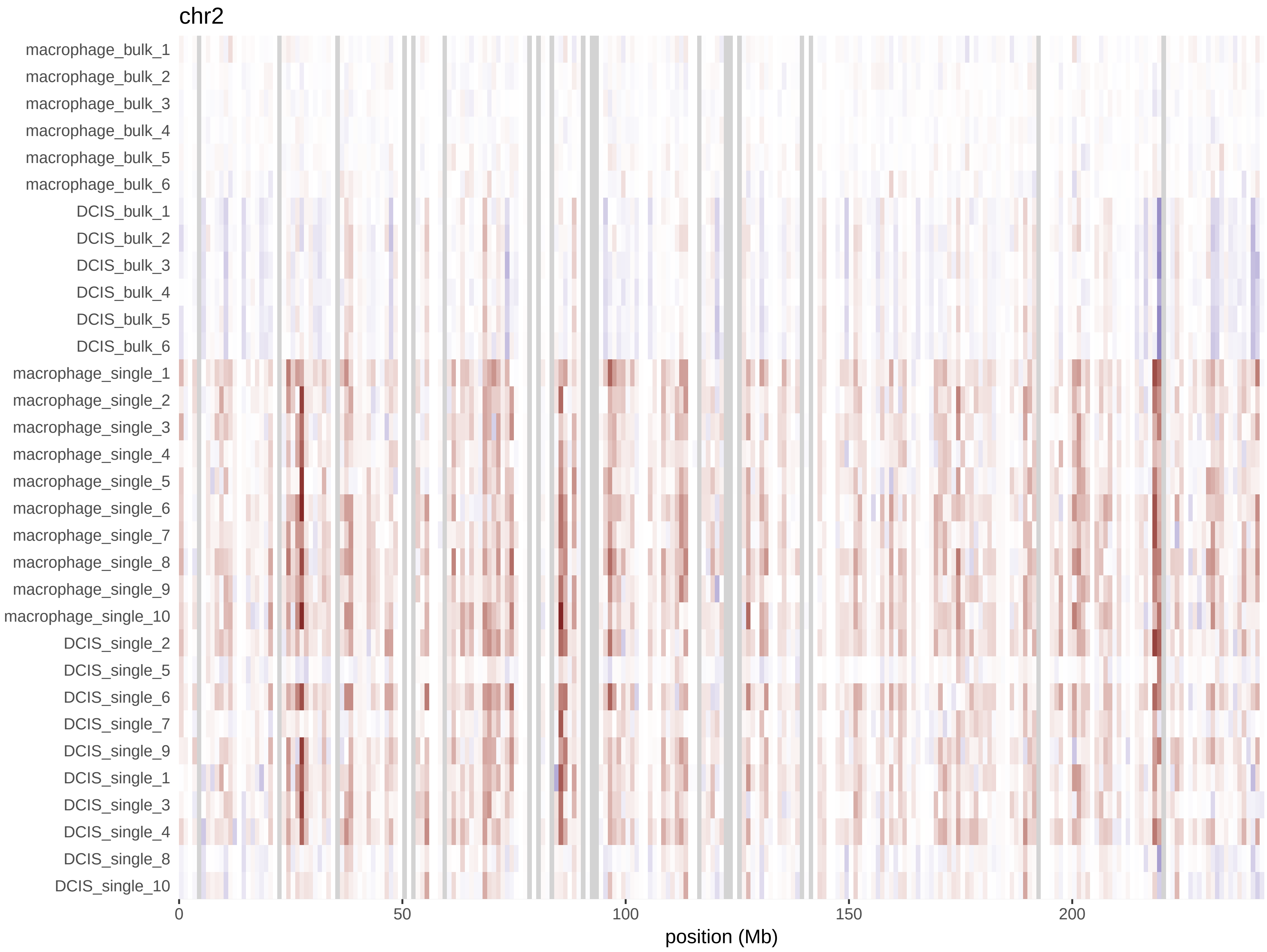
chr2
macrophage_bulk_1
macrophage_bulk_2
macrophage_bulk_3
macrophage_bulk_4
macrophage_bulk_5
macrophage_bulk_6
DCIS_bulk_1
DCIS_bulk_2
DCIS_bulk_3
DCIS_bulk_4
DCIS_bulk_5
DCIS_bulk_6
macrophage_single_1
macrophage_single_2
macrophage_single_3
macrophage_single_4
macrophage_single_5
macrophage_single_6
macrophage_single_7
macrophage_single_8
macrophage_single_9
macrophage_single_10
DCIS_single_2
DCIS_single_5
DCIS_single_6
DCIS_single_7
DCIS_single_9
DCIS_single_1
DCIS_single_3
DCIS_single_4
DCIS_single_8
DCIS_single_10
0
50
100
150
200
position (Mb)

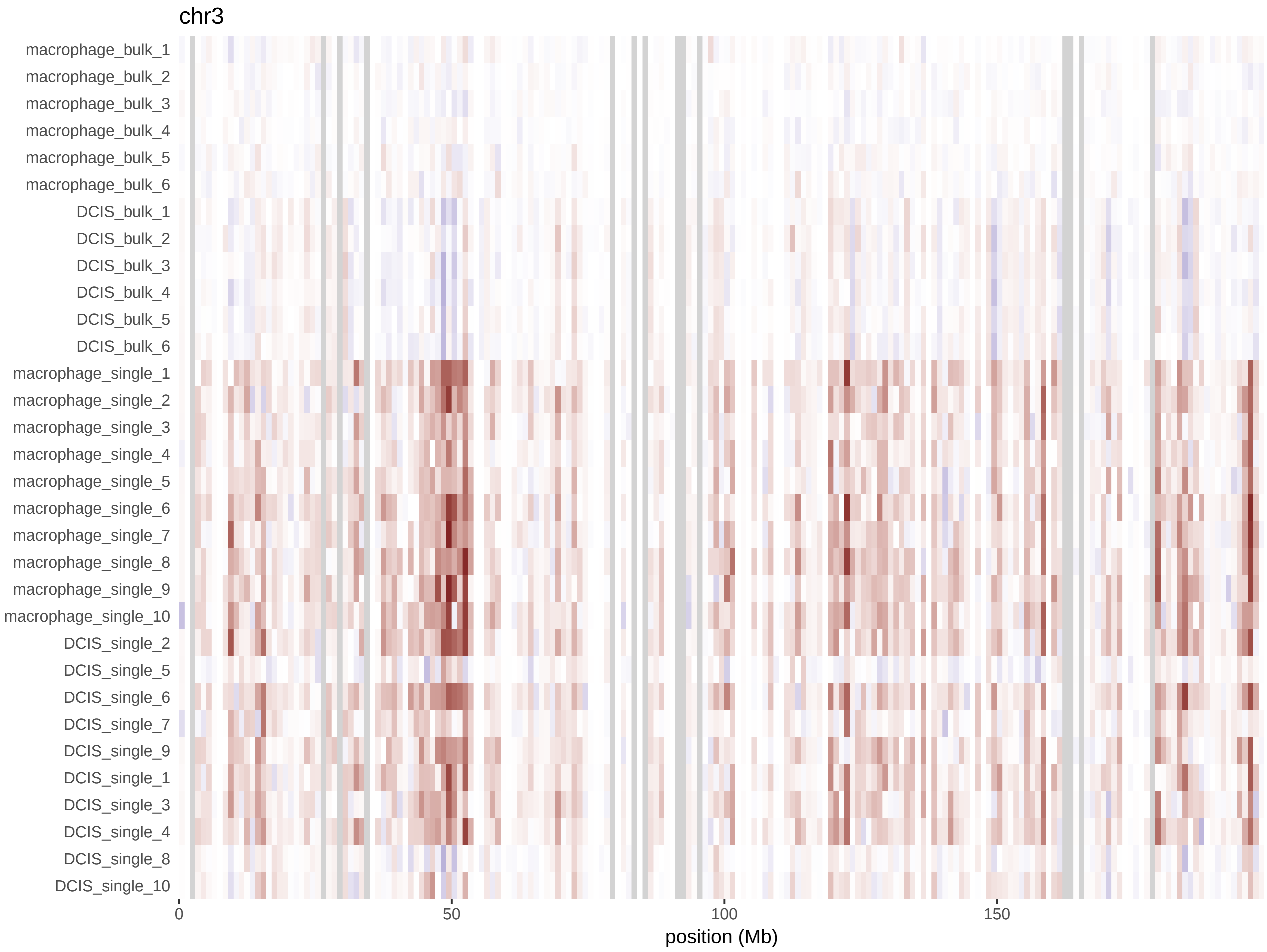

chr3
macrophage_bulk_1
macrophage_bulk_2
macrophage_bulk_3
macrophage_bulk_4
macrophage_bulk_5
macrophage_bulk_6
DCIS_bulk_1
DCIS_bulk_2
DCIS_bulk_3
DCIS_bulk_4
DCIS_bulk_5
DCIS_bulk_6
macrophage_single_1
macrophage_single_2
macrophage_single_3
macrophage_single_4
macrophage_single_5
macrophage_single_6
macrophage_single_7
macrophage_single_8
macrophage_single_9
macrophage_single_10
DCIS_single_2
DCIS_single_5
DCIS_single_6
DCIS_single_7
DCIS_single_9
DCIS_single_1
DCIS_single_3
DCIS_single_4
DCIS_single_8
DCIS_single_10
0
50
100
150
position (Mb)

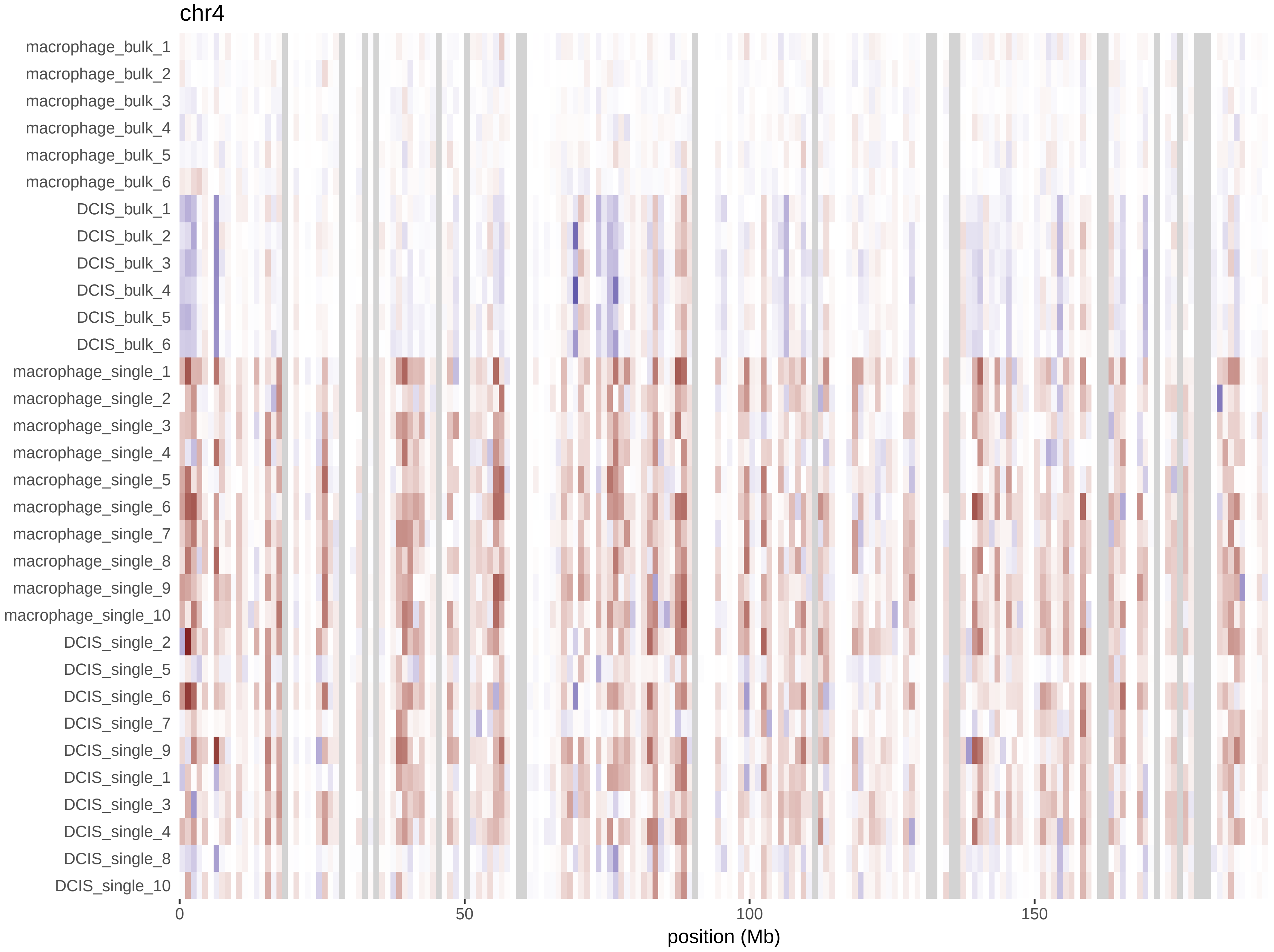
chr4
macrophage_bulk_1
macrophage_bulk_2
macrophage_bulk_3
macrophage_bulk_4
macrophage_bulk_5
macrophage_bulk_6
DCIS_bulk_1
DCIS_bulk_2
DCIS_bulk_3
DCIS_bulk_4
DCIS_bulk_5
DCIS_bulk_6
macrophage_single_1
macrophage_single_2
macrophage_single_3
macrophage_single_4
macrophage_single_5
macrophage_single_6
macrophage_single_7
macrophage_single_8
macrophage_single_9
macrophage_single_10
DCIS_single_2
DCIS_single_5
DCIS_single_6
DCIS_single_7
DCIS_single_9
DCIS_single_1
DCIS_single_3
DCIS_single_4
DCIS_single_8
DCIS_single_10
0
50
100
150
position (Mb)

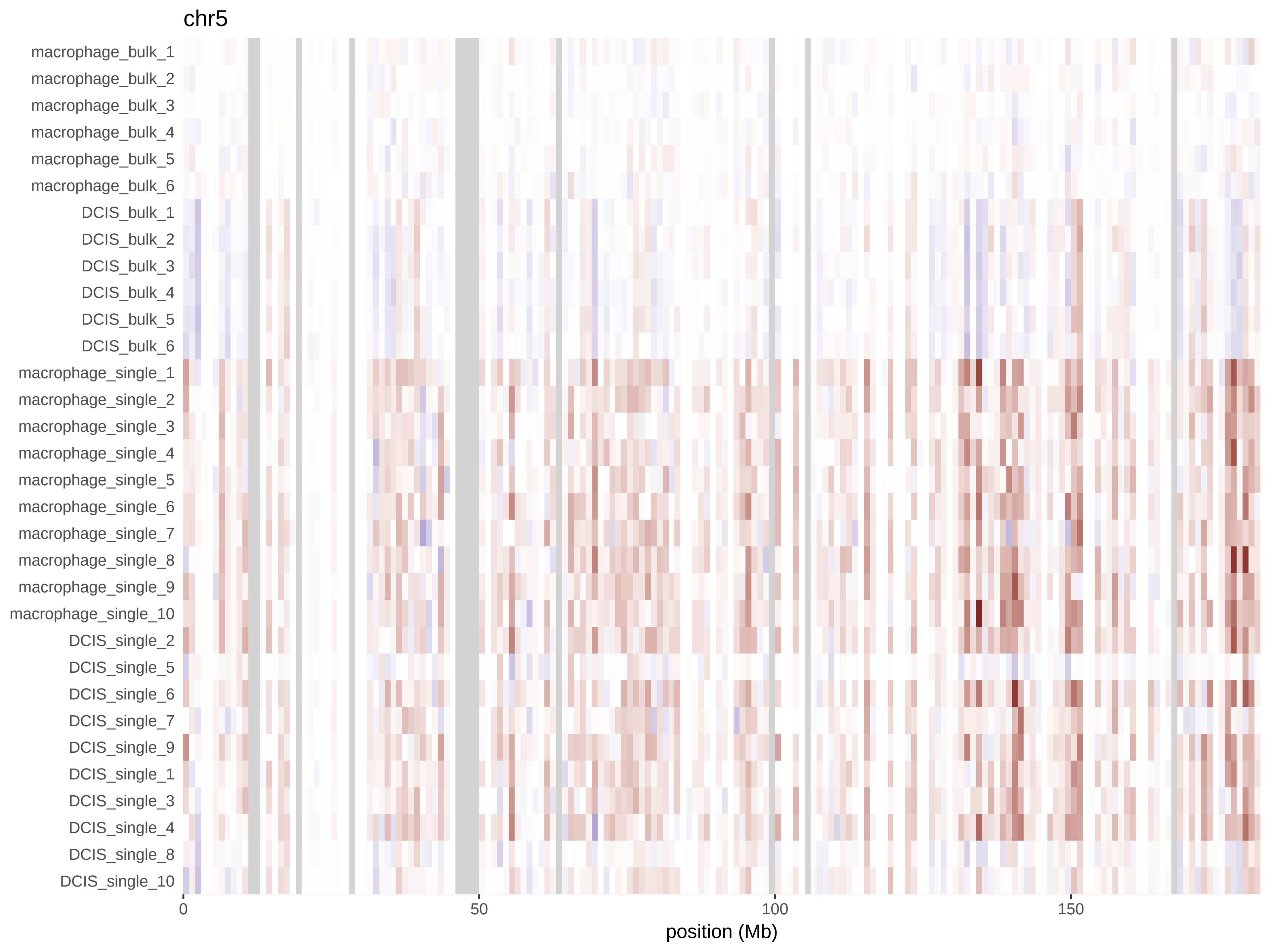

chr5
macrophage_bulk_1
macrophage_bulk_2
macrophage_bulk_3
macrophage_bulk_4
macrophage_bulk_5
macrophage_bulk_6
DCIS_bulk_1
DCIS_bulk_2
DCIS_bulk_3
DCIS_bulk_4
DCIS_bulk_5
DCIS_bulk_6
macrophage_single_1
macrophage_single_2
macrophage_single_3
macrophage_single_4
macrophage_single_5
macrophage_single_6
macrophage_single_7
macrophage_single_8
macrophage_single_9
macrophage_single_10
DCIS_single_2
DCIS_single_5
DCIS_single_6
DCIS_single_7
DCIS_single_9
DCIS_single_1
DCIS_single_3
DCIS_single_4
DCIS_single_8
DCIS_single_10
0
50
100
150
position (Mb)

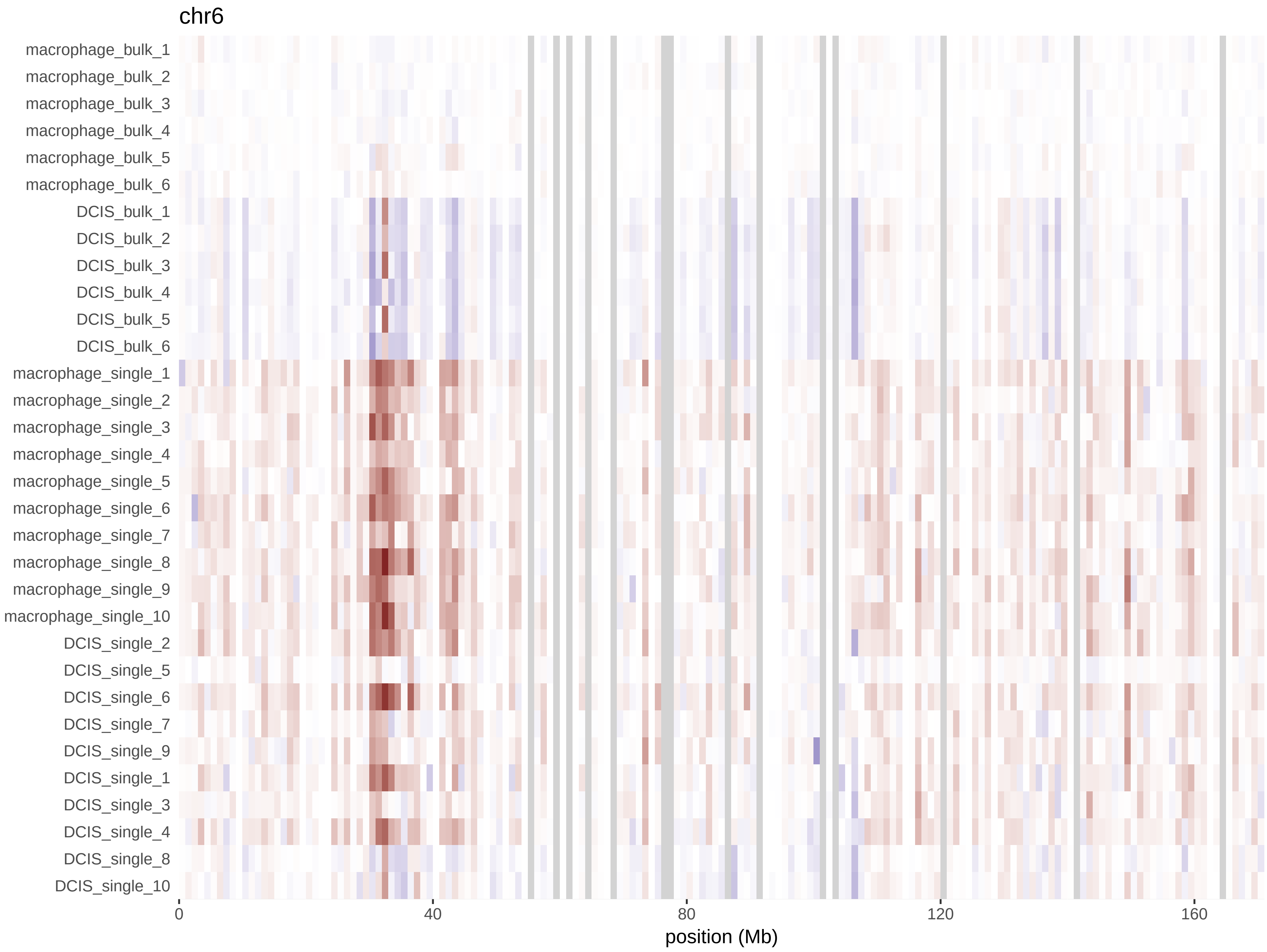

chr6
macrophage_bulk_1
macrophage_bulk_2
macrophage_bulk_3
macrophage_bulk_4
macrophage_bulk_5
macrophage_bulk_6
DCIS_bulk_1
DCIS_bulk_2
DCIS_bulk_3
DCIS_bulk_4
DCIS_bulk_5
DCIS_bulk_6
macrophage_single_1
macrophage_single_2
macrophage_single_3
macrophage_single_4
macrophage_single_5
macrophage_single_6
macrophage_single_7
macrophage_single_8
macrophage_single_9
macrophage_single_10
DCIS_single_2
DCIS_single_5
DCIS_single_6
DCIS_single_7
DCIS_single_9
DCIS_single_1
DCIS_single_3
DCIS_single_4
DCIS_single_8
DCIS_single_10
0
40
80
120
160
position (Mb)

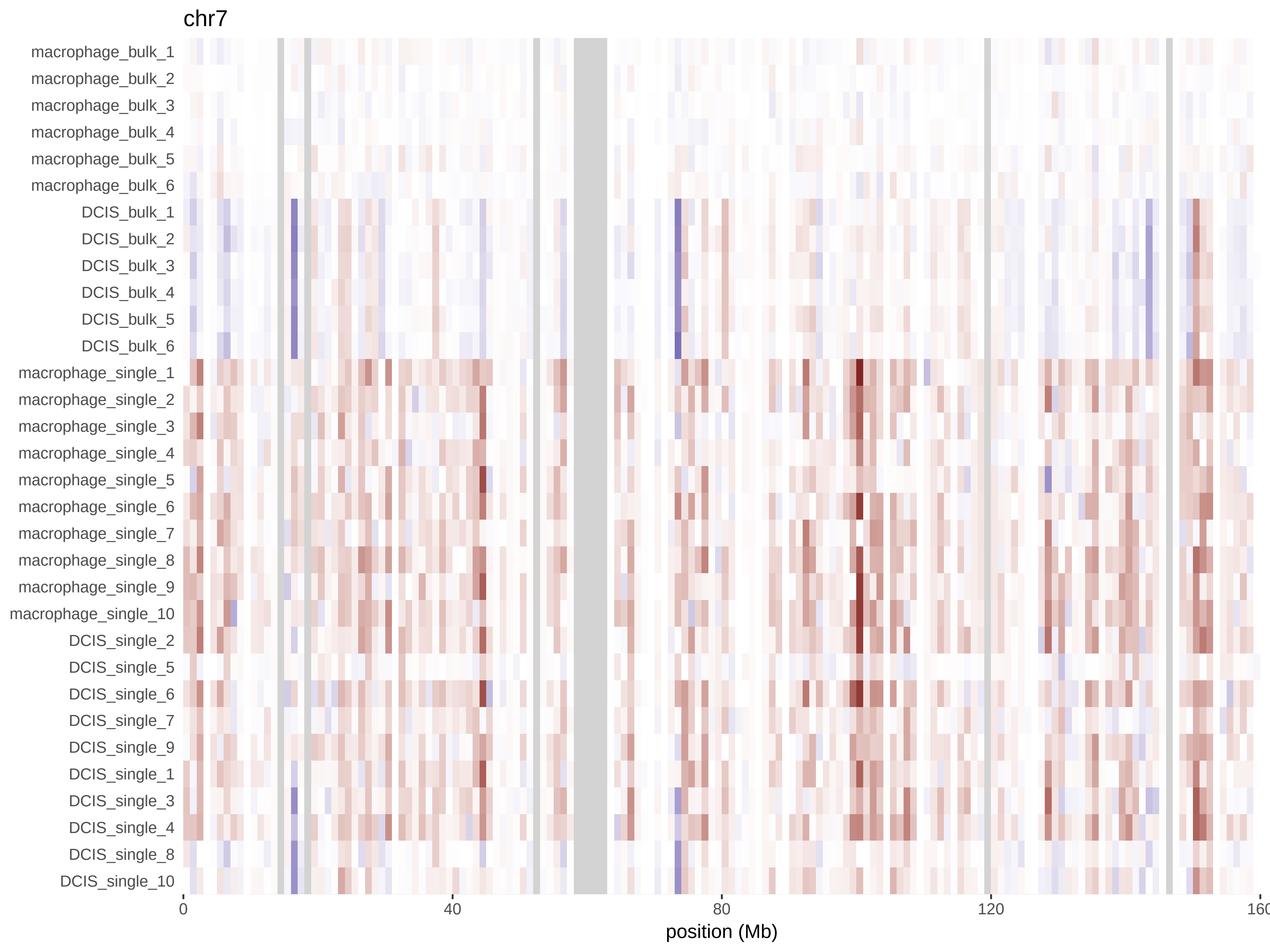

chr7
macrophage_bulk_1
macrophage_bulk_2
macrophage_bulk_3
macrophage_bulk_4
macrophage_bulk_5
macrophage_bulk_6
DCIS_bulk_1
DCIS_bulk_2
DCIS_bulk_3
DCIS_bulk_4
DCIS_bulk_5
DCIS_bulk_6
macrophage_single_1
macrophage_single_2
macrophage_single_3
macrophage_single_4
macrophage_single_5
macrophage_single_6
macrophage_single_7
macrophage_single_8
macrophage_single_9
macrophage_single_10
DCIS_single_2
DCIS_single_5
DCIS_single_6
DCIS_single_7
DCIS_single_9
DCIS_single_1
DCIS_single_3
DCIS_single_4
DCIS_single_8
DCIS_single_10
0
40
80
120
160
position (Mb)

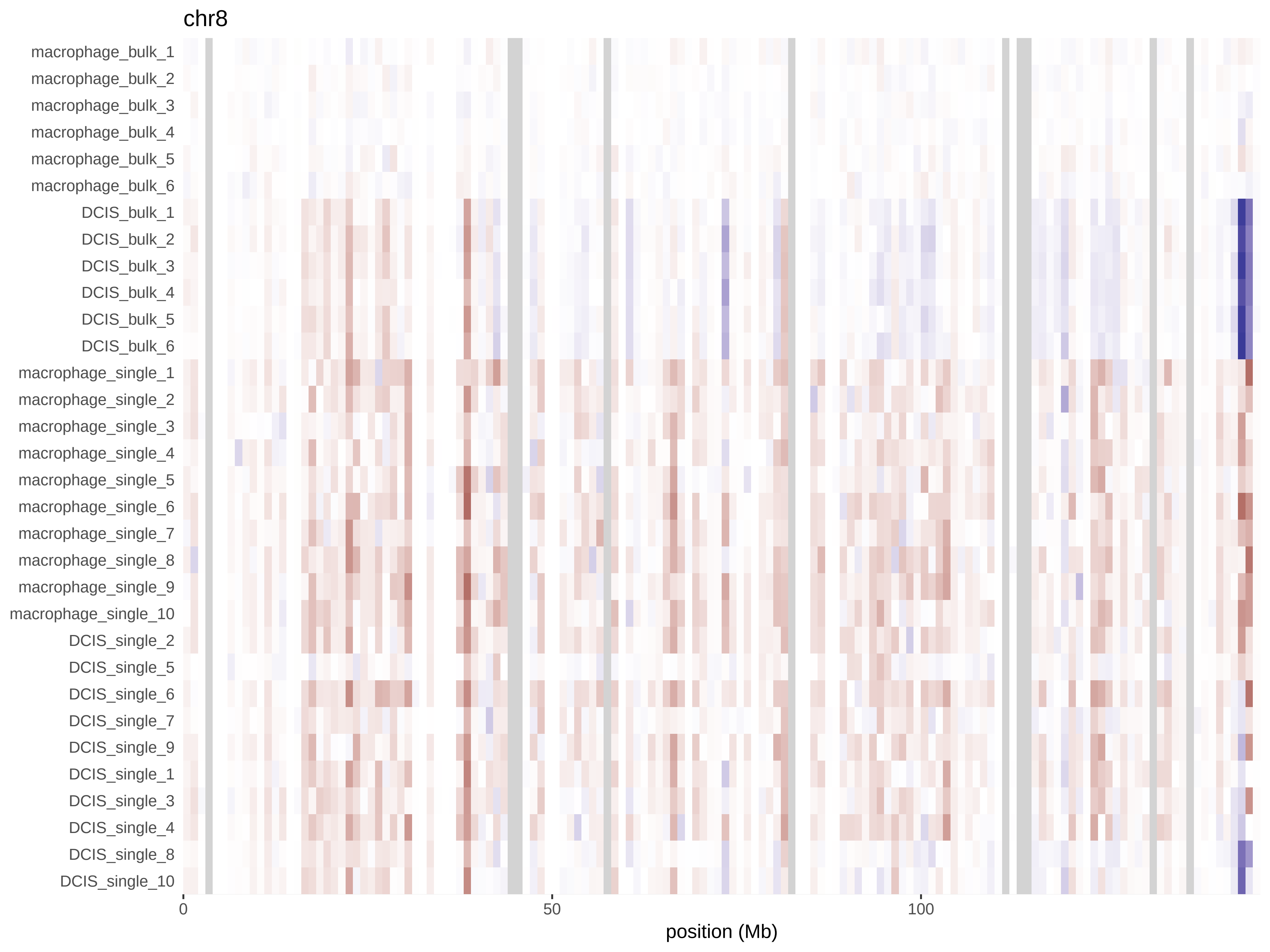

chr8
macrophage_bulk_1
macrophage_bulk_2
macrophage_bulk_3
macrophage_bulk_4
macrophage_bulk_5
macrophage_bulk_6
DCIS_bulk_1
DCIS_bulk_2
DCIS_bulk_3
DCIS_bulk_4
DCIS_bulk_5
DCIS_bulk_6
macrophage_single_1
macrophage_single_2
macrophage_single_3
macrophage_single_4
macrophage_single_5
macrophage_single_6
macrophage_single_7
macrophage_single_8
macrophage_single_9
macrophage_single_10
DCIS_single_2
DCIS_single_5
DCIS_single_6
DCIS_single_7
DCIS_single_9
DCIS_single_1
DCIS_single_3
DCIS_single_4
DCIS_single_8
DCIS_single_10
0
50
100
position (Mb)

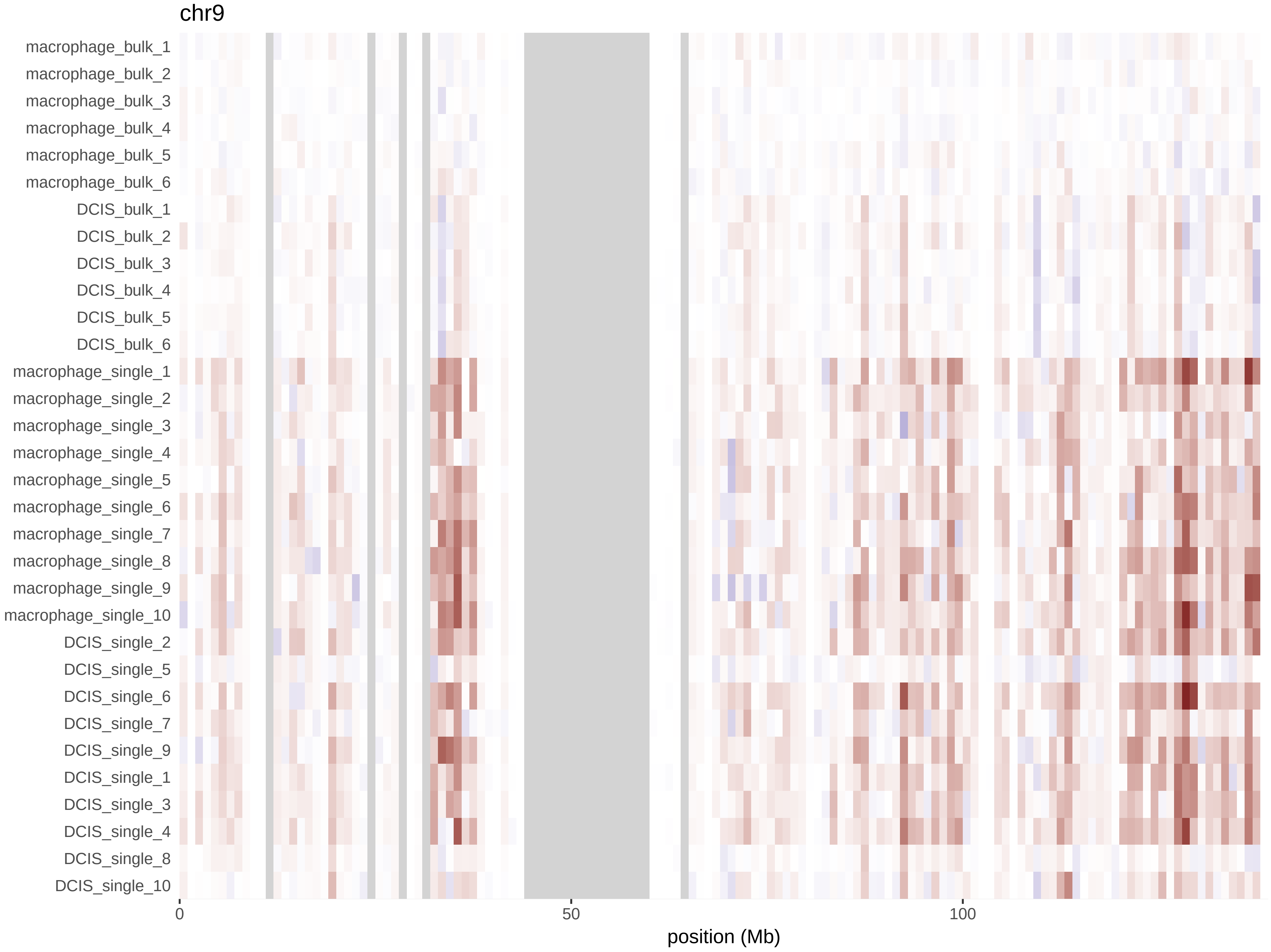
chr9
macrophage_bulk_1
macrophage_bulk_2
macrophage_bulk_3
macrophage_bulk_4
macrophage_bulk_5
macrophage_bulk_6
DCIS_bulk_1
DCIS_bulk_2
DCIS_bulk_3
DCIS_bulk_4
DCIS_bulk_5
DCIS_bulk_6
macrophage_single_1
macrophage_single_2
macrophage_single_3
macrophage_single_4
macrophage_single_5
macrophage_single_6
macrophage_single_7
macrophage_single_8
macrophage_single_9
macrophage_single_10
DCIS_single_2
DCIS_single_5
DCIS_single_6
DCIS_single_7
DCIS_single_9
DCIS_single_1
DCIS_single_3
DCIS_single_4
DCIS_single_8
DCIS_single_10
0
50
100
position (Mb)

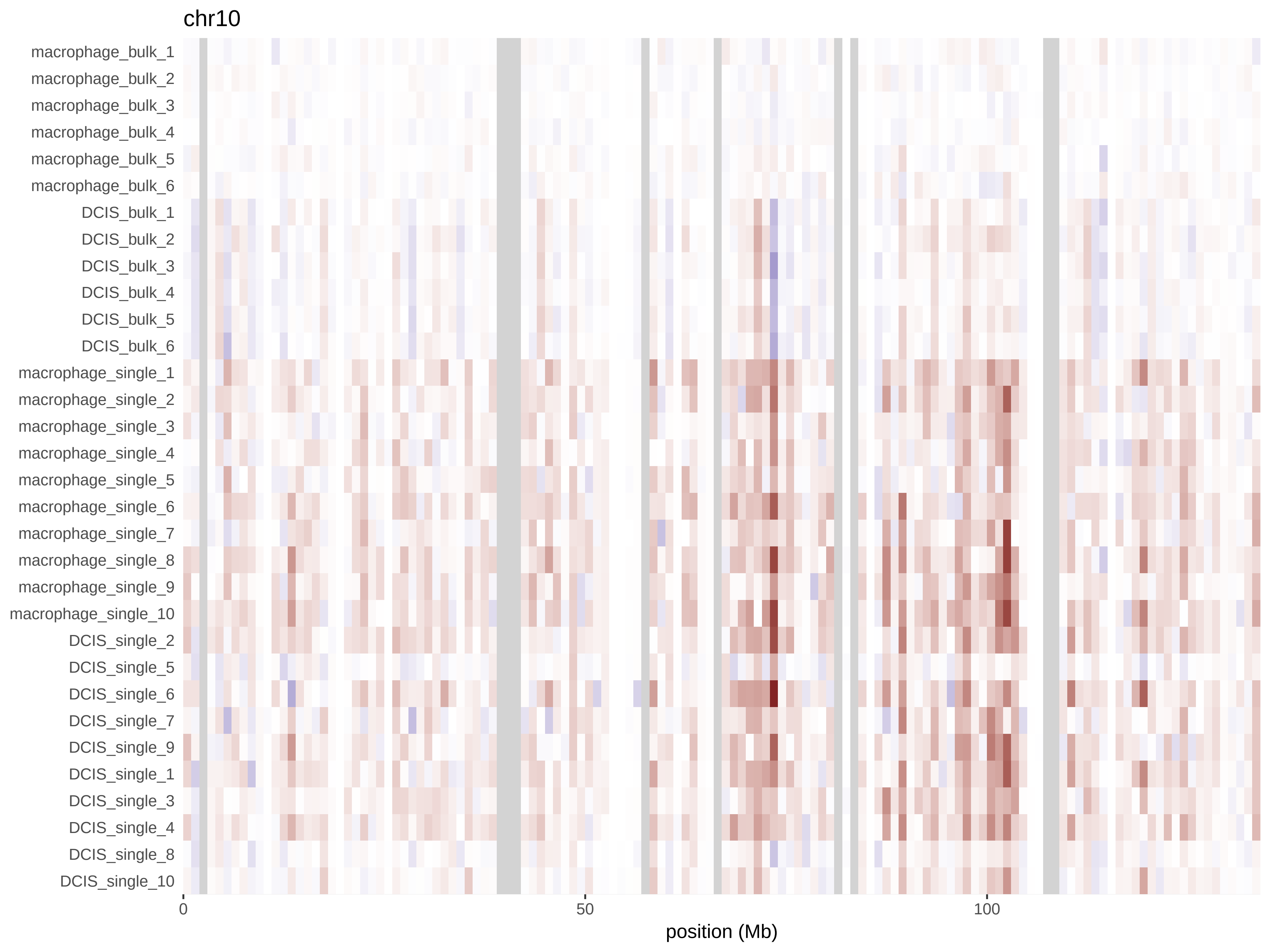
chr10
macrophage_bulk_1
macrophage_bulk_2
macrophage_bulk_3
macrophage_bulk_4
macrophage_bulk_5
macrophage_bulk_6
DCIS_bulk_1
DCIS_bulk_2
DCIS_bulk_3
DCIS_bulk_4
DCIS_bulk_5
DCIS_bulk_6
macrophage_single_1
macrophage_single_2
macrophage_single_3
macrophage_single_4
macrophage_single_5
macrophage_single_6
macrophage_single_7
macrophage_single_8
macrophage_single_9
macrophage_single_10
DCIS_single_2
DCIS_single_5
DCIS_single_6
DCIS_single_7
DCIS_single_9
DCIS_single_1
DCIS_single_3
DCIS_single_4
DCIS_single_8
DCIS_single_10
0
50
100
position (Mb)

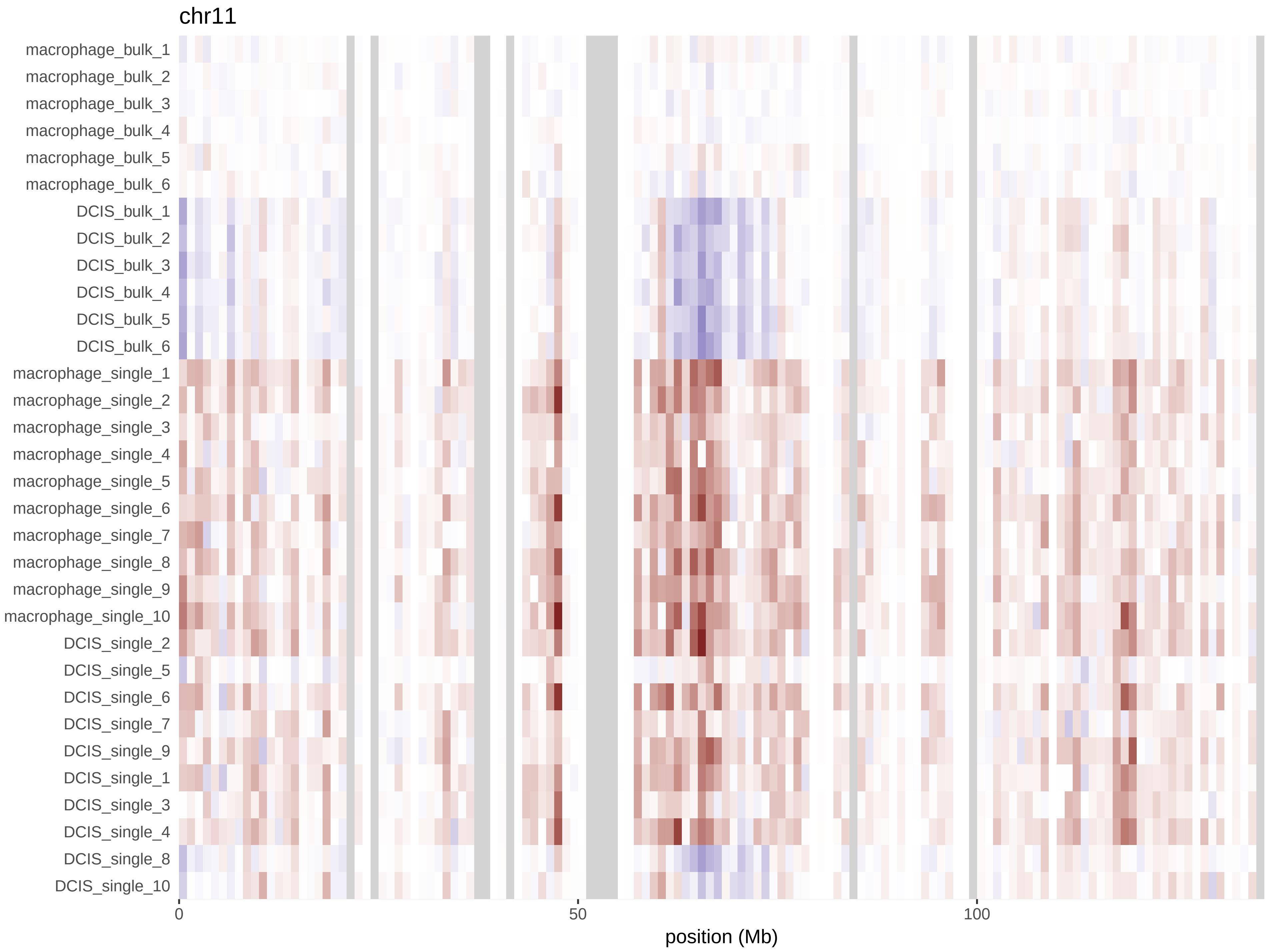

chr11
macrophage_bulk_1
macrophage_bulk_2
macrophage_bulk_3
macrophage_bulk_4
macrophage_bulk_5
macrophage_bulk_6
DCIS_bulk_1
DCIS_bulk_2
DCIS_bulk_3
DCIS_bulk_4
DCIS_bulk_5
DCIS_bulk_6
macrophage_single_1
macrophage_single_2
macrophage_single_3
macrophage_single_4
macrophage_single_5
macrophage_single_6
macrophage_single_7
macrophage_single_8
macrophage_single_9
macrophage_single_10
DCIS_single_2
DCIS_single_5
DCIS_single_6
DCIS_single_7
DCIS_single_9
DCIS_single_1
DCIS_single_3
DCIS_single_4
DCIS_single_8
DCIS_single_10
0
50
100
position (Mb)

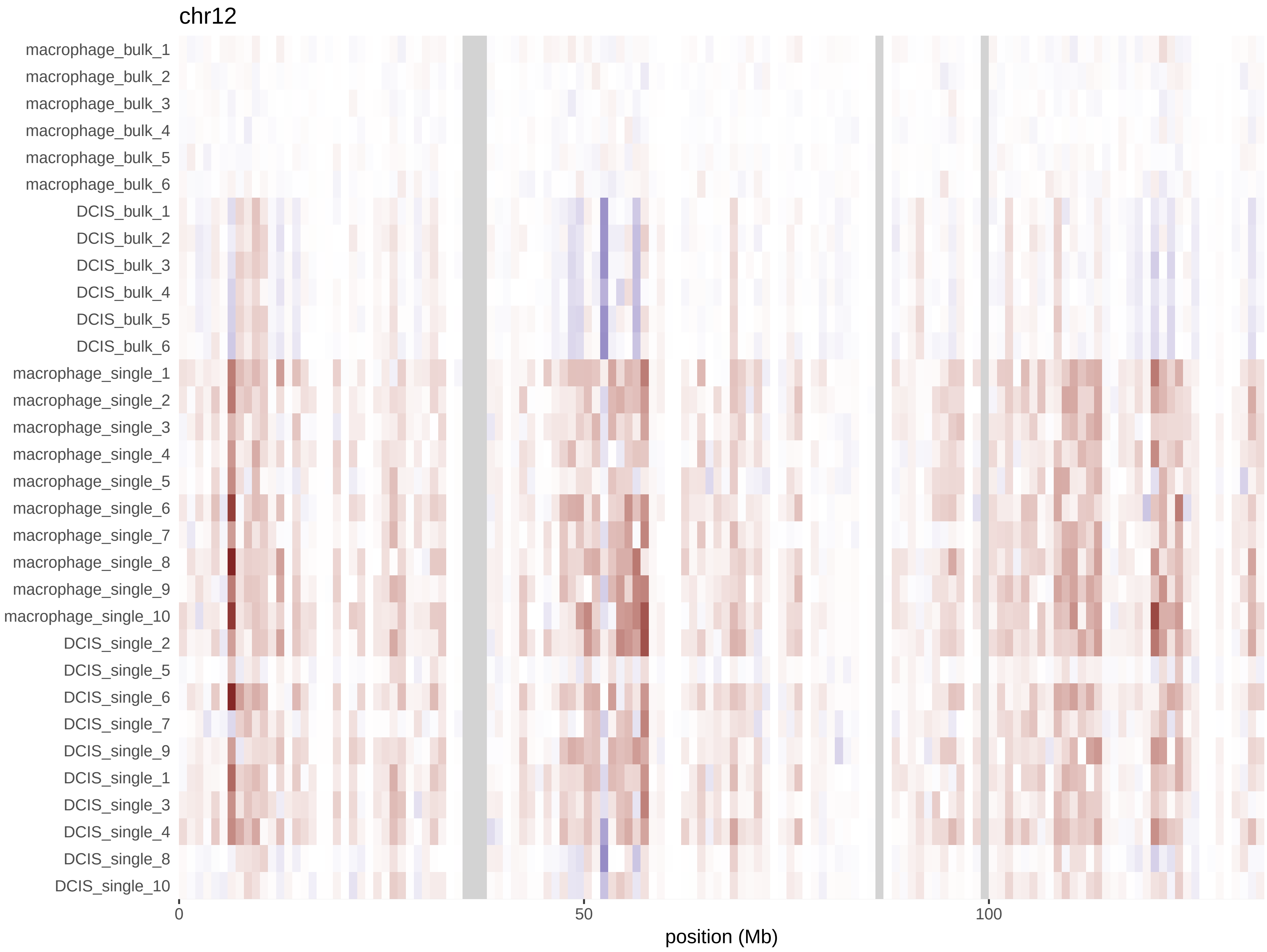
chr12
macrophage_bulk_1
macrophage_bulk_2
macrophage_bulk_3
macrophage_bulk_4
macrophage_bulk_5
macrophage_bulk_6
DCIS_bulk_1
DCIS_bulk_2
DCIS_bulk_3
DCIS_bulk_4
DCIS_bulk_5
DCIS_bulk_6
macrophage_single_1
macrophage_single_2
macrophage_single_3
macrophage_single_4
macrophage_single_5
macrophage_single_6
macrophage_single_7
macrophage_single_8
macrophage_single_9
macrophage_single_10
DCIS_single_2
DCIS_single_5
DCIS_single_6
DCIS_single_7
DCIS_single_9
DCIS_single_1
DCIS_single_3
DCIS_single_4
DCIS_single_8
DCIS_single_10
0
50
100
position (Mb)

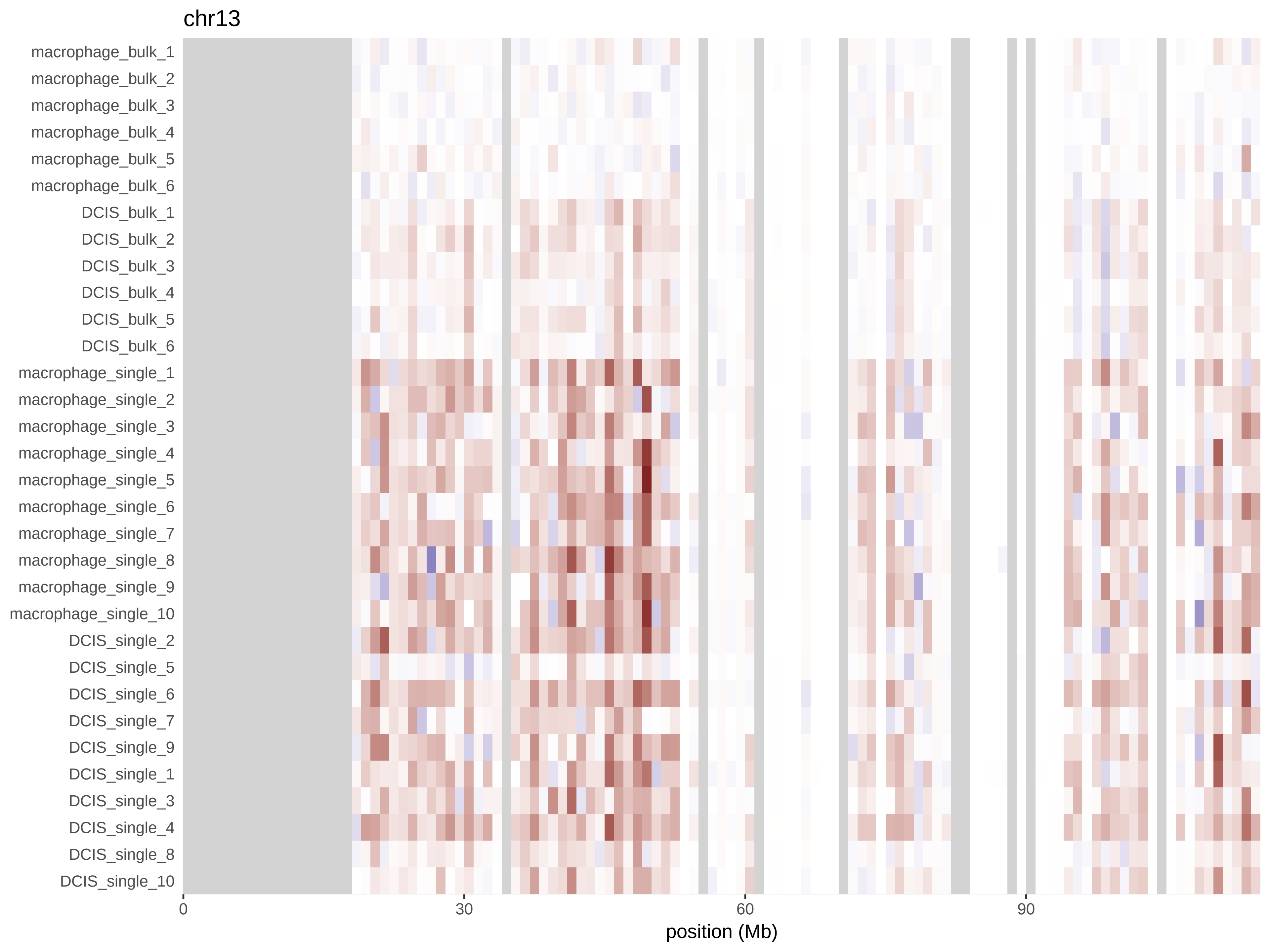
chr13
macrophage_bulk_1
macrophage_bulk_2
macrophage_bulk_3
macrophage_bulk_4
macrophage_bulk_5
macrophage_bulk_6
DCIS_bulk_1
DCIS_bulk_2
DCIS_bulk_3
DCIS_bulk_4
DCIS_bulk_5
DCIS_bulk_6
macrophage_single_1
macrophage_single_2
macrophage_single_3
macrophage_single_4
macrophage_single_5
macrophage_single_6
macrophage_single_7
macrophage_single_8
macrophage_single_9
macrophage_single_10
DCIS_single_2
DCIS_single_5
DCIS_single_6
DCIS_single_7
DCIS_single_9
DCIS_single_1
DCIS_single_3
DCIS_single_4
DCIS_single_8
DCIS_single_10
0
30
60
90
position (Mb)

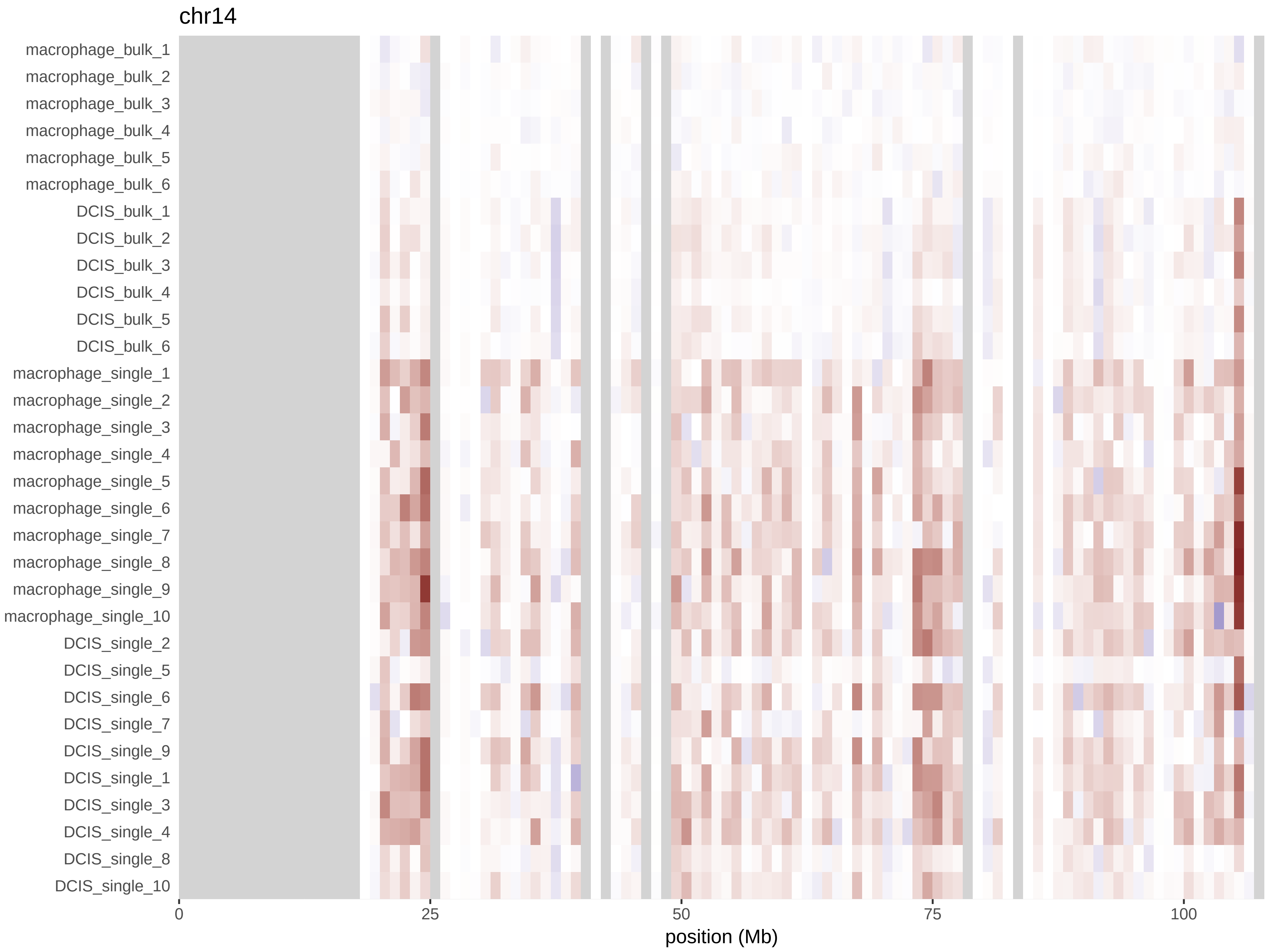

chr14
macrophage_bulk_1
macrophage_bulk_2
macrophage_bulk_3
macrophage_bulk_4
macrophage_bulk_5
macrophage_bulk_6
DCIS_bulk_1
DCIS_bulk_2
DCIS_bulk_3
DCIS_bulk_4
DCIS_bulk_5
DCIS_bulk_6
macrophage_single_1
macrophage_single_2
macrophage_single_3
macrophage_single_4
macrophage_single_5
macrophage_single_6
macrophage_single_7
macrophage_single_8
macrophage_single_9
macrophage_single_10
DCIS_single_2
DCIS_single_5
DCIS_single_6
DCIS_single_7
DCIS_single_9
DCIS_single_1
DCIS_single_3
DCIS_single_4
DCIS_single_8
DCIS_single_10
0
25
50
75
100
position (Mb)

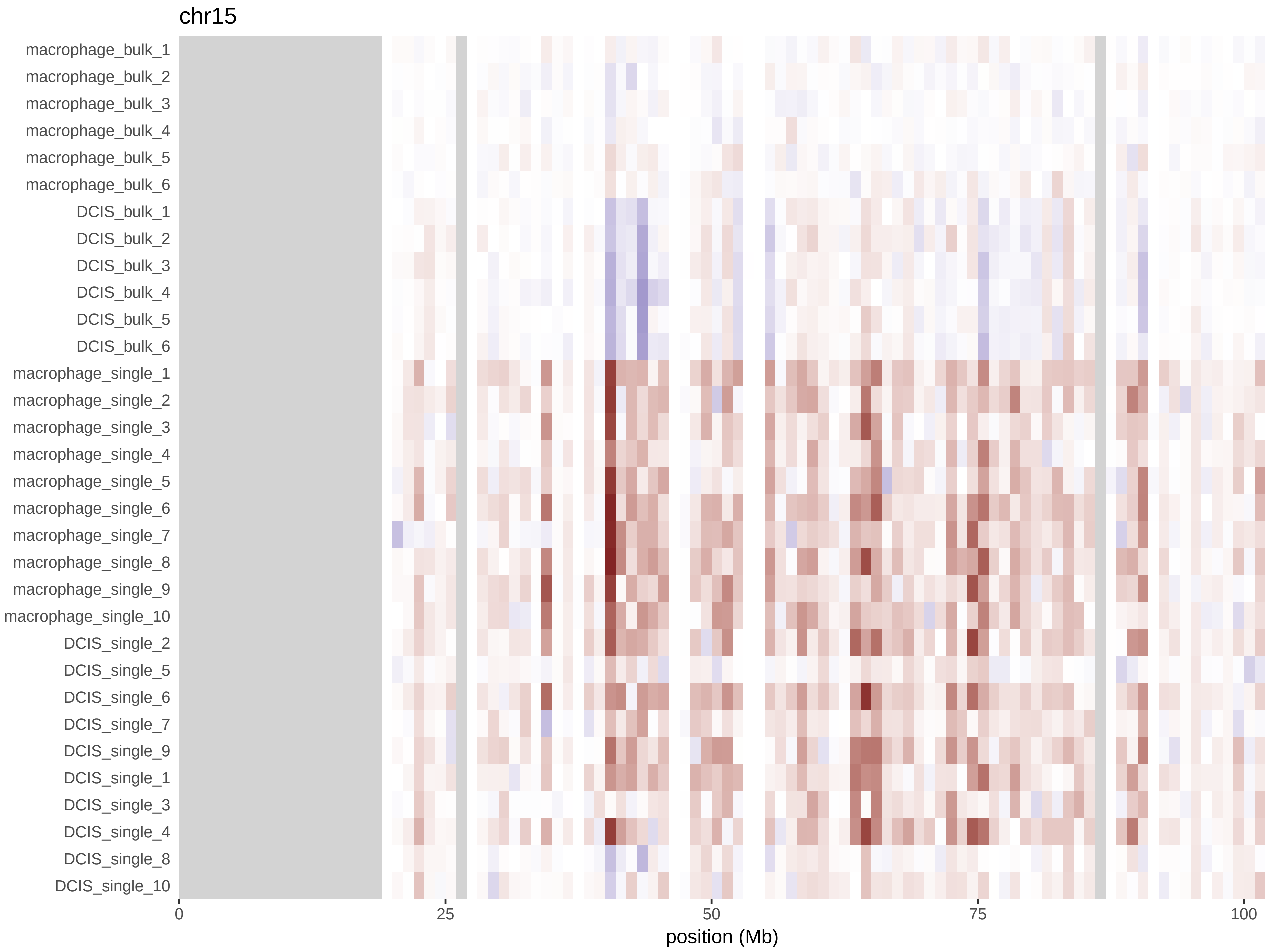
chr15
macrophage_bulk_1
macrophage_bulk_2
macrophage_bulk_3
macrophage_bulk_4
macrophage_bulk_5
macrophage_bulk_6
DCIS_bulk_1
DCIS_bulk_2
DCIS_bulk_3
DCIS_bulk_4
DCIS_bulk_5
DCIS_bulk_6
macrophage_single_1
macrophage_single_2
macrophage_single_3
macrophage_single_4
macrophage_single_5
macrophage_single_6
macrophage_single_7
macrophage_single_8
macrophage_single_9
macrophage_single_10
DCIS_single_2
DCIS_single_5
DCIS_single_6
DCIS_single_7
DCIS_single_9
DCIS_single_1
DCIS_single_3
DCIS_single_4
DCIS_single_8
DCIS_single_10
0
25
50
75
100
position (Mb)

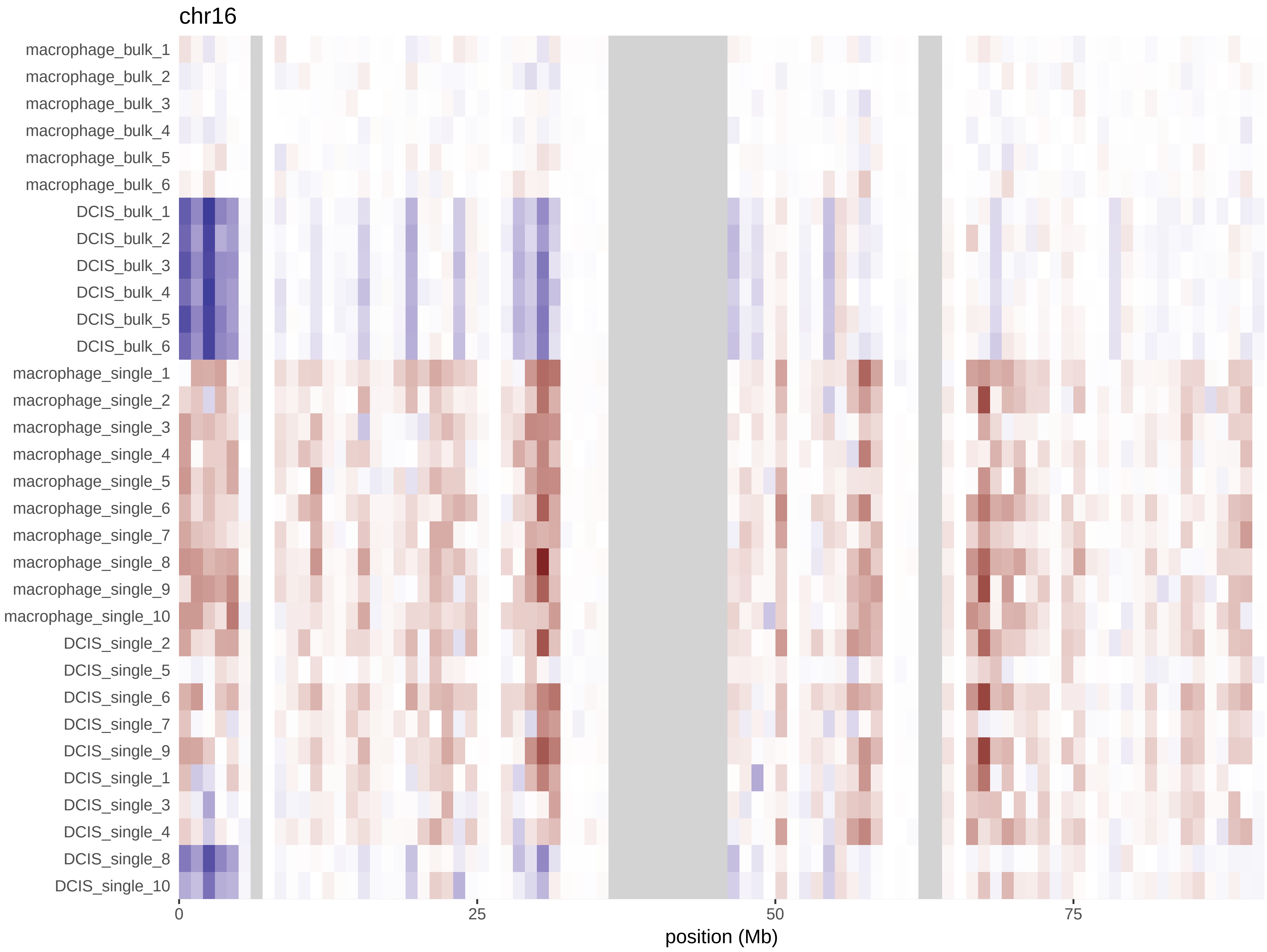

chr16
macrophage_bulk_1
macrophage_bulk_2
macrophage_bulk_3
macrophage_bulk_4
macrophage_bulk_5
macrophage_bulk_6
DCIS_bulk_1
DCIS_bulk_2
DCIS_bulk_3
DCIS_bulk_4
DCIS_bulk_5
DCIS_bulk_6
macrophage_single_1
macrophage_single_2
macrophage_single_3
macrophage_single_4
macrophage_single_5
macrophage_single_6
macrophage_single_7
macrophage_single_8
macrophage_single_9
macrophage_single_10
DCIS_single_2
DCIS_single_5
DCIS_single_6
DCIS_single_7
DCIS_single_9
DCIS_single_1
DCIS_single_3
DCIS_single_4
DCIS_single_8
DCIS_single_10
0
25
50
75
position (Mb)

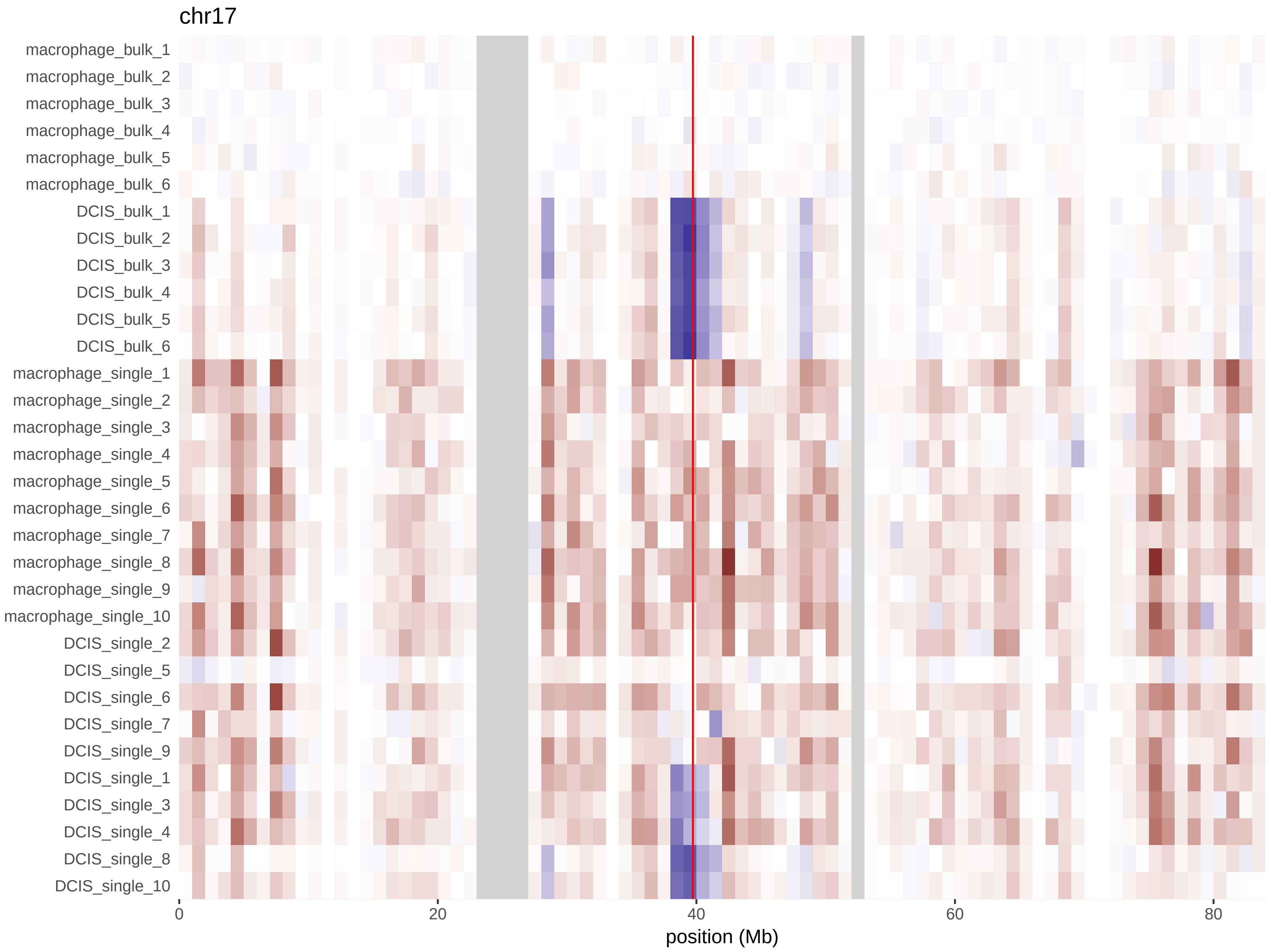
chr17
macrophage_bulk_1
macrophage_bulk_2
macrophage_bulk_3
macrophage_bulk_4
macrophage_bulk_5
macrophage_bulk_6
DCIS_bulk_1
DCIS_bulk_2
DCIS_bulk_3
DCIS_bulk_4
DCIS_bulk_5
DCIS_bulk_6
macrophage_single_1
macrophage_single_2
macrophage_single_3
macrophage_single_4
macrophage_single_5
macrophage_single_6
macrophage_single_7
macrophage_single_8
macrophage_single_9
macrophage_single_10
DCIS_single_2
DCIS_single_5
DCIS_single_6
DCIS_single_7
DCIS_single_9
DCIS_single_1
DCIS_single_3
DCIS_single_4
DCIS_single_8
DCIS_single_10
0
20
40
60
80
position (Mb)

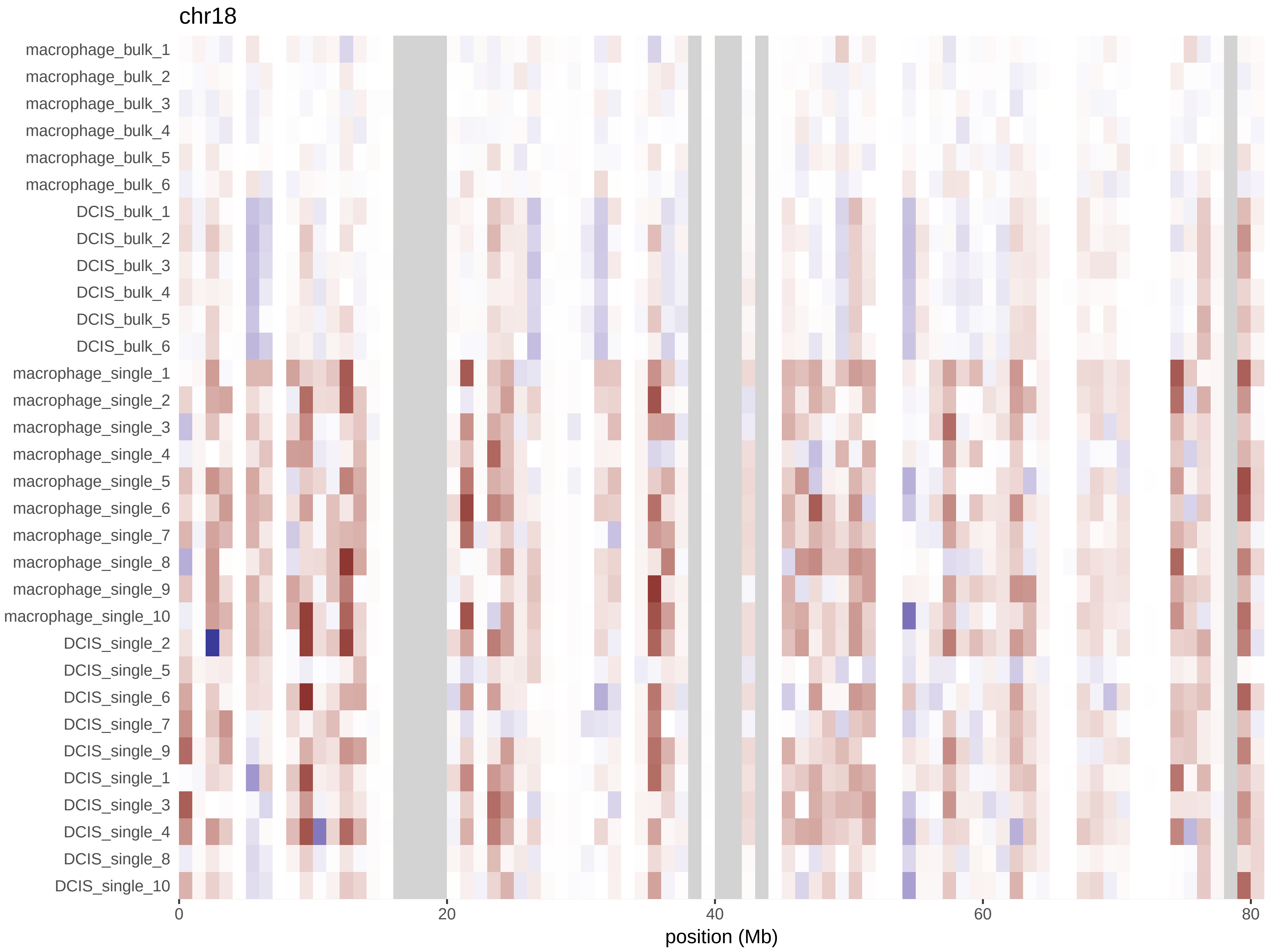
chr18
macrophage_bulk_1
macrophage_bulk_2
macrophage_bulk_3
macrophage_bulk_4
macrophage_bulk_5
macrophage_bulk_6
DCIS_bulk_1
DCIS_bulk_2
DCIS_bulk_3
DCIS_bulk_4
DCIS_bulk_5
DCIS_bulk_6
macrophage_single_1
macrophage_single_2
macrophage_single_3
macrophage_single_4
macrophage_single_5
macrophage_single_6
macrophage_single_7
macrophage_single_8
macrophage_single_9
macrophage_single_10
DCIS_single_2
DCIS_single_5
DCIS_single_6
DCIS_single_7
DCIS_single_9
DCIS_single_1
DCIS_single_3
DCIS_single_4
DCIS_single_8
DCIS_single_10
0
20
40
60
80
position (Mb)

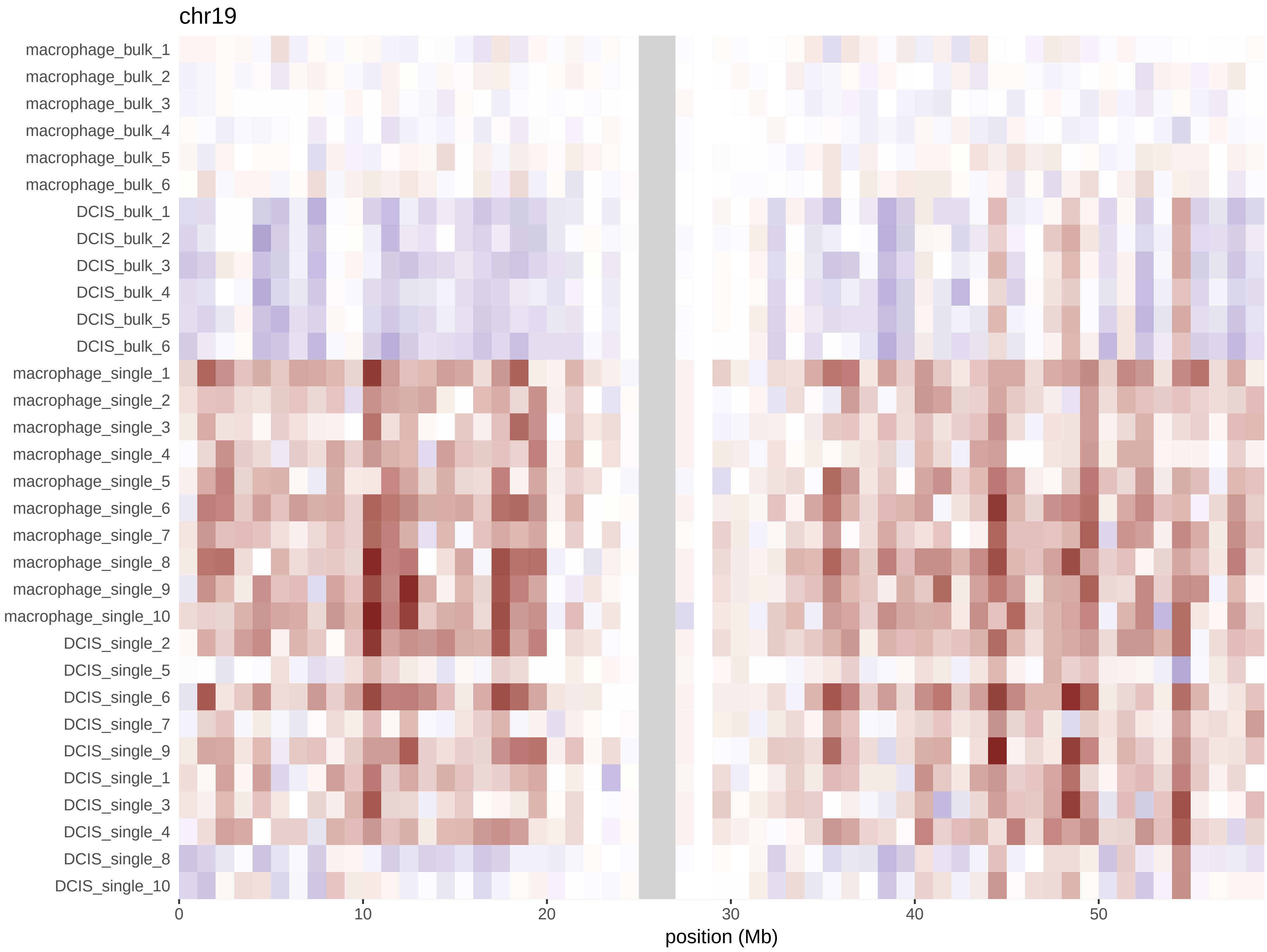
chr19
macrophage_bulk_1
macrophage_bulk_2
macrophage_bulk_3
macrophage_bulk_4
macrophage_bulk_5
macrophage_bulk_6
DCIS_bulk_1
DCIS_bulk_2
DCIS_bulk_3
DCIS_bulk_4
DCIS_bulk_5
DCIS_bulk_6
macrophage_single_1
macrophage_single_2
macrophage_single_3
macrophage_single_4
macrophage_single_5
macrophage_single_6
macrophage_single_7
macrophage_single_8
macrophage_single_9
macrophage_single_10
DCIS_single_2
DCIS_single_5
DCIS_single_6
DCIS_single_7
DCIS_single_9
DCIS_single_1
DCIS_single_3
DCIS_single_4
DCIS_single_8
DCIS_single_10
0
10
20
30
40
50
position (Mb)

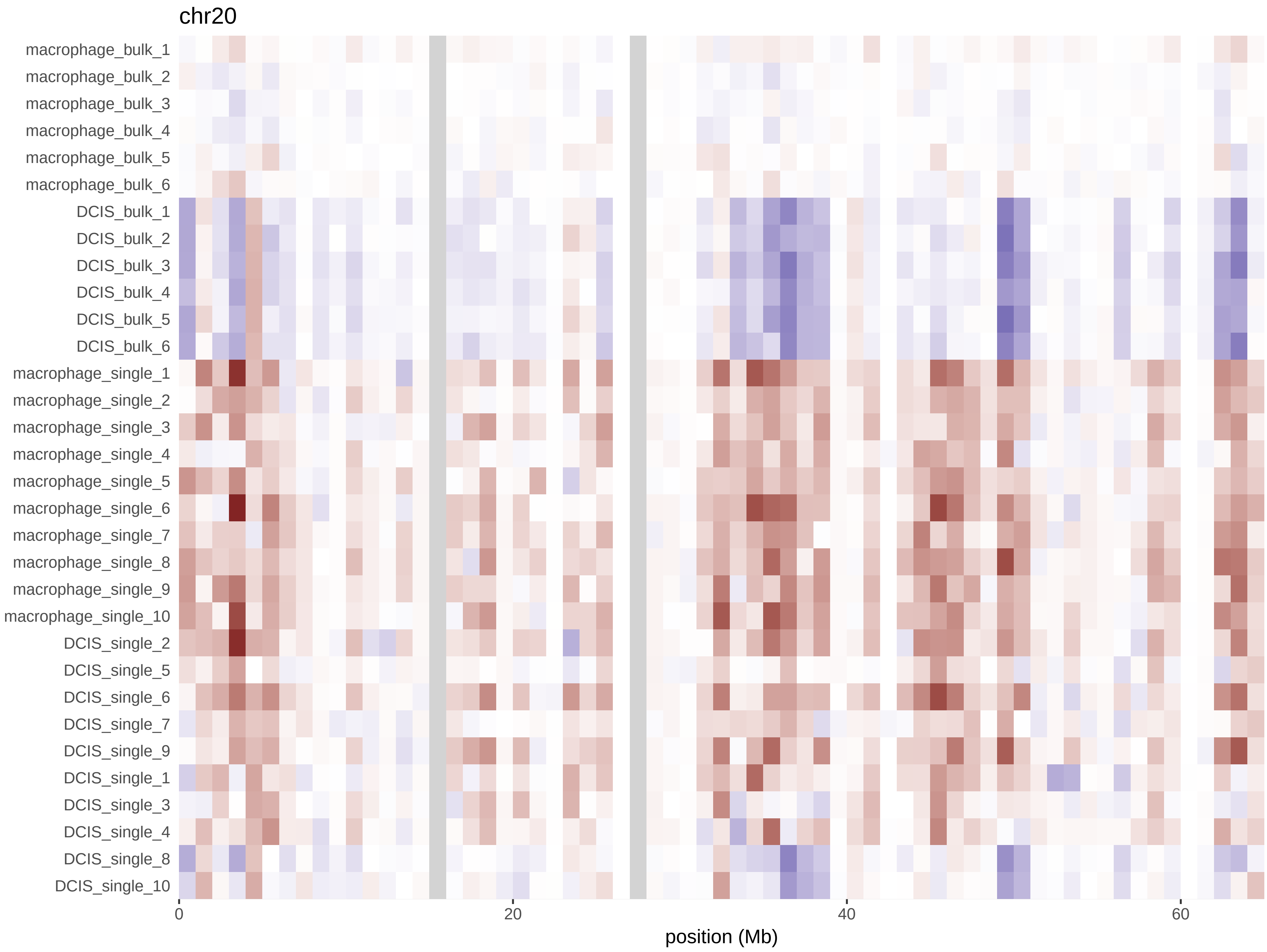

chr20
macrophage_bulk_1
macrophage_bulk_2
macrophage_bulk_3
macrophage_bulk_4
macrophage_bulk_5
macrophage_bulk_6
DCIS_bulk_1
DCIS_bulk_2
DCIS_bulk_3
DCIS_bulk_4
DCIS_bulk_5
DCIS_bulk_6
macrophage_single_1
macrophage_single_2
macrophage_single_3
macrophage_single_4
macrophage_single_5
macrophage_single_6
macrophage_single_7
macrophage_single_8
macrophage_single_9
macrophage_single_10
DCIS_single_2
DCIS_single_5
DCIS_single_6
DCIS_single_7
DCIS_single_9
DCIS_single_1
DCIS_single_3
DCIS_single_4
DCIS_single_8
DCIS_single_10
0
20
40
60
position (Mb)

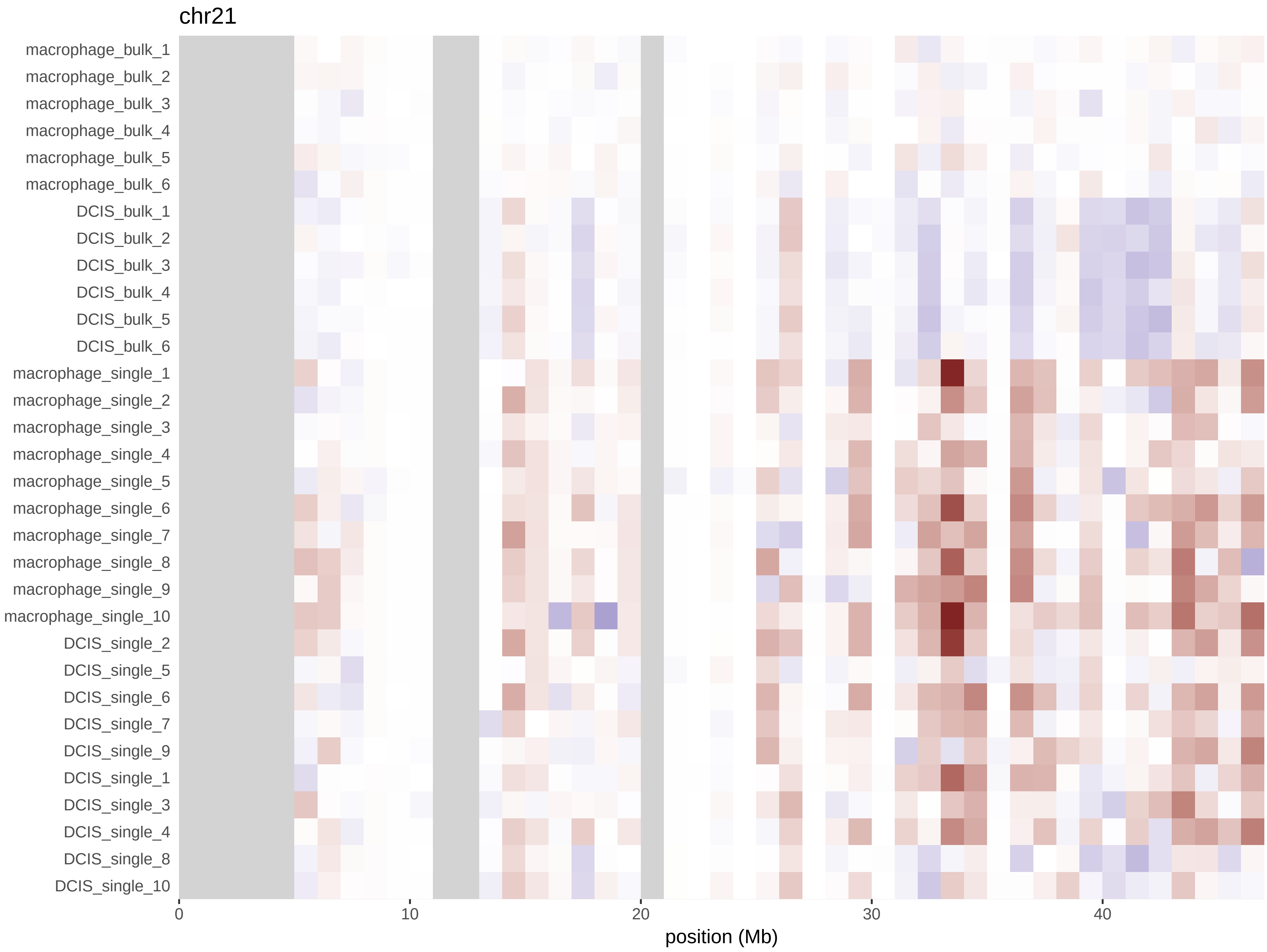
chr21
macrophage_bulk_1
macrophage_bulk_2
macrophage_bulk_3
macrophage_bulk_4
macrophage_bulk_5
macrophage_bulk_6
DCIS_bulk_1
DCIS_bulk_2
DCIS_bulk_3
DCIS_bulk_4
DCIS_bulk_5
DCIS_bulk_6
macrophage_single_1
macrophage_single_2
macrophage_single_3
macrophage_single_4
macrophage_single_5
macrophage_single_6
macrophage_single_7
macrophage_single_8
macrophage_single_9
macrophage_single_10
DCIS_single_2
DCIS_single_5
DCIS_single_6
DCIS_single_7
DCIS_single_9
DCIS_single_1
DCIS_single_3
DCIS_single_4
DCIS_single_8
DCIS_single_10
0
10
20
30
40
position (Mb)

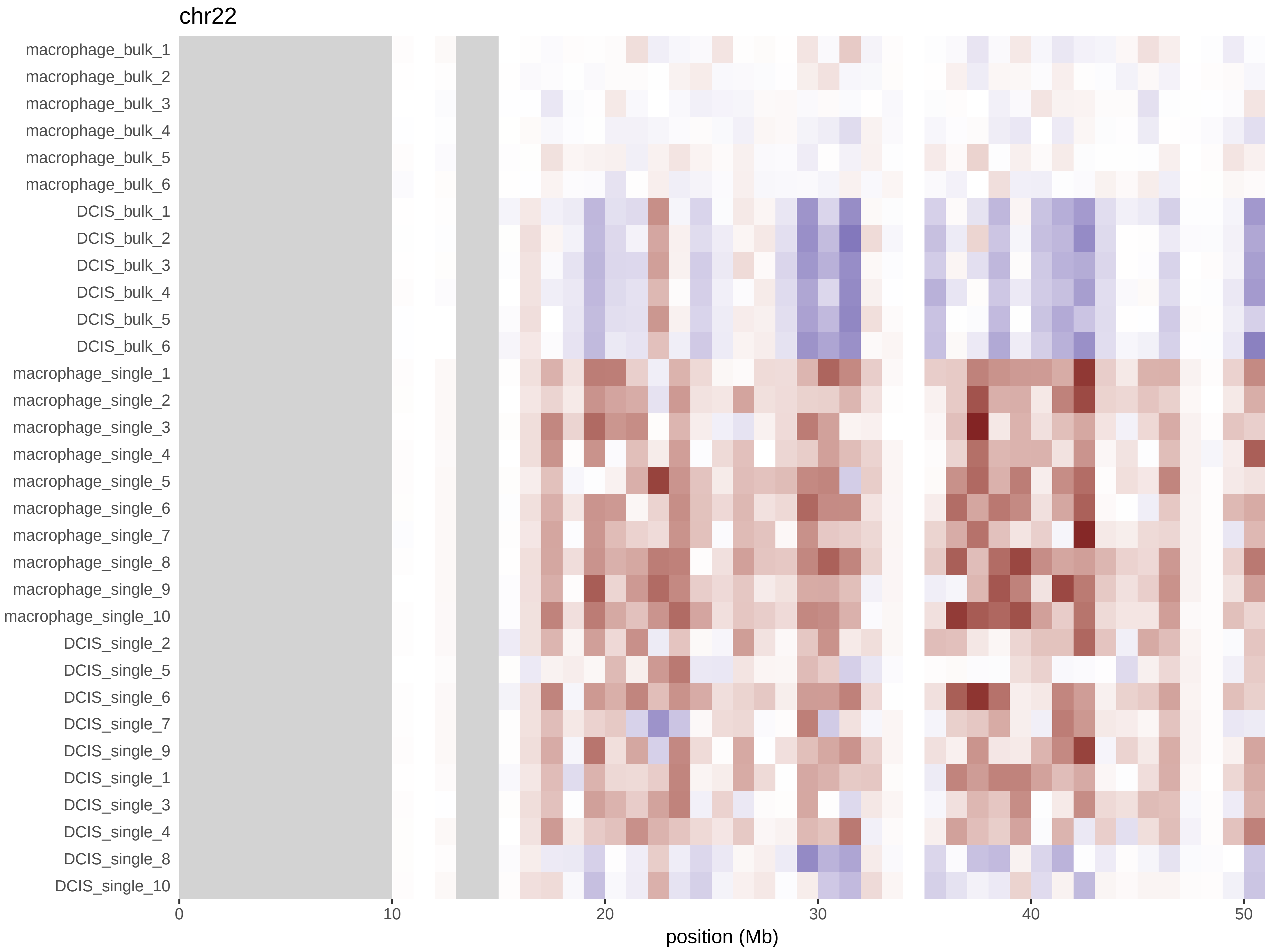
chr22
macrophage_bulk_1
macrophage_bulk_2
macrophage_bulk_3
macrophage_bulk_4
macrophage_bulk_5
macrophage_bulk_6
DCIS_bulk_1
DCIS_bulk_2
DCIS_bulk_3
DCIS_bulk_4
DCIS_bulk_5
DCIS_bulk_6
macrophage_single_1
macrophage_single_2
macrophage_single_3
macrophage_single_4
macrophage_single_5
macrophage_single_6
macrophage_single_7
macrophage_single_8
macrophage_single_9
macrophage_single_10
DCIS_single_2
DCIS_single_5
DCIS_single_6
DCIS_single_7
DCIS_single_9
DCIS_single_1
DCIS_single_3
DCIS_single_4
DCIS_single_8
DCIS_single_10
0
10
20
30
40
50
position (Mb)

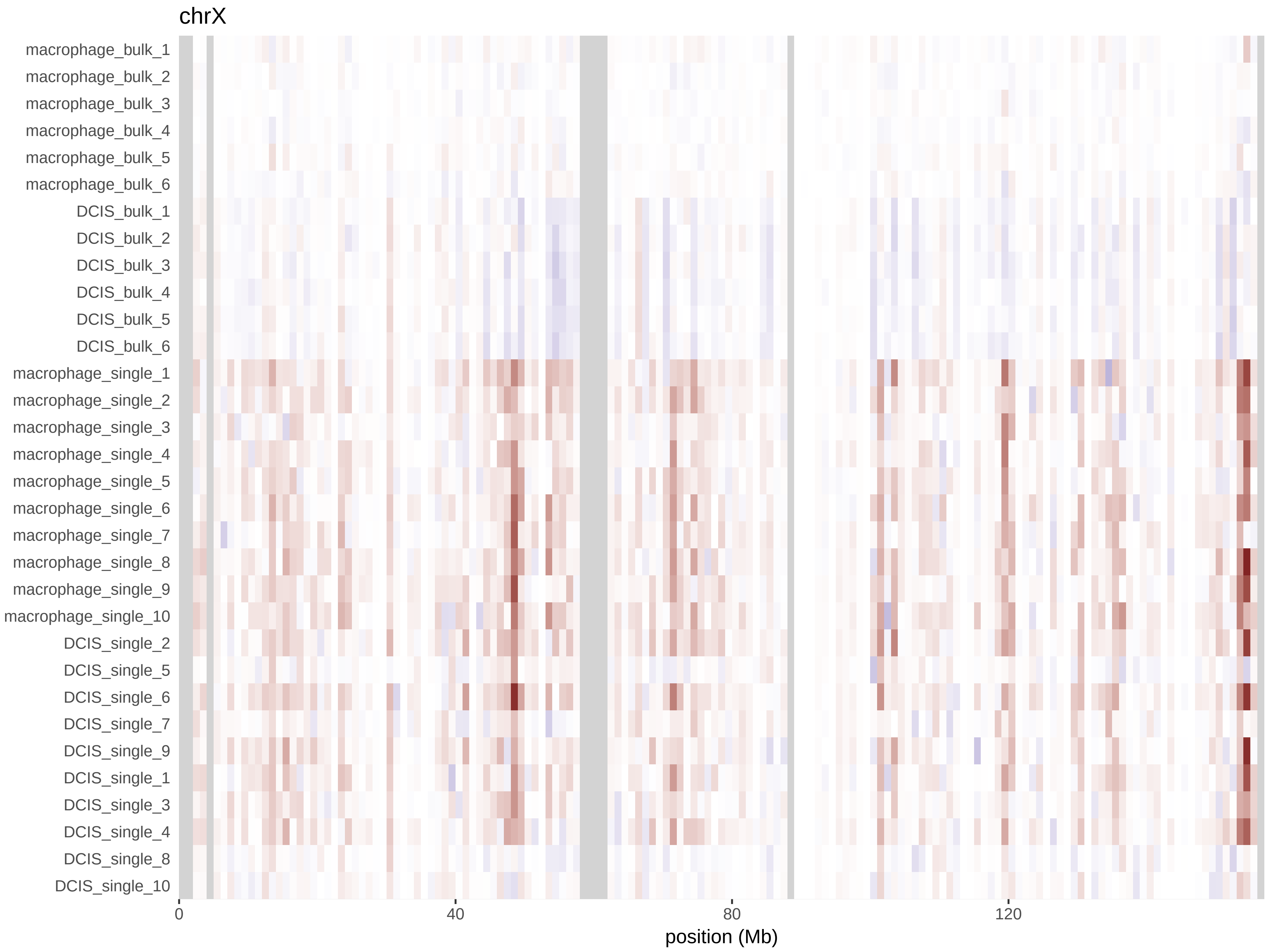

chrX
macrophage_bulk_1
macrophage_bulk_2
macrophage_bulk_3
macrophage_bulk_4
macrophage_bulk_5
macrophage_bulk_6
DCIS_bulk_1
DCIS_bulk_2
DCIS_bulk_3
DCIS_bulk_4
DCIS_bulk_5
DCIS_bulk_6
macrophage_single_1
macrophage_single_2
macrophage_single_3
macrophage_single_4
macrophage_single_5
macrophage_single_6
macrophage_single_7
macrophage_single_8
macrophage_single_9
macrophage_single_10
DCIS_single_2
DCIS_single_5
DCIS_single_6
DCIS_single_7
DCIS_single_9
DCIS_single_1
DCIS_single_3
DCIS_single_4
DCIS_single_8
DCIS_single_10
0
40
80
120
position (Mb)
