# Supplementary material for: Gene expression profiling of single cells from archival tissue with laser-capture microdissection and Smart-3SEQ
Source: Genome Res. 2019 Nov;29(11):1816–25. doi: 10.1101/gr.234807.118 (PMC6836736; doi:10.1101/gr.234807.118)
Supplement: Supplemental Material [file supp_gr.234807.118_Supplemental_Code.zip › Supplemental_Code.tar!/fig/categories_ffpe-lcm_legend-moved.pdf]

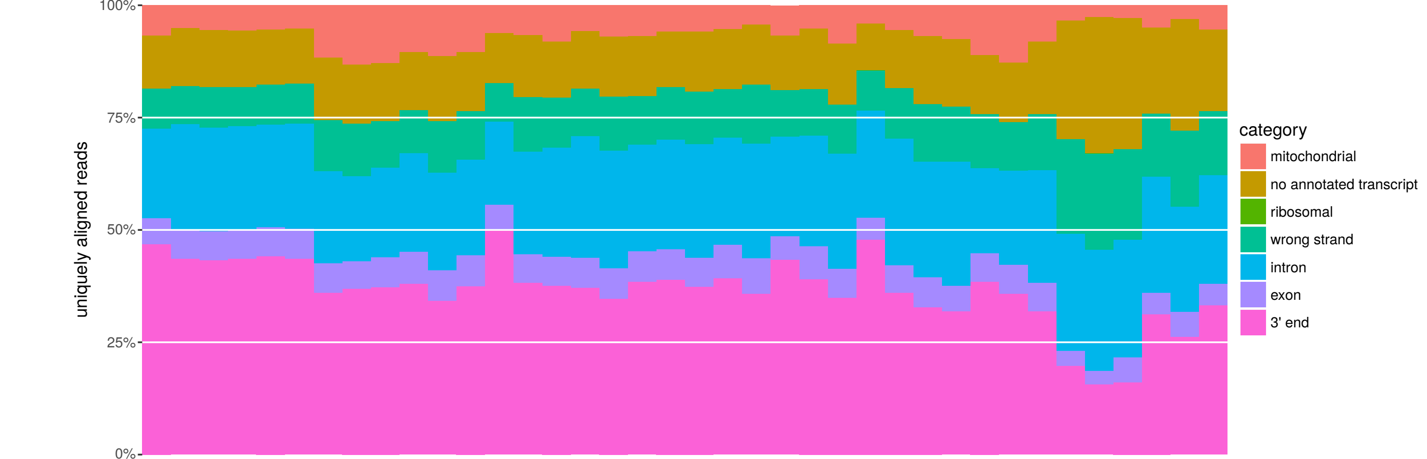
100%
75%
50%
25%
0%
uniquely aligned reads
category
mitochondrial
no annotated transcript
ribosomal
wrong strand
intron
exon
3' end
