# Supplementary material for: Gene expression profiling of single cells from archival tissue with laser-capture microdissection and Smart-3SEQ
Source: Genome Res. 2019 Nov;29(11):1816–25. doi: 10.1101/gr.234807.118 (PMC6836736; doi:10.1101/gr.234807.118)
Supplement: Supplemental Material [file supp_gr.234807.118_Supplemental_Code.zip › Supplemental_Code.tar!/fig/smart-3seq_workflow_diagram.pdf]

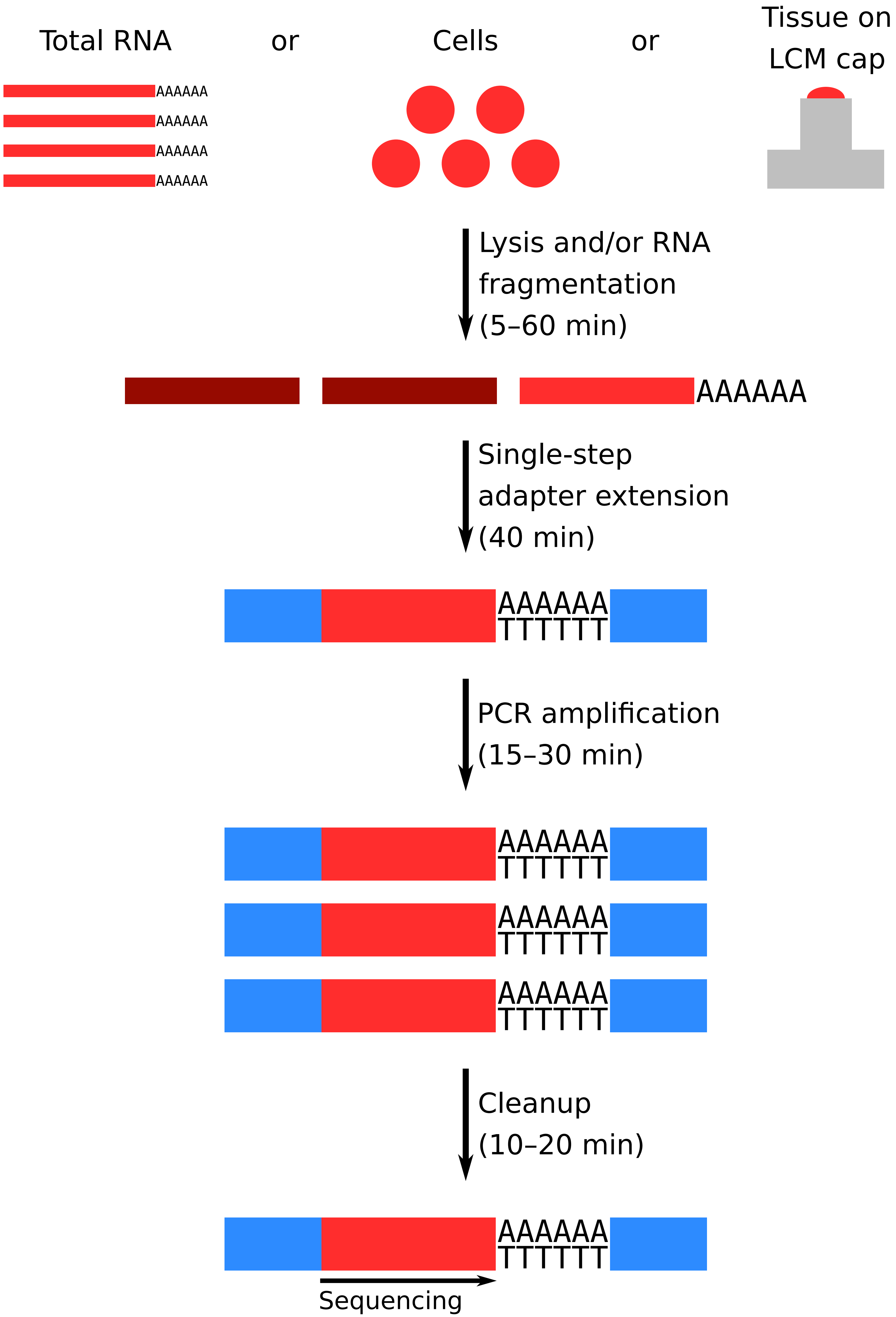
Total RNA
or
Cells
or
Tissue on LCM cap
AAAAAA
AAAAAA
AAAAAA
AAAAAA
Lysis and/or RNA fragmentation (5–60 min)
AAAAAA
Single-step adapter extension (40 min)
AAAAAA
TTTTTT
PCR amplification (15–30 min)
AAAAAA
TTTTTT
AAAAAA
TTTTTT
AAAAAA
TTTTTT
Cleanup (10–20 min)
AAAAAA
TTTTTT
Sequencing
