# Supplementary material for: Gene expression profiling of single cells from archival tissue with laser-capture microdissection and Smart-3SEQ
Source: Genome Res. 2019 Nov;29(11):1816–25. doi: 10.1101/gr.234807.118 (PMC6836736; doi:10.1101/gr.234807.118)
Supplement: Supplemental Material [file supp_gr.234807.118_Supplemental_Code.zip › Supplemental_Code.tar!/fig/alignability_ffpe-lcm.pdf]

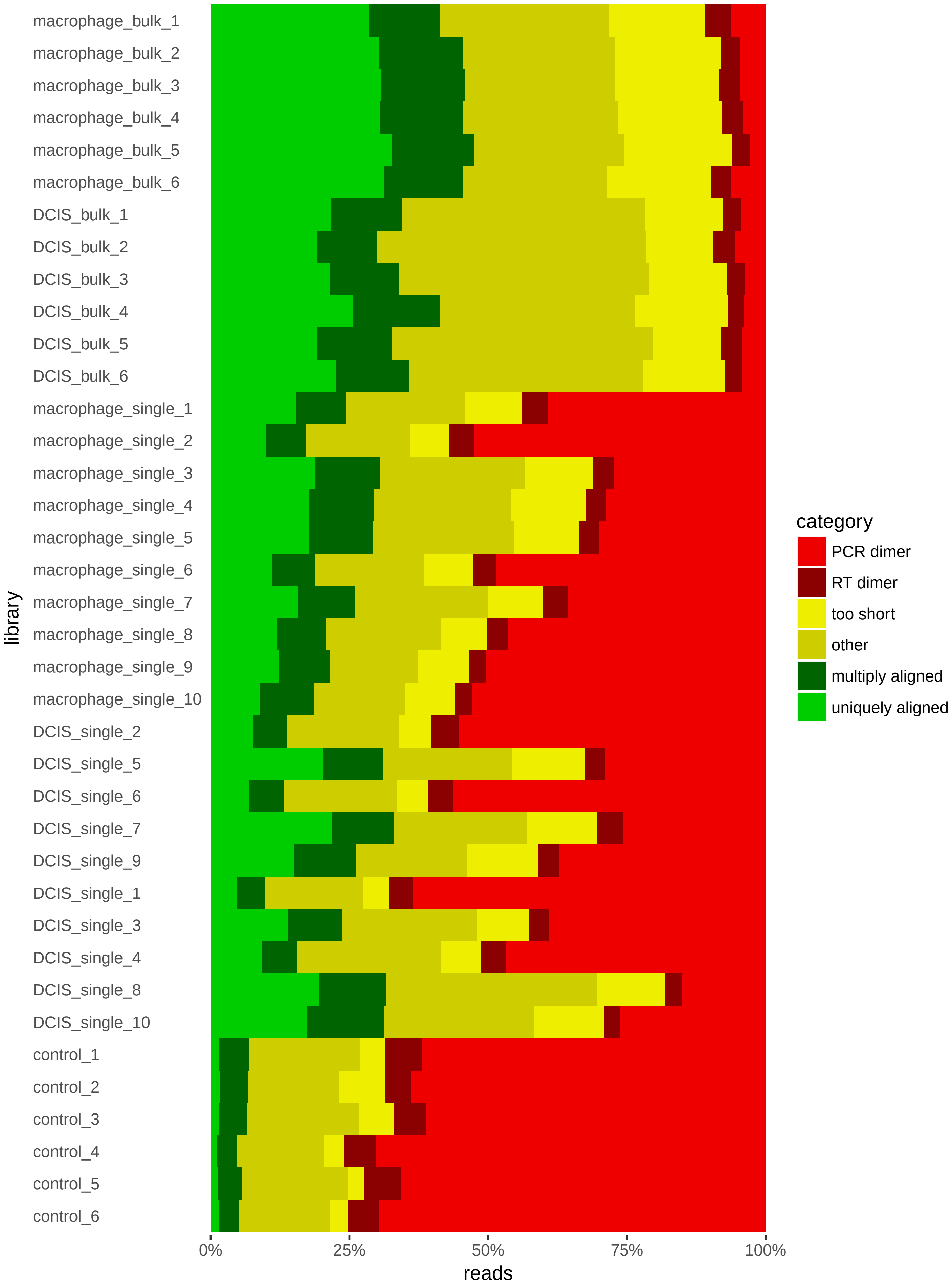

macrophage_bulk_1
macrophage_bulk_2
macrophage_bulk_3
macrophage_bulk_4
macrophage_bulk_5
macrophage_bulk_6
DCIS_bulk_1
DCIS_bulk_2
DCIS_bulk_3
DCIS_bulk_4
DCIS_bulk_5
DCIS_bulk_6
macrophage_single_1
macrophage_single_2
macrophage_single_3
macrophage_single_4
macrophage_single_5
macrophage_single_6
macrophage_single_7
macrophage_single_8
macrophage_single_9
macrophage_single_10
DCIS_single_2
DCIS_single_5
DCIS_single_6
DCIS_single_7
DCIS_single_9
DCIS_single_1
DCIS_single_3
DCIS_single_4
DCIS_single_8
DCIS_single_10
control_1
control_2
control_3
control_4
control_5
control_6
library
0%
25%
50%
75%
100%
reads
category
PCR dimer
RT dimer
too short
other
multiply aligned
uniquely aligned
