# Supplementary material for: Gene expression profiling of single cells from archival tissue with laser-capture microdissection and Smart-3SEQ
Source: Genome Res. 2019 Nov;29(11):1816–25. doi: 10.1101/gr.234807.118 (PMC6836736; doi:10.1101/gr.234807.118)
Supplement: Supplemental Material [file supp_gr.234807.118_Supplemental_Code.zip › Supplemental_Code.tar!/fig/compare_3seq_taqman.pdf]

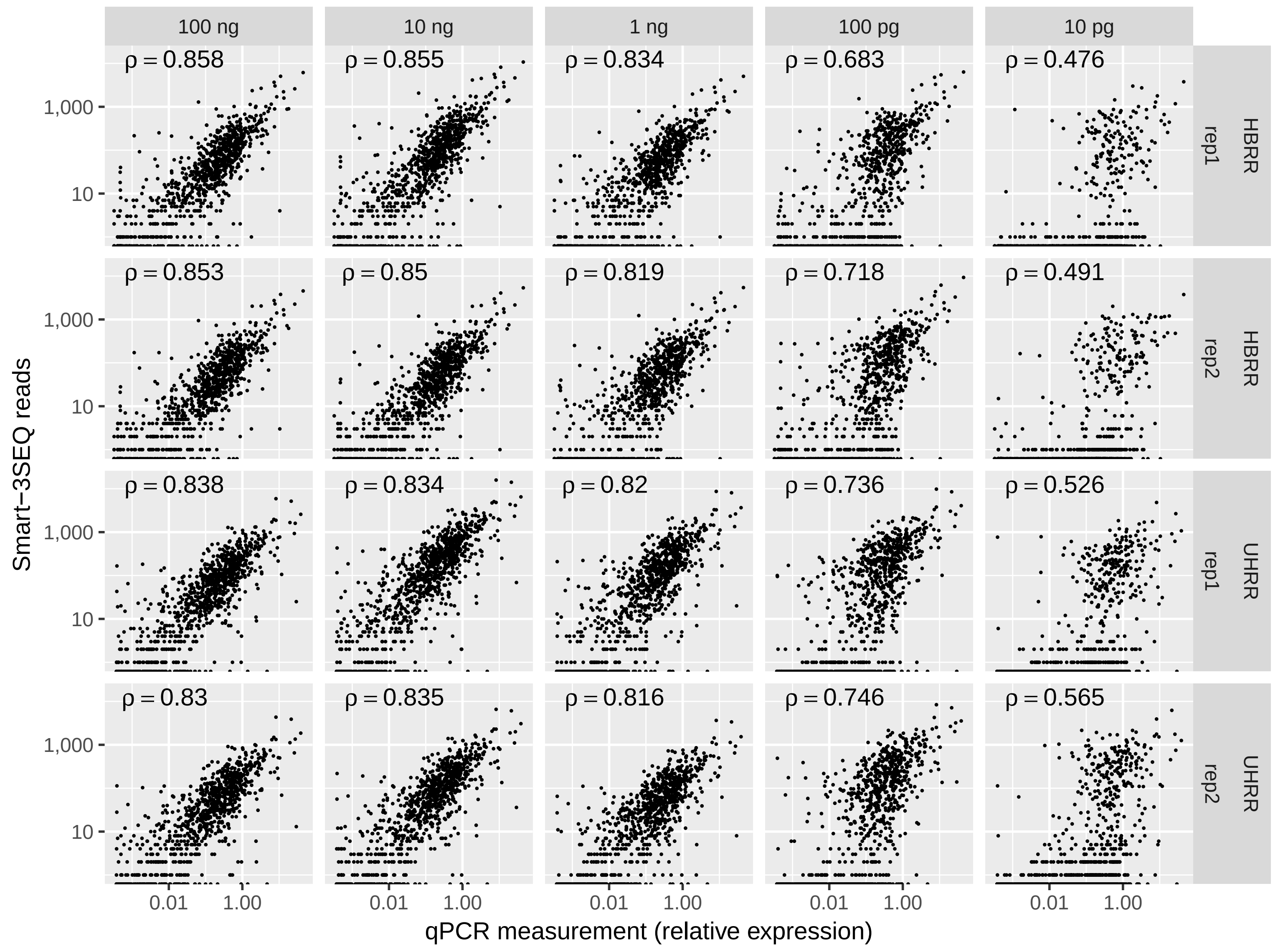
100 ng
10 ng
1 ng
100 pg
10 pg
HBRR rep1
HBRR rep2
UHRR rep1
UHRR rep2
ρ = 0.858
ρ = 0.855
ρ = 0.834
ρ = 0.683
ρ = 0.476
ρ = 0.853
ρ = 0.85
ρ = 0.819
ρ = 0.718
ρ = 0.491
ρ = 0.838
ρ = 0.834
ρ = 0.82
ρ = 0.736
ρ = 0.526
ρ = 0.83
ρ = 0.835
ρ = 0.816
ρ = 0.746
ρ = 0.565
1,000
10
0.01
1.00
Smart−3SEQ reads
qPCR measurement (relative expression)
