# Supplementary material for: Gene expression profiling of single cells from archival tissue with laser-capture microdissection and Smart-3SEQ
Source: Genome Res. 2019 Nov;29(11):1816–25. doi: 10.1101/gr.234807.118 (PMC6836736; doi:10.1101/gr.234807.118)
Supplement: Supplemental Material [file supp_gr.234807.118_Supplemental_Code.zip › Supplemental_Code.tar!/fig/expt_design_ercc.pdf]

**ERCC mix 1 or ERCC mix 2**	10 fmol	→	1 fmol	→	100 amol	→	10 amol	→	1 amol	No RNA
**PCR cycles**	7 or 11		11 or 15		14 or 18		17 or 22		21 or 25	21 or 25
