# Supplementary material for: Gene expression profiling of single cells from archival tissue with laser-capture microdissection and Smart-3SEQ
Source: Genome Res. 2019 Nov;29(11):1816–25. doi: 10.1101/gr.234807.118 (PMC6836736; doi:10.1101/gr.234807.118)
Supplement: Supplemental Material [file supp_gr.234807.118_Supplemental_Code.zip › Supplemental_Code.tar!/fig/stdcrv_3seq.pdf]

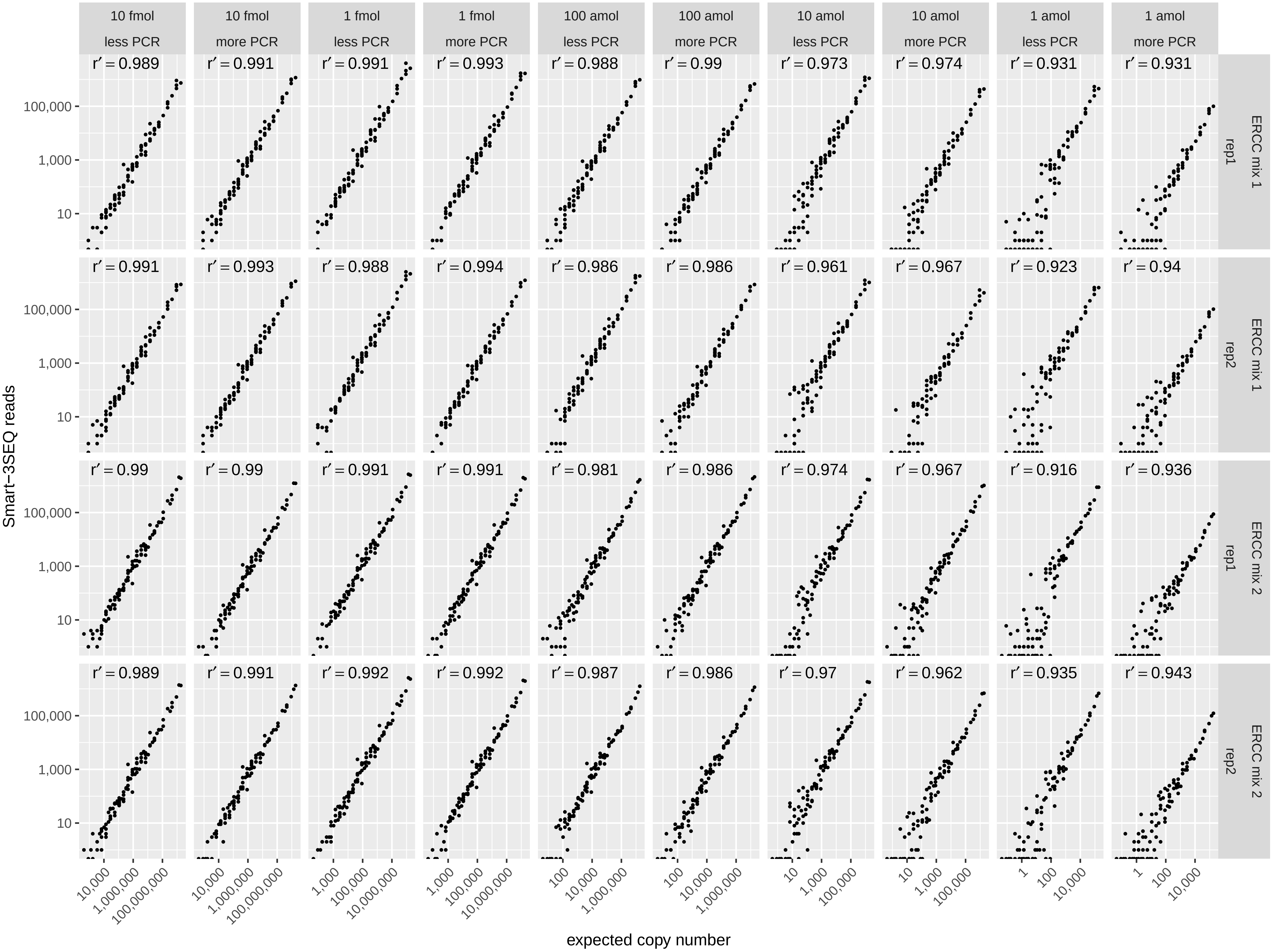
10 fmol less PCR
10 fmol more PCR
1 fmol less PCR
1 fmol more PCR
100 amol less PCR
100 amol more PCR
10 amol less PCR
10 amol more PCR
1 amol less PCR
1 amol more PCR
ERCC mix 1 rep1
ERCC mix 1 rep2
ERCC mix 2 rep1
ERCC mix 2 rep2
r′ = 0.989
r′ = 0.991
r′ = 0.991
r′ = 0.993
r′ = 0.988
r′ = 0.99
r′ = 0.973
r′ = 0.974
r′ = 0.931
r′ = 0.931
r′ = 0.991
r′ = 0.993
r′ = 0.988
r′ = 0.994
r′ = 0.986
r′ = 0.986
r′ = 0.961
r′ = 0.967
r′ = 0.923
r′ = 0.94
r′ = 0.99
r′ = 0.99
r′ = 0.991
r′ = 0.991
r′ = 0.981
r′ = 0.986
r′ = 0.974
r′ = 0.967
r′ = 0.916
r′ = 0.936
r′ = 0.989
r′ = 0.991
r′ = 0.992
r′ = 0.992
r′ = 0.987
r′ = 0.986
r′ = 0.97
r′ = 0.962
r′ = 0.935
r′ = 0.943
Smart−3SEQ reads
100,000
1,000
10
expected copy number
10,000
1,000,000
100,000,000
1,000
100,000
10,000,000
100
10,000
1,000,000
10
1,000
100,000
1
100
10,000
