# Supplementary material for: Gene expression profiling of single cells from archival tissue with laser-capture microdissection and Smart-3SEQ
Source: Genome Res. 2019 Nov;29(11):1816–25. doi: 10.1101/gr.234807.118 (PMC6836736; doi:10.1101/gr.234807.118)
Supplement: Supplemental Material [file supp_gr.234807.118_Supplemental_Code.zip › Supplemental_Code.tar!/fig/validation_summary.pdf]

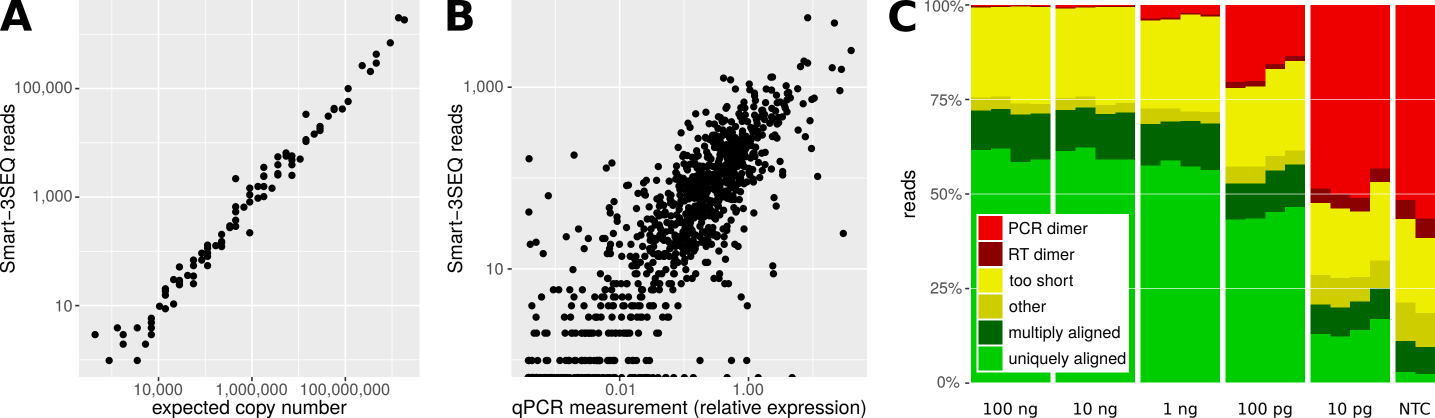
A
Smart−3SEQ reads
100,000
1,000
10
10,000
1,000,000
100,000,000
expected copy number
B
Smart−3SEQ reads
1,000
10
0.01
1.00
qPCR measurement (relative expression)
C
100%
75%
50%
25%
0%
reads
PCR dimer
RT dimer
too short
other
multiply aligned
uniquely aligned
100 ng
10 ng
1 ng
100 pg
10 pg
NTC
