# Supplementary material for: Gene expression profiling of single cells from archival tissue with laser-capture microdissection and Smart-3SEQ
Source: Genome Res. 2019 Nov;29(11):1816–25. doi: 10.1101/gr.234807.118 (PMC6836736; doi:10.1101/gr.234807.118)
Supplement: Supplemental Material [file supp_gr.234807.118_Supplemental_Code.zip › Supplemental_Code.tar!/fig/compare_clontech_sybr.pdf]

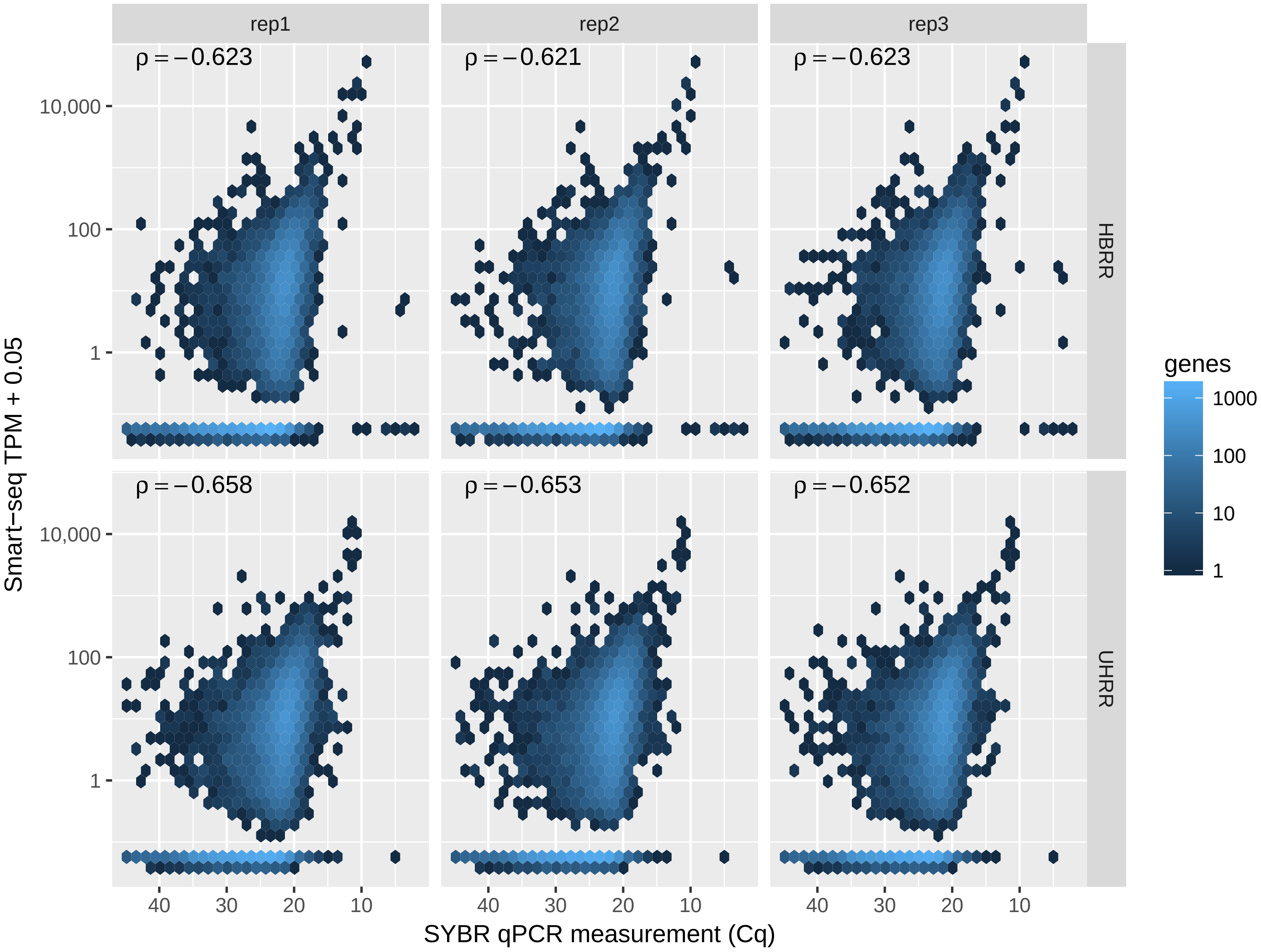
rep1
rep2
rep3
HBRR
UHRR
ρ = −0.623
ρ = −0.621
ρ = −0.623
ρ = −0.658
ρ = −0.653
ρ = −0.652
10,000
100
1
40
30
20
10
Smart−seq TPM + 0.05
SYBR qPCR measurement (Cq)
genes
1000
100
10
1
