# Supplementary material for: Gene expression profiling of single cells from archival tissue with laser-capture microdissection and Smart-3SEQ
Source: Genome Res. 2019 Nov;29(11):1816–25. doi: 10.1101/gr.234807.118 (PMC6836736; doi:10.1101/gr.234807.118)
Supplement: Supplemental Material [file supp_gr.234807.118_Supplemental_Code.zip › Supplemental_Code.tar!/fig/pc1_pc2_bulk_rotation_combined_ffpe-lcm2.pdf]

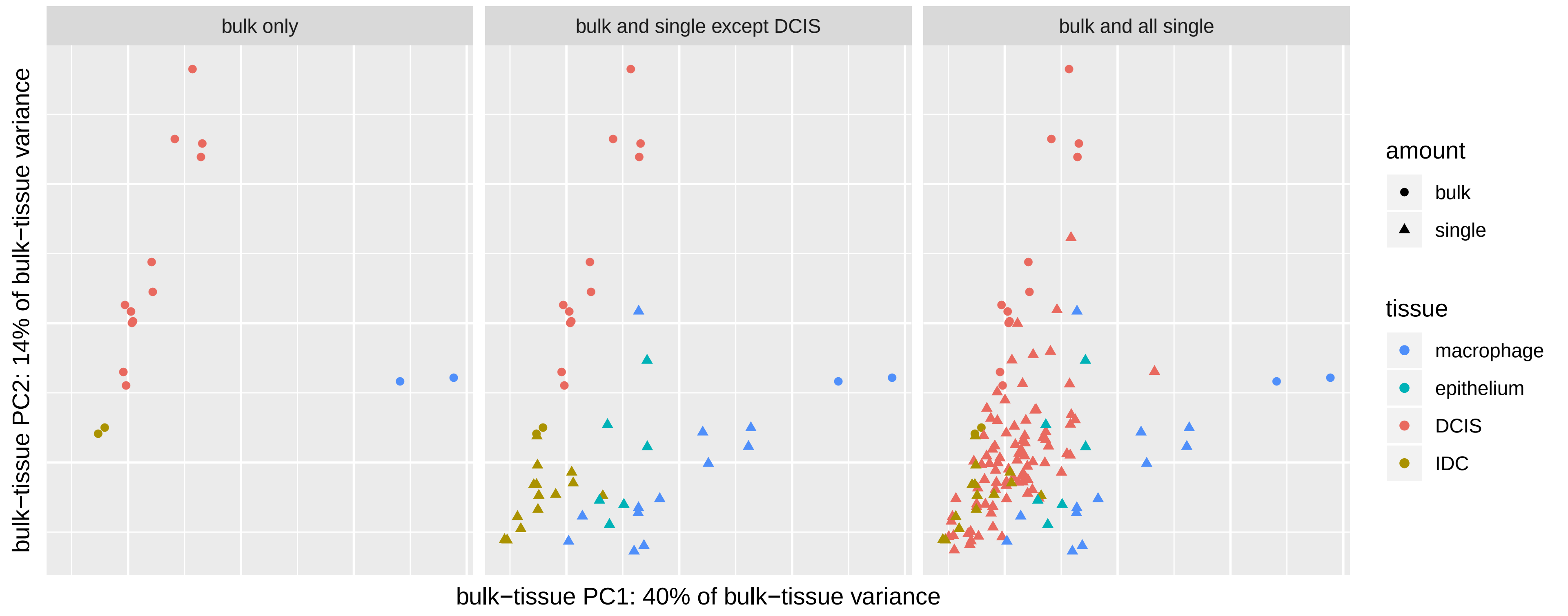

bulk only
bulk and single except DCIS
bulk and all single
bulk−tissue PC1: 40% of bulk−tissue variance
bulk−tissue PC2: 14% of bulk−tissue variance
amount
bulk
single
tissue
macrophage
epithelium
DCIS
IDC
