# Supplementary material for: Gene expression profiling of single cells from archival tissue with laser-capture microdissection and Smart-3SEQ
Source: Genome Res. 2019 Nov;29(11):1816–25. doi: 10.1101/gr.234807.118 (PMC6836736; doi:10.1101/gr.234807.118)
Supplement: Supplemental Material [file supp_gr.234807.118_Supplemental_Code.zip › Supplemental_Code.tar!/fig/heatmap_differential-genes_ablation.pdf]

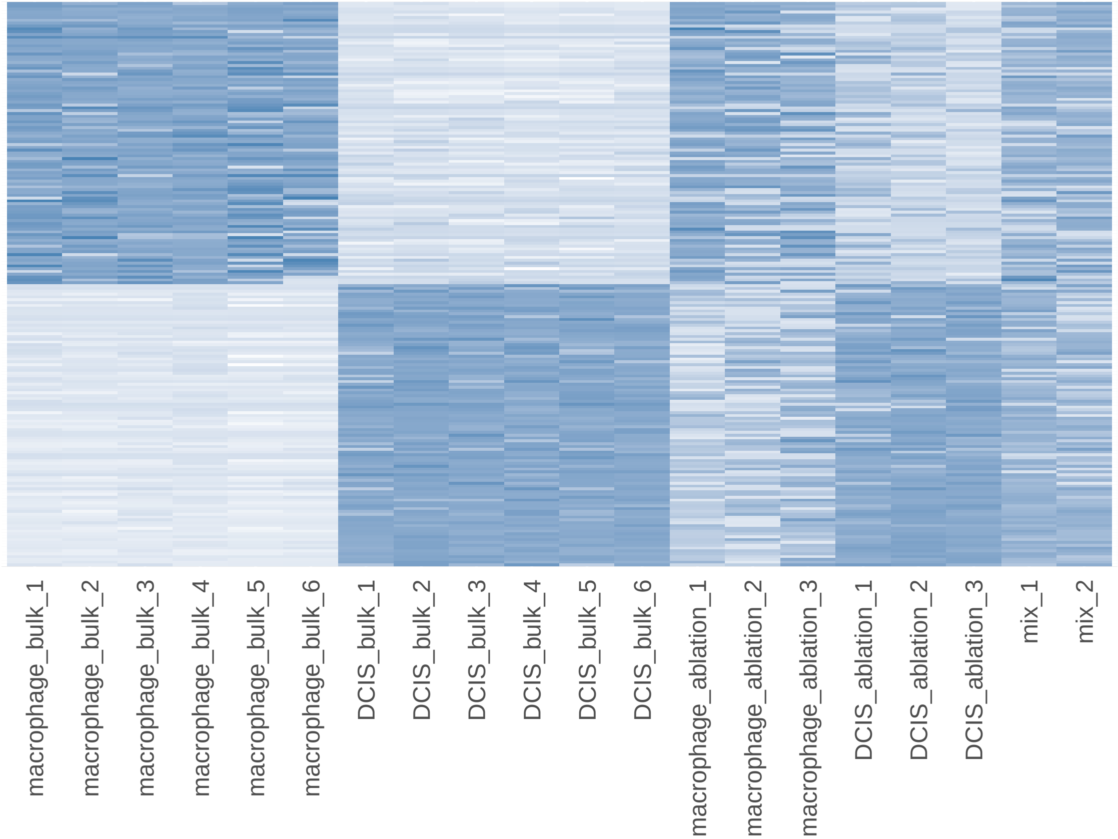
macrophage_bulk_1
macrophage_bulk_2
macrophage_bulk_3
macrophage_bulk_4
macrophage_bulk_5
macrophage_bulk_6
DCIS_bulk_1
DCIS_bulk_2
DCIS_bulk_3
DCIS_bulk_4
DCIS_bulk_5
DCIS_bulk_6
macrophage_ablation_1
macrophage_ablation_2
macrophage_ablation_3
DCIS_ablation_1
DCIS_ablation_2
DCIS_ablation_3
mix_1
mix_2
