# Supplementary material for: Gene expression profiling of single cells from archival tissue with laser-capture microdissection and Smart-3SEQ
Source: Genome Res. 2019 Nov;29(11):1816–25. doi: 10.1101/gr.234807.118 (PMC6836736; doi:10.1101/gr.234807.118)
Supplement: Supplemental Material [file supp_gr.234807.118_Supplemental_Code.zip › Supplemental_Code.tar!/fig/compare_seqc_taqman.pdf]

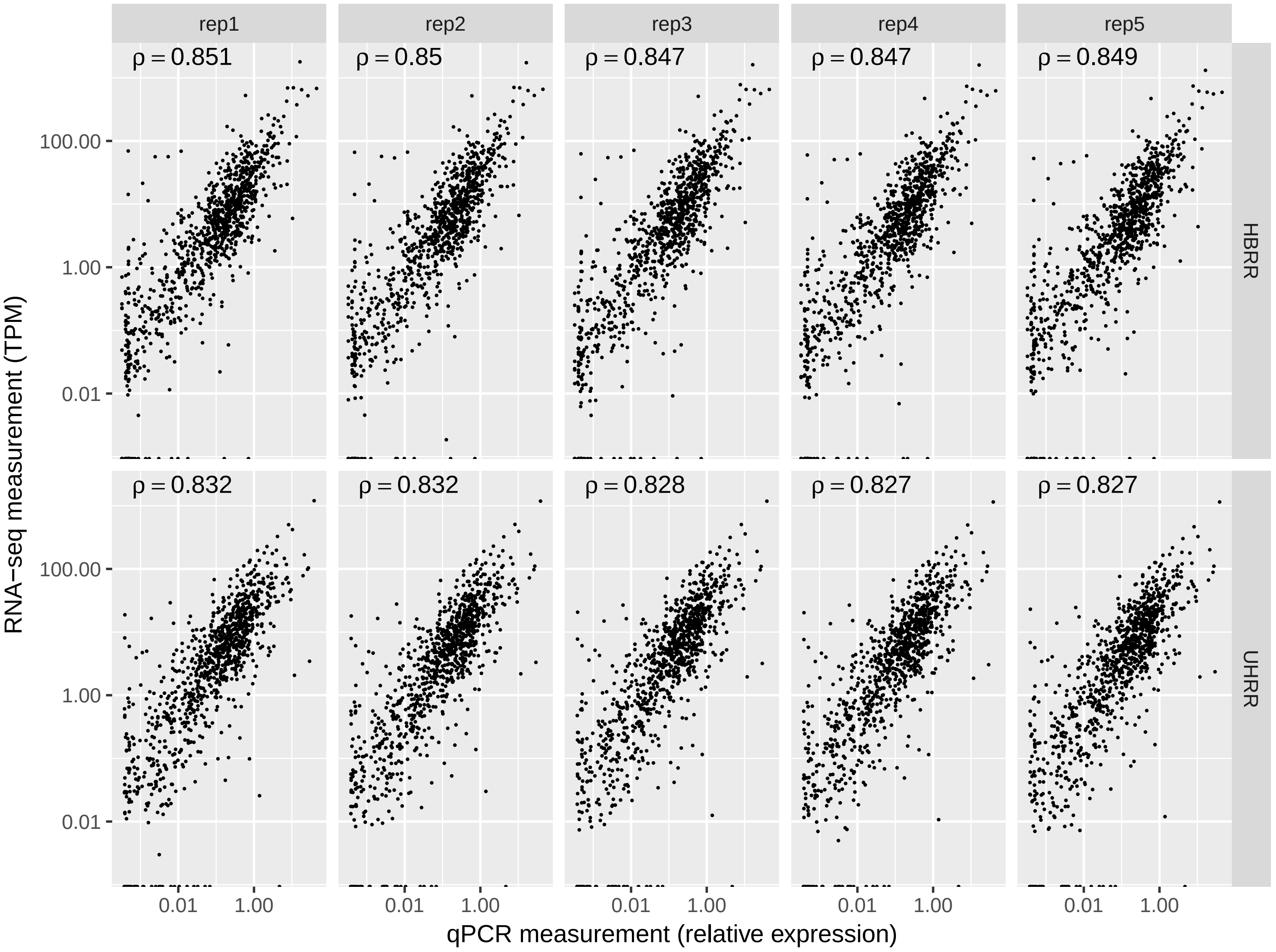

rep1
rep2
rep3
rep4
rep5
HBRR
UHRR
ρ = 0.851
ρ = 0.85
ρ = 0.847
ρ = 0.847
ρ = 0.849
ρ = 0.832
ρ = 0.832
ρ = 0.828
ρ = 0.827
ρ = 0.827
100.00
1.00
0.01
RNA−seq measurement (TPM)
qPCR measurement (relative expression)
