# Supplementary material for: Gene expression profiling of single cells from archival tissue with laser-capture microdissection and Smart-3SEQ
Source: Genome Res. 2019 Nov;29(11):1816–25. doi: 10.1101/gr.234807.118 (PMC6836736; doi:10.1101/gr.234807.118)
Supplement: Supplemental Material [file supp_gr.234807.118_Supplemental_Code.zip › Supplemental_Code.tar!/fig/insert_lengths_ffpe-vs-frozen.pdf]

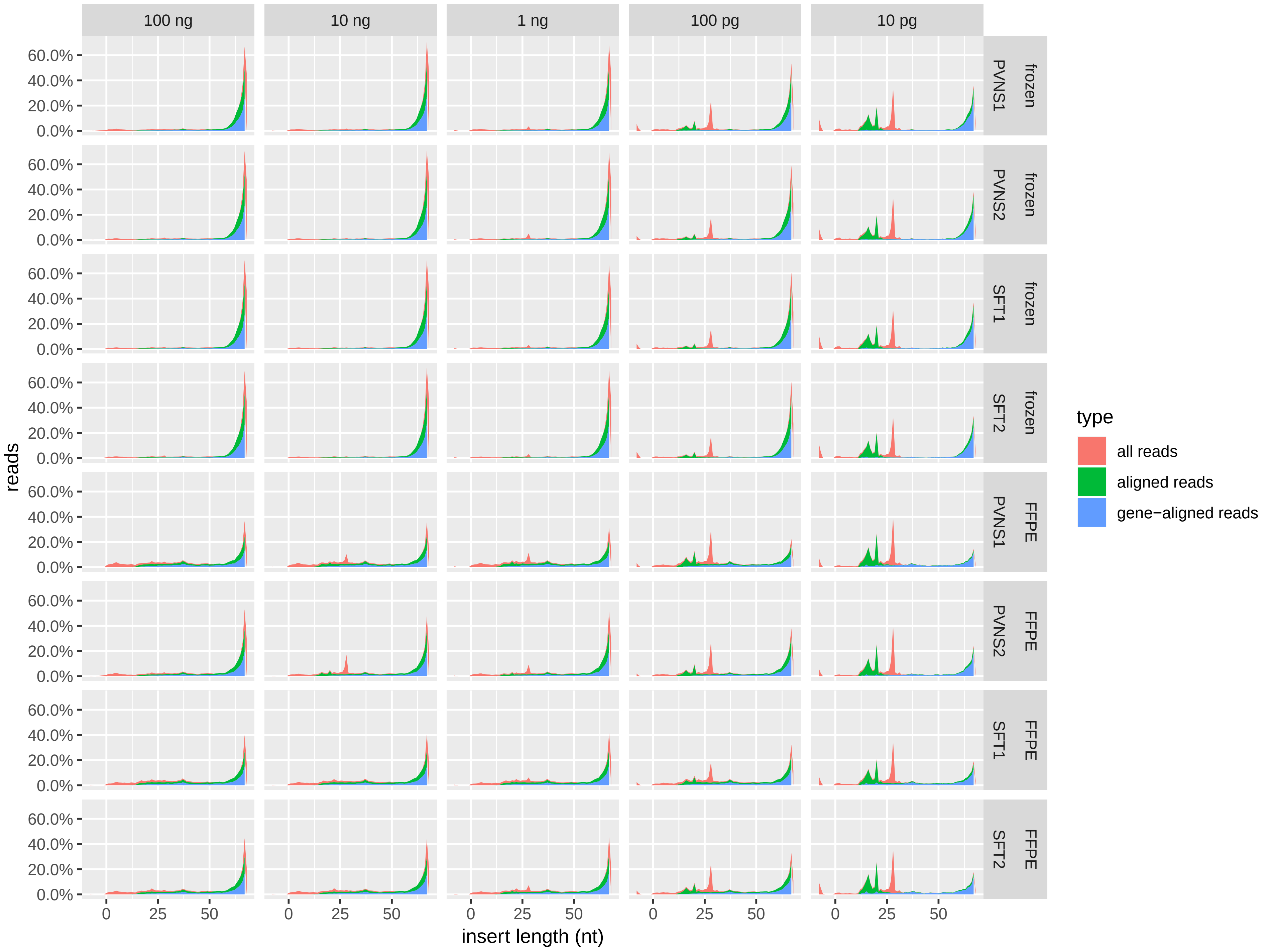

100 ng
10 ng
1 ng
100 pg
10 pg
PVNS1 frozen
PVNS2 frozen
SFT1 frozen
SFT2 frozen
PVNS1 FFPE
PVNS2 FFPE
SFT1 FFPE
SFT2 FFPE
reads
60.0%
40.0%
20.0%
0.0%
0
25
50
insert length (nt)
type
all reads
aligned reads
gene−aligned reads
