# Supplementary material for: Gene expression profiling of single cells from archival tissue with laser-capture microdissection and Smart-3SEQ
Source: Genome Res. 2019 Nov;29(11):1816–25. doi: 10.1101/gr.234807.118 (PMC6836736; doi:10.1101/gr.234807.118)
Supplement: Supplemental Material [file supp_gr.234807.118_Supplemental_Code.zip › Supplemental_Code.tar!/fig/ffpe-lcm_tsne.pdf]

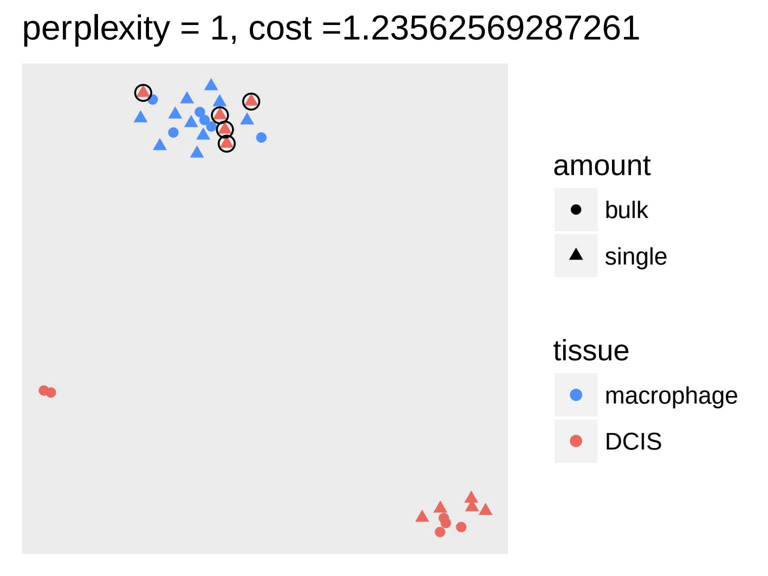
perplexity = 1, cost =1.23562569287261
amount
bulk
single
tissue
macrophage
DCIS

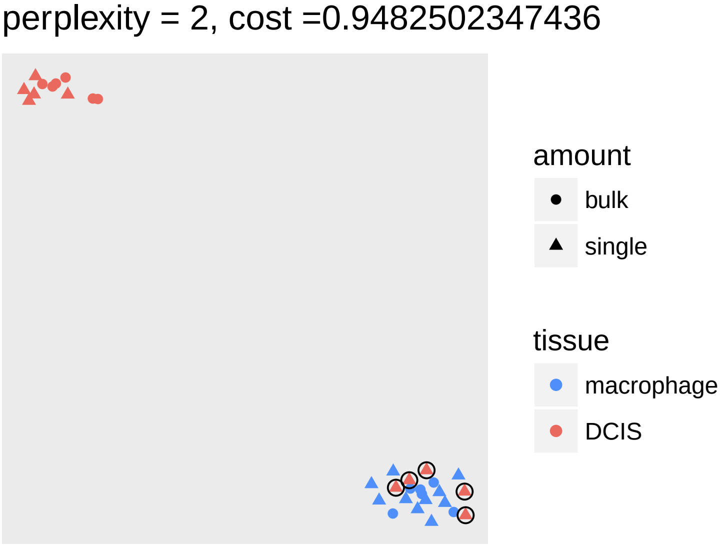
perplexity = 2, cost =0.9482502347436
amount
bulk
single
tissue
macrophage
DCIS

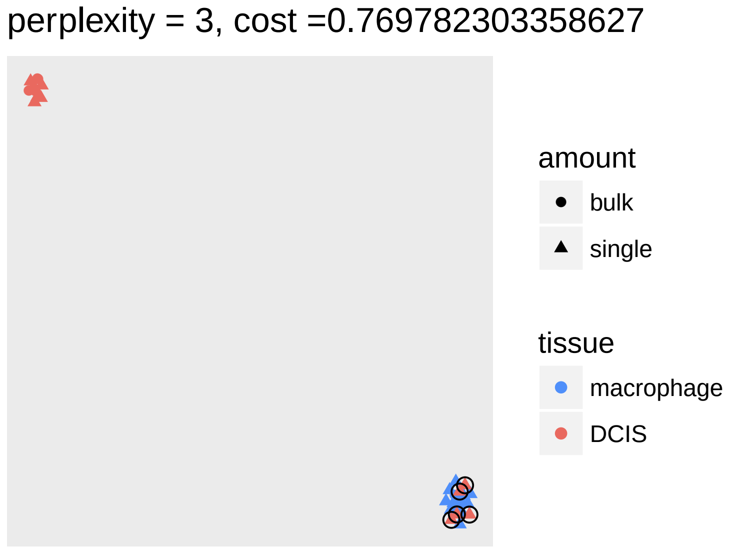
perplexity = 3, cost =0.769782303358627
amount
bulk
single
tissue
macrophage
DCIS

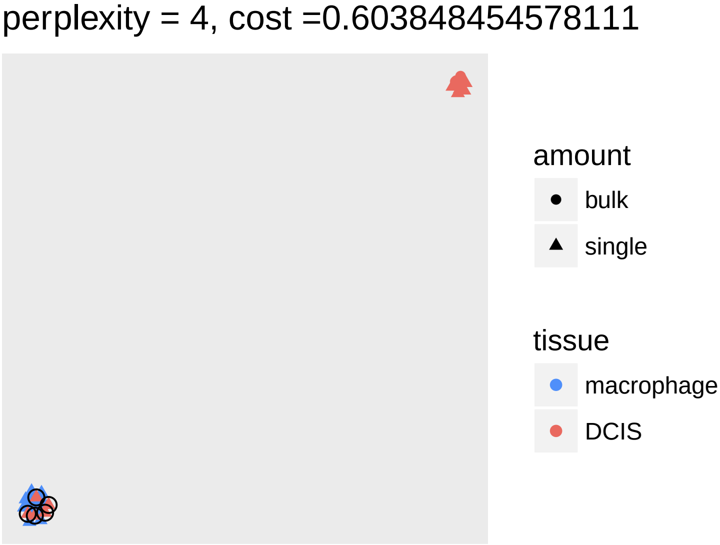
perplexity = 4, cost =0.603848454578111
amount
bulk
single
tissue
macrophage
DCIS

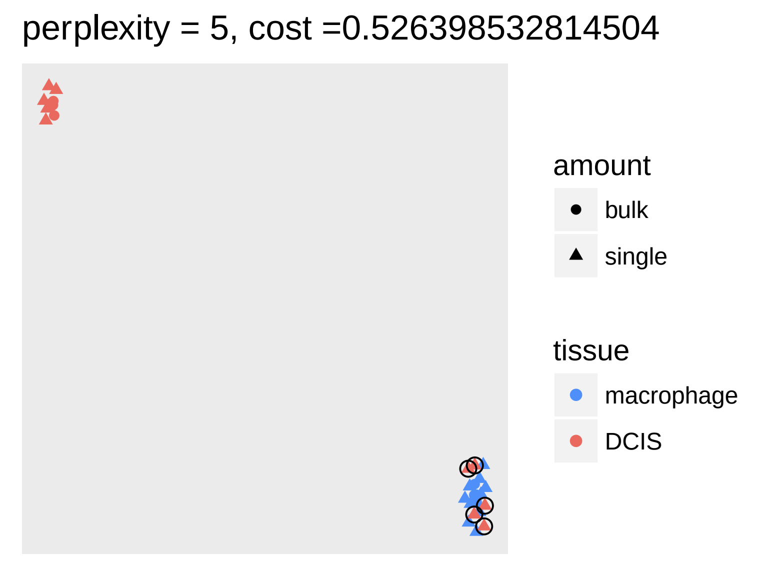

perplexity = 5, cost =0.526398532814504
amount
bulk
single
tissue
macrophage
DCIS

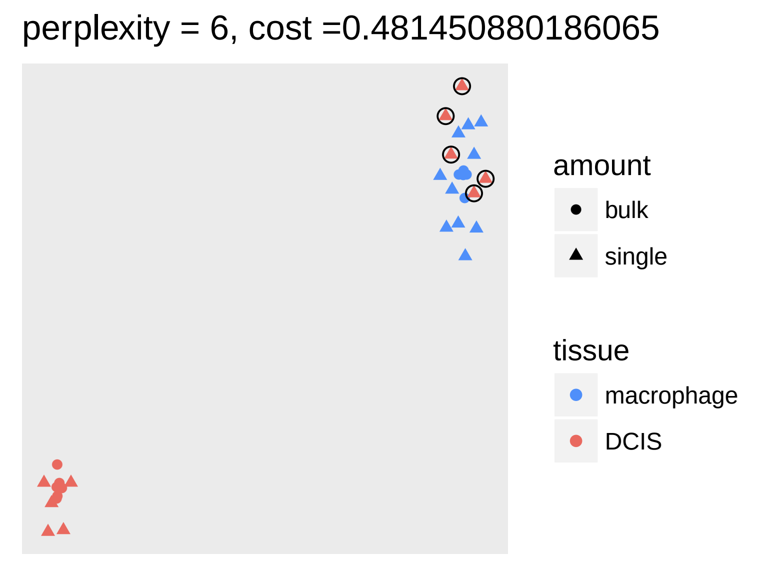
perplexity = 6, cost =0.481450880186065
amount
bulk
single
tissue
macrophage
DCIS

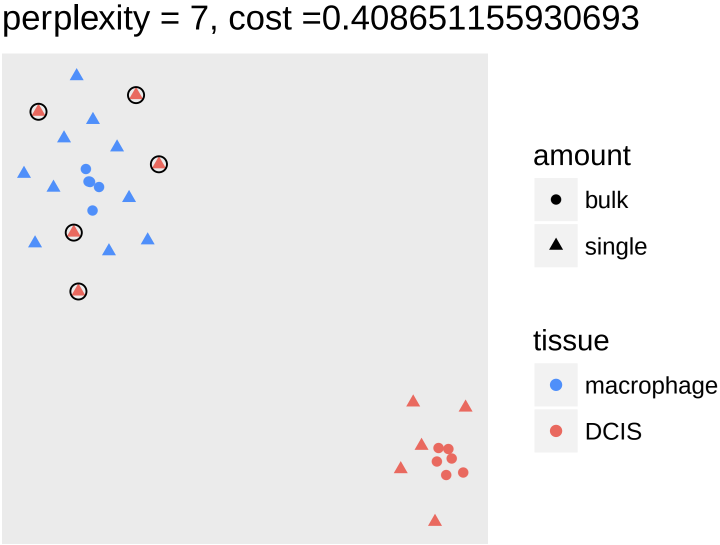
perplexity = 7, cost =0.408651155930693
amount
bulk
single
tissue
macrophage
DCIS

perplexity = 8, cost =0.347688497867533

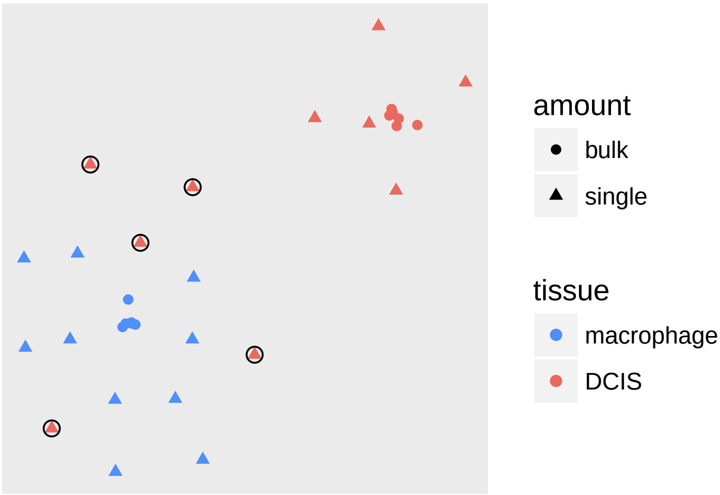

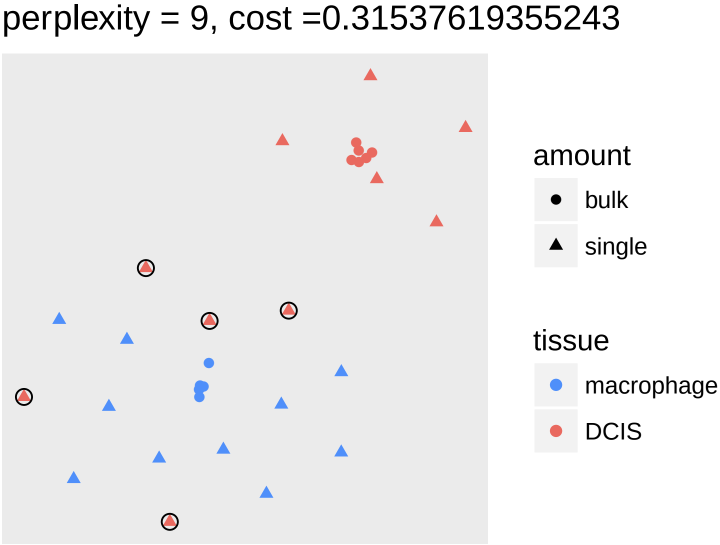
perplexity = 9, cost =0.31537619355243
amount
bulk
single
tissue
macrophage
DCIS

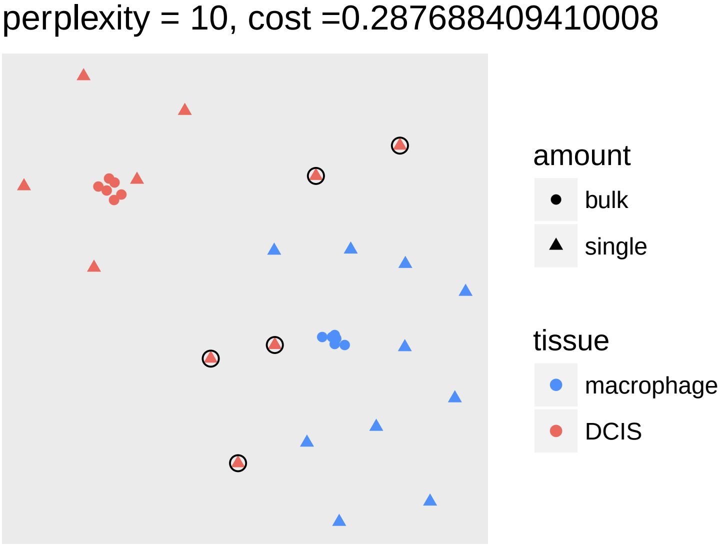
perplexity = 10, cost =0.287688409410008
amount
bulk
single
tissue
macrophage
DCIS
