# Supplementary material for: Gene expression profiling of single cells from archival tissue with laser-capture microdissection and Smart-3SEQ
Source: Genome Res. 2019 Nov;29(11):1816–25. doi: 10.1101/gr.234807.118 (PMC6836736; doi:10.1101/gr.234807.118)
Supplement: Supplemental Material [file supp_gr.234807.118_Supplemental_Code.zip › Supplemental_Code.tar!/fig/marker_genes_ffpe-lcm2.pdf]

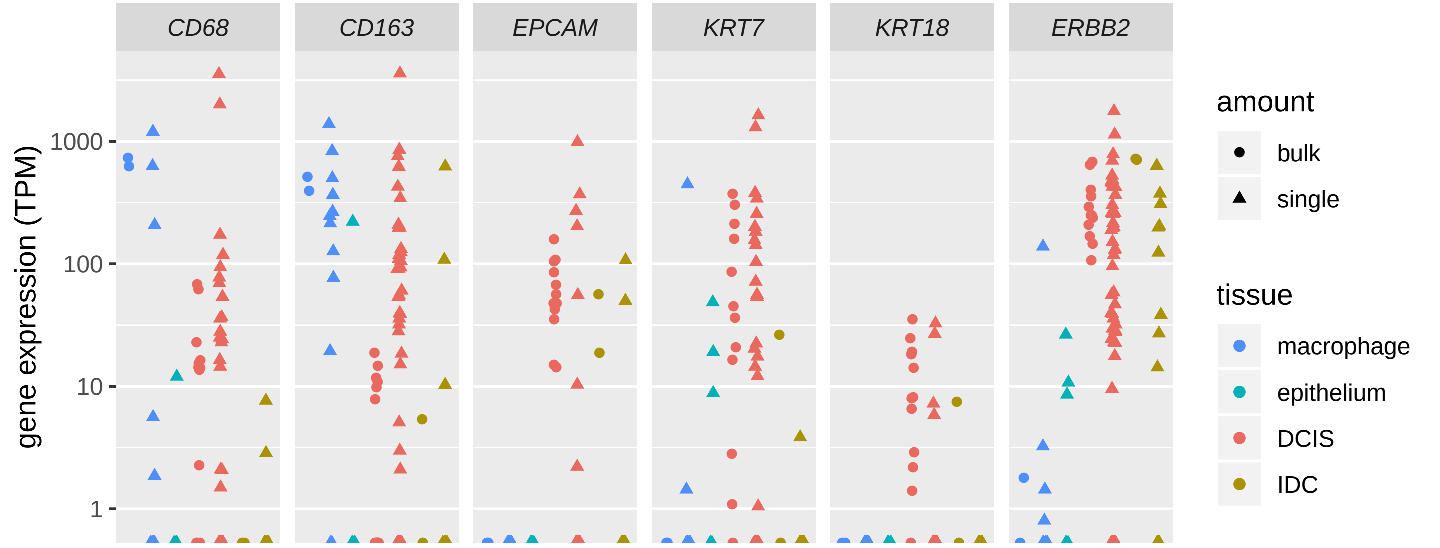

CD68
CD163
EPCAM
KRT7
KRT18
ERBB2
gene expression (TPM)
1000
100
10
1
amount
bulk
single
tissue
macrophage
epithelium
DCIS
IDC
