# Supplementary material for: Gene expression profiling of single cells from archival tissue with laser-capture microdissection and Smart-3SEQ
Source: Genome Res. 2019 Nov;29(11):1816–25. doi: 10.1101/gr.234807.118 (PMC6836736; doi:10.1101/gr.234807.118)
Supplement: Supplemental Material [file supp_gr.234807.118_Supplemental_Code.zip › Supplemental_Code.tar!/fig/insert_lengths_ref-rna.pdf]

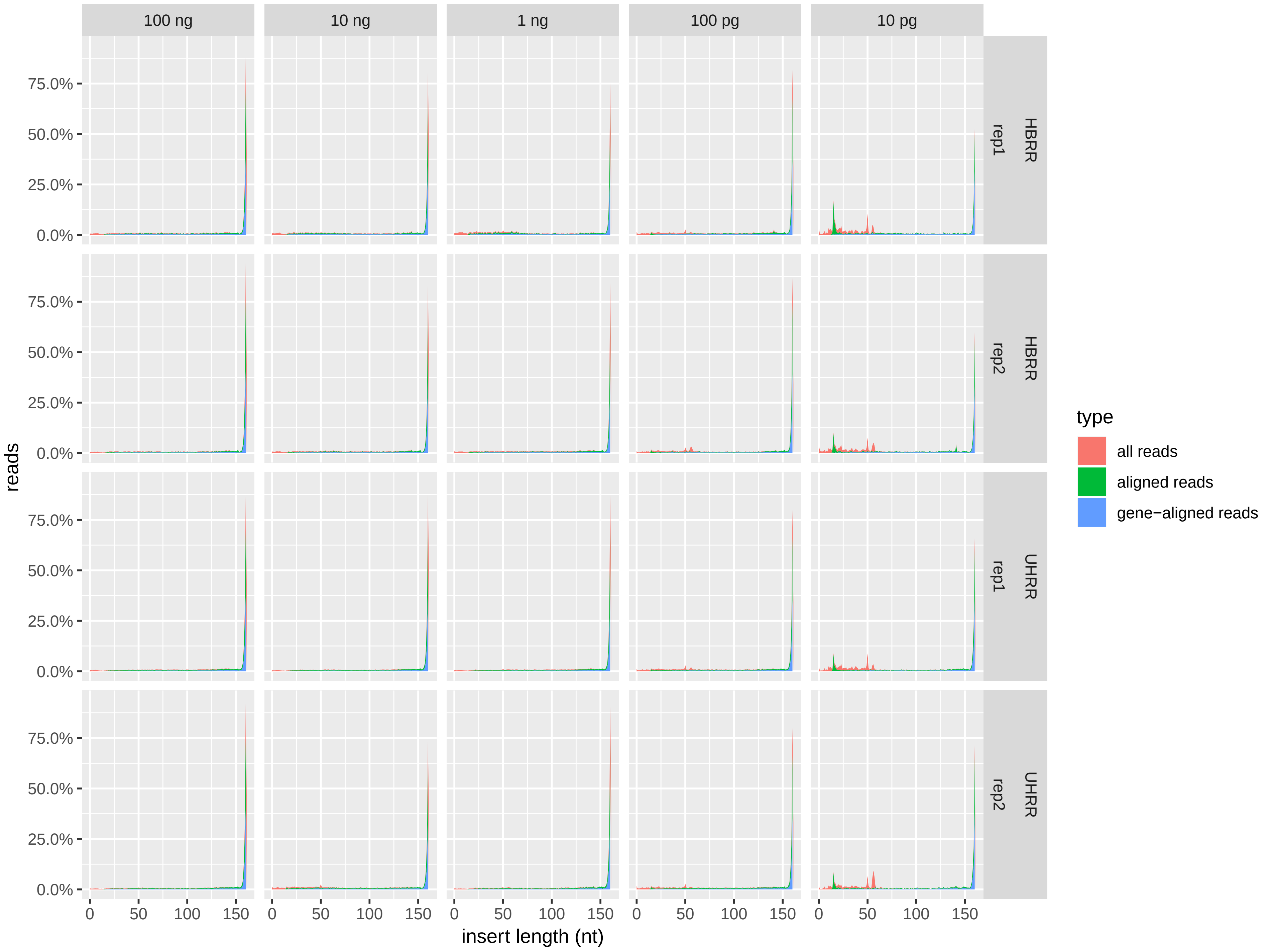

100 ng
10 ng
1 ng
100 pg
10 pg
HBRR rep1
HBRR rep2
UHRR rep1
UHRR rep2
75.0%
50.0%
25.0%
0.0%
reads
0
50
100
150
insert length (nt)
type
all reads
aligned reads
gene−aligned reads
