# Supplementary material for: Gene expression profiling of single cells from archival tissue with laser-capture microdissection and Smart-3SEQ
Source: Genome Res. 2019 Nov;29(11):1816–25. doi: 10.1101/gr.234807.118 (PMC6836736; doi:10.1101/gr.234807.118)
Supplement: Supplemental Material [file supp_gr.234807.118_Supplemental_Code.zip › Supplemental_Code.tar!/fig/compare_sybr_taqman.pdf]

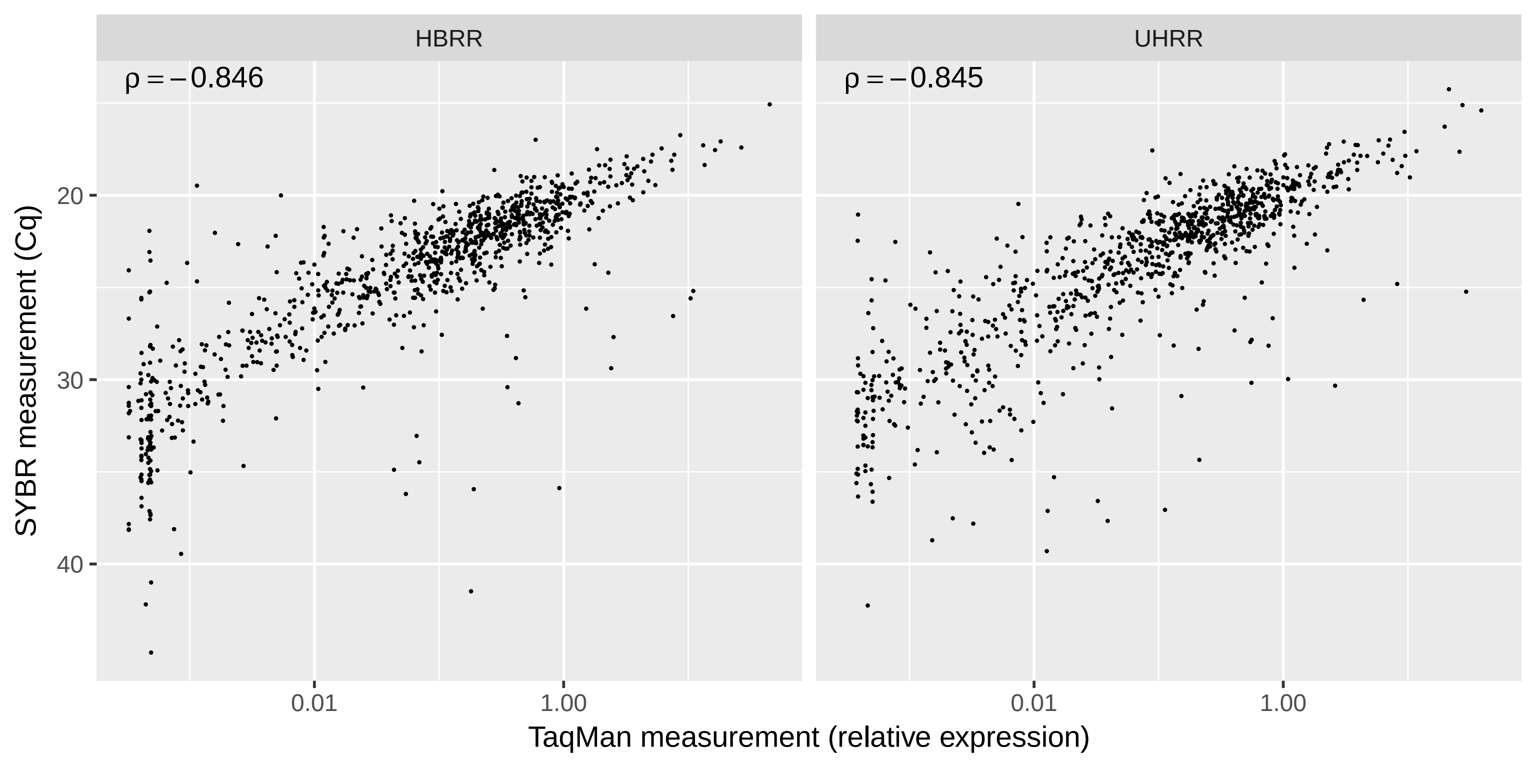
HBRR
UHRR
ρ = −0.846
ρ = −0.845
20
30
40
SYBR measurement (Cq)
0.01
1.00
0.01
1.00
TaqMan measurement (relative expression)
