# Supplementary material for: Gene expression profiling of single cells from archival tissue with laser-capture microdissection and Smart-3SEQ
Source: Genome Res. 2019 Nov;29(11):1816–25. doi: 10.1101/gr.234807.118 (PMC6836736; doi:10.1101/gr.234807.118)
Supplement: Supplemental Material [file supp_gr.234807.118_Supplemental_Code.zip › Supplemental_Code.tar!/fig/stdcrv_seqc.pdf]

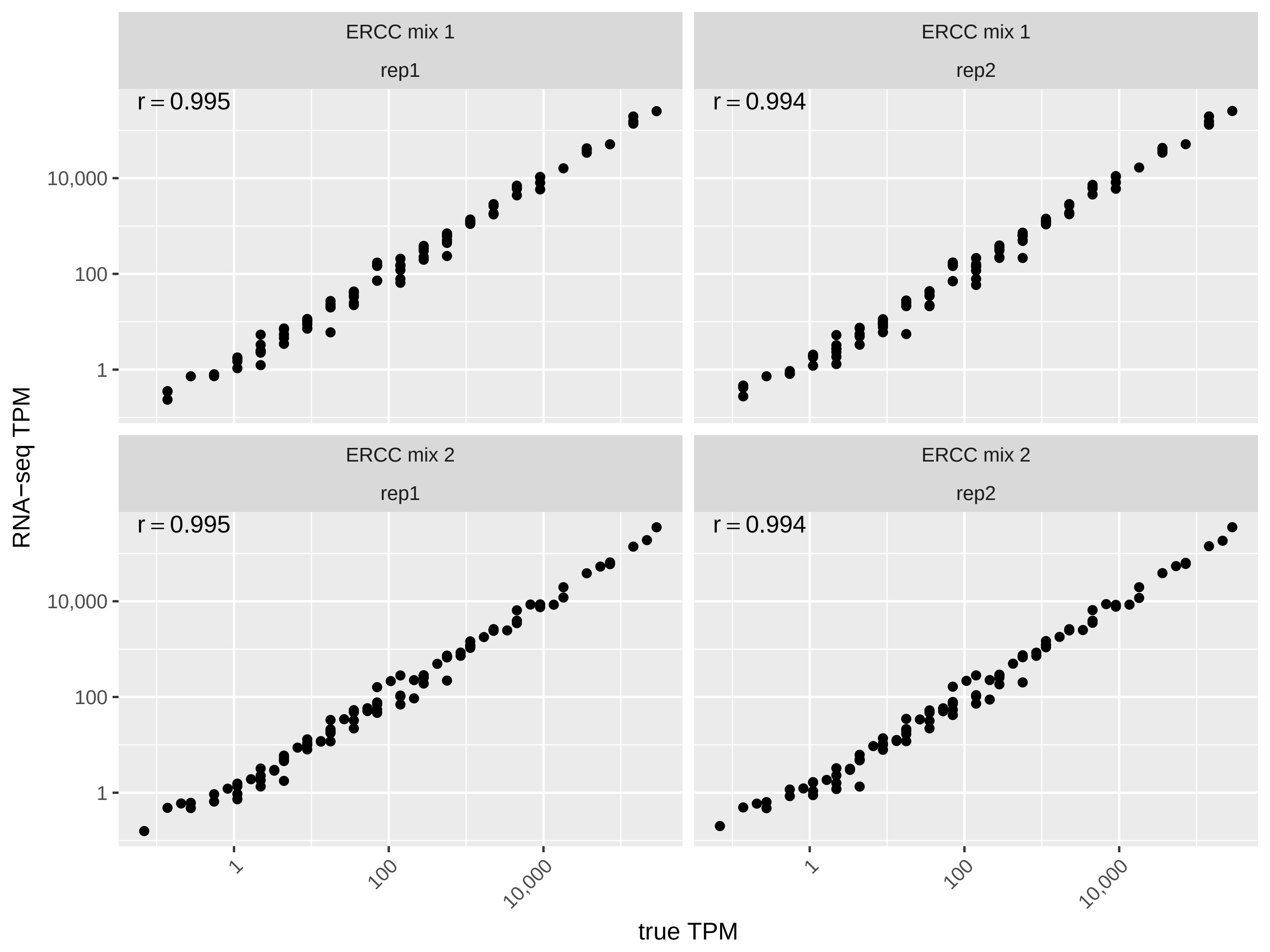
ERCC mix 1
rep1
r = 0.995
ERCC mix 1
rep2
r = 0.994
ERCC mix 2
rep1
r = 0.995
ERCC mix 2
rep2
r = 0.994
10,000
100
1
RNA−seq TPM
1
100
10,000
true TPM

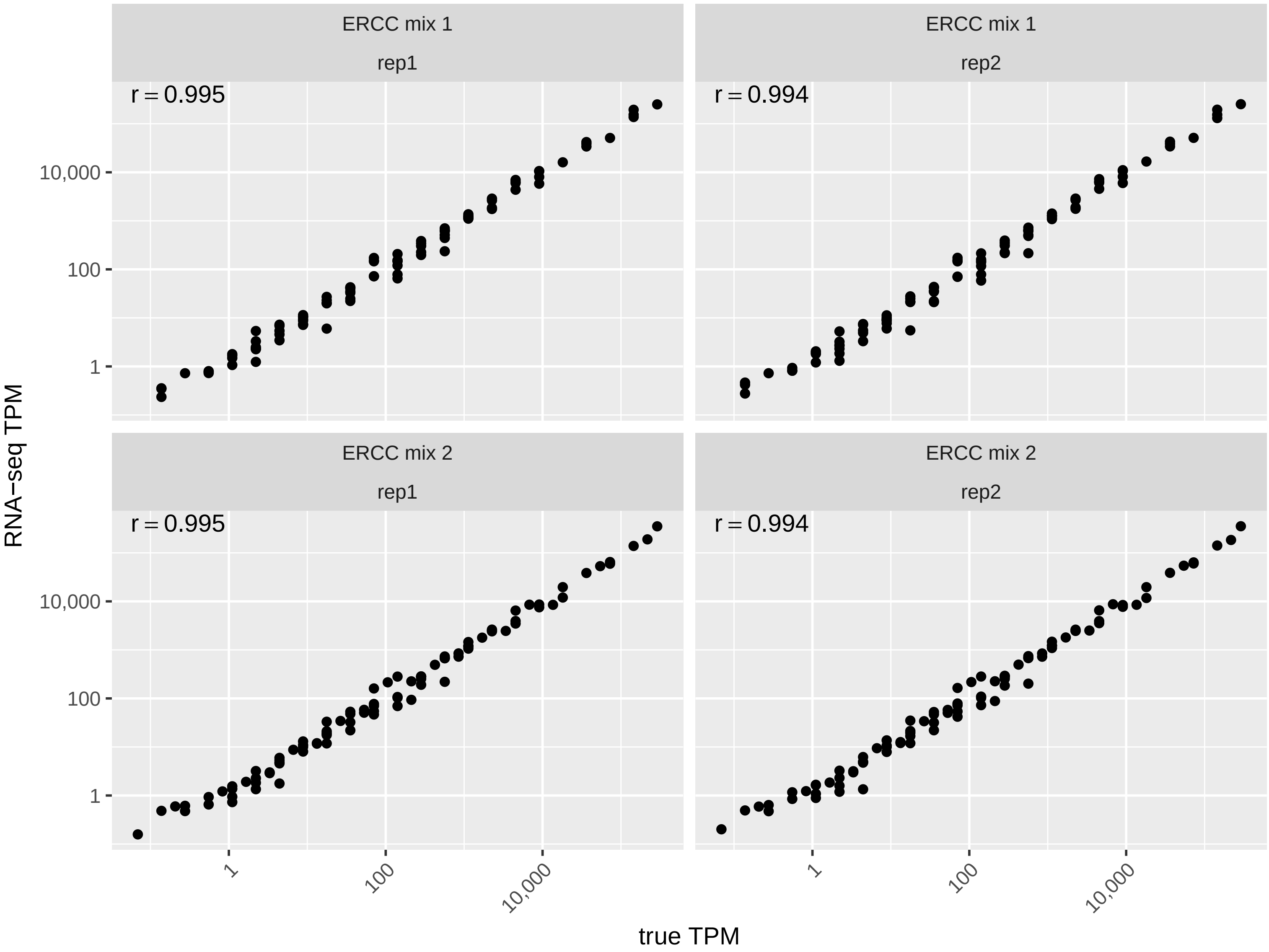

ERCC mix 1
rep1
ERCC mix 1
rep2
ERCC mix 2
rep1
ERCC mix 2
rep2
r = 0.995
r = 0.994
r = 0.995
r = 0.994
10,000
100
1
RNA−seq TPM
true TPM
