# Supplementary material for: Gene expression profiling of single cells from archival tissue with laser-capture microdissection and Smart-3SEQ
Source: Genome Res. 2019 Nov;29(11):1816–25. doi: 10.1101/gr.234807.118 (PMC6836736; doi:10.1101/gr.234807.118)
Supplement: Supplemental Material [file supp_gr.234807.118_Supplemental_Code.zip › Supplemental_Code.tar!/fig/replicates.pdf]

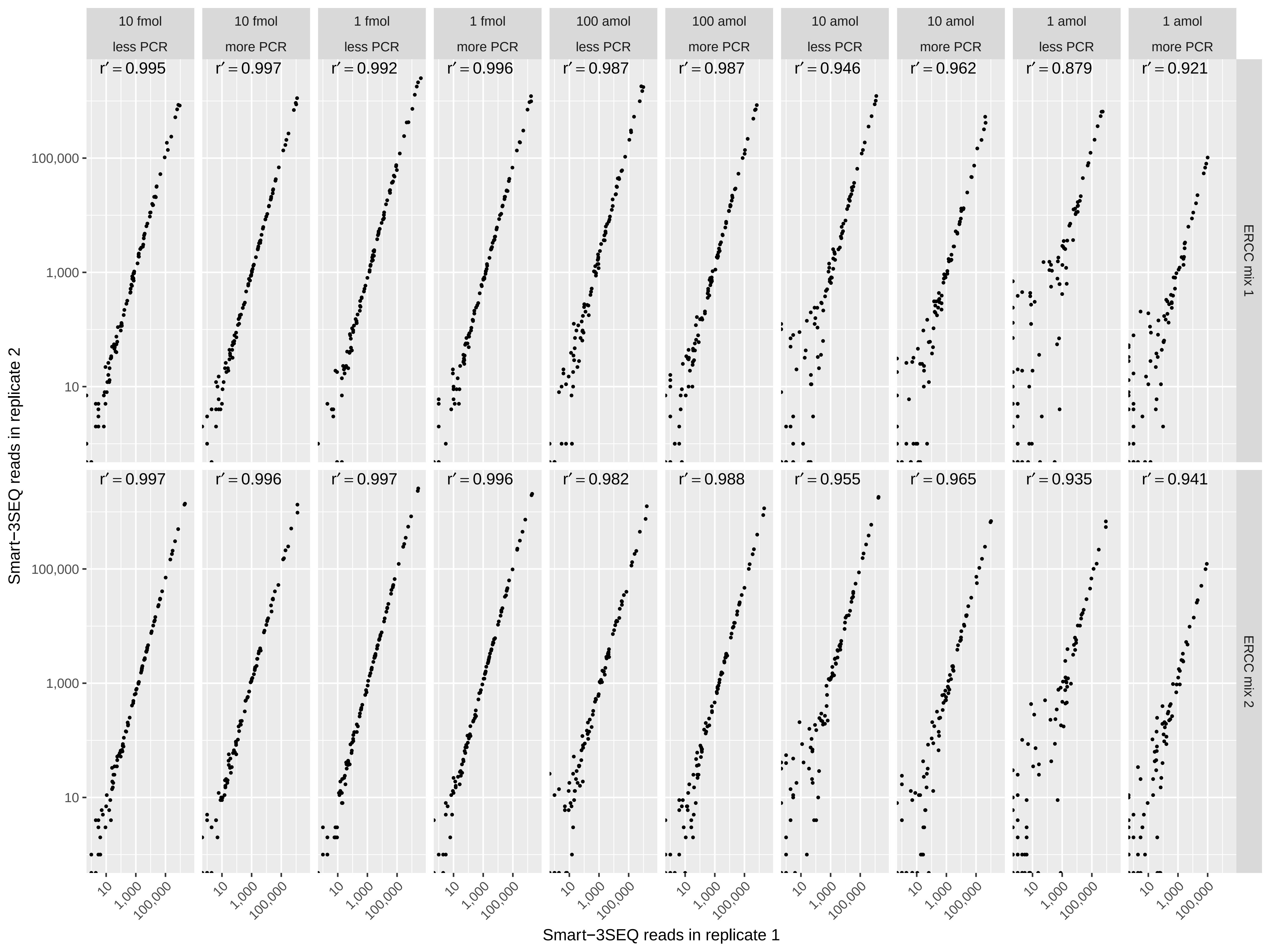
10 fmol less PCR
10 fmol more PCR
1 fmol less PCR
1 fmol more PCR
100 amol less PCR
100 amol more PCR
10 amol less PCR
10 amol more PCR
1 amol less PCR
1 amol more PCR
ERCC mix 1
ERCC mix 2
r′ = 0.995
r′ = 0.997
r′ = 0.992
r′ = 0.996
r′ = 0.987
r′ = 0.987
r′ = 0.946
r′ = 0.962
r′ = 0.879
r′ = 0.921
r′ = 0.997
r′ = 0.996
r′ = 0.997
r′ = 0.996
r′ = 0.982
r′ = 0.988
r′ = 0.955
r′ = 0.965
r′ = 0.935
r′ = 0.941
10
1,000
100,000
Smart−3SEQ reads in replicate 2
Smart−3SEQ reads in replicate 1
