# Supplementary material for: Gene expression profiling of single cells from archival tissue with laser-capture microdissection and Smart-3SEQ
Source: Genome Res. 2019 Nov;29(11):1816–25. doi: 10.1101/gr.234807.118 (PMC6836736; doi:10.1101/gr.234807.118)
Supplement: Supplemental Material [file supp_gr.234807.118_Supplemental_Code.zip › Supplemental_Code.tar!/fig/smart-3seq_diagram_simplified.pdf]

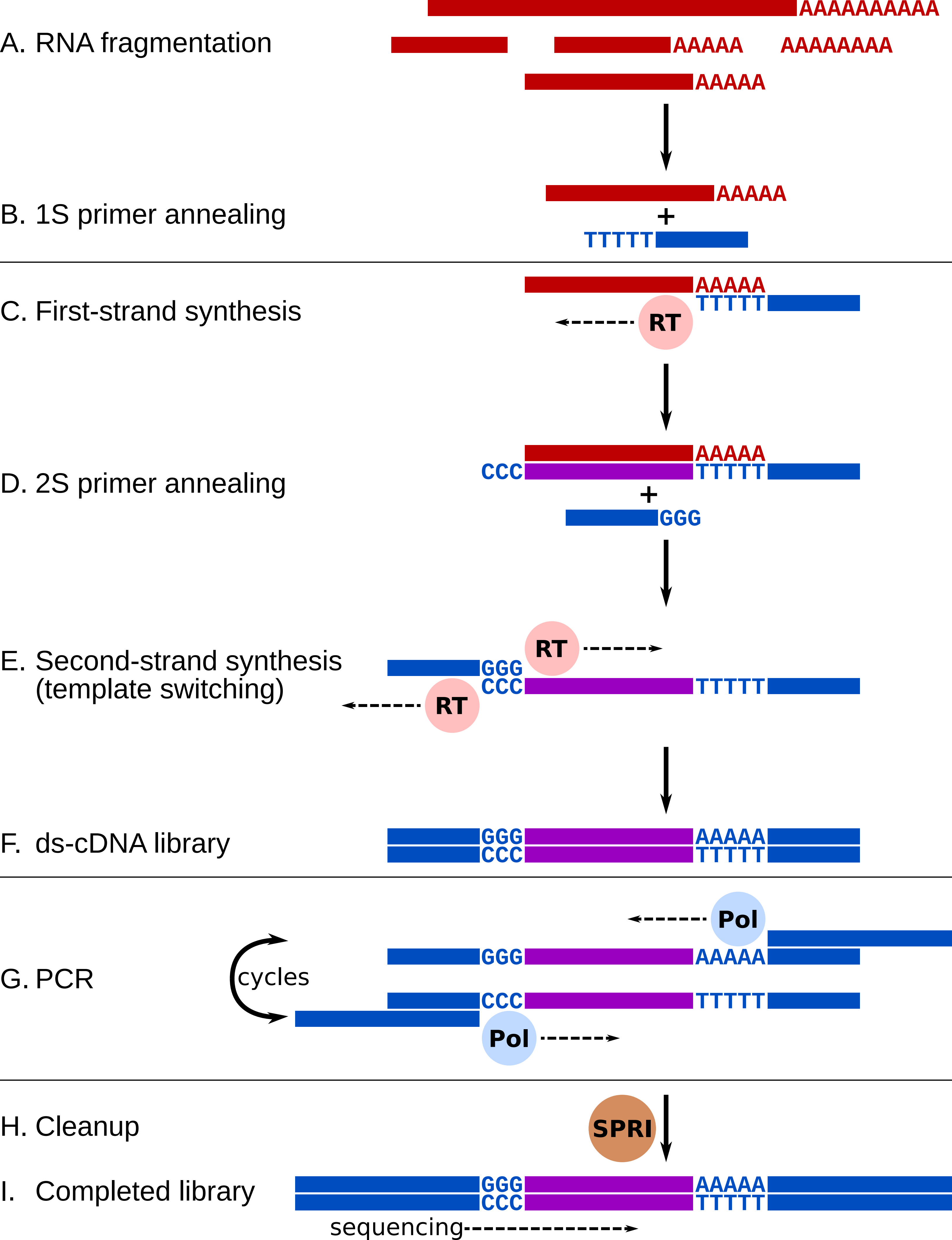
A. RNA fragmentation
AAAAAAAAAAA
AAAAA
AAAAAAAA
AAAAA
B. 1S primer annealing
AAAAA
+
TTTTT
C. First-strand synthesis
AAAAA
TTTTT
RT
D. 2S primer annealing
AAAAA
CCC
TTTTT
+
GGG
E. Second-strand synthesis
(template switching)
RT
GGG
CCC
TTTTT
RT
F. ds-cDNA library
GGG
AAAAA
CCC
TTTTT
G. PCR
Pol
cycles
GGG
AAAAA
CCC
TTTTT
Pol
H. Cleanup
SPRI
I. Completed library
GGG
AAAAA
CCC
TTTTT
sequencing
