# Supplementary material for: Gene expression profiling of single cells from archival tissue with laser-capture microdissection and Smart-3SEQ
Source: Genome Res. 2019 Nov;29(11):1816–25. doi: 10.1101/gr.234807.118 (PMC6836736; doi:10.1101/gr.234807.118)
Supplement: Supplemental Material [file supp_gr.234807.118_Supplemental_Code.zip › Supplemental_Code.tar!/fig/expt_design_ref-rna.pdf]

**Human Brain Reference RNA + ERCC mix 1 or Universal Human Reference RNA + ERCC mix 2**	100 ng + 1/500 ERCC	→	10 ng + 1/5000 ERCC	→	1 ng + 1/50000 ERCC	→	100 pg + 1/500000 ERCC	→	10 pg + 1/5000000 ERCC	No RNA
**PCR cycles**	8		12		15		19		22	22
