# Supplementary material for: Gene expression profiling of single cells from archival tissue with laser-capture microdissection and Smart-3SEQ
Source: Genome Res. 2019 Nov;29(11):1816–25. doi: 10.1101/gr.234807.118 (PMC6836736; doi:10.1101/gr.234807.118)
Supplement: Supplemental Material [file supp_gr.234807.118_Supplemental_Code.zip › Supplemental_Code.tar!/fig/read_sequence.pdf]

CGAGGGGGGCCCATATTTATCACCAGCACTTTGATTCTGTGATGCAGCTGCAAGAGGAGGCCCACCTCAACACCTCCTTTAAGCACTTTATTTTCTTTGTTCAGGAGTTTAATCTGATTGATAGGCGTGAGCTGGCACCTCTTCAAGAATTAATAGAGAAACTTGGATC

AAGCTGGGGAATCGATTGAACCCGGGAGGCGGAGGTAGCAGTGAGCCGAGATCGTGCCACTGCACTCCAGCCTAAGTGACAGAGCTAGACTCCGTCTCAAAAAAAAAAAAAAAAAAAAAAAAAAGAACGGAAGAGCACACGTCGGAACCCCAGGCAAACCACAAACGCC

AGAGGGGGCATCATAGGAGGCTTCATTCACTGATTTCCCCTATTCTCAGGCTACACCCTAGACCAAACCTACGCCAAAATCCATTTCACTATCATATTCATCGGCGTAAATCTAACTTTCTTCCCACAACACTTTCTCGGCCTATCCGGAATGCCCCGACGTTACTCGG

GAACCGGGCAAGCATTTTTTCAACTAATCTTATGTTATTAACCATTTCCTTAAATTCTTCTGGGTCTGCTGACAAAGCATGATCAGGACCTTCCATATTTTTATCTAAGGTAAAGTGCTTCTCAATAACATCCGCTCCTAAGGCAACAGAAACTACTGGGGCGAGTATT

ACAGAGGGAGGGATCTGGCAAGATCGTTAGAGCAAAACAGCCCAGGGAGCCGGAGGAGAGAGTGGAGCCCCGGGCGAGGCTGAGAGCTCCAGGCTCTCGTGGAGGCGGGAGGGACTGGGGGATGCCGCCTGGGGCGGGAGACGGCTGCCTGGCGAGGCCAACAGTCCGG

AACAGGGGGAATACGCTGAAGTAAATCCTTGTTCACTGAAGTCTTTCAATTGAGCTGGTTGAATACTTTGAAAAATGCTCAGTTCTAACTAATGAAATGGATTTCCCAGTAGGGGTTTCTGCATATCACCTGTATAGTAGTTATATGCATATGTTTCTGTGCATGTTCT

AAAGGGGGAAGATTGACTGGGGAGGGCTTAAAATGATTTGGGAAAACAATTGCTTTTGAGGCTCAGTGACAACGGCAAAGATTACAACTCAAAAAAAAAAAAAAAAAAAAAAAAAAAAAAGAACGAAAGAGCACACGCCGGAACACCAGACACACCCAGCACGCGTATG

ACGCGGGGATCCCCATCAAGGTTTGAGTCCACAAAAGTGGACCTCCCTATCATGCTTCCCCTTCCCTCTAGCATGTGGGAAGGGACTGCTGTGAAGAATGACAGATGTGGGGCCTCTGCCAAGTTCTGCATTGCTAAATAAGGGCTTCCTCTGCCTTCTACCTACAGTG

CTAGGGGGAGTGGTGCTGGGAGTGTCGTGGACGCCGTGCCGTTACTCGTAGTCAGGCGGCGGCGCAGGCGGCGGCGGCATAGCGCACAGCGCGCCTTAGCAGCAGCAGCAGCAGCAGCATCGGAGGTACCCCCGCCGTCGCAGCCCCGCGCTGGTGCAGCCACCCC

GGGCTGGGGAGCTTGAAGCAGATGATATCCCGATGCTGACGGCTTACAATAAACGTGATCAAAAAACTGCCTGATTTTATACCGACCGCCGGAAGGGATCACATTATGGTCAGTGCGAAATTTGAGGACGACGCTGCAGCCTTTAAAGAAGCGATTCAGCGCTATTTTC

25 50 75 100 125 150
