# Supplementary material for: Gene expression profiling of single cells from archival tissue with laser-capture microdissection and Smart-3SEQ
Source: Genome Res. 2019 Nov;29(11):1816–25. doi: 10.1101/gr.234807.118 (PMC6836736; doi:10.1101/gr.234807.118)
Supplement: Supplemental Material [file supp_gr.234807.118_Supplemental_Code.zip › Supplemental_Code.tar!/fig/smart-3seq_diagram.pdf]

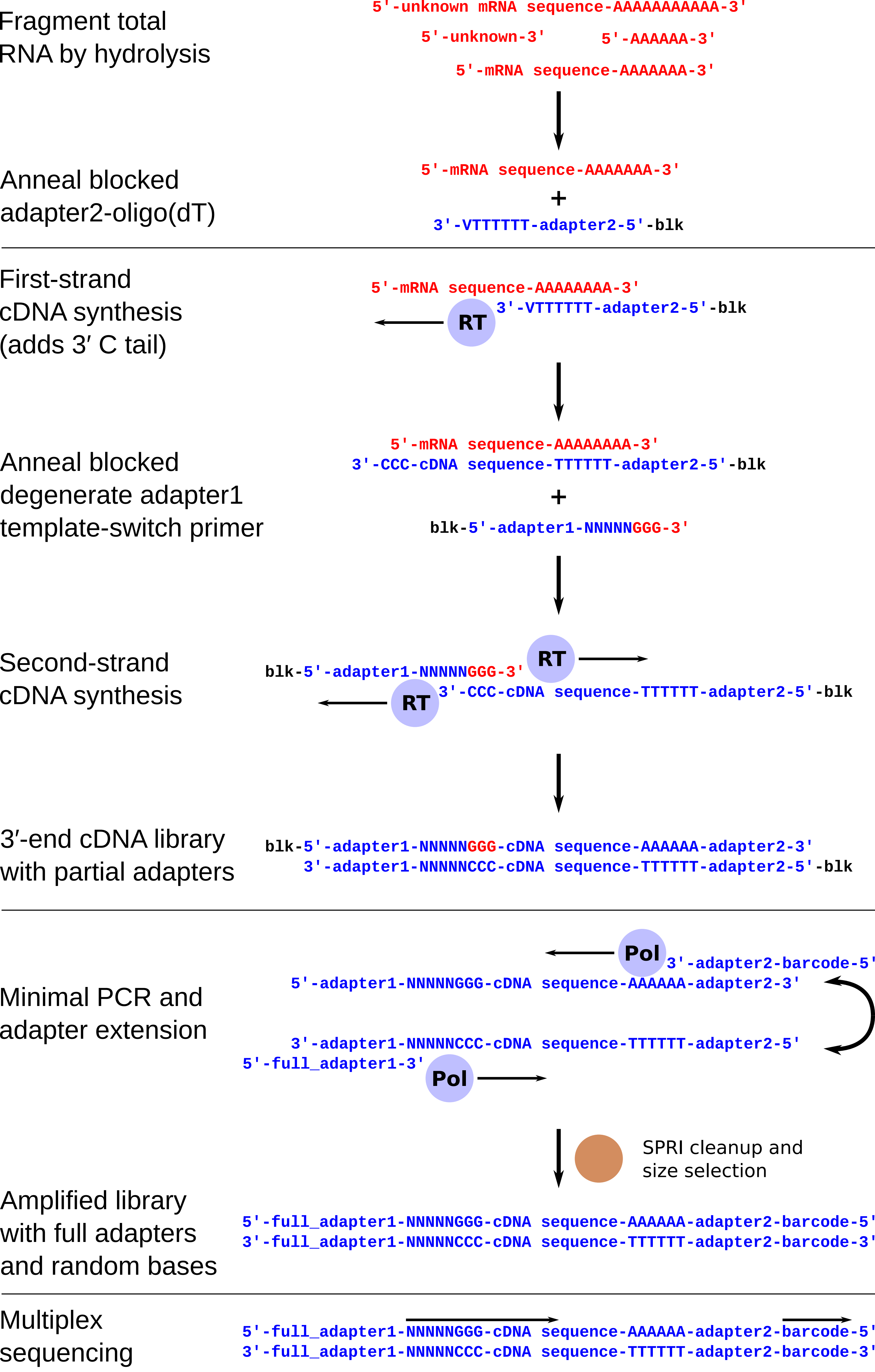
Fragment total RNA by hydrolysis
5'-unknown mRNA sequence-AAAAAAAAAAA-3'
5'-unknown-3'
5'-AAAAAA-3'
5'-mRNA sequence-AAAAAAA-3'
Anneal blocked adapter2-oligo(dT)
5'-mRNA sequence-AAAAAAA-3'
+
3'-VTTTTTT-adapter2-5'-blk
First-strand cDNA synthesis (adds 3′ C tail)
5'-mRNA sequence-AAAAAAAA-3'
RT
3'-VTTTTTT-adapter2-5'-blk
Anneal blocked degenerate adapter1 template-switch primer
5'-mRNA sequence-AAAAAAAA-3'
3'-CCC-cDNA sequence-TTTTTT-adapter2-5'-blk
+
blk-5'-adapter1-NNNNNGGG-3'
Second-strand cDNA synthesis
RT
blk-5'-adapter1-NNNNNGGG-3'
RT
3'-CCC-cDNA sequence-TTTTTT-adapter2-5'-blk
3′-end cDNA library with partial adapters
blk-5'-adapter1-NNNNNGGG-cDNA sequence-AAAAAA-adapter2-3'
3'-adapter1-NNNNNCCC-cDNA sequence-TTTTTT-adapter2-5'-blk
Minimal PCR and adapter extension
Pol
3'-adapter2-barcode-5'
5'-adapter1-NNNNNGGG-cDNA sequence-AAAAAA-adapter2-3'
3'-adapter1-NNNNNCCC-cDNA sequence-TTTTTT-adapter2-5'
5'-full_adapter1-3'
Pol
SPRI cleanup and size selection
Amplified library with full adapters and random bases
5'-full_adapter1-NNNNNGGG-cDNA sequence-AAAAAA-adapter2-barcode-5'
3'-full_adapter1-NNNNNCCC-cDNA sequence-TTTTTT-adapter2-barcode-3'
Multiplex sequencing
5'-full_adapter1-NNNNNGGG-cDNA sequence-AAAAAA-adapter2-barcode-5'
3'-full_adapter1-NNNNNCCC-cDNA sequence-TTTTTT-adapter2-barcode-3'
