# Supplementary material for: Gene expression profiling of single cells from archival tissue with laser-capture microdissection and Smart-3SEQ
Source: Genome Res. 2019 Nov;29(11):1816–25. doi: 10.1101/gr.234807.118 (PMC6836736; doi:10.1101/gr.234807.118)
Supplement: Supplemental Material [file supp_gr.234807.118_Supplemental_Code.zip › Supplemental_Code.tar!/fig/smart-3seq_sequence_diagram.pdf]

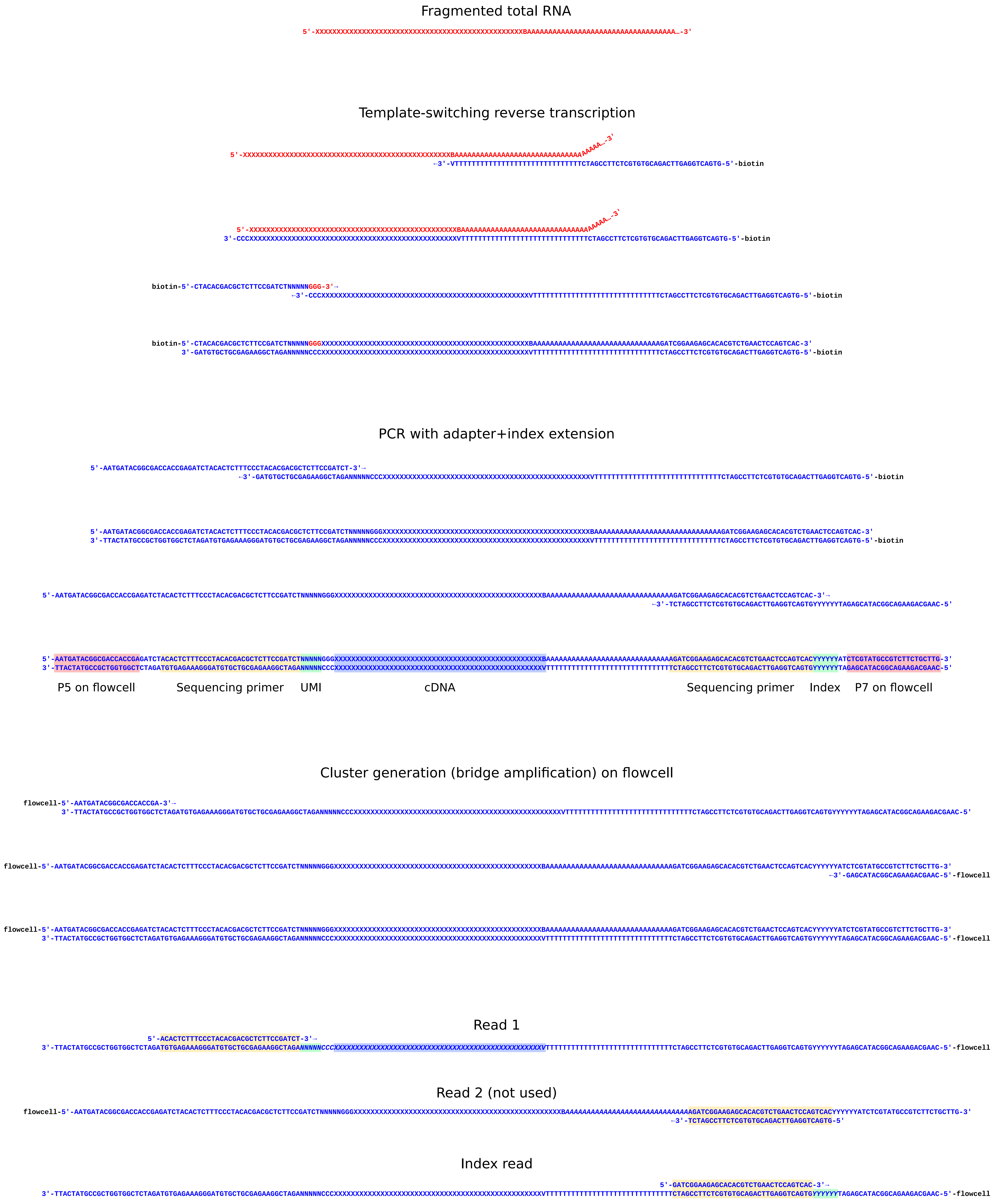

Fragmented total RNA
Template-switching reverse transcription
PCR with adapter+index extension
5'-AATGATACGGCGACCACCGAGATCTACACTCTTTCCCTACACGACGCTCTTCCGATCT-3'→
←3'-TCTAGCCTTCTCGTGTGCAGACTTGAGGTCAGTGYYYYYYTAGAGCATACGGCAGAAGACGAAC-5'
P5 on flowcell
Sequencing primer
UMI
cDNA
Sequencing primer
Index
P7 on flowcell
Cluster generation (bridge amplification) on flowcell
flowcell-5'-AATGATACGGCGACCACCGA-3'→
←3'-GAGCATACGGCAGAAGACGAAC-5'-flowcell
Read 1
5'-ACACTCTTTCCCTACACGACGCTCTTCCGATCT-3'→
Read 2 (not used)
←3'-TCTAGCCTTCTCGTGTGCAGACTTGAGGTCAGTG-5'
Index read
5'-GATCGGAAGAGCACACGTCTGAACTCCAGTCAC-3'→
