# Supplementary material for: Gene expression profiling of single cells from archival tissue with laser-capture microdissection and Smart-3SEQ
Source: Genome Res. 2019 Nov;29(11):1816–25. doi: 10.1101/gr.234807.118 (PMC6836736; doi:10.1101/gr.234807.118)
Supplement: Supplemental Material [file supp_gr.234807.118_Supplemental_Code.zip › Supplemental_Code.tar!/fig/compare_3seq_seqc.pdf]

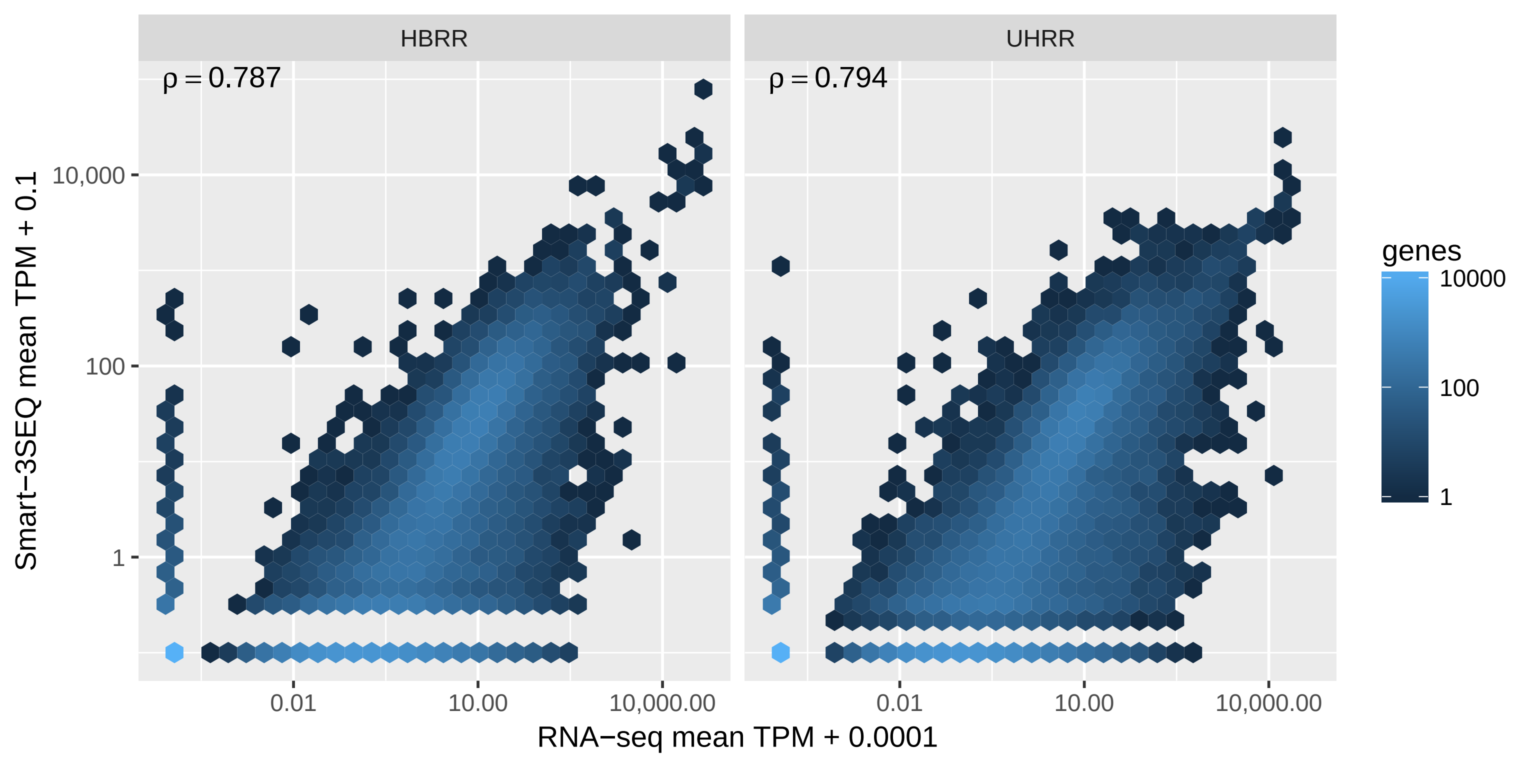
HBRR
UHRR
ρ = 0.787
ρ = 0.794
10,000
100
1
0.01
10.00
10,000.00
Smart−3SEQ mean TPM + 0.1
RNA−seq mean TPM + 0.0001
genes
10000
100
1
