# Supplementary material for: Gene expression profiling of single cells from archival tissue with laser-capture microdissection and Smart-3SEQ
Source: Genome Res. 2019 Nov;29(11):1816–25. doi: 10.1101/gr.234807.118 (PMC6836736; doi:10.1101/gr.234807.118)
Supplement: Supplemental Material [file supp_gr.234807.118_Supplemental_Code.zip › Supplemental_Code.tar!/fig/compare_seqc_sybr.pdf]

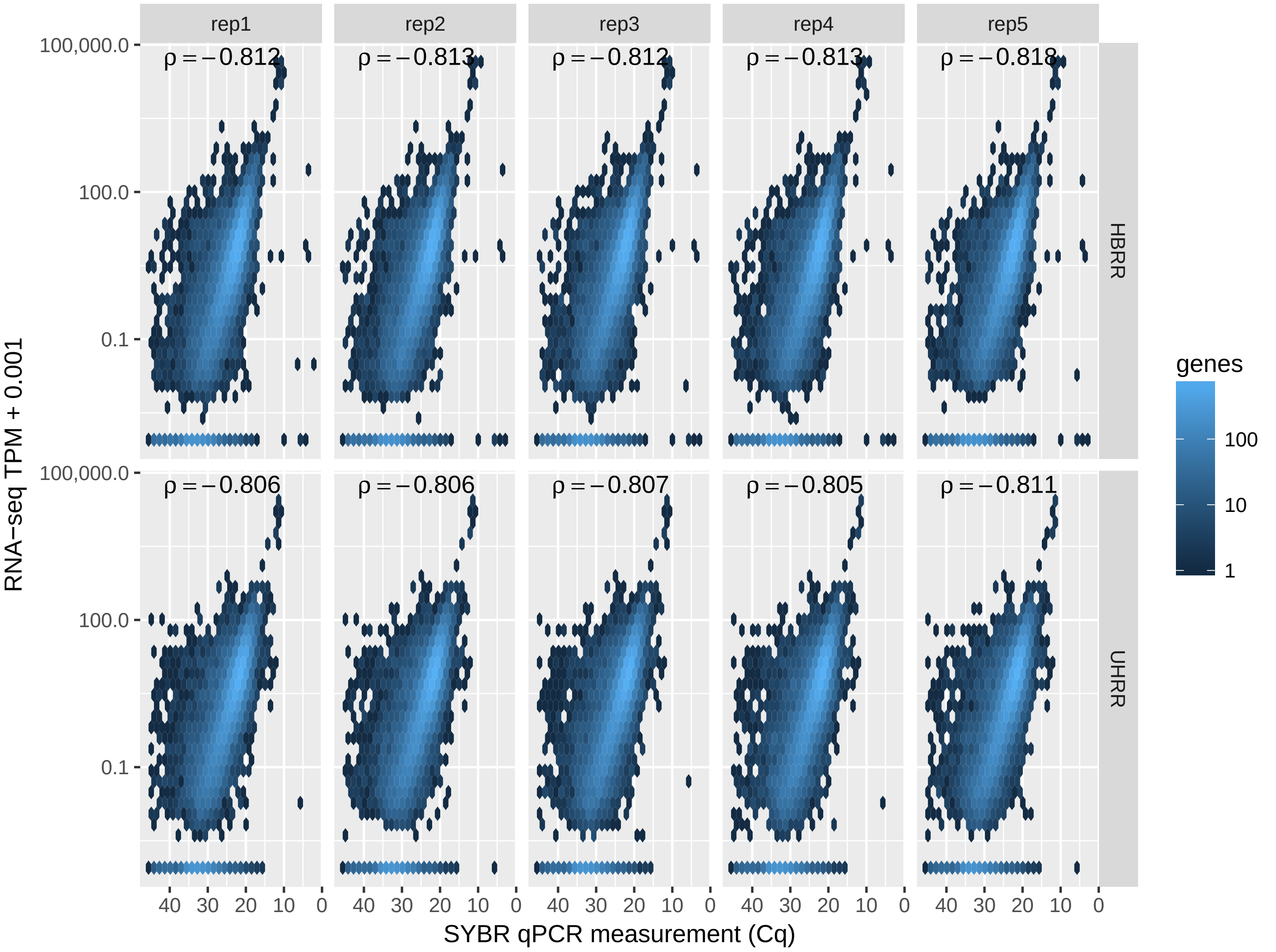
rep1
rep2
rep3
rep4
rep5
HBRR
UHRR
ρ = −0.812
ρ = −0.813
ρ = −0.812
ρ = −0.813
ρ = −0.818
ρ = −0.806
ρ = −0.806
ρ = −0.807
ρ = −0.805
ρ = −0.811
100,000.0
100.0
0.1
40 30 20 10 0
RNA−seq TPM + 0.001
SYBR qPCR measurement (Cq)
genes
100
10
1
