# Supplementary material for: Gene expression profiling of single cells from archival tissue with laser-capture microdissection and Smart-3SEQ
Source: Genome Res. 2019 Nov;29(11):1816–25. doi: 10.1101/gr.234807.118 (PMC6836736; doi:10.1101/gr.234807.118)
Supplement: Supplemental Material [file supp_gr.234807.118_Supplemental_Code.zip › Supplemental_Code.tar!/fig/intron_polyA.pdf]

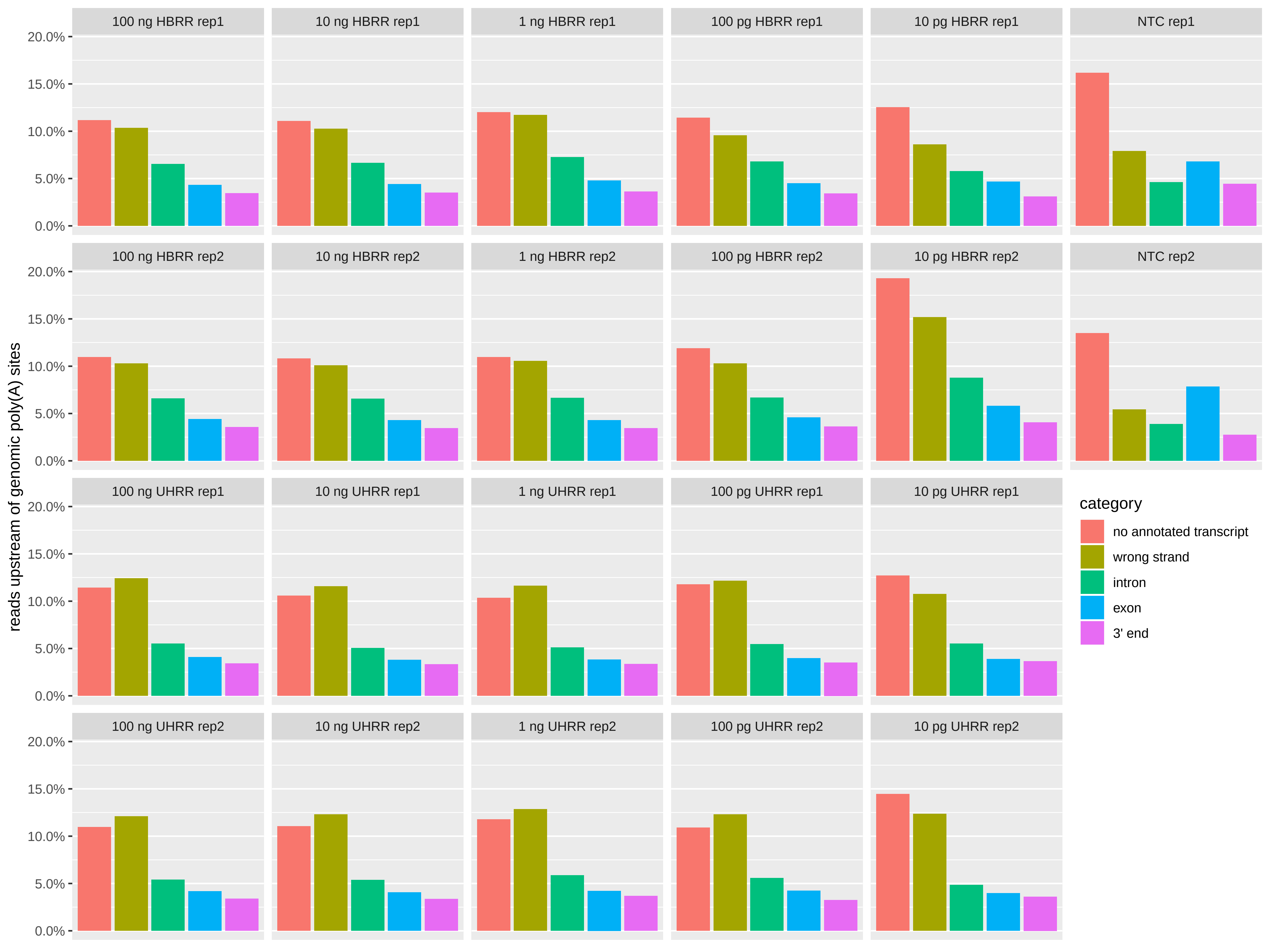

100 ng HBRR rep1
10 ng HBRR rep1
1 ng HBRR rep1
100 pg HBRR rep1
10 pg HBRR rep1
NTC rep1
100 ng HBRR rep2
10 ng HBRR rep2
1 ng HBRR rep2
100 pg HBRR rep2
10 pg HBRR rep2
NTC rep2
100 ng UHRR rep1
10 ng UHRR rep1
1 ng UHRR rep1
100 pg UHRR rep1
10 pg UHRR rep1
100 ng UHRR rep2
10 ng UHRR rep2
1 ng UHRR rep2
100 pg UHRR rep2
10 pg UHRR rep2
reads upstream of genomic poly(A) sites
20.0%
15.0%
10.0%
5.0%
0.0%
category
no annotated transcript
wrong strand
intron
exon
3' end
